# Synthesis of 2-BMIDA Indoles via Heteroannulation: Applications in Drug Scaffold and Natural Product Synthesis

**DOI:** 10.1021/acs.orglett.2c00959

**Published:** 2022-04-15

**Authors:** George E. Bell, James W. B. Fyfe, Eva M. Israel, Alexandra M. Z. Slawin, Matthew Campbell, Allan J. B. Watson

**Affiliations:** † EaStCHEM, School of Chemistry, University of St Andrews, North Haugh, St Andrews, Fife KY16 9ST, United Kingdom; ‡ GlaxoSmithKline, Medicines Research Centre, Gunnels Wood Road, Stevenage, SG1 2NY, United Kingdom

## Abstract

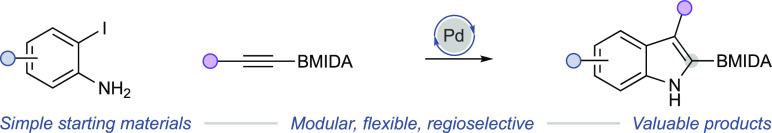

A Pd-catalyzed heteroannulation approach for the synthesis of C2 borylated indoles is reported. The process allows access to highly functionalized 2-borylated indole scaffolds with complete control of regioselectivity. The utility of the process is demonstrated in the synthesis of borylated sulfa drugs and in the concise synthesis of the *Aspidosperma* alkaloid Goniomitine.

Azaheterocycles are prolific in agrochemicals, pharmaceuticals, and natural products. Among the variety of classes, indoles remain a template of enduring prominence.^1^ The academic and industrial utility of this scaffold has inspired the development of numerous methodologies for its construction and functionalization.^2^ Selective functionalization of the indole scaffold has been integral to the development of bioactive compounds (e.g., Scheme 1a), and strategies that allow selective and/or late-stage modification remain a target for methodological development.^3^ On the basis of their wide scope of potential applications and familiarity of use, methods to install boron functional groups have been a particular target for development. These methods include classical strategies based on stoichiometric metalation and reaction with, for example, B(OMe)_3_,^4^ and extend to contemporary approaches using C–H activation^3,5^ and direct borylation with borenium cations (Scheme 1b).^6^

**Scheme 1:**
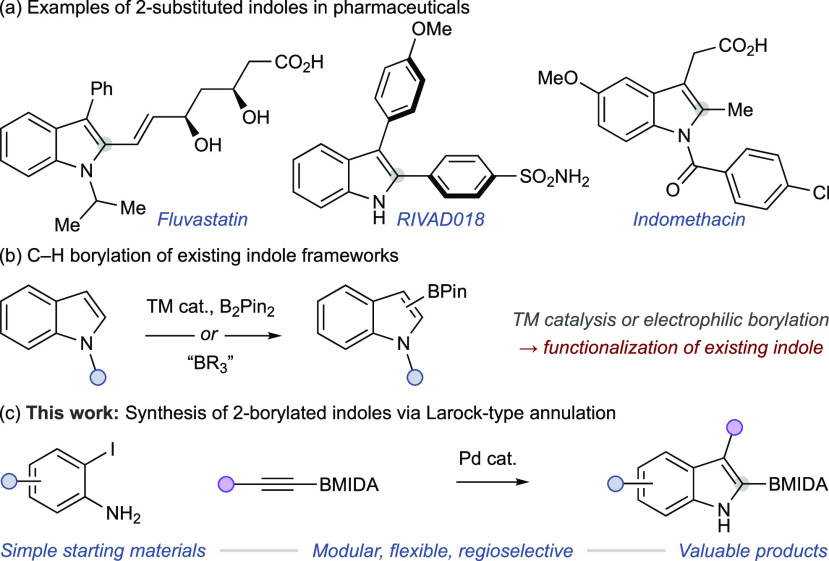
Accessing Borylated Indoles Cat. = catalyst, MIDA = *N*-methyliminodiacetoxy, Pin = pinacolato, TM = transition metal.

These methods rely on borylation of an established indole scaffold and are necessarily constrained by available functionality. Regioselectivity is a key consideration, and examples of these methodologies have demonstrated exquisite selectivity, with others exhibiting lower levels of regiocontrol.

Here we report an alternative approach to the regioselective synthesis of C2-borylated indoles. A Larock-type annulation^7−9^ allows regioselective synthesis of functionalized 2-borylated indoles under mild conditions (Scheme 1c).^10,11^ The process avoids the need for protecting groups on the indole nitrogen and avoids the restrictions imposed by using commercial indole scaffolds. The utility of this approach is demonstrated in the synthesis of drug scaffolds and alkaloid natural products.

Exploration of the annulation began with an initial survey of reaction conditions using 2-iodoaniline (**1a**) and propynyl BMIDA^12,13^ (**2a**) as a benchmark system (Table 1). Optimization provided a system that delivered 2-BMIDA-3-methylindole **3** in good yield (entry 1; for full details, see Supporting Information (SI)). These conditions were equivalent to more standard Larock-type conditions (entry 2); however, the chloride effect^7−9^ could be replicated by the catalytic chloride available from the Pd catalyst, which delivered a small practical advantage. In the absence of chloride, reaction efficiency was ca. 20% lower (entry 3). The main issue that required navigation was compatibility of the reaction conditions with the BMIDA unit. For example, stronger bases led to MIDA hydrolysis^14^ and subsequent protodeboronation^15−17^ lowering the yield of **3** (entry 4).

**Table 1:**
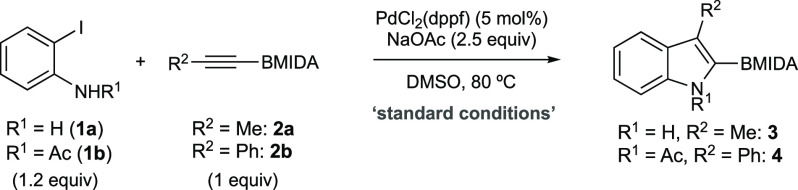
Reaction Development^a^

entry	components	deviation from “standard condtions”	yield (%),^b^ product
1	**1a/2a**		84,^c^ **3**
2	**1a/2a**	Pd(OAc)_2_, LiCl (1 equiv)	83, **3**
3	**1a/2a**	Pd(OAc)_2_	66, **3**
4	**1a/2a**	replace NaOAc with K_2_CO_3_ or K_3_PO_4_	<20%, **3**
5	**1a/2b**		16, **4**
6	**1b/2b**	Pd(OAc)_2_ (10 mol %), LiCl (2 equiv), DMF, 65 °C	60,^c^ **4**

a Reactions performed on 0.2 mmol scale.

b Determined by ^1^H NMR using an internal standard as an average of 2 runs.

c Isolated yield.

Moving from propynyl BMIDA **2a** to phenylacetylenyl BMIDA **2b** was less straightforward than expected. The optimal conditions for **2a** delivered only 16% of 2-BMIDA-3-phenylindole (**4**) when using **2b** (entry 5), and an independent optimization was necessary (see SI). Ultimately, this required the use of *N*-acyl 2-iodoaniline (**1b**) under the more classical Larock conditions for this aryl-substituted alkyne, giving **4** in good yield (entry 6). Acetate was the optimal N-protecting group (see SI). The origin of this difference in reactivity is uncertain, but the increased steric bulk of alkyne **2b** is very likely to dominate,^7−9,18−20^ with electronic effects also a minor contributor.^21,22^ These conditions were subsequently assessed for generality across a series of annulations (Scheme 2).

**Scheme 2:**
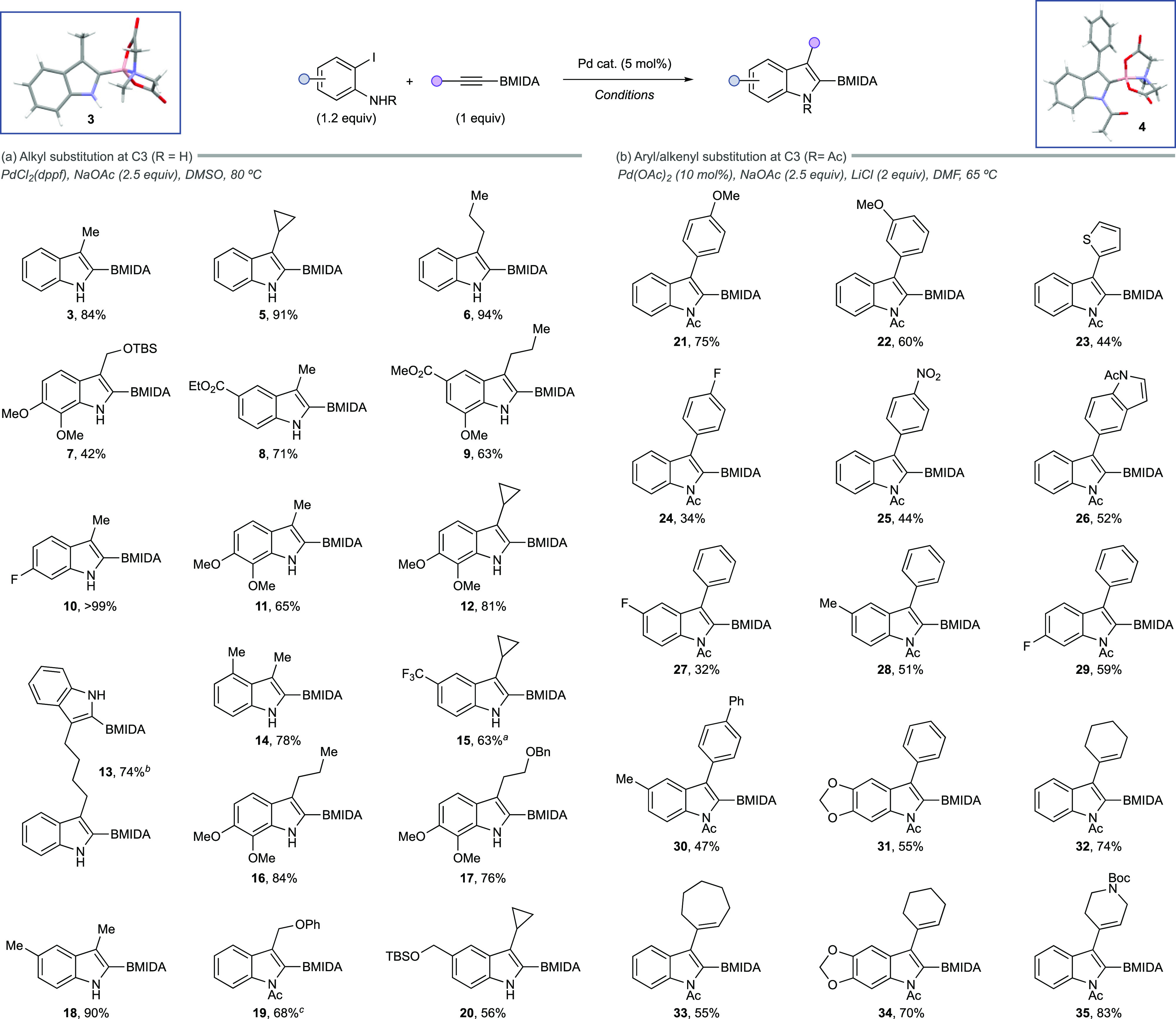
Example Scope of the Annulation Process Determined by ^1^H NMR assay using 1,4-dinitrobenzene as an internal standard. Using 10 mol % Pd(dppf)Cl_2_. Using Pd(OAc)_2_ (5 mol %), NaOAc (2.5 equiv), LiCl (2 equiv), DMF, 65 °C.

A series of alkyl alkynes were successfully accommodated to generate a small library of 2-BMIDA-3-alkyl indole products in good to excellent yield (Scheme 2a). The alkyl-substituted BMIDA alkyne progenitors were accessed via a metalation/borylation sequence using the requisite alkyl alkyne (see SI).^23^ Compound **19** was delivered in low yields under the PdCl_2_(dppf) general conditions; however, this was found to improve when using the ligand-free conditions developed for the aryl/alkenyl alkynes. The origin of this subtle substrate divergence remains unclear.

Aryl- and alkenyl-substituted alkynes were also broadly compatible with the annulation, giving a similar series of products; however, a general lower efficiency was noted for aryl-substituted alkynes, consistent with previous observations with bulky alkynes in this area.^7−9,18−20^

The aryl-substituted BMIDA alkyne components can also be accessed via metalation/borylation or via a simpler Sonogashira coupling of the commercially available acetylene BMIDA (see SI).^24,25^

The reaction is completely regioselective. Regioselectivity was unequivocally established by X-ray crystallography (**3** and **4**, Scheme 2) and NMR, showing that the BMIDA occupies the 2-position consistent with a larger steric footprint of this unit in comparison to the alkyl/aryl groups.^26^

A demonstration of the utility of the 2-BMIDA indole products is shown in Scheme 3. The sulfa drugs are a particularly important class of antibiotics.^27^ The developed methodology enables the rapid, regioselective synthesis of the sulfa drug chemotype, included marketed compound **37**(28) via annulation and subsequent Suzuki–Miyaura cross-coupling (Scheme 3a). Importantly, while a Larock approach to **38** could be envisaged via direct heteroannulation using the appropriate diaryl alkyne, steric issues lead to low yields for diaryl alkynes, and in addition, the subtle electronic differences lead to regioisomeric mixtures in these diaryl systems.^7−9,18−22^

**Scheme 3:**
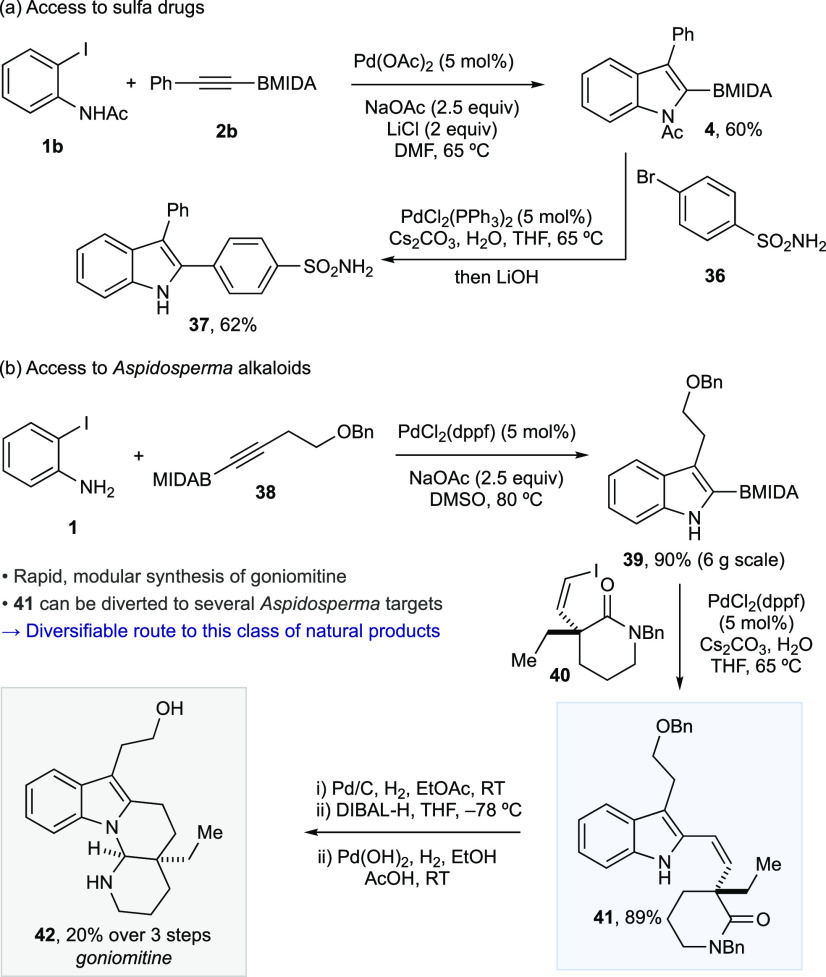
Utility of the Process in Drug and Natural Product Synthesis

Finally, this annulation/cross-coupling approach can be deployed to enable the modular synthesis of the *Aspidosperma* alkaloid goniomitine (Scheme 3b).^29^ Annulation using alkyne **38** delivers indole **39** on a multigram scale. Cross-coupling with lactam fragment **40** provided **41**, establishing the full carbon framework needed for the natural product. Hydrogenation, cyclization, and deprotection rapidly provided goniomitine (**42**). Importantly, intermediate **41** can be potentially diverted to other members of the *Aspidosperma* family following established approaches.^30^

In summary, a Larock-type annulation has been developed for the synthesis of 2-BMIDA indoles, allowing access to readily modifiable borylated heterocyclic scaffolds. The process accommodates a range of functionalized alkyne and aryl iodide coupling partners and delivers the products in good to excellent yield. The utility of the products has been highlighted in the rapid synthesis of drug and natural product scaffolds.^31^

## Supporting Information Available

Characterization and crystal structure data (PDF)
NMR spectra (PDF)

## Accession Codes

CCDC 2133112 and 2149746 contain the supplementary crystallographic data for this paper. These data can be obtained free of charge via www.ccdc.cam.ac.uk/data_request/cif, or by emailing data_request@ccdc.cam.ac.uk, or by contacting The Cambridge Crystallographic Data Centre, 12 Union Road, Cambridge CB2 1EZ, UK; fax: +44 1223 336033.
