# Supplementary material for: Synthesis of 2-BMIDA Indoles via Heteroannulation: Applications in Drug Scaffold and Natural Product Synthesis
Source: Org Lett. 2022 Apr 15;24(16):3024–7. doi: 10.1021/acs.orglett.2c00959 (PMC9062883; doi:10.1021/acs.orglett.2c00959)
Supplement: Supplementary file 1 — ol2c00959_si_001.pdf [file ol2c00959_si_001.pdf]

Supporting Information

Synthesis of 2-BMIDA indoles via heteroannulation: Applications in drug scaffold and natural product synthesis

George E. Bell,[a] James W. B. Fyfe,[a] Eva M. Israel,[a] Alexandra M. Z. Slawin,[a] Matthew Campbell,[b] and Allan J. B. Watson*[a]

[a] EaStCHEM, School of Chemistry, University of St Andrews, North Haugh, St Andrews, Fife, KY16 9ST, U.K.
[b] GlaxoSmithKline, Medicines Research Centre, Gunnels Wood Road, Stevenage, SG1 2NY, U.K.

*Email: aw260@st-andrews.ac.uk

Contents

1. General Experimental Details S2
2. General Experimental Procedures S2
3. Reaction Optimisation Data S4
4. Characterization Data S10
5. X-Ray Crystallography S36
6. BMIDA Volume Calculation S40
7. References S40

1. General Experimental Details

1.1 Purification of Solvents & Reagents

Dry DMSO was obtained by standing DMSO over activated alumina overnight before filtration then distilling over CaH_2 under vacuum and storing over activated 4 Å molecular sieves under a blanket of N_2. Dry DMF was obtained by stirring over 4 Å molecular sieves overnight before distilling over fresh 4 Å molecular sieves under vacuum and storing over activated 4 Å molecular sieves under a blanket of N_2. Dry THF was obtained from a PureSolv SPS-400-5 solvent purification system. DCM, MeCN, Et_2O, EtOAc, and hexane for purification purposes were used as obtained from suppliers without further purification. NaOAc was heated to melting under vacuum, then allowed to cool to room temperature, backfilled with N_2, and stored in a capped vial under N_2. LiCl was placed in a vacuum oven kept at 60 °C for at least 24 hours prior to use. $B(OMe)_3$ was distilled over CaH_2 and stored over activated 4 Å molecular sieves under N_2. All other reagents and solvents were obtained from commercial suppliers and were used without further purification unless otherwise stated. Purification was carried out according to standard laboratory methods.[1]

1.2 Experimental Details

Reactions were carried out using conventional glassware (preparation of intermediates) or in capped 5 or 20 mL microwave vials. Microwave vials were oven-dried (150 °C) and cooled to room temperature under vacuum and backfilled with N_2 prior to use. Reaction mixtures were prepared in a microwave vial before being capped with a septum and purged using N_2/vacuum (three cycles). Reactions were carried out at elevated temperatures in a sand bath atop a temperature-regulated hotplate/stirrer. Cooling to 0 °C was achieved using an ice/water bath. Cooling to –78 °C was achieved using a dry ice/acetone bath.

1.3 Purification of Products

Thin layer chromatography was carried out using Merck silica plates coated with fluorescent indicator UV254. These were analysed under 254 nm UV light and/or developed using potassium permanganate or vanillin solution. Normal phase flash chromatography was carried out using ZEOprep 60 HYD 40-63 µm silica gel.

1.4 Analysis of Products

Fourier Transformed Infra-Red (FTIR) spectra were obtained on a Shimadzu IRAffinity-1 machine. ^{1}H and ^{13}C NMR spectra were obtained on either a Bruker AV 400 at 400 MHz and 101 MHz, respectively, or Bruker DRX 500 at 500 MHz and 126 MHz, respectively. ^{19}F NMR spectra were obtained on a Bruker AV 400 spectrometer at 376 MHz. ^{11}B NMR spectra were obtained on a Bruker AV 300 spectrometer at 96 MHz. Chemical shifts are reported in ppm and coupling constants are reported in Hz with $CDCl_3$ referenced at 7.26 (^{1}H) and 77.16 ppm (^{13}C), DMSO-d_6 referenced at 2.50 (^{1}H) and 39.5 (^{13}C), acetone-d_6 referenced at 2.05 (^{1}H) and 28.9 and 206.3 ppm (^{13}C), and MeCN-d_3 referenced at 1.94 (^{1}H) and 1.3 and 118.3 ppm (^{13}C). ^{11}B NMR spectra are referenced to $BF_3{\cdot}Et_2O$. s = singlet, d = doublet, t = triplet, q = quartet, app = apparent, br = broad. High-resolution mass spectra were obtained through analysis at the University of St Andrews with a Thermo Exactive Orbitrap mass spectrometer. NMR conversions for optimisation studies were carried out by adding a known standard (0.05 M 1,4-dinitrobenzene in DMSO-d_6) to the crude reaction mixture. Note: (i) BMIDA products were typically poorly soluble in most NMR solvents; (ii) Restricted rotation was observed for BMIDA products, within ^{13}C NMR in particular.

2. General Experimental Procedures

General Procedure A:

For example, synthesis of **Compound 3**

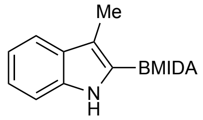

An oven-dried 5 mL microwave vial was charged with Pd(dppf)Cl_2 (7.3 mg, 10 μmol, 5 mol%), propyne boronic acid MIDA ester (39.0 mg, 0.2 mmol, 1.0 equiv.), 2-iodoaniline (52.6 mg, 0.24 mmol, 1.2 equiv.), and NaOAc (41.0 mg, 0.5 mmol, 2.5 equiv.). The vial was capped and purged with N_2 before DMSO (2 mL, 0.1 M) was added via syringe. The mixture was then stirred at 80 °C for 18 hours. The vial was allowed to cool to room temperature, decapped, and diluted with EtOAc (10 mL). The mixture was then washed with 10% aqueous LiCl solution (2 x 5 mL). The organic extract was dried over Na_2SO_4, filtered, and concentrated to give a residue that was purified by flash column chromatography (silica gel, 5–20% MeCN in DCM) to give the product as an off-white solid (48 mg, 84%).

General procedure B:
For example, synthesis of **Compound S1**

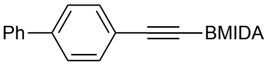

An oven-dried 20 mL microwave vial was charged with CuI (19.0 mg, 0.1 mmol, 10 mol%), Pd(dppf)Cl_2 (36.6 mg, 50 μmol, 5 mol%), 4-iodobiphenyl (336 mg, 1.20 mmol, 1.20 equiv.), and ethynyl boronic acid MIDA ester (181 mg, 1.0 mmol, 1.0 equiv.). The vial was capped and purged with N_2 before Et_3N (418 μL, 3.0 mmol, 3.0 equiv.) and DMF (5.0 mL, 0.2 M) were added via syringe. The mixture was stirred at room temperature for 18 hours before being decapped and diluted with EtOAc (50 mL). The mixture was then washed with 10% aqueous LiCl solution (2 x 25 mL). The organic extract was dried over Na_2SO_4, filtered, and concentrated to give a residue that was purified by flash column chromatography (silica gel, 5–20% MeCN in DCM) to give the product as a brown solid (241 mg, 72%).

General procedure C:
For example, synthesis of **Compound 38**

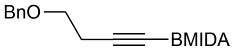

To a flame-dried 250 mL round bottom flask, ((but-3-yn-1-yloxy)methyl)benzene (6.63 g, 41.4 mmol, 1.0 equiv.) was dissolved in THF (124 mL, 0.33 M) and cooled to 0 °C. To this solution was added EtMgBr (22.6 mL, 49.7 mmol, 2.2 M in Et_2O, 1.2 equiv.) dropwise and the resulting suspension was stirred at 0 °C for 10 minutes before the ice bath was removed and stirring was continued for a further 30 minutes.

In a separate, flame-dried 500 mL round bottom flask, $B(OMe)_3$ (9.23 mL, 82.8 mmol, 2.0 equiv.) was dissolved in THF (100 mL, 0.82 M) and cooled to –78 °C. To this cold solution was added, *via* cannula, the above Grignard suspension over approximately 10 minutes. After complete addition, the reaction mixture was stirred at –78 °C for one hour before removing the dry ice/acetone bath and stirring for a further two hours as it warmed to room temperature. The flask was unstoppered and *N*-methyliminodiacetic acid (12.2 g, 82.8 mmol, 2.0 equiv.) was added followed by DMSO (50 mL). The flask was placed on a rotary evaporator with the water bath set at 60 °C to remove the majority of the volatiles. The flask contents were then distilled under high vacuum (<0.5 mbar, 80 °C) to remove remaining DMSO to leave a gum-like residue. To this was added 1:1 brine/H_2O (300 mL) and this was extracted with 3:2 EtOAc/acetone (2 x 300 mL). The organic layers were combined, washed with water (50 mL), dried over Na_2SO_4, filtered, and concentrated to a residue that was purified by flash column chromatography (silica gel, 0–25% MeCN in DCM) to give an off-white solid which was then triturated from EtOAc and hexane to give the product as a fluffy white solid (7.80 g, 60%).

General procedure D:
For example, synthesis of **Compound 31**

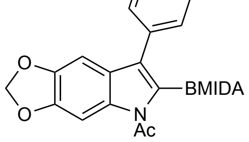

An oven-dried 5 mL microwave vial was charged with $Pd(OAc)_2$ (4.5 mg, 20 µmol, 10 mol%), (phenylethynyl)boronic acid MIDA ester (51.4 mg, 0.2 mmol, 1.0 equiv.), *N*-(6-iodobenzo[*d*][1,3]dioxol-5-yl)acetamide (73.2 mg, 0.24 mmol, 1.2 equiv.), LiCl (17.0 mg, 0.4 mmol, 2.0 equiv.), and NaOAc (41.0 mg, 0.5 mmol, 2.5 equiv.). The vial was capped and purged with N_2 before DMF (2 mL, 0.1 M) was added *via* syringe. The mixture was then stirred at 65 °C for 48 hours. The vial was allowed to cool to room temperature, decapped, and diluted with EtOAc (10 mL). The mixture was then washed with 10% aqueous LiCl solution (2 x 5 mL). The organic extract was dried over Na_2SO_4, filtered, and concentrated to give a residue that was purified by flash column chromatography (silica gel, 8–30% MeCN in DCM) to give the product as a light brown solid (48 mg, 55%).

General procedure E:

For example, synthesis of **Compound S2**

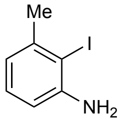

A 100 mL round bottom flask was charged with 2-iodo-3-nitrotoluene (2.63 g, 10.0 mmol, 1.0 equiv.), iron powder (4.05 g, 62.0 mmol, 6.20 equiv.), and EtOH (30 mL). The mixture was stirred vigorously while conc. HCl (37%, 0.99 mL, 12.0 mmol, 1.2 equiv.) was added dropwise. The mixture was then heated to reflux for 3 h. The mixture was allowed to cool to room temperature then diluted with EtOAc (50 mL). The mixture was filtered through a pad of celite, washing the cake with H_2O (50 mL). The biphasic mixture was separated, the organic extract was dried over Na_2SO_4, filtered, and concentrated to give a residue that was purified by flash column chromatography (silica gel, 5–10% Et_2O in petrol) to give the product as a light pink solid (2.12 g, 91%).

3. Reaction Optimisation Data

3.1 Alkyl Alkynes

3.1.1 Solvent

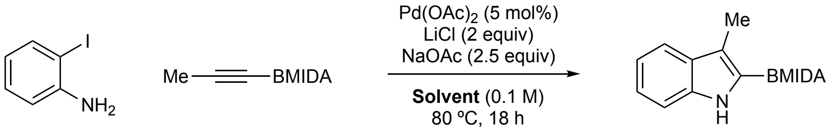

Reactions were carried out according to General Procedure A using $Pd(OAc)_2$ (2.2 mg, 10 µmol, 5 mol%), propyne BMIDA (39.0 mg, 0.2 mmol, 1.0 equiv.), 2-iodoaniline (52.6 mg, 0.24 mmol, 1.2 equiv.), LiCl (17.0 mg, 0.4 mmol, 2.0 equiv.), and NaOAc (41.0 mg, 0.5 mmol, 2.5 equiv.). The vial was capped and purged with N_2 prior to addition of **solvent** (2 mL, 0.1 M) via syringe**.** The reaction was stirred at 80 °C for 18 h, then allowed to cool to room temperature and decapped. The reaction mixture was diluted with EtOAc (10 mL) and washed with 10% aqueous LiCl solution (2 x 5 mL). The organic extract was dried over Na_2SO_4, filtered, and concentrated. 1,4-Dinitrobenzene in DMSO-d_6 (0.05 M, 1 mL) was added to the crude product and an aliquot was analysed by ^{1}H NMR.

Table S1

Entry	Solvent	Yield (%)
1	DMF	69
2	THF	0
3	MeCN	<5
4	Dioxane	0
5	Toluene	0
6	Sulpholane	0
7	DMSO	76

8	NMP	69

3.1.2 Temperature

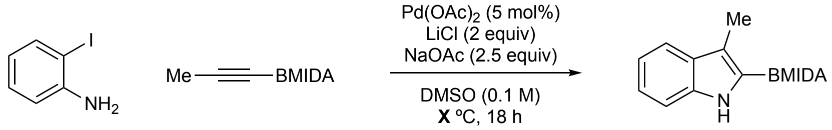

1a **2a** **3**

Reactions were carried out according to General Procedure A using $Pd(OAc)_2$ (2.2 mg, 10 µmol, 5 mol%), propyne BMIDA (39.0 mg, 0.2 mmol, 1.0 equiv.), 2-iodoaniline (52.6 mg, 0.24 mmol, 1.2 equiv.), LiCl (17.0 mg, 0.4 mmol, 2.0 equiv.), and NaOAc (41.0 mg, 0.5 mmol, 2.5 equiv.). The vial was capped and purged with N_2 prior to addition of DMSO (2 mL, 0.1 M) via syringe**.** The reaction was stirred at **X** °C for 18 h, then allowed to cool to room temperature and decapped. The reaction mixture was diluted with EtOAc (10 mL) and washed with 10% aqueous LiCl solution (2 x 5 mL). The organic extract was dried over Na_2SO_4, filtered, and concentrated. 1,4-Dinitrobenzene in DMSO-d_6 (0.05 M, 1 mL) was added to the crude product and an aliquot was analysed by ^{1}H NMR.

Table S2

Entry	Temp. (ºC)	Yield (%)
1	RT	trace
2	40	5
3	60	42
4	100	50

3.1.3 Time

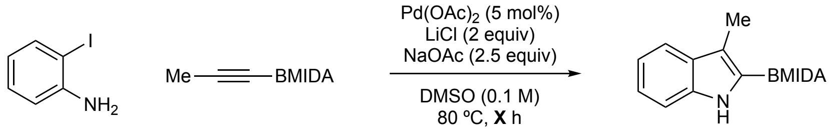

1a **2a** **3**

Reactions were carried out according to General Procedure A using $Pd(OAc)_2$ (2.2 mg, 10 µmol, 5 mol%), propyne BMIDA (39.0 mg, 0.2 mmol, 1.0 equiv.), 2-iodoaniline (52.6 mg, 0.24 mmol, 1.2 equiv.), LiCl (17.0 mg, 0.4 mmol, 2.0 equiv.), and NaOAc (41.0 mg, 0.5 mmol, 2.5 equiv.). The vial was capped and purged with N_2 prior to addition of DMSO (2 mL, 0.1 M) *via* syringe**.** The reaction was stirred at 80 °C for **X** h, then allowed to cool to room temperature and decapped. The reaction mixture was diluted with EtOAc (10 mL) and washed with 10% aqueous LiCl solution (2 x 5 mL). The organic extract was dried over Na_2SO_4, filtered, and concentrated. 1,4-Dinitrobenzene in DMSO-d_6 (0.05 M, 1 mL) was added to the crude product and an aliquot was analysed by ^{1}H NMR.

Table S3

Entry	Time (h)	Yield (%)
1	2	49
2	4	62
3	6	65
4	8	72
5	24	73

3.1.4 Stoichiometry

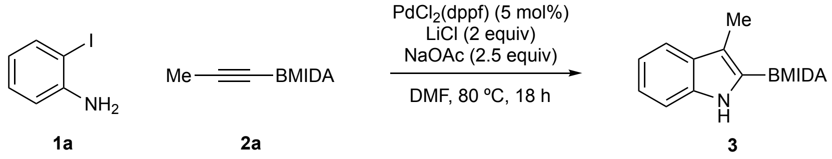

Reactions were carried out according to General Procedure A using $Pd(OAc)_2$ (2.2 mg, 10 µmol, 5 mol%), propyne BMIDA (**X** equiv.), 2-iodoaniline (**Y** equiv.), LiCl (17.0 mg, 0.4 mmol, 2.0 equiv.), and NaOAc (41.0 mg, 0.5 mmol, 2.5 equiv.). The vial was capped and purged with N_2 prior to addition of DMSO (2 mL, 0.1 M) *via* syringe**.** The reaction was stirred at 80 °C for 18 h, then allowed to cool to room temperature and decapped. The reaction mixture was diluted with EtOAc (10 mL) and washed with 10% aqueous LiCl solution (2 x 5 mL). The organic extract was dried over Na_2SO_4, filtered, and concentrated. 1,4-Dinitrobenzene in DMSO-d_6 (0.05 M, 1 mL) was added to the crude product and an aliquot was analysed by ^{1}H NMR.

Table S4

Entry	X:Y (h)	Yield (%)
1	1.5:1	77
2	2.0:1	74
3	1:1.2	76
4	1:1.5	72
5	1:2.0	73

3.1.5 Concentration

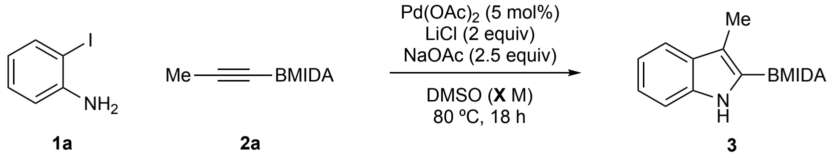

Reactions were carried out according to General Procedure A using $Pd(OAc)_2$ (2.2 mg, 10 µmol, 5 mol%), propyne BMIDA (39.0 mg, 0.2 mmol, 1.0 equiv.), 2-iodoaniline (52.6 mg, 0.24 mmol, 1.2 equiv.), LiCl (17.0 mg, 0.4 mmol, 2.0 equiv.), and NaOAc (41.0 mg, 0.5 mmol, 2.5 equiv.). The vial was capped and purged with N_2 prior to addition of DMSO (**X** M) *via* syringe**.** The reaction was stirred at 80 °C for 18 h, then allowed to cool to room temperature and decapped. The reaction mixture was diluted with EtOAc (10 mL) and washed with 10% aqueous LiCl solution (2 x 5 mL). The organic extract was dried over Na_2SO_4, filtered, and concentrated. 1,4-Dinitrobenzene in DMSO-d_6 (0.05 M, 1 mL) was added to the crude product and an aliquot was analysed by ^{1}H NMR.

Table S5

Entry	Concentration (M)	Yield (%)
1	0.05	66
2	0.1	72
3	0.2	67
4	0.5	55

3.1.6 Base

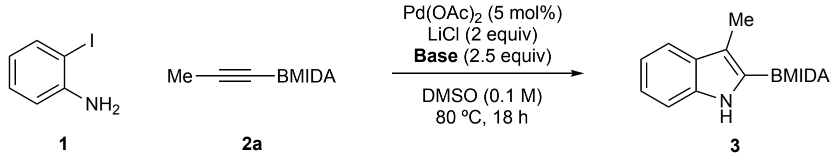

Reactions were carried out according to General Procedure A using $Pd(OAc)_2$ (2.2 mg, 10 μmol, 5 mol%), propyne BMIDA (39.0 mg, 0.2 mmol, 1.0 equiv.), 2-iodoaniline (52.6 mg, 0.24 mmol, 1.2 equiv.), LiCl (17.0 mg, 0.4 mmol, 2.0 equiv.), and **base** (0.5 mmol, 2.5 equiv.). The vial was capped and purged with N_2 prior to addition of DMSO (2 mL, 0.1 M) *via* syringe**.** The reaction was stirred at 80 °C for 18 h, then allowed to cool to room temperature and decapped. The reaction mixture was diluted with EtOAc (10 mL) and washed with 10% aqueous LiCl solution (2 x 5 mL). The organic extract was dried over Na_2SO_4, filtered, and concentrated. 1,4-Dinitrobenzene in DMSO-d_6 (0.05 M, 1 mL) was added to the crude product and an aliquot was analysed by ^{1}H NMR.

Table S6

Entry	Base	Yield (%)
1	KOAc (49.1 mg)	78
2	LiOAc (33.0 mg)	77
3	K_3PO_4 (106.2 mg)	17
4	K_2CO_3 (69.1 mg)	<5
5	Et_3N (70.0 μL)	71

3.1.7 Salt

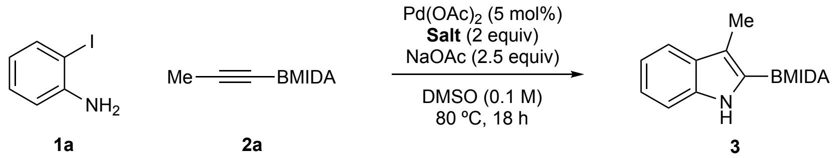

Reactions were carried out according to General Procedure A using $Pd(OAc)_2$ (2.2 mg, 10 μmol, 5 mol%), propyne BMIDA (39.0 mg, 0.2 mmol, 1.0 equiv.), 2-iodoaniline (52.6 mg, 0.24 mmol, 1.2 equiv.), **salt** (0.4 mmol, 2.0 equiv.), and NaOAc (41.0 mg, 0.5 mmol, 2.5 equiv.). The vial was capped and purged with N_2 prior to addition of DMSO (2 mL, 0.1 M) *via* syringe**.** The reaction was stirred at 80 °C for 18 h, then allowed to cool to room temperature and decapped. The reaction mixture was diluted with EtOAc (10 mL) and washed with 10% aq. LiCl solution (2 x 5 mL). The organic extract was dried over Na_2SO_4, filtered, and concentrated. 1,4-Dinitrobenzene in DMSO-d_6 (0.05 M, 1 mL) was added to the crude product and an aliquot was analysed by ^{1}H NMR.

Table S7

Entry	Salt	Yield (%)
1	LiF (10.4 mg)	77
2	NaCl (23.4 mg)	77
3	KCl (29.8 mg)	82
4	TBACl hydrate (111.2 mg)	56

3.1.8 Salt/base Stoichiometry

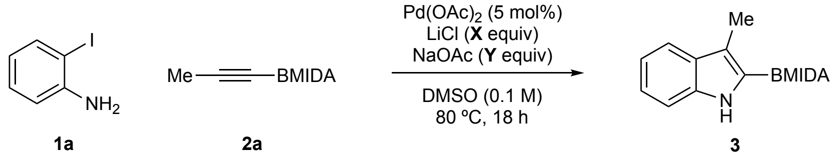

Reactions were carried out according to General Procedure A using $Pd(OAc)_2$ (2.2 mg, 10 μmol, 5 mol%), propyne BMIDA (39.0 mg, 0.2 mmol, 1.0 equiv.), 2-iodoaniline (52.6 mg, 0.24 mmol, 1.2 equiv.), LiCl (**X** equiv.), and NaOAc (**Y** equiv.). The vial was capped and purged with N_2 prior to addition of DMSO (2 mL, 0.1 M) *via* syringe**.** The reaction was stirred at 80 °C for 18 h, then allowed to cool to room temperature and decapped. The reaction mixture was diluted with EtOAc (10 mL) and washed with 10% aq. LiCl solution (2 x

5 mL). The organic extract was dried over Na_2SO_4, filtered, and concentrated. 1,4-Dinitrobenzene in DMSO-d_6 (0.05 M, 1 mL) was added to the crude product and an aliquot was analysed by ^{1}H NMR.

Table S8

Entry	LiCl:NaOAc (equiv.)	Yield (%)
1	2:3 (17.0: 49.2 mg)	82
2	2:1 (17.0: 16.4 mg)	79
3	2:0.5 (17.0: 8.2 mg)	45
4	2:0 (17.0 mg)	7
5	3:2.5 (25.4 mg: 41 mg)	78
6	1:2.5 (8.5 mg: 41 mg)	83
7	0.5:2.5 (4.2: 41 mg)	83
8	0:2.5 (41 mg)	66

3.1.9 Pd Catalyst

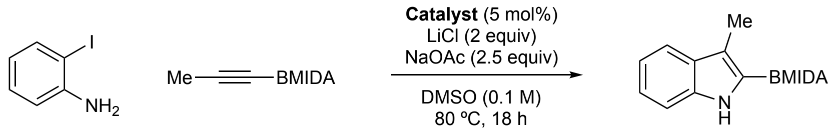

1a **2a** **3**

Reactions were carried out according to General Procedure A using **Pd catalyst** (10 µmol, 5 mol%), propyne BMIDA (39.0 mg, 0.2 mmol, 1.0 equiv.), 2-iodoaniline (52.6 mg, 0.24 mmol, 1.2 equiv.), LiCl (17.0 mg, 0.4 mmol, 2.0 equiv.), and NaOAc (41.0 mg, 0.5 mmol, 2.5 equiv.). The vial was capped and purged with N_2 prior to addition of DMSO (2 mL, 0.1 M) *via* syringe**.** The reaction was stirred at 80 °C for 18 h, then allowed to cool to room temperature and decapped. The reaction mixture was diluted with EtOAc (10 mL) and washed with 10% aq. LiCl solution (2 x 5 mL). The organic extract was dried over Na_2SO_4, filtered, and concentrated. 1,4-Dinitrobenzene in DMSO-d_6 (0.05 M, 1 mL) was added to the crude product and an aliquot was analysed by ^{1}H NMR.

Table S9

Entry	Pd catalyst	Yield (%)
1	$PdCl_2$ (1.8 mg)	85
2	Pd(dppf)Cl_2 (7.3 mg)	87
3	Pd(PPh_3)$_4$ (11.6 mg)	10
4	[Pd(allyl)Cl]$_2$ (1.8 mg)	84
5	Pd(MeCN)$_2$Cl$_2$ (2.6 mg)	85
6	Pd(PPh_3)$_2$Cl$_2$ (7.0 mg)	62

3.2 Aryl Alkynes

3.2.1 NaOAc/Temperature

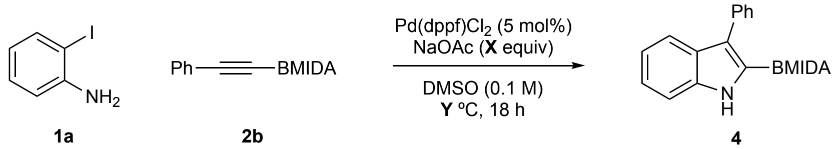

1a **2b** **4**

Reactions were carried out according to General Procedure D using Pd(dppf)Cl_2 (1.8 mg, 10 µmol, 5 mol%), (phenylethynyl)boronic acid MIDA ester (51.4 mg, 0.2 mmol, 1.0 equiv.), 2-iodoaniline (52.6 mg, 0.24 mmol, 1.2 equiv.), and NaOAc (**X** equiv.). The vial was capped and purged with N_2 before DMSO (2 mL, 0.1 M) was added *via* syringe. The mixture was then stirred at **Y** °C for 18 hours. The vial was allowed to cool to room temperature, and decapped. The mixture was diluted with EtOAc (10 mL) and washed with 10% aq. LiCl

solution (2 x 5 mL). The organic extract was dried over Na_2SO_4, filtered, and concentrated. 1,4-Dinitrobenzene in DMSO-d_6 (0.05 M, 1 mL) was added to the crude product and an aliquot was analysed by ^{1}H NMR.

Table S10

Entry	NaOAc (equiv.)	Temp. (°C)	Yield (%)	Remaining 2b (%)
1	1.2 (20 mg)	80	14	65
2	2.5 (41 mg)	80	16	58
3	1.2 (20 mg)	100	37	25
4	2.5 (41 mg)	100	35	24
5	1.2 (20 mg)	120	41	5
6	2.5 (41 mg)	120	30	7
7	1.2 (20 mg)	140	37	8
8	2.5 (41 mg)	140	14	2

3.2.2 Temperature/Time

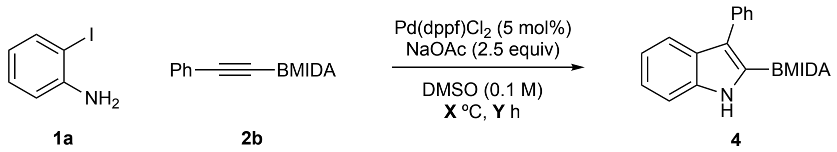

Reactions were carried out according to General Procedure D using Pd(dppf)Cl_2 (1.8 mg, 10 µmol, 5 mol%), (phenylethynyl)boronic acid MIDA ester (51.4 mg, 0.2 mmol, 1.0 equiv.), 2-iodoaniline (52.6 mg, 0.24 mmol, 1.2 equiv.), and NaOAc (41.0 mg, 0.5 mmol, 2.5 equiv.). The vial was capped and purged with N_2 before DMSO (2 mL, 0.1 M) was added *via* syringe. The mixture was then stirred at **X** °C for **Y** hours. The vial was allowed to cool to room temperature, and decapped. The mixture was diluted with EtOAc (10 mL) and washed with 10% aq. LiCl solution (2 x 5 mL). The organic extract was dried over Na_2SO_4, filtered, and concentrated. 1,4-Dinitrobenzene in DMSO-d_6 (0.05 M, 1 mL) was added to the crude product and an aliquot was analysed by ^{1}H NMR.

Table S11

Entry	Temp. (°C)	Time (h)	Yield (%)	Remaining 2b (%)
1	80	48	23	45
2	80	72	25	38
3	100	48	39	12
4	100	72	25	12

3.2.3 Pd Catalyst

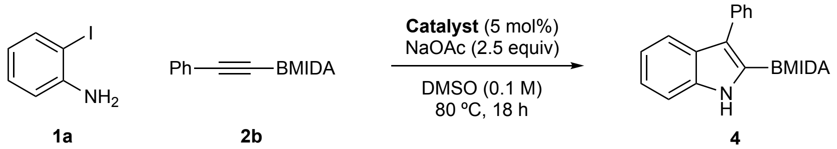

Reactions were carried out according to General Procedure D using **Pd catalyst** (10 µmol, 5 mol%), (phenylethynyl)boronic acid MIDA ester (51.4 mg, 0.2 mmol, 1.0 equiv.), 2-iodoaniline (52.6 mg, 0.24 mmol, 1.2 equiv.), and NaOAc (41.0 mg, 0.5 mmol, 2.5 equiv.). The vial was capped and purged with N_2 before DMSO (2 mL, 0.1 M) was added *via* syringe. The mixture was then stirred at 80 °C for 18 hours. The vial was allowed to cool to room temperature and decapped. The mixture was diluted with EtOAc (10 mL) and washed with 10% aq. LiCl solution (2 x 5 mL). The organic extract was dried over Na_2SO_4, filtered, and concentrated.

1,4-Dinitrobenzene in DMSO-d_6 (0.05 M, 1 mL) was added to the crude product and an aliquot was analysed by ^{1}H NMR.

Table S12

Entry	Catalyst	Yield (%)	Remaining 2b (%)
1	$PdCl_2$ (1.8 mg)	34	30
2	$[Pd(allyl)Cl]_2$ (1.8 mg)	47	32
3	$Pd(MeCN)_2Cl_2$ (2.6 mg)	29	43

3.2.4 Aniline/Anilide with LiCl

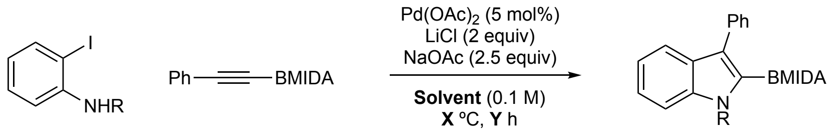

2b **4**

Reactions were carried out according to General Procedure D using $Pd(OAc)_2$ (4.5 mg, 20 µmol, 10 mol%), (phenylethynyl)boronic acid MIDA ester (51.4 mg, 0.2 mmol, 1.0 equiv.), **aniline/anilide** (0.24 mmol, 1.2 equiv.), LiCl (17.0 mg, 0.4 mmol, 2.0 equiv.), and NaOAc (41.0 mg, 0.5 mmol, 2.5 equiv.). The vial was capped and purged with N_2 before **solvent** (2 mL, 0.1 M) was added *via* syringe. The mixture was then stirred at **X** °C for **Y** hours. The vial was allowed to cool to room temperature and decapped. The mixture was diluted with EtOAc (10 mL) and washed with 10% aq. LiCl solution (2 x 5 mL). The organic extract was dried over Na_2SO_4, filtered, and concentrated. 1,4-Dinitrobenzene in DMSO-d_6 (0.05 M, 1 mL) was added to the crude product and an aliquot was analysed by ^{1}H NMR.

Table S13

Entry	R	Solvent	Temp. (°C)	Time (h)	Yield (%)	Remaining 2b (%)
1	H (**1a**)	DMF	65	24	38	44
2	Ac (**1b**)	DMF	65	24	55	20
3	Ac (**1b**)	DMF	65	48	60[a]	20
4	Ts (**S27**)	DMF	65	48	n.d.	72
5	Ac (**1b**)	DMSO	80	18	11	52

[a]Isolated yield.

4. Characterization Data

4.1 Synthesis of Starting Materials and Intermediates

Compound S1

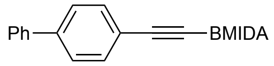

Prepared according to General Procedure B using $Pd(dppf)Cl_2$ (36.6 mg, 50 µmol, 5 mol%) 4-iodobiphenyl (336 mg, 1.20 mmol, 1.20 equiv.) CuI (19.0 mg, 100 µmol, 10 mol%), acetylene boronic acid MIDA ester (181 mg, 1.0 mmol, 1.0 equiv.), and Et_3N (418 uL, 3.0 mmol, 3.0 equiv.). Stirred at 50 °C for 17 h. Flash column chromatography (5–20% MeCN in DCM) gave the product as a brown solid (241 mg, 72%).

^{1}H NMR (500 MHz, Acetone-d_6): δ 7.72 – 7.64 (m, 4H), 7.62 – 7.53 (m, 2H), 7.51 – 7.44 (m, 2H), 7.43 – 7.35 (m, 1H), 4.35 (d, J = 17.0 Hz, 2H), 4.20 (d, J = 16.9 Hz, 2H), 3.35 (s, 3H).

^{13}C NMR (126 MHz, Acetone-d_6): δ 168.6, 142.0, 140.8, 133.1, 129.8, 128.7, 127.7, 127.7, 122.9, 62.4, 48.5. The carbon bearing boron was not observed.

^{11}B NMR (96 MHz, Acetone-d_6): δ 6.5.
υ_{max} (solid): 3024, 2191, 1765, 1483, 1288, 1254, 1020, 1005 cm^{-1}.
HRMS (ESI) *m/z*: $[M+Na]^+$ Calcd for $C_{19}H_{16}BNNaO_4$ 356.1070; Found 356.1067.

Compound S2

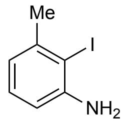

Prepared according to General procedure E using 2-iodo-3-nitrotoluene (2.63 g, 10.0 mmol, 1.0 equiv.), iron powder (4.05 g, 62.0 mmol, 6.20 equiv.), and conc. HCl (37%, 0.99 mL, 12.0 mmol, 1.2 equiv.). Flash column chromatography (silica gel, 5–10% Et_2O in petrol) gave the product as a light pink solid (2.12 g, 91%).
^1H NMR (500 MHz, $CDCl_3$) δ 7.03 – 7.00 (m, 1H), 6.65 (dd, *J* = 7.3, 1.6 Hz, 1H), 6.58 (dd, *J* = 7.9, 1.5 Hz, 1H), 4.09 (br s, 2H), 2.43 (s, 3H).
^{13}C NMR (126 MHz, $CDCl_3$) δ 147.2, 142.5, 128.6, 119.7, 112.0, 91.7, 29.4.
Spectral data in agreement with literature values.[2]

Compound S3

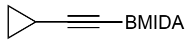

Prepared according to General Procedure C using cyclopropylacetylene (2.54 mL, 30 mmol, 1.0 equiv.), EtMgBr (3.0 M in Et_2O, 12 mL, 36.0 mmol 1.2 equiv.), $B(OMe)_3$ (6.69 mL, 60 mmol, 2.0 equiv.), and *N*-methyliminodiacetic acid (8.83 g, 60.0 mmol, 2.0 equiv.). Flash column chromatography (silica gel, 10–30% MeCN in DCM) gave the product as a white solid (3.83 g, 58%).
^1H NMR (500 MHz, DMSO-d_6): δ 4.20 (d, *J* = 17.1 Hz, 2H), 4.01 (d, *J* = 17.1 Hz, 2H), 2.92 (s, 3H), 1.36 – 1.29 (m, 1H), 0.82 – 0.73 (m, 2H), 0.63 – 0.61 (m, 2H).
^{13}C NMR (126 MHz, DMSO-d_6): δ 168.8, 104.6, 61.3, 47.7, 8.2, −0.2. The carbon bearing boron was not observed.
^{11}B NMR (160 MHz, DMSO-d_6): δ 5.8.
ν_{max} (solid): 3013, 2369, 2205, 1765, 1464, 1288, 1020 cm^{-1}.
HRMS (ESI) *m/z*: $[M+Na]^+$ Calcd for $C_{10}H_{12}BNaNO_4$ 244.0757; Found 244.0753.

Compound S4

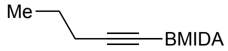

Prepared according to General Procedure C using 1-pentyne (0.99 mL, 10 mmol, 1.0 equiv.), EtMgBr (3.0 M in Et_2O, 4 mL, 12 mmol, 1.2 equiv.), $B(OMe)_3$ (2.23 mL, 20 mmol, 2.0 equiv.), and *N*-methyliminodiacetic acid (2.95 g, 20 mmol, 2.0 equiv.). Flash column chromatography (silica gel, 10–30% MeCN in DCM) gave the product as a white solid (1.10 g, 49%).
^1H NMR (500 MHz, $CDCl_3$): δ 4.09 – 4.00 (m, 2H), 3.79 (d, *J* = 16.8 Hz, 2H), 3.08 (s, 3H), 2.20 (t, *J* = 7.1 Hz, 2H), 1.57 – 1.50 (m, 2H), 0.97 (t, *J* = 7.4 Hz, 3H).
^{13}C NMR (126 MHz, $CDCl_3$): δ 168.1, 103.9, 61.6, 48.0, 22.0, 21.5, 13.7. The carbon bearing boron was not observed.
^{11}B NMR (160 MHz, $CDCl_3$): δ 6.3.
ν_{max} (solid): 3019, 2189, 1767, 1514, 1464, 1450, 1422 cm^{-1}.
HRMS (ESI) *m/z*: $[M+H]^+$ Calcd for $C_{10}H_{15}BNO_4$ 224.1094; Found 224.1088.

Compound S5

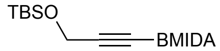

Prepared according to General Procedure C using *tert*-butyldimethyl(prop-2-yn-1-yloxy)silane (5.11 g, 30.0 mmol 1.0 equiv.), EtMgBr (3.08 M in Et_2O, 11.7 mL, 36.0 mmol, 1.2 equiv.), $B(OMe)_3$ (6.69 mL, 60.0 mmol, 2.0 equiv.), and *N*-methyliminodiacetic acid (8.83 g, 60.0 mmol, 2.0 equiv.). Flash column chromatography (silica gel 10–30% MeCN in DCM) gave the product as a white solid (2.47 g, 25%).
^1H NMR (500 MHz, DMSO-d_6): δ 4.32 (s, 2H), 4.26 (d, J = 17.2 Hz, 2H), 4.05 (d, J = 17.2 Hz, 2H), 2.97 (s, 3H), 0.86 (s, 9H), 0.09 (s, 6H).
^{13}C NMR (126 MHz, DMSO-d_6): δ 168.7, 99.2, 61.4, 51.6, 47.8, 25.7, 17.9, −5.1. The carbon bearing boron was not observed.
^{11}B NMR (96 MHz, DMSO-d_6): δ 5.83.
υ_{max} (solid): 2930, 1763, 1462, 1287 cm^{-1}.
HRMS (ESI) *m/z*: $[M+Na]^+$ Calcd for $C_{14}H_{24}BNNaO_5Si$ 348.1414; Found 348.1405.

Compound S6

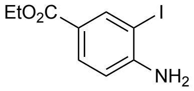

A mixture of benzocaine (826 mg, 5.00 mmol, 1.00 equiv.), potassium periodate (1.15 g, 5.00 mmol, 1.00 equiv.), NaCl (584 mg, 10.0 mmol, 2.00 equiv.), and KI (830 mg, 5.00 mmol, 1.00 equiv.) was stirred vigorously in AcOH/H_2O (9:1, 10 mL) at room temperature for 24 h. EtOAc (25 mL) was then added, and the layers were separated. The organic extract was washed successively with brine (10 mL), sat. aq. $Na_2S_2O_3$ (10 mL), and sat. aq. $NaHCO_3$ (10 mL). The organic layer was dried over Na_2SO_4, filtered, and concentrated to a residue that was purified by flash column chromatography (silica gel, 10–30% Et_2O in hexane) to afford the product as a white solid (1.26 g, 87%).
^1H NMR (500 MHz, $CDCl_3$): δ 8.33 (d, J = 1.9 Hz, 1H), 7.82 (dd, J = 8.4, 1.9 Hz, 1H), 6.70 (d, J = 8.4 Hz, 1H), 4.52 (s, 2H), 4.31 (q, J = 7.1 Hz, 2H), 1.36 (t, J = 7.1 Hz, 3H).
^{13}C NMR (126 MHz, $CDCl_3$): δ 165.4, 150.7, 141.1, 131.3, 121.7, 113.2, 82.3, 60.8, 14.5.
Spectral data in agreement with literature values.[3]

Compound S7

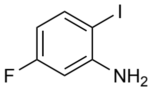

Prepared according to General procedure E using 2-iodo-5-fluoronitrobenzene (1.33 g, 5.0 mmol, 1.0 equiv.), iron powder (2.03 g, 31.0 mmol, 6.20 equiv.), and conc. HCl (37%, 493 µL, 12.0 mmol, 1.2 equiv.). Flash column chromatography (silica gel, 2–4% Et_2O in petrol) gave the product as a pale yellow solid (902 mg, 76%).
^1H NMR (500 MHz, $CDCl_3$): δ 7.56 – 7.53 (m, 1H), 6.48 – 6.45 (m, 1H), 6.28 – 6.24 (m, 1H), 4.19 (br s, 2H).
^{13}C NMR (126 MHz, $CDCl_3$): δ 164.1 (d, ^1J = 244.8 Hz), 148.2 (d, ^3J = 11.0 Hz), 139.8 (d, ^3J = 9.6 Hz), 107.3 (d, ^2J = 22.2 Hz), 101.7 (d, ^2J = 25.6 Hz), 77.1 (d, ^4J = 2.6 Hz).
^{19}F NMR (470 MHz, $CDCl_3$): δ –113.4.
Spectral data in agreement with literature values.[4]

Compound S8

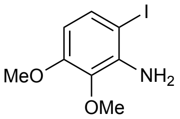

A mixture of 2,3-dimethoxyaniline (157 mg, 1.02 mmol, 1.00 equiv.) in Et_2O (6.8 mL, 0.15 M) and sat. aq. Na_2CO_3 (2 mL) was stirred vigorously in the dark at room temperature. A solution of ICl (271 mg, 1.67 mmol, 1.63 equiv.) in Et_2O (2 mL) was added in one portion and the mixture was stirred for 3 h at room temperature. The layers were separated, and the organic extract was washed successively with sat. aq. $Na_2S_2O_3$ (10 mL) and sat. aq. $NaHCO_3$ (10 mL). The organic extract was dried over Na_2SO_4, filtered, and concentrated to a residue

that was purified by flash column chromatography (silica gel, 2.5–5% acetone in hexane) to give the product as a pale yellow oil which solidified on standing (146 mg, 51%).
^{1}H NMR (500 MHz, $CDCl_3$): δ 7.30 (d, *J* = 8.8 Hz, 1H), 6.18 (d, *J* = 8.8 Hz, 1H), 4.25 (s, 2H), 3.83 (s, 3H), 3.82 (s, 3H).
^{13}C NMR (126 MHz, $CDCl_3$): δ 153.2, 141.7, 135.4, 133.2, 104.3, 74.0, 60.0, 56.0.
Spectral data in agreement with literature values.[5]

Compound S9

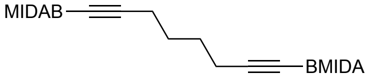

Prepared according to General Procedure C with 1,7-octadiyne (1.33 mL, 10.0 mmol, 1.0 equiv.), EtMgBr (2.85 M in Et_2O, 8.42 mL, 2.4 equiv.), $B(OMe)_3$ (4.46 mL, 40.0 mmol, 2.0 equiv.), and *N*-methyliminodiacetic acid (5.89 g, 40.0 mmol, 4.0 equiv.). Flash column chromatography (silica gel 10–60% MeCN in DCM) gave the product as a white solid (448 mg, 11%).
^{1}H NMR (500 MHz, DMSO-*d*$_6$): δ 4.23 (d, *J* = 17.2 Hz, 4H), 4.03 (d, *J* = 17.1 Hz, 4H), 2.95 (s, 6H), 2.27 – 2.21 (m, 4H), 1.56 – 1.54 (m, 4H).
^{13}C NMR (126 MHz, DMSO-*d*$_6$): δ ^{13}C NMR (126 MHz, DMSO) δ 168.7, 101.3, 61.3, 47.7, 27.2, 18.3. The carbon bearing boron is not observed due to quadrupolar relaxation.
^{11}B NMR (96 MHz, DMSO-*d*$_6$): δ 6.3.
υ_{max} (solid): 1749, 1454, 1339, 1290, 1167, 1140, 1022, 1005 cm^{-1}.
HRMS (ESI) *m/z*: $[M–H]^-$ Calcd for $C_{18}H_{21}B_2N_2O_8$ 415.1490; Found 415.1490.

Compound S10

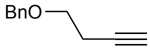

A slurry of NaH (60% dispersion in mineral oil, 3.0 g, 75 mmol, 1.5 equiv.) in THF (100 mL, 0.36 M) and DMF (40 mL, 0.36 M) was cooled to 0 °C and stirred. To this was added 3-butyn-1-ol (3.50 g, 50 mmol, 1.0 equiv.) dropwise *via* syringe. The reaction mixture was then stirred at 0 °C for 30 min before allowing to warm to room temperature where it was stirred for 30 min. The mixture was then cooled to 0 °C prior to addition of benzyl bromide (8.9 mL, 75 mmol, 1.5 equiv.). The reaction mass was then allowed to warm to room temperature and stirred for a further 4 h before being quenched with sat. aq. NH_4Cl (20 mL). The layers were separated, and the aqueous layer extracted with Et_2O (2 x 250 mL). The combined organics were then washed with water, dried over Na_2SO_4, filtered, and concentrated to give a residue that was purified by flash column chromatography (silica gel, 0–10% Et_2O in hexane) gave the product as a colourless oil (8.19 g, >99%).
^{1}H NMR (400 MHz, $CDCl_3$): δ 7.38 – 7.32 (m, 4H), 7.32 – 7.26 (m, 1H), 4.57 (s, 2H), 3.61 (t, *J* = 6.9 Hz, 2H), 2.51 (td, *J* = 6.9, 2.7 Hz, 2H), 2.00 (t, *J* = 2.7 Hz, 1H).
^{13}C NMR (126 MHz, $CDCl_3$): δ 138.1, 128.5, 127.8, 127.8, 81.4, 73.1, 69.5, 68.2, 20.0. Spectral data in agreement with literature values.[6]

Compound S11

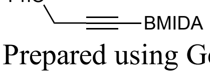

Prepared using General Procedure C using phenyl propargyl ether (1.59 g, 12 mmol, 1 equiv.), EtMgBr (3.05 M in Et_2O, 4.72 mL, 14.4 mmol, 1.2 equiv.), $B(OMe)_3$ (2.68 mL, 24 mmol, 2 equiv.), and *N*-methyliminodiacetic acid (3.53 g, 24 mmol, 2 equiv.). Flash column chromatography (silica gel, 0–10% MeCN in DCM) gave the product as an off-white solid (1.05 g, 30%).
^{1}H NMR (500 MHz, DMSO-*d*$_6$) δ 7.33 – 7.28 (m, 2H), 7.00 – 6.95 (m, 3H), 4.82 (s, 2H), 4.26 (d, *J* = 17.2 Hz, 2H), 4.05 (d, *J* = 17.1 Hz, 2H), 2.91 (s, 3H).
^{13}C NMR (126 MHz, DMSO-*d*$_6$): δ 168.6, 157.3, 129.5, 121.2, 114.9, 95.5, 61.5, 55.9, 47.8. The carbon bearing boron was not observed.

^{11}B NMR (96 MHz, DMSO-d_6): δ 6.1.
$υ_{max}$ (solid): 3215, 1768, 1492, 1134, 1028, 752, 688 cm^{-1}.
HRMS (ESI) *m/z*: $[M+Na]^+$ Calcd for $C_{14}H_{14}BNO_5Na$ 310.0863; Found 310.0851.

Compound S12

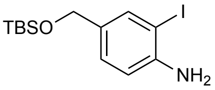

A solution of 4-amino-3-iodobenzyl alcohol (400 mg, 1.61 mmol, 1.0 equiv.) and imidazole (153 mg, 2.24 mmol, 1.40 equiv.) in DCM (4.9 mL, 0.33 M) was treated with *tert*-butyldimethylsilyl chloride (290 mg, 1.93 mmol, 1.20 equiv.). The reaction mixture was stirred at room temperature for 24 h then filtered through a plug of silica, eluting with EtOAc/hexane (1/1, 100 mL). The filtrate was concentrated to give the product as a brown oil (570 mg, 98%).
^{1}H NMR (500 MHz, $CDCl_3$): δ 7.60 (dd, *J* = 1.9, 0.9 Hz, 1H), 7.12 – 7.06 (m, 1H), 6.71 (d, *J* = 8.1 Hz, 1H), 4.58 (d, *J* = 0.9 Hz, 2H), 4.04 (br s, 2H), 0.93 (s, 9H), 0.09 (s, 6H).
^{13}C NMR (126 MHz, $CDCl_3$): δ 145.8, 137.1, 133.3, 127.8, 114.6, 84.1, 64.2, 26.1, 18.5, −5.0.
Spectral data in agreement with literature values.[7]

Compound S13

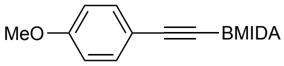

Prepared according to General Procedure B using 4-iodoanisole (155 mg, 663 µmol, 1.2 equiv.), Pd(dppf)Cl_2 (20.2 mg, 27.6 µmol, 5 mol%), CuI (10.5 mg, 55.3 µmol, 0.1 equiv.), acetylene boronic acid MIDA ester (100 mg, 553 µmol, 1 equiv.), and Et_3N (0.231 mL, 1.66 mmol, 3 equiv.). Flash column chromatography (5–30% MeCN in DCM) gave the product as a light brown solid (115 mg, 72%).
^{1}H NMR (500 MHz, DMSO-d_6): δ 7.42 (d, *J* = 8.7 Hz, 2H), 6.94 (d, *J* = 8.8 Hz, 2H), 4.30 (d, *J* = 17.2 Hz, 2H), 4.12 (d, *J* = 17.1 Hz, 2H), 3.77 (s, 3H), 3.05 (s, 3H).
^{13}C NMR (126 MHz, DMSO-d_6): δ 168.7, 159.6, 133.1, 114.4, 114.2, 99.6, 61.4, 55.2, 47.8.
^{11}B NMR (96 MHz, DMSO-d_6): δ 6.5.
$υ_{max}$ (solid): 2183, 1749, 1508, 1285, 1242, 1005 cm^{-1}.
HRMS (ESI) *m/z*: $[M+H]^+$ Calcd for $C_{14}H_{15}BNO_5$ 288.1043; Found 288.1039.

Compound S14

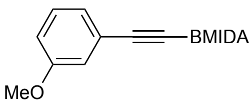

Prepared according to General Procedure B using 3-iodoanisole (155 mg, 663 µmol, 1.2 equiv.), Pd(dppf)Cl_2 (20.2 mg, 27.6 µmol, 5 mol%), CuI (10.5 mg, 55.3 µmol, 10 mol%), acetylene boronic acid MIDA ester (100 mg, 553 µmol, 1 equiv.), and Et_3N (0.231 mL, 1.66 mmol, 3 equiv.). Flash column chromatography (5–30% MeCN in DCM) gave the product as a white solid (92 mg, 57%).
^{1}H NMR (500 MHz, CD_3CN): δ 7.32 – 7.27 (m, 1H), 7.10 – 6.94 (m, 3H), 4.32 (d, *J* = 17.2 Hz, 2H), 4.14 (d, *J* = 17.2 Hz, 2H), 3.76 (s, 3H), 3.06 (s, 3H).
^{13}C NMR (126 MHz, CD_3CN): δ 168.7, 159.1, 129.8, 124.0, 123.5, 116.3, 115.4, 99.3, 61.5, 55.2, 47.9. The carbon bearing boron was not observed.
^{11}B NMR (96 MHz, DMSO-d_6): δ 6.3.
$υ_{max}$ (solid): 3013, 2197, 1767, 1574, 1464, 1290, 1202, 1018 cm^{-1}.
HRMS (ESI) *m/z*: $[M–H]^-$ Calcd for $C_{14}H_{13}BNO_5$ 286.0892; Found 286.0891.

Compound S15

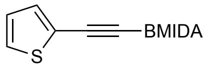

Prepared according to General Procedure B using $Pd(PPh_3)_2Cl_2$ (35.1 mg, 0.05 mmol, 5 mol%), acetylene boronic acid MIDA ester (181.0 mg, 1 mmol, 1.0 equiv.), 2-iodothiophene (133 µL, 1.2 mmol, 1.2 equiv.), CuI (19.1 mg, 0.1 mmol, 10 mol%), and Et_3N (420 µL, 3.0 mmol, 3.0 equiv.). Flash column chromatography (silica gel, 5–25% MeCN in DCM) gave the product as a pale yellow solid (151 mg, 57%).
^{1}H NMR (400 MHz, Acetone-d_6): δ 7.50 (dd, J = 5.2, 1.1 Hz, 1H), 7.30 (dd, J = 3.6, 1.2 Hz, 1H), 7.05 (dd, J = 5.2, 3.6 Hz, 1H), 4.34 (d, J = 17.0 Hz, 2H), 4.19 (d, J = 17.0 Hz, 2H), 3.30 (s, 3H).
^{13}C NMR (101 MHz, Acetone-d_6): δ 168.5, 133.6, 128.8, 128.1, 123.6, 93.3, 62.4, 48.5. The carbon bearing boron was not observed.
^{11}B NMR (96 MHz, Acetone-d_6): δ 6.5.
ν_{max} (solid): 3019, 2189, 1767, 1514, 1464, 1450, 1422 cm^{-1}.
HRMS (ESI) *m/z*: [M+Na]$^+$ Calcd for $C_{11}H_{10}BNNaO_4S$ 286.0321; Found 286.0317.

Compound S16

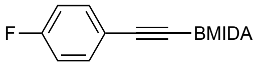

Prepared using General Procedure B using 4-fluoroiodobenzene (444 mg, 2 mmol, 1 equiv.), $Pd(PPh_3)_2Cl_2$ (70.2 mg, 0.1 µmol, 5 mol%), CuI (38.1 mg, 0.2 mmol, 10 mol%), acetylene boronic acid MIDA ester (434 mg, 2.40 mmol, 1.2 equiv.), and Et_3N (0.84 mL, 6 mmol, 3 equiv.). After work-up, the crude material was triturated with Et_2O to afford the product as an off-white solid (522 mg, 95%).
^{1}H NMR (400 MHz, DMSO-d_6): δ 7.58 – 7.51 (m, 2H), 7.25 – 7.22 (m, 2H), 4.32 (d, J = 17.1 Hz, 2H), 4.14 (d, J = 17.1 Hz, 2H), 3.07 (s, 3H).
^{13}C NMR (126 MHz, DMSO-d_6): δ 168.7, 162.1 (d, ^1J = 247.6 Hz), 133.9 (d, ^3J = 8.6 Hz), 118.9 (d, ^4J = 3.3 Hz), 115.9 (d, ^2J = 22.0 Hz), 98.3, 61.5, 47.9. The carbon bearing boron was not observed.
^{19}F NMR (376 MHz, DMSO-d_6): δ –110.3.
^{11}B NMR (96 MHz, DMSO-d_6): δ 6.0.
υ_{max} (solid): 1768, 1506, 1292, 1234, 1022, 837 cm^{-1}.
HRMS (ESI) *m/z*: [M+Na]$^+$ Calcd for $C_{13}H_{11}BFNO_4Na$ 298.0657; Found 298.0655.

Compound S17

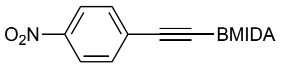

Prepared according to General Procedure B using 4-iodonitrobenzene (388 mg, 1.56 mmol, 1.20 equiv.), Pd(dppf)Cl_2 (47.6 mg, 65.0 µmol, 5 mol%), CuI (24.8 mg, 130 µmol, 10 mol%), acetylene boronic acid MIDA ester (235 mg, 1.30 mmol, 1 equiv.), and Et_3N (0.544 mL, 3.90 mmol, 3 equiv.). Flash column chromatography (5–30% MeCN in DCM) gave the product as a yellow solid (323 mg, 82%).
^{1}H NMR (500 MHz, DMSO-d_6): δ 8.27 – 8.20 (m, 2H), 7.80 – 7.71 (m, 2H), 4.35 (d, J = 17.2 Hz, 2H), 4.17 (d, J = 17.1 Hz, 2H), 3.10 (s, 3H).
^{13}C NMR (126 MHz, DMSO-d_6): δ 168.7, 147.1, 132.9, 129.1, 123.9, 97.4, 61.6, 48.0. The carbon bearing boron was not observed.
^{11}B NMR (96 MHz, DMSO-d_6): δ 6.4.
υ_{max} (solid): 2361, 1765, 1514, 1348, 1072, 1022 cm^{-1}.
HRMS (ESI) *m/z*: [M+Na]$^+$ Calcd for $C_{13}H_{11}BN_2NaO_6$ 325.0608; Found 325.0603.

Compound S18

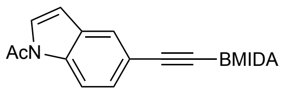

Prepared using General Procedure B using 5-iodo-*N*-acyl-indole (570 mg, 2 mmol, 1 equiv.), $Pd(PPh_3)_2Cl_2$ (70.2 mg, 0.1 µmol, 5 mol%), CuI (38.1 mg, 0.2 mmol, 10 mol%), acetylene boronic acid MIDA ester (434 mg, 2.40 mmol, 1.2 equiv.), and Et_3N (0.84 mL, 6 mmol, 3 equiv.). Flash column chromatography (silica gel, 20% MeCN in DCM) gave the product as a beige solid (516 mg, 76%).

^{1}H NMR (400 MHz, DMSO-d_6): δ 8.31 (d, J = 8.6 Hz, 1H), 7.92 (d, J = 3.8 Hz, 1H), 7.78 (dd, J = 1.6, 0.7 Hz, 1H), 7.43 (dd, J = 8.6, 1.7 Hz, 1H), 6.75 (dd, J = 3.8, 0.7 Hz, 1H), 4.32 (d, J = 17.2 Hz, 2H), 4.15 (d, J = 17.1 Hz, 2H), 3.09 (s, 3H), 2.65 (s, 3H).
^{13}C NMR (126 MHz, DMSO-d_6): δ 169.7, 168.8, 134.6, 130.3, 128.5, 128.0, 124.4, 117.3, 116.0, 107.9, 100.1, 61.5, 47.9, 23.8. The carbon bearing boron was not observed.
^{11}B NMR (96 MHz, DMSO-d_6): δ 7.2.
υ_{max} (solid): 1776, 1755, 1712, 1463, 1217, 1022, 894, 715 cm^{-1}.
HRMS (ESI) *m/z*: $[M+Na]^+$ Calcd for $C_{17}H_{15}BN_2O_5Na$ 361.0972; Found 361.0957.

Compound S19

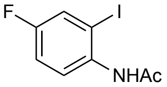

To a mixture of 4-fluoro-2-iodoaniline (237 mg, 1.00 mmol, 1.00 equiv.) and Et_3N (307 µL, 2.2 mmol, 2.2 equiv.) in DCM (5 mL, 0.2 M) was added Ac_2O (113 µL, 1.2 mmol, 1.2 equiv.) dropwise. The mixture was heated to reflux for 24 h then allowed to cool to room temperature. The mixture was treated with sat. aq. $NaHCO_3$ (10 mL) and the layers were separated. The aqueous layer was extracted with DCM (10 mL). The combined organic extracts were dried over Na_2SO_4, filtered, and concentrated to a residue that was purified by flash column chromatography (silica gel, 30–50% EtOAc in hexane) to afford the product as a beige solid (241 mg, 89%).
^{1}H NMR (400 MHz, $CDCl_3$): δ 8.09 (dd, J = 9.1, 5.5 Hz, 1H), 7.50 (dd, J = 7.7, 2.9 Hz, 1H), 7.26 (br s, 1H), 7.08 (ddd, J = 9.1, 7.8, 2.9 Hz, 1H), 2.23 (s, 3H).
^{13}C NMR (126 MHz, $CDCl_3$): δ 168.4, 158.8 (d, ^{1}J = 248.9 Hz), 134.9 (d, ^{4}J = 3.1 Hz), 125.5 (d, ^{2}J = 24.8 Hz), 123.3 (d, ^{3}J = 7.9 Hz), 116.2 (d, ^{2}J = 21.7 Hz), 89.9 (d, ^{3}J = 8.3 Hz), 24.8.
^{19}F NMR (471 MHz, $CDCl_3$): δ –116.1.
Spectral data in agreement with literature values.[8]

Compound S20

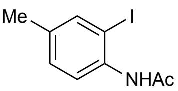

A solution of 4-methyl-2-iodoaniline (1.17 g, 5.00 mmol, 1.00 equiv.) and Et_3N (767 µL, 5.50 mmol, 1.10 equiv.) in DCM (16.7 mL, 0.33 M) was cooled to 0 °C. AcCl (945 µL, 13.0 mmol, 2.60 equiv.) was added dropwise and the mixture was allowed to warm to room temperature and stir for 68 h. Methanol (10 mL) was added and the mixture was stirred for 10 min before water (20 mL) was added. The layers were separated, and the aqueous layer was extracted with DCM (20 mL). The combined organics were dried over Na_2SO_4, filtered, and concentrated to give a residue that was purified by flash column chromatography (silica gel, 20–40% EtOAc in hexanes) to give the product as a pale yellow solid (1.03 g, 75%).
^{1}H NMR (500 MHz, $CDCl_3$): δ 8.02 (d, J = 8.3 Hz, 1H), 7.60 (d, J = 2.0 Hz, 1H), 7.32 (br s, 1H), 7.16 – 7.12 (m, 1H), 2.28 (s, 3H), 2.23 (s, 3H).
^{13}C NMR (126 MHz, $CDCl_3$): δ 168.3, 139.1, 136.2, 135.9, 130.1, 122.1, 90.3, 24.9, 20.5.
Spectral data in agreement with literature values.[9]

Compound S21

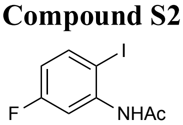

To a mixture of 2-fluoro-5-iodoaniline (510 mg, 2.15 mmol, 1.00 equiv.) and Et_3N (330 µL, 2.37 mmol, 1.10 equiv.) in DCM (7.2 mL, 0.3 M) at 0 °C, was added AcCl (406 µL, 5.59 mmol, 2.60 equiv.) dropwise. The mixture was allowed to warm to room temperature and stir for 66 hours. MeOH (10 mL) was then added, followed by H_2O (20 mL). The layers were separated, and the aqueous layer extracted with DCM (20 mL). The combined organic extracts were dried over Na_2SO_4, filtered, and concentrated to a residue that was purified

by flash column chromatography (silica gel, 10–30% EtOAc in hexane) to give the product as a white solid (349 mg, 58%).
^{1}H NMR (500 MHz, $CDCl_3$): δ 8.17 – 8.15 (m, 1H), 7.72 – 7.69 (m, 1H), 7.47 (br s, 1H), 6.65 – 6.62 (m, 1H), 2.25 (s, 3H).
^{13}C NMR (126 MHz, $CDCl_3$): δ 168.4, 163.4 (d, ^{1}J = 246.4 Hz), 139.6 (d, ^{3}J = 11.7 Hz), 139.2 (d, ^{3}J = 8.9 Hz), 113.1 (d, ^{2}J = 22.7 Hz), 109.3 (d, ^{2}J = 28.4 Hz), 81.9, 25.1.
^{19}F NMR (471 MHz, $CDCl_3$): δ –110.5.
Spectral data in agreement with literature values.[10]

Compound S22

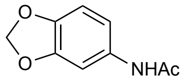

To a solution of benzo[*d*][1,3]dioxol-5-amine (960 mg, 7.00 mmol, 1.0 equiv.) in 1,4-dioxane (10 mL, 0.07 M) at 0 °C, was added acetic anhydride (79.4 µL, 8.40 mmol, 1.2 equiv.). The resulting mixture was allowed to warm to room temperature and stirred for 3 h. MeOH was then added and the resulting mixture stirred at room temperature for 10 min, then concentrated to give a residue that was purified by flash column chromatography (silica gel, 30–50%) EtOAc in petroleum ether to give the product as a grey/brown solid (1.11 g, 89%).

^{1}H NMR (500 MHz, $CDCl_3$): δ 7.21 (d, J = 1.9 Hz, 1H), 7.11 (br s, 1H), 6.77 – 6.71 (m, 2H), 5.95 (s, 2H), 2.15 (s, 3H).
^{13}C NMR (126 MHz, $CDCl_3$): δ 168.3, 147.9, 144.4, 132.1, 113.3, 108.2, 103.1, 101.4, 24.6.
Spectral data in agreement with literature values.[11]

Compound S23

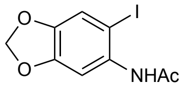

To a mixture of 3,4-(methylenedioxy)acetanilide (1.12 g, 6.23 mmol, 1.1 equiv.) and AcOH (971 µL, 17.0 mmol, 3.0 equiv.) in DCM (13.5 mL, 0.42 M) at room temperature was added a solution of ICl in DCM (919 mg, 5.66 mmol, 1.0 equiv, 0.57 M) via syringe. After 24 h, sat. aq. $Na_2S_2O_5$ was added, and the layers were separated. The aqueous layer was extracted with DCM (10 mL). The combined organics were dried over Na_2SO_4, filtered, and concentrated to a residue that was purified by flash column chromatography (silica gel, 20–30% EtOAc in petrol) to give the product as a white solid (500 mg, 29%).
^{1}H NMR (500 MHz, DMSO-d_6): δ 9.37 (s, 1H), 7.37 (s, 1H), 6.95 (s, 1H), 6.06 (s, 2H), 2.00 (s, 3H).^{13}C NMR (126 MHz, DMSO-d_6): δ 168.5, 147.9, 146.3, 133.7, 116.9, 108.6, 102.1, 86.0, 23.1.
υ_{max} (solid): 3244, 1645, 1533, 1476, 1234 cm^{-1}.
HRMS (ESI) *m/z*: $[M+Na]^+$ Calcd for $C_9H_8INNaO_3$ 327.9447; Found 327.9429.

Compound S24

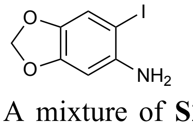

A mixture of **S23** (458 mg, 1.50 mmol, 1.0 equiv.) and NaOH pellets (3.00 g, 75.0 mmol, 55 equiv.) in EtOH/H_2O (4.4/1, 75 mL, 0.02 M) was refluxed for 4 h. The reaction mixture was concentrated to give a crude residue, which was treated with water (10 mL) and extracted with DCM (5 x 10 mL). The combined organics were dried over Na_2SO_4, filtered, and concentrated to give the product as a beige solid (360 mg, >99%).
^{1}H NMR (500 MHz, $CDCl_3$): δ 7.05 (s, 1H), 6.38 (s, 1H), 5.88 (s, 2H), 3.85 (br s, 2H).
^{13}C NMR (126 MHz, $CDCl_3$): δ 149.3, 141.8, 141.3, 117.4, 101.3, 97.0, 70.9.
υ_{max} (solid): 3304, 2897, 1673, 1603, 1497, 1466, 1258, 1229, 1196, 1113 cm^{-1}.
HRMS (ESI) *m/z*: $[M+H]^+$ Calcd for $C_7H_7INO_2$ 263.9521; Found 263.9509.

Compound S25

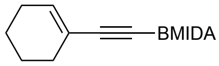

Prepared according to General Procedure C using 1-ethynylcyclohexene (3.19 mL, 30 mmol, 1.0 equiv.), EtMgBr (3.0 M in Et_2O, 12 mL, 36 mmol, 1.2 equiv.), $B(OMe)_3$ (6.69 mL, 60 mmol, 2.0 equiv.), and *N*-methyliminodiacetic acid (8.83 g, 60 mmol, 2.0 equiv.). Flash column chromatography (silica gel, 10–30% MeCN in DCM) gave the product as a white solid (4.99 g, 64%).

^{1}H NMR (500 MHz, DMSO-d_6): δ 6.16 – 6.07 (m, 1H), 4.24 (d, *J* = 17.2 Hz, 2H), 4.06 (d, *J* = 17.1 Hz, 2H), 2.96 (s, 3H), 2.07 – 2.04 (m, 4H), 1.60 – 1.47 (m, 4H).

^{13}C NMR (126 MHz, DMSO-d_6): δ 167.8, 135.3, 120.8, 102.0, 61.4, 47.5, 28.8, 25.2, 22.0, 21.3. The carbon bearing boron was not observed.

^{11}B NMR (160 MHz, DMSO-d_6) δ 6.4.

ν_{max} (solid): 2361, 2343, 1761, 1749, 1734, 1558, 1506, 1456 cm^{-1}.

HRMS (ESI) *m/z*: [M+Na]$^+$ Calcd for $C_{13}H_{16}BNaNO_4$ 284.1070; Found 284.1058.

Compound S26

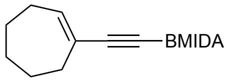

Prepared using General Procedure B using cycloheptenyl triflate (244 mg, 1 mmol, 1 equiv.), $Pd(PPh_3)_2Cl_2$ (35.1 mg, 50 μmol, 5 mol%), CuI (19 mg, 0.1 mmol, 10 mol%), ethynyl boronic acid MIDA ester (217 mg, 1.20 mmol, 1.2 equiv.), Et_3N (0.42 mL, 3 mmol, 3 equiv.). Flash column chromatography (silica gel, 0–10% MeCN in DCM) gave the product as a white solid (165 mg, 60%).

^{1}H NMR (500 MHz, DMSO-d_6): δ 6.29 (t, *J* = 6.7 Hz, 1H), 4.24 (d, *J* = 17.1 Hz, 2H), 4.06 (d, *J* = 17.1 Hz, 2H), 2.96 (s, 3H), 2.31 – 2.23 (m, 2H), 2.20 – 2.10 (m, 2H), 1.74 – 1.64 (m, 2H), 1.55 – 1.39 (m, 4H).

^{13}C NMR (126 MHz, DMSO-d_6): δ 168.7, 140.6, 126.4, 103.2, 61.4, 47.7, 33.5, 31.4, 28.5, 26.1. The carbon bearing boron was not observed.

^{11}B NMR (96 MHz, DMSO-d_6): δ 7.1.

υ_{max} (solid): 1766, 1290, 1236, 1022, 1002, 956, 858 cm^{-1}.

HRMS (ESI) *m/z*: [M+Na]$^+$ Calcd for $C_{14}H_{18}BNO_4Na$ 298.1221; Found 298.1214.

Compound S27

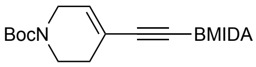

Prepared according to General Procedure B using $Pd(dppf)Cl_2$ (73.2 mg, 0.10 mmol, 5 mol%), *tert*-butyl 4-(((trifluoromethyl)sulfonyl)oxy)-3,6-dihydropyridine-carboxylate (795 mg, 1.20 mmol, 1.20 equiv.), CuI (38.1 mg, 0.20 mmol, 10 mol%), acetylene boronic acid MIDA ester (181 mg, 1.0 mmol, 1.0 equiv.), and Et_3N (836 μL, 3.0 mmol, 3.0 equiv.). Stirred at 50 °C for 16 h. Flash column chromatography (5–20% MeCN in DCM) followed by trituration from acetone with Et_2O to give the product as a pale yellow solid (235 mg, 65%).

^{1}H NMR (500 MHz, DMSO-d_6): δ 6.11 (br s, 1H), 4.27 (d, *J* = 17.2 Hz, 2H), 4.07 (d, *J* = 17.1 Hz, 2H), 3.89 (br s, 2H), 3.39 (t, *J* = 5.7 Hz, 2H), 2.97 (s, 3H), 2.17 – 2.14 (m, 2H), 1.40 (s, 9H).

^{13}C NMR (126 MHz, DMSO-d_6): δ 168.7, 153.8, 132.1, 118.6, 99.8, 79.1, 61.4, 47.8, 43.3, 42.8, 28.5, 28.1.

^{11}B NMR (96 MHz, DMSO-d_6): δ 7.3.

υ_{max} (solid): 3976, 2374, 2191, 1769, 1695, 1288, 1024 cm^{-1}.

HRMS (ESI) *m/z*: [M+Na]$^+$ Calcd for $C_{17}H_{23}BN_2NaO_6$ 385.1547; Found 385.1537.

Compound S28

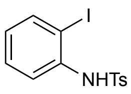

Tosyl chloride (0.92 g, 4.80 mmol, 1.05 equiv.) was added in one portion to a solution of 2-iodoaniline (1.00 g, 4.57 mmol, 1.00 equiv.) in pyridine (10 mL). The reaction mixture was stirred at room temperature for 3 h before addition of H_2O (20 mL). The mixture was extracted with DCM (3 x 20 mL). The combined organic extracts were then washed with aqueous $CuSO_4$ solution (2 x 20 mL), dried over Na_2SO_4, filtered, and concentrated to a residue that was purified by flash column chromatography (silica gel, 10–20% EtOAc in hexane) to give the product as a pale yellow solid (1.54 g, 90%).
^{1}H NMR (500 MHz, $CDCl_3$): δ 7.68 – 7.59 (m, 4H), 7.30 (ddd, J = 8.4, 7.3, 1.4 Hz, 1H), 7.24 – 7.18 (m, 2H), 6.83 (ddd, J = 8.0, 7.4, 1.5 Hz, 1H), 6.79 (br s, 1H), 2.38 (s, 3H).
^{13}C NMR (126 MHz, $CDCl_3$): δ 144.4, 139.2, 137.6, 136.0, 129.8, 129.6, 127.6, 127.0, 122.6, 92.5, 21.7.
Spectral data in agreement with literature.[12]

4.2 Products from Table 1, Scheme 2, and Scheme 3
Compound 1b

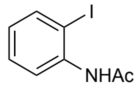

A solution of 2-iodoaniline (21.9 g, 100 mmol, 1.00 equiv.) and Et_3N (30.7 mL, 220 mmol, 2.20 equiv.) in DCM (250 mL) was cooled to 0 °C. Ac_2O (11.3 mL, 120 mmol, 1.20 equiv.) was added. The reaction mixture was then warmed to a gentle reflux for 18 h. The mixture was allowed to cool to room temperature before addition of sat. aq. $NaHCO_3$ (200 mL). The layers were separated, and the aqueous layer was extracted with DCM (2 x 100 mL). The combined organic extracts were then dried over Na_2SO_4, filtered, and concentrated to a residue that was purified by flash column chromatography (silica gel, 20–80% EtOAc in hexane) to give the product as a white solid (23.1 g, 88%).
^{1}H NMR (500 MHz, $CDCl_3$): δ 8.21 (d, J = 8.2 Hz, 1H), 7.80 – 7.73 (m, 1H), 7.41 (br s, 1H), 7.34 (ddd, J = 8.5, 7.5, 1.5 Hz, 1H), 6.86 – 6.83 (t, J = 7.4 Hz, 1H), 2.24 (s, 3H).
^{13}C NMR (126 MHz, $CDCl_3$): δ 168.3, 138.9, 138.3, 129.4, 126.1, 122.2, 90.1, 25.0.
Spectral data in agreement with literature values.[13]

Compound 2b

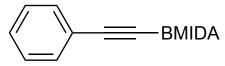

Prepared according to General Procedure C using phenylacetylene (5.11 g, 50 mmol, 1.0 equiv.), EtMgBr (3.28 M in Et_2O, 18.3 mL, 60 mmol, 1.2 equiv.), $B(OMe)_3$ (11.1 mL, 100 mmol, 2.0 equiv.), and *N*-methyliminodiacetic acid (14.7 g, 100 mmol, 2.0 equiv.). Flash column chromatography (silica gel, 0–30% MeCN in DCM) gave the product as a pale yellow solid (7.82 g, 61%).
^{1}H NMR (500 MHz, DMSO-d_6): δ 7.54 – 7.43 (m, 2H), 7.43 – 7.34 (m, 3H), 4.30 (d, J = 17.2 Hz, 2H), 4.13 (d, J = 17.2 Hz, 2H), 3.06 (s, 3H).
^{13}C NMR (126 MHz, DMSO-d_6): δ 168.8, 131.6, 128.9, 128.7, 122.4, 99.5, 61.5, 47.9. The carbon bearing boron was not observed.
^{11}B NMR (96 MHz, DMSO-d_6): δ 6.2.
Spectral data in agreement with literature values.[14]

Compound 3

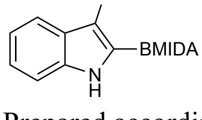

Prepared according to General Procedure A using $Pd(dppf)Cl_2$ (7.3 mg, 0.01 mmol, 5 mol%), propyne boronic acid MIDA ester (39.0 mg, 0.2 mmol, 1.0 equiv.), 2-iodoaniline (52.6 mg, 0.24 mmol, 1.2 equiv.), and NaOAc (41.0 mg, 0.5 mmol, 2.5 equiv.). Flash column chromatography (silica gel, 5–20% MeCN in DCM) gave the product as a tan solid (48.0 mg, 84%).

^{1}H NMR (400 MHz, Acetone-d_6): δ 9.87 (br s, 1H), 7.52 (dd, J = 8.0, 1.3 Hz, 1H), 7.37 – 7.35 (m, 1H), 7.08 (ddd, J = 8.0, 7.0, 1.2 Hz, 1H), 7.02 – 6.96 (m, 1H), 4.38 (d, J = 17.1 Hz, 2H), 4.15 (d, J = 17.1 Hz, 2H), 2.82 (s, 3H), 2.37 (s, 3H).
^{13}C NMR (101 MHz, Acetone-d_6): δ 169.2, 139.0, 130.7, 122.4, 119.2, 119.0, 118.1, 111.9, 62.8, 48.0, 9.9. The carbon bearing boron was not observed.
^{11}B NMR (96 MHz, Acetone-d_6): δ 10.7.
ν_{max} (solid): 3366, 1771, 1749, 1539, 1458, 1333, 1294, 1244, 1211, 1138 cm^{-1}.
HRMS (ESI) *m/z*: [M+H]$^+$ Calcd for $C_{14}H_{16}BN_2O_4$ 287.1203; Found 287.1197.

Compound 4

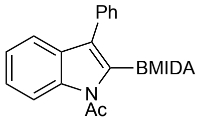

Prepared according to General Procedure D using $Pd(OAc)_2$ (4.5 mg, 20 µmol, 10 mol%), phenylethynylboronic acid MIDA ester (51.4 mg, 0.2 mmol, 1.0 equiv.), NaOAc (41.0 mg, 0.5 mmol, 2.50 equiv.), LiCl (17.0 mg, 0.4 mmol, 2 equiv.), and *N*-(2-iodophenyl)acetamide (62.6 mg, 0.24 mmol, 1.2 equiv.). Flash column chromatography (silica gel, 5–20% MeCN in DCM) gave the product as a light brown solid (47 mg, 60%).
^{1}H NMR (500 MHz, CD_3CN): δ 7.81 (d, J = 8.5 Hz, 1H), 7.47 – 7.33 (m, 4H), 7.32 – 7.16 (m, 4H), 4.28 – 4.21 (m, 1H), 3.99 (d, J = 17.1 Hz, 3H), 3.20 (s, 3H), 2.85 (s, 3H).
^{13}C NMR (126 MHz, CD_3CN): δ 173.0, 137.7, 136.8, 136.2, 133.1, 132.2, 130.2, 129.2, 128.0, 126.2, 123.7, 121.2, 115.1, 66.3, 51.6, 27.7. The carbon bearing boron was not observed.
^{11}B NMR (96 MHz, CD_3CN): δ 10.9.
υ_{max} (film): 2922, 1740, 1686, 1310, 1030, 1012, 748 cm^{-1}.
HRMS (ESI) *m/z*: [M+Na]$^+$ Calcd for $C_{23}H_{19}BNaN_2O_5$ 413.1285; Found 413.1288.

Compound 5

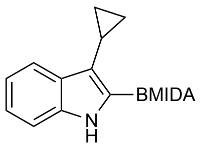

Prepared according to General Procedure A using $Pd(dppf)Cl_2$ (7.3 mg, 0.01 mmol, 5 mol%), **S3** (44.2 mg, 0.2 mmol, 1.0 equiv.), 2-iodoaniline (52.6 mg, 0.24 mmol, 1.2 equiv.), and NaOAc (41.0 mg, 0.5 mmol, 2.5 equiv.). Flash column chromatography (silica gel, 0–35% MeCN in DCM) gave the product as a tan solid (57 mg, 91%).
^{1}H NMR (400 MHz, Acetone-d_6): δ 9.88 (br s, 1H), 7.65 – 7.63 (m, 1H), 7.39 – 7.37 (m, 1H), 7.05 (ddd, J = 8.1, 6.9, 1.2 Hz, 1H), 6.95 (ddd, J = 8.0, 6.9, 1.1 Hz, 1H), 4.39 (d, J = 17.0 Hz, 2H), 4.18 (d, J = 17.0 Hz, 2H), 2.87 (s, 3H), 1.98 (tt, J = 8.3, 5.5 Hz, 1H), 0.91 – 0.83 (m, 4H).
^{13}C NMR (126 MHz, Acetone-d_6): δ 169.2, 138.7, 129.8, 123.6, 122.2, 120.3, 119.1, 112.3, 63.0, 48.1, 8.1, 6.6. The carbon bearing boron was not observed.
^{11}B NMR (96 MHz, Acetone-d_6): δ 10.6.
ν_{max} (solid): 1761, 1533, 1456, 1290, 1194, 1032, 1009 cm^{-1}.
HRMS (ESI) *m/z*: [M–H]$^-$ Calcd for $C_{16}H_{16}BN_2O_4$ 311.1209; Found 311.1212.

Compound 6

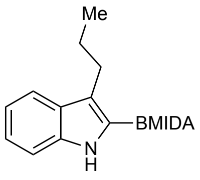

Prepared according to General Procedure A using Pd(dppf)Cl_2 (7.3 mg, 0.01 mmol, 5 mol%), **S4** (44.6 mg, 0.2 mmol, 1.0 equiv.), 2-iodoaniline (52.6 mg, 0.24 mmol, 1.2 equiv.), and NaOAc (41.0 mg, 0.5 mmol, 2.5 equiv.). Flash column chromatography (silica gel, 0–35% MeCN in DCM) gave the product as a tan solid (59 mg, 94%).

^{1}H NMR (500 MHz, CD_3CN): δ 9.05 (br s, 1H), 7.59 – 7.57 (m, 1H), 7.36 – 7.34 (m, 1H), 7.11 (ddd, *J* = 8.2, 6.9, 1.2 Hz, 1H), 7.00 (ddd, *J* = 8.0, 6.9, 1.0 Hz, 1H), 4.10 (d, *J* = 17.2 Hz, 2H), 3.92 (d, *J* = 17.2 Hz, 2H), 2.74 – 2.69 (m, 2H), 2.59 (s, 3H), 1.67 – 1.59 (m, 2H), 0.95 (t, *J* = 7.4 Hz, 3H).

^{13}C NMR (126 MHz, CD_3CN): δ 169.3, 138.9, 130.0, 124.3, 122.8, 119.8, 119.3, 112.0, 62.9, 48.4, 28.2, 26.1, 14.6. The carbon bearing boron was not observed.

ν_{max} (solid): 3360, 2953, 2361, 1765, 1744, 1541, 1454, 1225, 1292, 1034, 1009 cm^{-1}.

HRMS (ESI) *m/z*: $[M–H]^-$ Calcd for $C_{16}H_{18}BN_2O_4$ 313.1365; Found 313.1368.

Compound 7

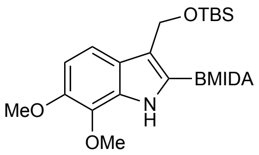

Prepared according to General Procedure A using Pd(dppf)Cl_2 (7.3 mg, 0.01 mmol, 5 mol%), **S5** (65.0 mg, 0.2 mmol, 1.0 equiv.), 2,3-dimethoxy-6-iodoaniline (67.0 mg, 0.24 mmol, 1.2 equiv.), and NaOAc (41.0 mg, 0.5 mmol, 2.5 equiv.). Flash column chromatography (silica gel, 5–30% MeCN in DCM) gave the product as a light pink solid (40.0 mg, 42%).

^{1}H NMR (500 MHz, Acetone-*d6*): δ 9.96 (br s, 1H), 7.30 (d, *J* = 8.6 Hz, 1H), 6.87 (d, *J* = 8.6 Hz, 1H), 4.95 (s, 2H), 4.36 (d, *J* = 16.8 Hz, 2H), 4.16 (d, *J* = 16.8 Hz, 2H), 3.88 (app d, 6H), 2.89 (s, 3H), 0.91 (s, 9H), 0.15 (s, 6H).

^{13}C NMR (126 MHz, Acetone-*d6*): δ 169.2, 148.1, 135.7, 133.0, 126.8, 123.0, 114.2, 109.5, 62.9, 60.8, 57.8, 57.2, 48.3, 26.5, 19.1, −5.0. The carbon bearing boron was not observed.

^{11}B NMR (96 MHz, Acetone-d_6): δ 10.5.

υ_{max} (solid): 3310, 2930, 1765, 1517, 1449, 1248, 1088,1045, 999 cm^{-1}.

HRMS (ESI) *m/z*: $[M+Na]^+$ Calcd for $C_{24}H_{33}BNaN_2O_7Si$ 499.2048; Found 499.2034.

Compound 8

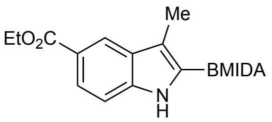

Prepared according to General Procedure A using Pd(dppf)Cl_2 (7.3 mg, 10 μmol, 5 mol%), propyne boronic acid MIDA ester (39.0 mg, 0.2 mmol, 1.0 equiv.), 2-iodobenzocaine (52.6 mg, 0.24 mmol, 1.2 equiv.), and NaOAc (41.0 mg, 0.5 mmol, 2.5 equiv.). Flash column chromatography (silica gel, 5–20% MeCN in DCM) gave the product as an off-white solid (72 mg, 71%).

^{1}H NMR (500 MHz, DMSO-d_6): δ 10.99 (s, 1H), 8.19 – 8.15 (m, 1H), 7.69 (dd, *J* = 8.5, 1.6 Hz, 1H), 7.39 (d, *J* = 8.5 Hz, 1H), 4.39 (d, *J* = 17.3 Hz, 2H), 4.30 (q, *J* = 7.1 Hz, 2H), 4.12 (d, *J* = 17.3 Hz, 2H), 2.57 (s, 3H), 2.31 (s, 3H), 1.33 (t, *J* = 7.1 Hz, 3H).

^{13}C NMR (126 MHz, DMSO-d_6): δ 169.2, 166.9, 140.1, 128.8, 122.2, 120.9, 119.6, 118.2, 111.1, 61.8, 60.0, 47.4, 14.4, 9.4. The carbon bearing boron was not observed.

^{11}B NMR (96 MHz, DMSO-d_6): δ 12.9.

υ_{max} (solid): 1767, 1694, 1449, 1250, 1099, 1034, 1001 cm^{-1}.

HRMS (ESI) *m/z*: $[M+Na]^+$ Calcd for $C_{19}H_{19}BNaN_2O_6$ 381.1234; Found 381.1222.

Compound 9

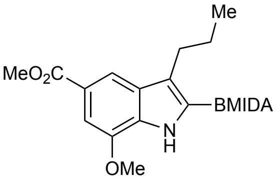

Prepared using General Procedure A using Pd(dppf)Cl_2 (7.3 mg, 10 µmol, 5 mol%), **S4** (44.6 mg, 0.2 mmol, 1.0 equiv.), NaOAc (41.0 mg, 0.5 mmol, 2.50 equiv.), and methyl 4-amino-3-iodo-5-methoxybenzoate (73.7 mg, 0.24 mmol, 1.2 equiv.). Flash column chromatography (silica gel, 10–30% MeCN in DCM) gave the product as a light brown powder (51 mg, 63%).

^{1}H NMR (500 MHz, Acetone-d_6): δ 10.22 (s, 1H), 8.00 – 7.99 (m, 1H), 7.25 (d, J = 1.2 Hz, 1H), 4.42 (d, J = 17.2 Hz, 2H), 4.19 (d, J = 17.2 Hz, 2H), 3.96 (s, 3H), 3.87 (s, 3H), 2.96 (s, 3H), 2.84 – 2.78 (m, 2H), 1.71 – 1.62 (m, 2H), 0.98 (t, J = 7.4 Hz, 3H).

^{13}C NMR (126 MHz, Acetone-d_6): δ 169.1, 168.4, 146.7, 132.1, 130.7, 126.5, 122.0, 116.1, 102.7, 63.3, 55.7, 51.9, 48.6, 28.1, 26.4, 14.7. The carbon bearing boron was not observed.

^{11}B NMR (96 MHz, Acetone-d_6): δ 10.5.

υ_{max} (solid): 2953, 1763, 1697, 1435, 1254, 1229, 1034 cm^{-1}.

HRMS (ESI) *m/z*: [M–H]$^-$ Calcd for $C_{19}H_{22}BN_2O_7$ 401.1526; Found 401.1525.

Compound 10

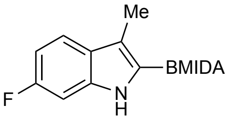

Prepared using General Procedure A using Pd(dppf)Cl_2 (7.3 mg, 10 µmol, 5 mol%), 1-propynylboronic acid MIDA ester (39.0 mg, 0.2 mmol, 1.0 equiv.), NaOAc (41.0 mg, 0.5 mmol, 2.50 equiv.), and **S7** (56.9 mg, 0.24 mmol, 1.2 equiv.). Flash column chromatography (silica gel, 5–20% MeCN in DCM) gave the product as a light brown powder (62 mg, >99%).

^{1}H NMR (500 MHz, Acetone-d_6): δ 9.98 (br s, 1H), 7.49 (dd, J = 8.6, 5.4 Hz, 1H), 7.07 (dd, J = 10.1, 2.3 Hz, 1H), 6.80 (ddd, J = 9.8, 8.6, 2.3 Hz, 1H), 4.39 (d, J = 17.1 Hz, 2H), 4.16 (d, J = 17.2 Hz, 2H), 2.85 (s, 3H), 2.35 (s, 3H).

^{13}C NMR (126 MHz, Acetone-d_6): δ 169.1, 160.9 (d, ^1J = 234.8 Hz), 138.8 (d, ^3J = 12.5 Hz), 127.6, 120.2 (d, ^3J = 10.5 Hz), 118.4, 107.5 (d, ^2J = 25.0 Hz), 97.7 (d, ^2J = 25.5 Hz), 62.8, 48.0, 9.9. The carbon bearing boron was not observed.

^{19}F NMR (471 MHz, Acetone-d_6): δ –123.7.

^{11}B NMR (96 MHz, Acetone-d_6): δ 10.5.

υ_{max} (solid): 1763, 1075, 1456, 1288, 1209, 1032 cm^{-1}.

HRMS (ESI) *m/z*: [M–H]$^-$ Calcd for $C_{14}H_{13}BFN_2O_4$ 303.0958; Found 303.0959.

Compound 11

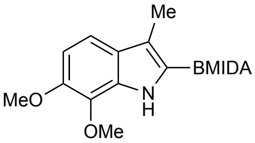

Prepared according to General Procedure A using Pd(dppf)Cl_2 (7.3 mg, 0.01 mmol, 5 mol%), propyne boronic acid MIDA ester (39.0 mg, 0.2 mmol, 1.0 equiv.), **S8** (67.0 mg, 0.24 mmol, 1.2 equiv.), and NaOAc (41.0 mg, 0.5 mmol, 2.5 equiv.). Flash column chromatography (silica gel, 5–30% MeCN in DCM) gave the product as a tan solid (45.0 mg, 65%).

^{1}H NMR (500 MHz, Acetone-d_6): δ 9.66 (br s, 1H), 7.17 (d, J = 8.5 Hz, 1H), 6.82 (d, J = 8.6 Hz, 1H), 4.35 (d, J = 17.1 Hz, 2H), 4.13 (d, J = 17.1 Hz, 2H), 3.86 (app d, 6H), 2.89 (s, 3H), 2.31 (s, 3H).

^{13}C NMR (126 MHz, Acetone-d_6): δ 169.3, 148.2, 135.5, 133.3, 128.0, 118.9, 114.2, 108.8, 62.9, 60.7, 57.9, 48.1, 10.0. The carbon bearing boron was not observed.

^{11}B NMR (96 MHz, Acetone-d_6): δ 10.7.
υ_{max} (solid): 3383, 2932, 1757, 1294, 1217 cm^{-1}.
HRMS (ESI) *m/z*: $[M+Na]^+$ Calcd for $C_{18}H_{19}BNaN_2O_6$ 369.1234; Found 369.1228.

Compound 12

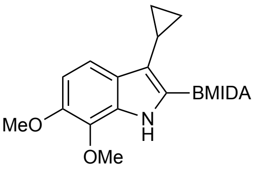

Prepared according to General Procedure A using Pd(dppf)Cl_2 (7.3 mg, 0.01 mmol, 5 mol%), **S3** (44.2 mg, 0.2 mmol, 1.0 equiv.) **S8** (67.0 mg, 0.24 mmol, 1.2 equiv.), and NaOAc (41.0 mg, 0.5 mmol, 2.5 equiv.). Flash column chromatography (silica gel, 5–30% MeCN in DCM) gave the product as a light pink solid (60.0 mg, 81%).
^1H NMR (500 MHz, DMSO-d_6): δ 10.30 (s, 1H), 7.21 (d, J = 8.7 Hz, 1H), 6.74 (d, J = 8.7 Hz, 1H), 4.34 (d, J = 17.3 Hz, 2H), 4.06 (d, J = 17.3 Hz, 2H), 3.81 (d, J = 13.1 Hz, 6H), 2.65 (s, 3H), 1.88 (tt, J = 8.5, 5.4 Hz, 1H), 0.81 – 0.75 (m, 2H), 0.68 (dt, J = 5.5, 2.8 Hz, 2H).
^{13}C NMR (126 MHz, DMSO-d_6): δ 169.4, 146.4, 134.2, 131.8, 125.2, 122.9, 114.2, 107.5, 62.4, 60.4, 57.2, 47.8, 7.3, 6.0. The carbon bearing boron was not observed.
^{11}B NMR (96 MHz, DMSO-d_6): δ 10.6.
υ_{max} (solid): 3374, 2963, 1748, 1450, 1246 cm^{-1}.
HRMS (EI) *m/z*: $[M]^+$ Calcd for $C_{18}H_{21}BN_2O_6$ 372.1493; Found 372.1491.

Compound 13

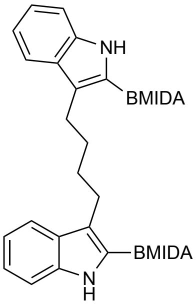

Prepared using General Procedure A using Pd(dppf)Cl_2 (14.6 mg, 20 μmol, 10 mol%), **S9** (83.2 mg, 0.2 mmol, 1.0 equiv.), NaOAc (82.0 mg, 1.0 mmol, 5.0 equiv.), and 2-iodoaniline (105 mg, 0.48 mmol, 2.4 equiv.). Flash column chromatography (silica gel, 5–50% MeCN in DCM) gave the product as a brown solid (89.0 mg, 74%).
^1H NMR (500 MHz, Acetone-d_6): δ 9.86 (s, 2H), 7.60 – 7.58 (m, 2H), 7.35 – 7.33 (m, 2H), 7.06 (ddd, J = 8.1, 6.9, 1.2 Hz, 2H), 6.96 (ddd, J = 7.9, 6.9, 1.0 Hz, 2H), 4.33 (d, J = 17.2 Hz, 4H), 4.06 (d, J = 17.2 Hz, 4H), 2.92 – 2.89 (m, 4H), 2.76 (s, 6H), 1.78 (p, J = 3.8 Hz, 4H).
^{13}C NMR (126 MHz, Acetone-d_6): δ 169.3, 139.1, 130.2, 124.6, 122.4, 119.8, 119.0, 112.0, 63.0, 48.4, 33.8, 26.0. The carbon bearing boron was not observed.
^{11}B NMR (96 MHz, Acetone-d_6): δ 10.8.
υ_{max} (solid): 1761, 1539, 1456, 1331, 1290, 1036 cm^{-1}.
HRMS (ESI) *m/z*: $[M+H]^+$ Calcd for $C_{32}H_{32}B_2NaN_4O_8$ 621.2304; Found 621.2286.

Compound 14

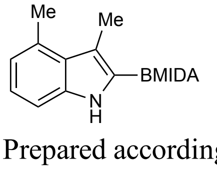

Prepared according to General Procedure A using Pd(dppf)Cl_2 (7.3 mg, 0.01 mmol, 5 mol%), propyne boronic acid MIDA ester (39.0 mg, 0.2 mmol, 1.0 equiv.), **S2** (55.9 mg, 0.24 mmol, 1.2 equiv.), and NaOAc (41.0 mg,

0.5 mmol, 2.5 equiv.). Flash column chromatography (silica gel, 5–30% MeCN in DCM) gave the product as a beige solid (47 mg, 78%).
^{1}H NMR (500 MHz, Acetone-d_6): δ 9.77 (br s, 1H), 7.19 – 7.17 (m, 1H), 6.90 (dd, J = 8.2, 7.0 Hz, 1H), 6.67 – 6.65 (m, 1H), 4.37 (d, J = 17.1 Hz, 2H), 4.14 (d, J = 17.1 Hz, 2H), 2.83 (s, 3H), 2.68 (s, 3H), 2.57 (s, 3H).
^{13}C NMR (126 MHz, Acetone-d_6): δ 169.2, 139.4, 131.4, 129.1, 122.4, 120.8, 119.2, 110.2, 62.9, 48.0, 20.9, 13.1. The carbon bearing boron was not observed.
^{11}B NMR (96 MHz, Acetone-d_6): δ 10.8.
υ_{max} (solid): 3381, 2918, 1757, 1456, 1327, 1211, 1030, 993 cm^{-1}
HRMS (ESI) m/z: [M–H]$^{-}$ Calcd for $C_{15}H_{16}BN_2O_4$ 299.1209; Found 299.1210.

Compound 15

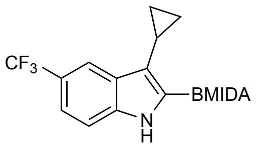

Prepared according to General Procedure A using Pd(dppf)Cl_2 (22.0 mg, 0.03 mmol, 5 mol%), **S3** (133 mg, 0.6 mmol, 1.0 equiv.), 4-amino-3-iodobenzotrifluoride (207 mg, 0.72 mmol, 1.2 equiv.), and NaOAc (123 mg, 1.5 mmol, 2.5 equiv.). Analysis of the reaction mixture by ^{1}H NMR indicated 63% conversion to the desired product. Flash column chromatography (silica gel, 1–5% IPA in DCM) gave the product as a brown solid (90.0 mg, 39%).
^{1}H NMR (500 MHz, Acetone-d_6): δ 10.36 (s, 1H), 7.99 (dd, J = 1.9, 1.0 Hz, 1H), 7.57 (d, J = 8.5 Hz, 1H), 7.35 (dd, J = 8.6, 1.8 Hz, 1H), 4.44 (d, J = 17.1 Hz, 2H), 4.23 (d, J = 17.0 Hz, 2H), 2.91 (s, 3H), 2.00 (tt, J = 8.4, 5.3 Hz, 1H), 0.99 – 0.91 (m, 2H), 0.88 – 0.80 (m, 2H).
^{13}C NMR (126 MHz, Acetone-d_6): δ 169.0, 139.9, 129.3, 126.8 (d, ^{1}J = 270.3 Hz), 124.6, 120.9 (q, ^{2}J = 31.1 Hz), 118.6 (q, ^{3}J = 3.6 Hz), 117.7 (q, ^{3}J = 4.2 Hz), 112.9, 63.1, 48.2, 7.6, 6.7. The carbon bearing boron was not observed.
^{19}F NMR (376 MHz, Acetone-d_6): δ –60.4.
^{11}B NMR (96 MHz, Acetone-d_6): δ 10.4.
υ_{max} (solid): 1767, 1744, 1456, 1331, 1296, 1049, 1034 cm^{-1}.
HRMS (ESI) m/z: [M+Na]$^{+}$ Calcd for $C_{19}H_{16}BF_3NaN_2O_4$ 403.1053; Found 403.1044.

Compound 16

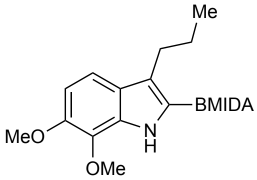

Prepared according to General Procedure A using Pd(dppf)Cl_2 (7.3 mg, 0.01 mmol, 5 mol%), **S4** (44.6 mg, 0.2 mmol, 1.0 equiv.), **S8** (67.0 mg, 0.24 mmol, 1.2 equiv.), and NaOAc (41.0 mg, 0.5 mmol, 2.5 equiv.). Flash column chromatography (silica gel, 5–30% MeCN in DCM) gave the product as a light pink solid (63 mg, 84%).
^{1}H NMR (500 MHz, Acetone-d_6): δ 9.68 (br s, 1H), 7.21 (dd, J = 8.6, 0.7 Hz, 1H), 6.80 (d, J = 8.6 Hz, 1H), 4.37 (d, J = 17.0 Hz, 2H), 4.13 (d, J = 17.1 Hz, 2H), 3.86 (app d, 6H), 2.92 (s, 3H), 2.77 – 2.70 (m, 2H), 1.69 – 1.59 (m, 2H), 0.95 (t, J = 7.3 Hz, 3H).
^{13}C NMR (126 MHz, Acetone-d_6): δ 169.2, 148.1, 135.6, 133.5, 127.4, 124.8, 114.6, 108.8, 63.1, 60.7, 57.9, 48.4, 28.3, 26.2, 14.7. The carbon bearing boron was not observed.
^{11}B NMR (96 MHz, Acetone-d_6): δ 10.7.
υ_{max} (solid): 3389, 2957, 1748, 1450, 1246, 1213, 1028, 1009 cm^{-1}.
HRMS (EI) m/z: [M]$^{+}$ Calcd for ($C_{18}H_{23}BN_2O_6$) 374.1649; Found 374.1643.

Compound 17

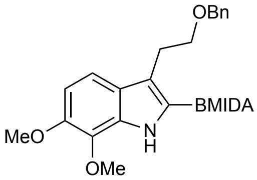

Prepared according to General Procedure A using Pd(dppf)Cl_2 (7.3 mg, 0.01 mmol, 5 mol%), **38** (63.0 mg, 0.2 mmol, 1.0 equiv.), **S8** (63.0 mg, 0.24 mmol, 1.2 equiv.), and NaOAc (41.0 mg, 0.5 mmol, 2.5 equiv.). Flash column chromatography (silica gel, 5–20% MeCN in DCM) gave the product as a light brown solid (71.0 mg, 76%).

^{1}H NMR (500 MHz, Acetone-d_6): δ 9.73 (br s, 1H), 7.33 – 7.23 (m, 5H), 7.22 (d, J = 8.6 Hz, 1H), 6.81 (d, J = 8.6 Hz, 1H), 4.45 (s, 2H), 4.15 (d, J = 17.0 Hz, 2H), 4.02 (d, J = 17.0 Hz, 2H), 3.87 (app d, 6H), 3.77 (t, J = 6.2 Hz, 2H), 3.11 (t, J = 6.2 Hz, 2H), 2.75 (s, 3H).

^{13}C NMR (126 MHz, Acetone-d_6): δ 169.4, 148.2, 139.4, 135.6, 133.5, 129.1, 129.1, 128.4, 127.0, 121.2, 114.5, 108.9, 73.6, 71.9, 63.1, 60.7, 57.9, 48.1, 26.7. The carbon bearing boron was not observed.

^{11}B NMR (96 MHz, Acetone-d_6): δ 10.7.

υ_{max} (solid): 3374, 1771, 1744, 1310, 1217, 1024 cm^{-1}.

HRMS (ESI) m/z: $[M+Na]^+$ Calcd for $C_{26}H_{27}BNaN_2O_7$ 489.1809; Found 489.1802.

Compound 18

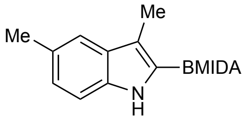

Prepared according to General Procedure A using Pd(dppf)Cl_2 (7.3 mg, 0.01 mmol, 5 mol%), propyne boronic acid MIDA ester (39.0 mg, 0.2 mmol, 1.0 equiv.), 4-amino-3-iodotoluene (55.9 mg, 0.24 mmol, 1.2 equiv.) and NaOAc (41.0 mg, 0.5 mmol, 2.5 equiv.). Flash column chromatography (silica gel, 5–30% MeCN in DCM) gave the product as a beige solid (54 mg, 90%).

^{1}H NMR (500 MHz, Acetone-d_6): δ 9.73 (br s, 1H), 7.30 (dd, J = 1.7, 0.8 Hz, 1H), 7.25 (dd, J = 8.2, 0.7 Hz, 1H), 6.92 (dd, J = 8.4, 1.6 Hz, 1H), 4.37 (d, J = 17.1 Hz, 2H), 4.13 (d, J = 17.0 Hz, 2H), 2.81 (s, 3H), 2.40 (s, 3H), 2.34 (s, 3H).

^{13}C NMR (126 MHz, Acetone-d_6): δ 169.2, 137.4, 131.0, 127.7, 124.1, 118.9, 117.6, 111.7, 62.8, 48.0, 21.6, 9.9. The carbon bearing boron was not observed.

^{11}B NMR (96 MHz, Acetone-d_6): δ 10.7.

υ_{max} (solid): 3414, 2924, 1759, 1456, 1288, 1215, 1030 cm^{-1}.

HRMS (ESI) m/z: $[M+Na]^+$ Calcd for $C_{17}H_{17}BNaN_2O_4$ 323.1179; Found 323.1172.

Compound 19

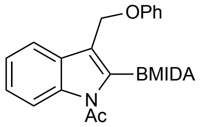

Prepared using General Procedure D using Pd$(OAc)_2$ (4.5 mg, 20 µmol, 10 mol%), **S11** (57.4 mg, 0.2 mmol, 1.0 equiv.), NaOAc (41.0 mg, 0.5 mmol, 2.50 equiv.), LiCl (17.0 mg, 0.4 mmol, 2 equiv.), and **1b** (62.6 mg, 0.24 mmol, 1.2 equiv.). Flash column chromatography (silica gel, 5–20% MeCN in DCM) gave the product as an off-white solid (57 mg, 68%).

^{1}H NMR (500 MHz, DMSO-d_6): δ 7.88 – 7.77 (m, 2H), 7.39 (ddd, J = 8.5, 7.1, 1.4 Hz, 1H), 7.32 – 7.27 (m, 3H), 7.04 – 7.01 (m, 2H), 6.96 – 6.91 (m, 1H), 5.31 (s, 2H), 4.50 (d, J = 17.8 Hz, 1H), 4.37 – 4.22 (m, 2H), 4.03 (d, J = 17.7 Hz, 1H), 3.03 (s, 3H), 2.85 (s, 3H).

^{13}C NMR (126 MHz, DMSO-d_6): δ 172.1, 169.8, 168.6, 158.5, 136.1, 130.2, 129.5, 127.4, 125.0, 122.6, 120.7, 119.3, 114.4, 114.2, 65.3, 64.9, 61.2, 50.0, 27.1. The carbon bearing boron was not observed.

^{11}B NMR (96 MHz, DMSO-d_6): δ 10.9.
υ$_{max}$ (solid): 1776, 1757, 1691, 1315, 1301, 1209, 1041, 873, 754 cm^{-1}.
HRMS (ESI) *m/z*: [M+Na]$^+$ Calcd for $C_{24}H_{21}BNaN_2O_6$ 443.1385; Found 443.1373.

Compound 20

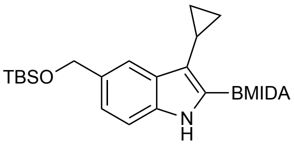

Prepared using General Procedure A using Pd(dppf)Cl_2 (7.3 mg, 10 µmol, 5 mol%), **S3** (44.2 mg, 0.2 mmol, 1.0 equiv.), NaOAc (41.0 mg, 0.5 mmol, 2.50 equiv.) and **S12** (87.2 mg, 0.24 mmol, 1.2 equiv.). Flash column chromatography (silica gel, 5–30% MeCN in DCM) gave the product as a light brown solid (51 mg, 56%).
^{1}H NMR (500 MHz, DMSO-d_6): δ 10.54 (s, 1H), 7.50 (s, 1H), 7.28 (d, *J* = 8.3 Hz, 1H), 6.95 (dd, *J* = 8.4, 1.6 Hz, 1H), 4.73 (s, 2H), 4.37 (d, *J* = 17.3 Hz, 2H), 4.10 (d, *J* = 17.3 Hz, 2H), 2.58 (s, 3H), 1.91 (tt, *J* = 8.5, 5.4 Hz, 1H), 0.91 (s, 9H), 0.82 – 0.79 (m, 2H), 0.76 – 0.73 (m, 2H), 0.07 (s, 6H).
^{13}C NMR (126 MHz, DMSO-d_6): δ 169.2, 136.8, 130.6, 127.7, 121.9, 120.2, 116.8, 111.3, 65.3, 62.0, 47.5, 25.9, 18.1, 7.4, 6.0, −5.1.
^{11}B NMR (96 MHz, DMSO): δ 11.4.
υ$_{max}$ (solid): 2923, 2361, 1771, 1456, 1339, 1292, 1250, 1215, 1032, 835 cm^{-1}.
HRMS (ESI) *m/z*: [M–H]$^-$ Calcd for $C_{23}H_{32}BN_2O_5Si$ 455.2179; Found 455.2186.

Compound 21

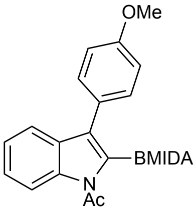

Prepared using General Procedure D using Pd$(OAc)_2$ (4.5 mg, 20 µmol, 10 mol%), **S13** (57.4 mg, 0.2 mmol, 1.0 equiv.), NaOAc (41.0 mg, 0.5 mmol, 2.50 equiv.), LiCl (17.0 mg, 0.4 mmol, 2 equiv.), and **1b** (62.6 mg, 0.24 mmol, 1.2 equiv.). Flash column chromatography (silica gel, 5–20% MeCN in DCM) gave the product as an off-white solid (63 mg, 75%).
^{1}H NMR (500 MHz, DMSO-d_6): δ 7.84 (dd, *J* = 8.6, 0.8 Hz, 1H), 7.38 (ddd, *J* = 8.5, 6.9, 1.5 Hz, 1H), 7.28 – 7.11 (m, 4H), 6.96 (d, *J* = 8.1 Hz, 2H), 4.30 (d, *J* = 17.1 Hz, 2H), 4.15 (s, 2H), 3.78 (s, 3H), 3.15 (s, 3H), 2.85 (s, 3H).
^{13}C NMR (126 MHz, DMSO-d_6): δ 172.3, 170.3, 168.4, 158.6, 144.8, 136.7, 134.6, 132.2, 130.7, 127.8, 125.6, 123.1, 120.5, 114.7, 114.3, 65.3, 55.5, 50.8, 27.6. The carbon bearing boron was not observed.
^{11}B NMR (96 MHz, DMSO-d_6): δ 10.5.
υ$_{max}$ (solid): 1766, 1755, 1689, 1319, 1029, 754 cm^{-1}.
HRMS (ESI) *m/z*: [M+Na]$^+$ Calcd for $C_{24}H_{21}BNaN_2O_6$ 443.1385; Found 443.1372.

Compound 22

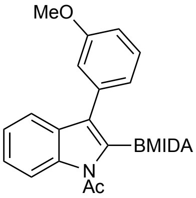

Prepared using General Procedure D using Pd$(OAc)_2$ (4.5 mg, 20 µmol, 10 mol%), **S14** (57.4 mg, 0.2 mmol, 1.0 equiv.), NaOAc (41.0 mg, 0.5 mmol, 2.50 equiv.), LiCl (17.0 mg, 0.4 mmol, 2 equiv.), and **1b** (62.6 mg,

0.24 mmol, 1.2 equiv.). Flash column chromatography (silica gel, 5–20% MeCN in DCM) gave the product as a beige solid (50 mg, 60%).

^{1}H NMR (500 MHz, DMSO-d_6): δ 7.85 (d, J = 8.4 Hz, 1H), 7.40 – 7.37 (m, 1H), 7.30 (br s, 1H), 7.24 – 7.19 (m, 2H), 6.93 – 6.86 (m, 1H), 6.83 – 6.80 (m, 2H), 4.32 (d, J = 17.4 Hz, 2H), 4.24 – 4.03 (m, 2H), 3.77 (s, 3H), 3.20 – 3.07 (s, 3H), 2.86 (s, 3H).

^{13}C NMR (126 MHz, DMSO-d_6): δ 171.9, 169.8, 167.9, 159.0, 136.7, 136.3, 134.2, 131.6, 131.5, 129.4, 125.2, 122.7, 120.1, 114.6, 114.3, 112.7, 64.9, 64.7, 55.0, 50.4, 27.2. The carbon bearing boron was not observed.

^{11}B NMR (96 MHz, DMSO-d_6): δ 12.0.

υ_{max} (solid): 1759, 1685, 1373, 1311, 1251, 1215, 1042, 1001, 746, 704 cm^{-1}.

HRMS (ESI) *m/z*: [M+Na]$^+$ Calcd for $C_{24}H_{21}BNaN_2O_6$ 443.1385; found 443.1366.

Compound 23

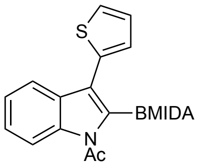

Prepared using General Procedure D using Pd(OAc)$_2$ (4.5 mg, 20 μmol, 10 mol%), **S15** (52.6 mg, 0.2 mmol, 1.0 equiv.), NaOAc (41.0 mg, 0.5 mmol, 2.50 equiv.), LiCl (17.0 mg, 0.4 mmol, 2 equiv.), and **1b** (62.6 mg, 0.24 mmol, 1.2 equiv.). Flash column chromatography (silica gel, 10–15% MeCN in DCM) afforded a brown film, with subsequent trituration with Et$_2$O giving the product as a brown solid (35 mg, 44%).

^{1}H NMR (500 MHz, DMSO-d_6): δ 7.85 (d, J = 8.5 Hz, 1H), 7.57 (dd, J = 5.1, 1.2 Hz, 1H), 7.42 – 7.36 (m, 2H), 7.26 (t, J = 7.5 Hz, 1H), 7.13 – 7.07 (m, 2H), 4.37 (d, J = 17.6 Hz, 2H), 4.22 (d, J = 17.7 Hz, 2H), 3.16 (s, 3H), 2.86 (s, 3H).

^{13}C NMR (126 MHz, DMSO-d_6): δ 172.2, 169.6, 168.3, 136.2, 135.4, 131.3, 127.6, 127.1, 126.3, 126.2, 125.5, 122.9, 119.9, 114.2, 65.1, 64.8, 50.5, 27.1. The carbon bearing boron was not observed.

^{11}B NMR (96 MHz, DMSO-d_6): δ 10.4.

υ_{max} (film): 1716, 1683, 1298, 1034, 1004, 750 cm^{-1}.

HRMS (ESI) *m/z*: [M+Na]$^+$ Calcd for $C_{21}H_{17}BNaN_2O_5S$ 419.0843; Found 419.0836.

Compound 24

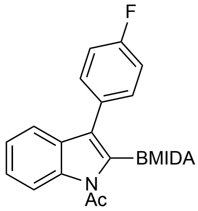

Prepared using General Procedure D using Pd(OAc)$_2$ (4.5 mg, 20 μmol, 10 mol%), **S16** (55.0 mg, 0.2 mmol, 1.0 equiv.), NaOAc (41.0 mg, 0.5 mmol, 2.50 equiv.), LiCl (17.0 mg, 0.4 mmol, 2 equiv.), and **1b** (62.6 mg, 0.24 mmol, 1.2 equiv.). Flash column chromatography (silica gel, 5–20% MeCN in DCM) gave the product as a brown solid (28 mg, 34%).

^{1}H NMR (500 MHz, DMSO-d_6): δ 7.86 (d, J = 8.6 Hz, 1H), 7.40 (ddd, J = 8.5, 7.0, 1.4 Hz, 1H), 7.32 – 7.27 (m, 2H), 7.25 – 7.21 (m, 2H), 7.20 – 7.11 (m, 2H), 4.31 (d, J = 17.4 Hz, 2H), 4.25 – 4.08 (m, 2H), 3.14 (s, 3H), 2.86 (s, 3H).

^{13}C NMR (126 MHz, DMSO-d_6): δ 171.8, 169.8, 168.0, 161.3 (d, ^1J = 242.8 Hz), 136.2, 133.3, 131.7 (d, ^3J = 3.1 Hz), 131.5, 131.1, 125.3, 122.8, 119.9, 115.2 (d, ^2J = 21.2 Hz), 114.4, 65.2, 64.6, 50.4, 27.1. The carbon bearing boron was not observed.

^{19}F NMR (471 MHz, DMSO-d_6): δ –115.9.

^{11}B NMR (96 MHz, DMSO-d_6): δ 11.1.

υ_{max} (solid): 1745, 1601, 1333, 1199, 1041, 904, 879 cm^{-1}.

HRMS (ESI) *m/z*: [M+Na]$^+$ Calcd for $C_{23}H_{18}BFNaN_2O_5$ 431.1185; Found 431.1178.

Compound 25

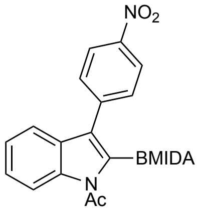

Prepared using General Procedure D using $Pd(OAc)_2$ (4.5 mg, 20 µmol, 10 mol%), **S17** (60.4 mg, 0.2 mmol, 1.0 equiv.), NaOAc (41.0 mg, 0.5 mmol, 2.50 equiv.), LiCl (17.0 mg, 0.4 mmol, 2 equiv.), and **1b** (62.6 mg, 0.24 mmol, 1.2 equiv.). Flash column chromatography (silica gel, 5–20% MeCN in DCM) gave the product as an off-white solid (38 mg, 44%).

^{1}H NMR (500 MHz, DMSO-d_6): δ 8.38 – 8.13 (m, 2H), 7.90 (d, J = 8.5 Hz, 1H), 7.57 (d, J = 8.3 Hz, 2H), 7.45 – 7.42 (m, 1H), 7.28 – 7.25 (m, 1H), 7.18 (d, J = 7.9 Hz, 1H), 4.40 – 4.29 (m, 2H), 4.26 – 4.20 (m, 2H), 3.18 (s, 3H), 2.89 (s, 3H).

^{13}C NMR (126 MHz, DMSO-d_6): δ 171.9, 169.7, 168.1, 146.3, 143.5, 136.3, 132.2, 130.7, 130.6, 125.5, 123.5, 123.0, 119.7, 114.6, 65.4, 64.7, 50.4, 27.1. The carbon bearing boron was not observed.

^{11}B NMR (96 MHz, DMSO-d_6): δ 11.4.

υ_{max} (film): 1759, 1685, 1514, 1350, 1311, 1037, 750 cm^{-1}.

HRMS (ESI) *m/z*: $[M+Na]^+$ Calcd for $C_{23}H_{18}BNaN_3O_7$ 458.1130; Found 458.1117.

Compound 26

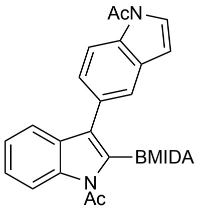

Prepared using General Procedure D using $Pd(OAc)_2$ (4.5 mg, 20 µmol, 10 mol%), **S18** (67.6 mg, 0.2 mmol, 1.0 equiv.), NaOAc (41.0 mg, 0.5 mmol, 2.50 equiv.), LiCl (17.0 mg, 0.4 mmol, 2 equiv.), and **1b** (62.6 mg, 0.24 mmol, 1.2 equiv.). Flash column chromatography (silica gel, 10–30% MeCN in DCM) gave the product as a colourless solid (42 mg, 52%).

^{1}H NMR (500 MHz, DMSO-d_6): δ 8.35 (d, J = 8.4 Hz, 1H), 7.90 – 7.83 (m, 2H), 7.52 (br s, 1H), 7.39 (ddd, J = 8.5, 6.8, 1.6 Hz, 1H), 7.25 – 7.14 (m, 3H), 6.78 (br s, 1H), 4.34 – 4.10 (m, 4H), 3.20 (s, 3H), 2.87 (s, 3H), 2.68 (s, 3H).

^{13}C NMR (126 MHz, DMSO-d_6): δ 171.9, 169.5, 136.3, 134.5, 134.0, 131.8, 130.5, 130.4, 127.4, 126.0, 125.2, 122.7, 121.3, 120.1, 115.8, 114.3, 108.6, 65.2, 64.6, 50.5, 27.2, 23.8. The carbon bearing boron was not observed. Due to poor solubility the BMIDA carbonyl signals were not visible.

^{11}B NMR (96 MHz, DMSO-d_6): δ 10.4.

υ_{max} (solid): 1761, 1693, 1460, 1369, 1325, 1199, 933, 873, 744, 725, 611 cm^{-1}.

HRMS (ESI) *m/z*: $[M+Na]^+$ Calcd for $C_{27}H_{22}BNaN_3O_6$ 494.1494; Found 494.1482.

Compound 27

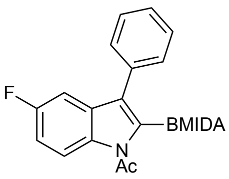

Prepared according to General Procedure D using $Pd(OAc)_2$ (4.5 mg, 20 µmol, 10 mol%), **2b** (51.4 mg, 0.2 mmol, 1.0 equiv.), **S19** (67.0 mg, 0.24 mmol, 1.2 equiv.), NaOAc (41.0 mg, 0.5 mmol, 2.5 equiv.), and LiCl

(17.0 mg, 0.4 mmol, 2.0 equiv.). Flash column chromatography (silica gel, 6–30% MeCN in DCM) gave the product as a brown solid (26 mg, 32%).
^{1}H NMR (500 MHz, DMSO-d_6): δ 7.88 (dd, J = 9.2, 4.1 Hz, 1H), 7.43 – 7.35 (m, 2H), 7.34 – 7.32 (m, 1H), 7.27 – 7.22 (m, 3H), 6.82 (dd, J = 8.8, 2.7 Hz, 1H), 4.31 (d, J = 17.4 Hz, 2H), 4.26 – 4.05 (m, 2H), 3.16 (s, 3H), 2.86 (s, 3H).
^{13}C NMR (126 MHz, DMSO-d_6): δ 171.7, 169.7, 167.8, 158.5 (d, ^{1}J = 238.3 Hz), 134.8, 133.9 (d, ^{4}J = 3.8 Hz), 132.8, 132.6 (d, ^{3}J = 9.1 Hz), 129.0, 128.4, 127.1, 115.9 (d, ^{3}J = 9.2 Hz), 112.8 (d, ^{2}J = 25.1 Hz), 104.8 (d, ^{2}J = 23.4 Hz), 65.0, 64.6, 50.4, 27.0. The carbon bearing boron was not observed.
^{19}F NMR (376 MHz, DMSO-d_6): δ –120.9.
^{11}B NMR (96 MHz, DMSO-d_6): δ 9.1.
υ_{max} (solid): 1749, 1709, 1269, 1304, 1121, 1043 cm^{-1}.
HRMS (ESI) *m/z*: $[M+Na]^+$ Calcd for $C_{23}H_{18}BFNaN_2O_5$ 431.1191; Found 431.1176.

Compound 28

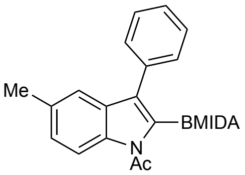

Prepared according to General Procedure D using $Pd(OAc)_2$ (4.5 mg, 20 µmol, 10 mol%), **2b** (51.4 mg, 0.2 mmol, 1.0 equiv.), **S20** (66.0 mg, 0.24 mmol, 1.2 equiv.), NaOAc (41.0 mg, 0.5 mmol, 2.5 equiv.) and LiCl (17.0 mg, 0.4 mmol, 2.0 equiv.). Flash column chromatography (silica gel, 8–30% MeCN in DCM) gave the product as a light brown solid (41 mg, 51%).
^{1}H NMR (500 MHz, DMSO-d_6): δ 7.73 (d, J = 8.6 Hz, 1H), 7.42 – 7.39 (m, 2H), 7.34 – 7.30 (m, 1H), 7.25 – 7.23 (m, 2H), 7.20 (dd, J = 8.8, 1.8 Hz, 1H), 6.94 – 6.90 (m, 1H), 4.29 (d, J = 17.2 Hz, 2H), 4.21 – 4.04 (m, 2H), 3.14 (s, 3H), 2.83 (s, 3H), 2.32 (s, 3H).
^{13}C NMR (126 MHz, DMSO-d_6): δ 171.5, 169.8, 167.8, 135.4, 134.6, 134.3, 131.9, 131.7, 129.1, 128.3, 126.9, 126.5, 119.7, 114.1, 65.1, 64.6, 50.4, 27.1, 20.7. The carbon bearing boron was not observed.
^{11}B NMR (96 MHz, DMSO-d_6): δ 9.5.
υ_{max} (solid): 1746, 1697, 1449, 1310, 1038 cm^{-1}.
HRMS (ESI) *m/z*: $[M+Na]^+$ Calcd for $C_{24}H_{22}BNaN_2O_5$ 427.1441; Found 427.1432.

Compound 29

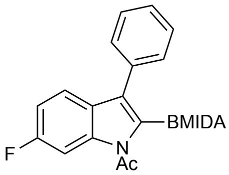

Prepared according to General Procedure D using $Pd(OAc)_2$ (4.5 mg, 20 µmol, 10 mol%), **2b** (51.4 mg, 0.2 mmol, 1.0 equiv.), **S21** (67.0 mg, 0.24 mmol, 1.2 equiv.), NaOAc (41.0 mg, 0.5 mmol, 2.5 equiv.), and LiCl (17.0 mg, 0.4 mmol, 2.0 equiv.). Flash column chromatography (silica gel, 6–30% MeCN in DCM) gave the title product as a dark brown solid (48 mg, 59%).
^{1}H NMR (500 MHz, DMSO-d_6): δ 7.70 (dd, J = 11.1, 2.1 Hz, 1H), 7.45 – 7.36 (m, 2H), 7.35 – 7.31 (m, 1H), 7.26 (d, J = 7.4 Hz, 2H), 7.15 – 7.08 (m, 2H), 4.30 (d, J = 17.3 Hz, 2H), 4.25 – 4.02 (m, 2H), 3.15 (s, 3H), 2.86 (s, 3H).
^{13}C NMR (126 MHz, DMSO-d_6): δ 171.9, 169.8, 167.8, 160.6 (d, ^{1}J = 238.8 Hz), 136.3 (d, ^{3}J = 12.1 Hz), 134.9, 133.9, 129.1, 128.4, 128.1, 127.1, 121.0 (d, ^{3}J = 10.3 Hz), 110.8 (d, ^{2}J = 24.3 Hz), 101.8 (d, ^{2}J = 28.6 Hz), 65.0, 64.7, 50.4, 26.9. The carbon bearing boron was not observed.
^{19}F NMR (376 MHz, DMSO-d_6): δ –116.3.
^{11}B NMR (96 MHz, DMSO-d_6): δ 10.7.

υ_{max} (solid): 1769, 1715, 1479, 1314, 1152, 1061 cm^{-1}.
HRMS (ESI) *m/z*: $[M+Na]^+$ Calcd for $C_{23}H_{18}BFNaN_2O_5$ 431.1191; Found 431.1174.

Compound 30

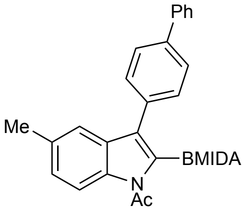

Prepared according to General Procedure D using $Pd(OAc)_2$ (4.5 mg, 20 μmol, 10 mol%), **S1** (66.6 mg, 0.2 mmol, 1.0 equiv.), **S20** (66.0 mg, 0.24 mmol, 1.2 equiv.), NaOAc (41.0 mg, 0.5 mmol, 2.5 equiv.) and LiCl (17.0 mg, 0.4 mmol, 2.0 equiv.). Flash column chromatography (silica gel, 6–30% MeCN in DCM) gave the title product as a light brown solid (45 mg, 47%).
^{1}H NMR (500 MHz, DMSO-d_6): δ 7.75 (d, *J* = 8.6 Hz, 1H), 7.74 – 7.66 (m, 4H), 7.52 – 7.49 (m, 2H), 7.42 – 7.30 (m, 3H), 7.22 (dd, *J* = 8.7, 1.8 Hz, 1H), 7.04 – 7.00 (m, 1H), 4.31 (d, *J* = 17.4 Hz, 2H), 4.27 – 4.11 (m, 2H), 3.18 (s, 3H), 2.85 (s, 3H), 2.34 (s, 3H).
^{13}C NMR (126 MHz, DMSO-d_6): δ 171.6, 169.8, 167.9, 140.0, 138.4, 134.7, 134.7, 133.9, 131.8, 131.7, 129.7, 129.0, 127.4, 126.5, 126.5, 119.7, 114.2, 65.2, 64.7, 50.5, 27.1, 20.7. The carbon bearing boron was not observed.
^{11}B NMR (96 MHz, DMSO-d_6): δ 11.2.
υ_{max} (solid): 1749, 1709, 1269, 1304, 1121, 1043 cm^{-1}.
HRMS (ESI) *m/z*: $[M+Na]^+$ Calcd for $C_{30}H_{25}BNaN_2O_5$ 503.1754; Found 503.1744.

Compound 31

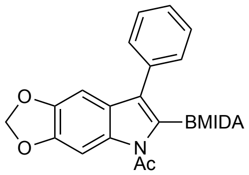

Prepared according to General Procedure D using $Pd(OAc)_2$ (4.5 mg, 20 μmol, 10 mol%), **2b** (51.4 mg, 0.2 mmol, 1.0 equiv.), **S23** (73.2 mg, 0.24 mmol, 1.2 equiv.), NaOAc (41.0 mg, 0.5 mmol, 2.5 equiv.) and LiCl (17.0 mg, 0.4 mmol, 2.0 equiv.). Flash column chromatography (silica gel, 8–30% MeCN in DCM) gave the title product as a light brown solid (48 mg, 55%).
^{1}H NMR (500 MHz, DMSO-d_6): δ 7.45 (s, 1H), 7.43 – 7.34 (m, 2H), 7.33 – 7.28 (m, 1H), 7.23 – 7.22 (m, 2H), 6.48 (s, 1H), 6.05 (s, 2H), 4.27 (d, *J* = 17.3 Hz, 2H), 4.17 – 4.05 (m, 2H), 3.14 (s, 3H), 2.80 (s, 3H).
^{13}C NMR (126 MHz, DMSO-d_6): δ 171.9, 169.7, 167.8, 146.8, 144.2, 135.4, 134.3, 131.1, 129.0, 128.3, 126.9, 125.7, 101.4, 98.1, 96.1, 65.0, 64.6, 50.4, 26.9. The carbon bearing boron was not observed. Due to poor solubility the BMIDA carbonyl signals were weak – these were confirmed by 2D NMR.
^{11}B NMR (96 MHz, DMSO-d_6): δ 11.2.
υ_{max} (solid): 2363, 1773, 1749, 1707, 1476, 1348, 1310, 1169, 1024 cm^{-1}.
HRMS (ESI) *m/z*: $[M+Na]^+$ Calcd for $C_{24}H_{19}BNaN_2O_7$ 457.1183; Found 457.1178.

Compound 32

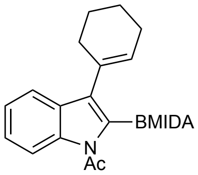

Prepared using General Procedure D using $Pd(OAc)_2$ (4.5 mg, 20 μmol, 10 mol%), **S25** (52.2 mg, 0.2 mmol, 1.0 equiv.), NaOAc (41.0 mg, 0.5 mmol, 2.50 equiv.), LiCl (17.0 mg, 0.4 mmol, 2 equiv.), and **1b** (62.6 mg,

0.24 mmol, 1.2 equiv.). Flash column chromatography (silica gel, 5–20% MeCN in DCM) gave the title product as a brown solid (58 mg, 74%).

^{1}H NMR (500 MHz, DMSO-d_6): δ 7.77 (d, J = 8.4 Hz, 1H), 7.39 (d, J = 7.7 Hz, 1H), 7.34 (ddd, J = 8.5, 7.1, 1.4 Hz, 1H), 7.25 – 7.22 (m, 1H), 5.57 – 5.51 (m, 1H), 4.43 – 4.35 (m, 2H), 4.19 (d, J = 17.3 Hz, 1H), 4.09 (d, J = 17.7 Hz, 1H), 3.00 (s, 3H), 2.79 (s, 3H), 2.23 (br s, 2H), 2.06 (br s, 2H), 1.83 (br s, 1H), 1.69 (br s, 2H), 1.64 – 1.62 (m, 1H).

^{13}C NMR (126 MHz, DMSO-d_6): δ 171.2, 169.9, 168.6, 136.7, 136.3, 133.9, 130.8, 125.3, 124.8, 122.4, 119.9, 114.3, 64.8, 64.5, 50.5, 29.6, 27.0, 25.1, 22.1, 21.3. The carbon bearing boron was not observed.

^{11}B NMR (96 MHz, DMSO-d_6): δ 11.3.

υ_{max} (solid): 1755, 1695, 1309, 1035, 1010, 744 cm^{-1}.

HRMS (ESI) *m/z*: $[M+Na]^+$ Calcd for $C_{23}H_{23}BNaN_2O_5$ 417.1592; Found 417.1582.

Compound 33

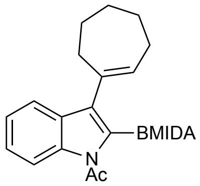

Prepared using General Procedure D using $Pd(OAc)_2$ (4.5 mg, 20 µmol, 10 mol%), **S26** (55.0 mg, 0.2 mmol, 1.0 equiv.), NaOAc (41.0 mg, 0.5 mmol, 2.50 equiv.), LiCl (17.0 mg, 0.4 mmol, 2 equiv.), and **1b** (62.6 mg, 0.24 mmol, 1.2 equiv.). Flash column chromatography (silica gel, 5–20% MeCN in DCM) gave the title product as an off-white solid (45 mg, 55%).

^{1}H NMR (500 MHz, DMSO-d_6): δ 7.77 (d, J = 8.5 Hz, 1H), 7.44 (br d, J = 7.7 Hz, 1H), 7.34 (ddd, J = 8.5, 7.2, 1.4 Hz, 1H), 7.28 – 7.23 (m, 1H), 5.66 (t, J = 6.1 Hz, 1H), 4.44 – 4.33 (m, 2H), 4.19 (d, J = 17.3 Hz, 1H), 4.05 (br d, J = 17.7 Hz, 1H), 2.99 (s, 3H), 2.79 (s, 3H), 2.64 – 2.53 (m, 1H), 2.30 – 2.21 (m, 1H), 2.21 – 2.12 (m, 2H), 1.95 – 1.79 (m, 2H), 1.77 – 1.60 (m, 3H), 1.52 – 1.41 (m, 1H).

^{13}C NMR (126 MHz, DMSO-d_6): δ 171.3, 169.9, 168.7, 139.9, 138.8, 136.3, 130.8, 130.4, 124.9, 122.5, 120.2, 114.3, 64.8, 64.6, 50.4, 34.7, 31.6, 28.5, 27.0, 26.6, 26.5. The carbon bearing boron was not observed.

^{11}B NMR (96 MHz, DMSO-d_6): δ 12.4.

υ_{max} (solid): 1755, 1695, 1446, 1307, 1201, 1018, 877, 744 cm^{-1}.

HRMS (ESI) *m/z*: $[M+Na]^+$ Calcd for $C_{24}H_{25}BNaN_2O_5$ 431.1749; Found 431.1736.

Compound 34

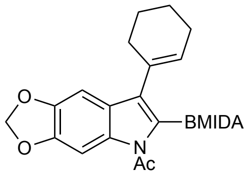

Prepared using General Procedure D using $Pd(OAc)_2$ (4.5 mg, 20 µmol, 10 mol%), **S25** (52.2 mg, 0.2 mmol, 1.0 equiv.), **S23** (73.2 mg, 0.24 mmol, 1.2 equiv.), NaOAc (41.0 mg, 0.5 mmol, 2.5 equiv.), and LiCl (17.0 mg, 0.4 mmol, 2.0 equiv.). Flash column chromatography (silica gel, 8–30% MeCN in DCM) gave the title product as a light brown solid (61 mg, 70%).

^{1}H NMR (500 MHz, DMSO-d_6): δ 7.36 (s, 1H), 6.78 (s, 1H), 6.05 (d, J = 5.5 Hz, 2H), 5.53 – 5.46 (m, 1H), 4.40 – 4.32 (m, 2H), 4.16 (d, J = 17.3 Hz, 1H), 4.12 – 3.97 (m, 1H), 2.97 (s, 3H), 2.73 (s, 3H), 2.18 (br s, 2H), 2.03 (br s, 2H), 1.80 (br s, 1H), 1.73 – 1.56 (m, 3H).

^{13}C NMR (126 MHz, DMSO-d_6): δ 171.1, 169.8, 168.6, 146.4, 143.9, 136.7, 134.0, 131.0, 125.3, 124.9, 101.3, 98.2, 96.1, 64.7, 64.4, 50.5, 29.4, 26.7, 25.1, 22.1, 21.3. The carbon bearing boron was not observed.

^{11}B NMR (96 MHz, DMSO-d_6): δ 10.6.

υ_{max} (solid): 1759, 1686, 1337, 1300, 1173, 1032 cm^{-1}.

HRMS (ESI) *m/z*: $[M+H]^+$ Calcd for $C_{22}H_{24}BN_2O_7$ 439.1677; Found 439.1664.

Compound 35

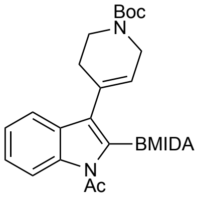

Prepared according to General Procedure D using $Pd(OAc)_2$ (4.5 mg, 20 µmol, 10 mol%), **S27** (72.4 mg, 0.2 mmol, 1.0 equiv.), **1b** (62.7 mg, 0.24 mmol, 1.2 equiv.), NaOAc (41.0 mg, 0.5 mmol, 2.5 equiv.) and LiCl (17.0 mg, 0.4 mmol, 2.0 equiv.). Flash column chromatography (silica gel, 6–30% MeCN in DCM) gave the product as a brown solid (82 mg, 83%).

^{1}H NMR (500 MHz, DMSO-d_6): δ 7.82 – 7.77 (m, 1H), 7.40 – 7.34 (m, 2H), 7.26 – 7.22 (m, 1H), 5.59 (br s, 1H), 4.44 (d, *J* = 17.8 Hz, 1H), 4.37 (d, *J* = 17.3 Hz, 1H), 4.22 – 4.18 (m, 2H), 4.07 – 3.95 (m, 1H), 3.80 – 3.65 (m, 2H), 3.60 – 3.51 (m, 1H), 3.01 (s, 3H), 2.81 (s, 3H), 2.47 – 2.34 (m, 1H), 2.23 (br d, *J* = 16.4 Hz, 1H), 1.46 (s, 9H).

^{13}C NMR (126 MHz, DMSO-d_6): δ 171.3, 169.9, 169.0, 154.2, 136.3, 135.1, 132.2, 130.4, 125.0, 122.6, 119.8, 114.4, 78.6, 65.0, 64.6, 50.5, 43.6, 43.0, 39.8, 29.7, 28.2, 27.0. The carbon bearing boron was not observed.

^{11}B NMR (96 MHz, DMSO-d_6): δ 11.1.

υ_{max} (solid): 1763, 1695, 1676, 1283, 1026 cm^{-1}.

HRMS (ESI) *m/z*: $[M+H]^+$ Calcd for $C_{25}H_{31}BN_3O_7$ 496.2255; Found 496.2238.

Compound 37

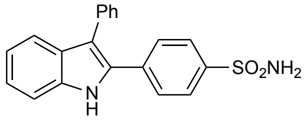

To a flame dried 10 mL microwave vial was added **4** (45 mg, 0.115 mmol, 1 equiv.), 4-bromobenzenesulfonamide (27.2 mg, 0.115 mmol, 1 equiv.), Cs_2CO_3 (113 mg, 0.346 mmol, 3 equiv.), and $Pd(PPh_3)_2Cl_2$ (4.05 mg, 5.7 µmol, 5 mol%). The vial was capped and purged with N_2 before adding THF (1.15 mL, 0.1 M) and H_2O (31 µL, 1.73 mmol, 15 equiv.). The reaction mixture was then heated to 65 ºC for 24 h. The reaction mixture was allowed to cool to room temperature and diluted with EtOAc (10 mL), washed with H_2O (10 mL), and brine (10 mL), before being dried over Na_2SO_4 and concentrated. The crude mixture was taken up in THF/H_2O (4/1, 1 mL, 0.1 M), LiOH pellets (6.9 mg, 0.288 mmol, 2.5 equiv.) were added, and the mixture was stirred at room temperature for 5 h. The reaction mixture was diluted with EtOAc (10 mL), washed with H_2O (10 mL), and brine (10 mL), before being dried over Na_2SO_4 and concentrated to a residue that was purified by flash column chromatography (silica gel, 20–40% EtOAc in hexanes) to afford the product as a white solid (25 mg, 62%).

^{1}H NMR (400 MHz, DMSO-d_6): δ 11.72 (s, 1H), 7.80 – 7.75 (m, 2H), 7.61 – 7.56 (m, 2H), 7.51 – 7.46 (m, 2H), 7.46 – 7.41 (m, 2H), 7.39 (br s, 2H), 7.36 – 7.31 (m, 3H), 7.21 (ddd, *J* = 8.3, 7.0, 1.1 Hz, 1H), 7.07 (ddd, *J* = 8.0, 7.0, 1.0 Hz, 1H).

^{13}C NMR (126 MHz, DMSO-d_6): δ 142.6, 136.4, 135.8, 134.8, 132.5, 129.9, 128.9, 128.4, 127.9, 126.5, 125.9, 122.8, 120.1, 118.9, 114.8, 111.7.

υ_{max} (solid): 3286, 3147, 1598, 1325, 1163, 1020, 740 cm^{-1}.

HRMS (ESI) *m/z*: $[M–H]^-$ Calcd for $C_{20}H_{15}N_2O_2S$ 347.0860; Found 347.0860.

Compound 38

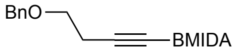

Prepared according to General Procedure C using **S10** (6.63 g, 41.4 mmol, 1.0 equiv.), THF (124 mL), EtMgBr (22.6 mL, 49.7 mmol, 2.2 M in Et_2O, 1.2 equiv.), $B(OMe)_3$ (9.23 mL, 82.8 mmol, 2.0 equiv.), THF (100 mL), and *N*-methyliminodiacetic acid (12.2 g, 82.8 mmol, 2.0 equiv.). Flash column chromatography (silica gel, 0–

25% MeCN in DCM) gave an off-white solid which was triturated from EtOAc and hexane to give the product as a fluffy white solid (7.80 g, 60%).
^{1}H NMR (500 MHz, $CDCl_3$): δ 7.37 – 7.26 (m, 5H), 4.52 (s, 2H), 3.85 (d, *J* = 16.5 Hz, 2H), 3.68 (d, *J* = 16.5 Hz, 2H), 3.61 (t, *J* = 6.7 Hz, 2H), 2.96 (s, 3H), 2.55 (t, *J* = 6.7 Hz, 2H).
^{13}C NMR (126 MHz, $CDCl_3$): δ 167.1, 138.1, 128.6, 128.0, 127.9, 100.7, 73.1, 68.2, 61.5, 47.7, 21.0. The carbon bearing boron was not observed.
^{11}B NMR (96 MHz, DMSO-d_6): δ 6.2.
υ_{max} (solid): 3017, 2866, 2208, 1763, 1462, 1329, 1275, 1167 cm^{-1}.
HRMS (ESI) *m/z*: $[M+Na]^+$ Calcd for $C_{16}H_{18}BNNaO_5$ 338.1176; Found 338.1164.

Compound 39

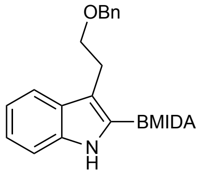

Prepared using General Procedure A using $Pd(dppf)Cl_2$ (707 mg, 966 µmol, 5 mol%), **38** (6.09 g, 19.3 mmol, 1.0 equiv.), NaOAc (3.96 g, 48.3 mmol, 2.5 equiv.) and 2-iodoaniline (5.08 g, 23.2 mmol, 1.2 equiv.). Flash column chromatography (silica gel, 6–30% MeCN in DCM) gave the product as a brown solid (7.08 g, 90%).
^{1}H NMR (500 MHz, DMSO-d_6): δ 10.63 (s, 1H), 7.49 (d, *J* = 7.9 Hz, 1H), 7.36 – 7.23 (m, 6H), 7.07 – 7.04 (m, 1H), 6.95 – 6.92 (m, 1H), 4.44 (s, 2H), 4.29 (d, *J* = 17.2 Hz, 2H), 3.97 (d, *J* = 17.3 Hz, 2H), 3.63 (t, *J* = 6.7 Hz, 2H), 3.05 (t, *J* = 6.7 Hz, 2H), 2.50 (s, 3H).
^{13}C NMR (126 MHz, DMSO-d_6): δ 169.2, 138.4, 137.8, 128.4, 128.2, 127.8, 127.5, 121.3, 118.5, 118.4, 118.0, 111.3, 71.9, 70.9, 61.9, 47.5, 25.4. The carbon bearing boron was not observed.
^{11}B NMR (96 MHz, DMSO-d_6): δ 10.6.
υ_{max} (solid): 3352, 1769, 1746, 1234, 1221, 1038, 997 cm^{-1}.
HRMS (ESI) *m/z*: $[M+Na]^+$ Calcd for $C_{24}H_{23}BNaN_2O_5$ 429.1598; Found 429.1590.

Compound 40

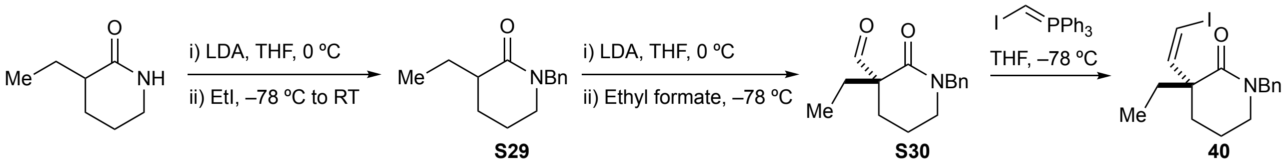

a) Compound S29. A flame-dried round-bottom flask was charged with *i*-Pr_2NH (9.95 mL, 71.0 mmol, 1.30 equiv.) and THF (250 mL, 0.28 M) and cooled to 0 °C. To the stirred solution was added *n*-BuLi (2.43 M in hexanes, 27.0 mL, 65.6 mmol, 1.20 equiv.) *via* syringe, and the solution was stirred at 0 °C for 30 min. A solution of 1-benzyl-2-piperidinone (10.3 g, 54.6 mmol, 1.0 equiv.) in THF (100 mL, 0.54 M) was added, resulting in a bright yellow coloured solution. This was stirred at 0 °C for 10 min before being allowed to warm to room temperature where it was stirred for a further 30 min. The mixture was then cooled to –78 °C. Ethyl iodide (5.71 mL, 71.0 mmol, 1.30 equiv.) was added *via* syringe over several minutes and the reaction mixture was stirred at this temperature for a further 30 min before being allowed to warm to room temperature and stir for 1 h. Sat. aq. NH_4Cl (100 mL) was added, and the layers were separated. The aqueous layer was extracted with EtOAc (2 x 100 mL). The combined organics were dried over Na_2SO_4, filtered, and concentrated to a residue that was purified by flash column chromatography (10–30% EtOAc in hexane) to give the product as a colourless oil (11.56 g, 97%).
^{1}H NMR (500 MHz, $CDCl_3$): δ 7.34 – 7.31 (m, 2H), 7.29 – 7.22 (m, 3H), 4.63 – 4.57 (m, 2H), 3.20 (dd, *J* = 7.3, 4.9 Hz, 2H), 2.35 – 2.29 (m, 1H), 2.05 – 1.97 (m, 1H), 1.96 – 1.91 (m, 1H), 1.88 – 1.82 (m, 1H), 1.74 – 1.67 (m, 1H), 1.66 – 1.53 (m, 2H), 0.98 (t, *J* = 7.5 Hz, 3H).
^{13}C NMR (126 MHz, $CDCl_3$): δ 172.8, 137.6, 128.6, 128.0, 127.3, 50.3, 47.5, 43.0, 25.9, 25.0, 21.7, 11.6. Spectral data in agreement with literature values.[15]

b) Compound S30 A flame-dried round-bottom flask was charged with *i*-Pr_2NH (2.80 mL, 12 mmol, 1.20 equiv.) and THF (30 mL, 0.04 M), and cooled to 0 °C. To the stirred solution was added *n*-BuLi (2.3 M in hexanes, 5.17 mL, 11.9 mmol, 1.19 equiv.) *via* syringe, and the solution was stirred at 0 °C for 30 min. A solution of **S29** (2.17 g, 10.0 mmol, 1.0 equiv.) in THF (20 mL, 0.5 M) was then added *via* cannula. The mixture was stirred for 1 h at 0 °C before being cooled to –78 °C. A solution of ethyl formate (1.05 mL, 13 mmol, 1.3 equiv.) in THF (10 mL) was then added *via* syringe then the reaction mixture was allowed to warm to room temperature and stir overnight. The mixture was quenched with sat. aq. NH_4Cl (20 mL) and extracted with EtOAc (2 x 50 mL). The combined organics were dried over Na_2SO_4, filtered, and concentrated to a residue that was purified by flash column chromatography (silica gel, 10–20% ethyl acetate in hexane) to give the product as a colourless oil (1.50 g, 61%).

^{1}H NMR (500 MHz, $CDCl_3$): δ 9.72 (s, 1H), 7.34 – 7.31 (m, 2H), 7.29 – 7.25 (m, 1H), 7.24 – 7.19 (m, 2H), 4.67 (d, *J* = 14.6 Hz, 1H), 4.55 (d, *J* = 14.6 Hz, 1H), 3.24 – 3.12 (m, 2H), 2.30 – 2.22 (m, 1H), 2.10 – 2.02 (m, 1H), 1.90 – 1.79 (m, 1H), 1.79 – 1.75 (m, 1H), 1.69 – 1.57 (m, 2H), 0.89 (t, *J* = 7.5 Hz, 3H).

^{13}C NMR (126 MHz, $CDCl_3$): δ 202.1, 169.0, 137.0, 128.8, 128.1, 127.6, 59.5, 50.8, 47.7, 27.9, 24.5, 20.4, 8.5.

υ_{max} (solid): 2967, 2940, 1724, 1628, 1489, 1452, 1352, 1265, 1200, 1169 cm^{-1}.

HRMS (ESI) *m/z*: $[M+H]^+$ Calcd for $C_{15}H_{20}NO_2$ 246.1494; Found 246.1484.

c) Compound 40. A slurry of iodomethylphosphonium iodide (6.76 g, 12.8 mmol, 1.40 equiv.) in THF (57 mL, 0.22 M) was cooled to 0 °C prior to addition of NaHMDS (0.85 M in THF, 13.9 mL, 11.8 mmol, 1.30 equiv.) dropwise *via* syringe. The deep yellow suspension was stirred at 0 °C for 10 min before being cooled to –78 °C and stirred for a further 30 min. A solution of **S30** (2.23 g, 9.11 mmol, 1.00 equiv.) was added as a solution in THF (3.5 mL) dropwise *via* syringe. The reaction mixture was stirred at –78 °C for 3 h before being quenched with sat. aq. NH_4Cl (50 mL) then allowed to warm to room temperature. The mixture was then extracted with EtOAc (3 x 100 mL). The combined organics were dried over Na_2SO_4, filtered, and concentrated prior to purification by flash column chromatography (silica gel, 5–30% EtOAc in hexane) to give the product as a light, yellow oil (3.07 g, 91%).

^{1}H NMR (500 MHz, $CDCl_3$): δ 7.34 – 7.24 (m, 5H), 6.69 (d, *J* = 8.3 Hz, 1H), 6.35 (d, *J* = 8.2 Hz, 1H), 5.03 (d, *J* = 14.5 Hz, 1H), 4.17 (d, *J* = 14.5 Hz, 1H), 3.36 – 3.31 (m, 1H), 3.25 – 3.20 (m, 1H), 2.27 – 2.22 (m, 1H), 2.02 – 1.86 (m, 3H), 1.84 – 1.78 (m, 2H), 0.97 (t, *J* = 7.4 Hz, 3H).

^{13}C NMR (126 MHz, $CDCl_3$): δ 171.9, 144.4, 137.4, 128.6, 128.5, 127.4, 78.8, 50.9, 50.1, 47.6, 31.5, 29.2, 19.6, 9.1.

υ_{max} (neat): 2936, 1632, 1487, 1294, 1194, 696 cm^{-1}.

HRMS (ESI) *m/z*: $[M+Na]^+$ Calcd for $C_{16}H_{20}INNaO$ 392.0487; Found 392.0470.

Compound 41

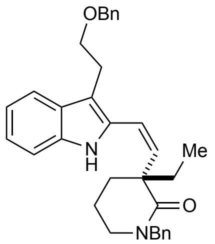

To an oven-dried 100 mL round bottom flask was added **41** (1.74 g, 4.71 mmol, 1.0 equiv.), Pd(dppf)Cl_2 (165 mg, 236 µmol, 5 mol%), Cs_2CO_3 (4.61 g, 14.1 mmol, 3.0 equiv.), and **40** (2.87 g, 7.07 mmol. 1.50 equiv.). The flask was purged with N_2 prior to the addition of THF (47 mL, 0.1 M) and H_2O (1.27 mL, 70.7 mmol, 15.0 equiv.). The mixture was then heated to 65 °C and stirred for 20 h. The reaction was allowed to cool to room temperature before brine (25 mL) was added and the mixture was extracted with EtOAc (3 x 25 mL). The combined organics were dried over Na_2SO_4, filtered, and concentrated to a residue that was purified by flash

column chromatography (5–20% EtOAc in hexane) to give the product as a yellow oil (2.07 g, 89%). Product isomerises on standing to a mixture of *E*/*Z* isomers. Used immediately in the next step.
^{1}H NMR (500 MHz, $CDCl_3$): δ 12.38 (s, 1H), 7.58 (d, *J* = 7.9 Hz, 1H), 7.54 – 7.45 (m, 1H), 7.38 – 7.29 (m, 8H), 7.27 – 7.21 (m, 2H), 7.18 (ddd, *J* = 8.2, 7.0, 1.2 Hz, 1H), 7.07 (ddd, *J* = 7.9, 7.0, 1.0 Hz, 1H), 6.74 (d, *J* = 13.2 Hz, 1H), 5.42 (d, *J* = 13.2 Hz, 1H), 4.75 – 4.63 (m, 2H), 4.57 (s, 2H), 3.73 – 3.65 (m, 2H), 3.34 – 3.17 (m, 3H), 3.16 – 3.10 (m, 1H), 2.16 – 1.96 (m, 3H), 1.96 – 1.82 (m, 3H), 0.78 (t, *J* = 7.4 Hz, 3H).
^{13}C NMR* (126 MHz, $CDCl_3$): δ 173.3, 138.6, 137.5, 136.3, 132.9, 132.5, 130.6, 128.8, 128.4, 128.1, 127.8, 127.6, 122.8, 122.1, 119.5, 118.8, 117.8, 111.6, 110.7, 73.1, 70.9, 50.8, 49.0, 47.9, 32.5, 28.5, 25.1, 19.5, 8.7.
HRMS (ESI) *m/z*: $[M+H]^+$ Calcd for $C_{33}H_{37}N_2O_2$ 493.2855; Found 493.2852.

*Data for the major isomer reported.

Compound 42

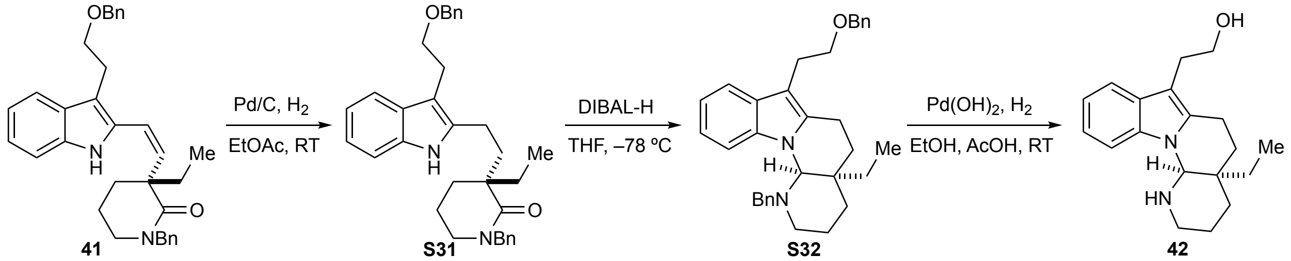

a) Compound S31. A mixture of **41** (526 mg, 1.07 mmol, 1 equiv.) and Pd/C (10 wt%, 114 mg, 107 µmol, 10 mol%) in EtOAc (11 mL, 0.1 M) was stirred vigorously as it was sparged with a balloon of H_2. The mixture was then stirred at room temperature under balloon pressure of H_2 for 3.5 h. The reaction mixture was then filtered through a plug of celite, and the cake washed with MeOH (3 x 10 mL). The filtrate was concentrated to a residue that was purified by flash column chromatography (silica gel, 10–30% EtOAc in hexane) to give the product as a light pink oil (527 mg, 89%).
^{1}H NMR (400 MHz, $CDCl_3$): δ 8.46 (s, 1H), 7.53 – 7.48 (m, 1H), 7.38 – 7.24 (m, 12H), 7.16 – 7.05 (m, 2H), 4.70 (d, *J* = 14.5 Hz, 1H), 4.57 (d, *J* = 12.6 Hz, 3H), 3.69 (t, *J* = 7.5 Hz, 2H), 3.24 (t, *J* = 5.6 Hz, 2H), 3.06 (t, *J* = 7.6 Hz, 2H), 2.91 (ddd, *J* = 14.4, 10.3, 6.7 Hz, 1H), 2.55 (ddd, *J* = 14.4, 10.2, 4.2 Hz, 1H), 2.14 (ddd, *J* = 14.2, 10.3, 4.3 Hz, 1H), 1.88 – 1.62 (m, 6H), 0.90 (t, *J* = 7.5 Hz, 3H).
^{13}C NMR (126 MHz, $CDCl_3$): δ 175.1, 138.6, 137.4, 136.7, 135.4, 128.7, 128.4, 128.3, 127.9, 127.7, 127.5, 127.4, 120.9, 118.8, 117.9, 110.6, 107.2, 73.0, 71.0, 50.7, 47.8, 45.8, 38.3, 31.7, 28.9, 25.0, 21.6, 19.6, 8.5.
υ_{max} (solid): 3277, 2936, 1609, 1452, 1096, 737, 696 cm^{-1}.
HRMS (ESI) *m/z*: $[M+H]^+$ Calcd for $C_{33}H_{39}N_2O_2$ 495.3012; Found 495.2997.

b) Compound S32. A solution of **S31** (143 mg, 0.29 mmol, 1.00 equiv.) in THF (6.1 mL, 0.05 M) was cooled to –78 °C and DIBAL-H (1 M in THF, 1.45 mL, 1.45 mmol, 5.0 equiv.) was added dropwise over 5 minutes. After complete addition, the reaction mixture was allowed to warm to room temperature, stirred for 1 h. The mixture was quenched with sat. aq. Rochelle's salt (10 mL) then stirred overnight at room temperature. The layers were separated, and the aqueous layer was extracted with EtOAc (3 x 10 mL). The combined organics were dried over Na_2SO_4, filtered, and concentrated to a residue that was purified by flash column chromatography (silica gel, 5–40% EtOAc in hexane) to give the product as a colourless gum (76 mg, 55%).
^{1}H NMR (500 MHz, $CDCl_3$): δ 8.59 (s, 1H), 7.49 (dd, *J* = 7.8, 1.2 Hz, 1H), 7.33 – 7.27 (m, 7H), 7.26 – 7.22 (m, 4H), 7.10 (ddd, *J* = 8.1, 7.1, 1.3 Hz, 1H), 7.05 (ddd, *J* = 8.1, 7.1, 1.2 Hz, 1H), 4.67 (d, *J* = 14.6 Hz, 1H), 4.56 – 4.53 (m, 3H), 3.72 – 3.60 (m, 2H), 3.23 – 3.21 (m, 2H), 3.03 (t, *J* = 7.6 Hz, 2H), 2.91 – 2.85 (m, 1H), 2.54 – 2.48 (m, 1H), 2.15 – 2.10 (m, 1H), 1.85 – 1.71 (m, 5H), 1.70 – 1.65 (m, 2H), 0.87 (t, *J* = 7.5 Hz, 3H).
^{13}C NMR (126 MHz, $CDCl_3$): δ 175.3, 138.7, 137.6, 136.9, 135.6, 128.8, 128.5, 128.5, 128.1, 127.8, 127.6, 127.5, 121.0, 119.0, 118.1, 110.7, 107.3, 73.1, 71.1, 50.8, 48.0, 45.9, 38.4, 31.8, 29.0, 25.2, 21.7, 19.7, 8.6.
υ_{max} (solid): 3734, 3628, 2922, 2851, 2361, 1458, 1096, 735 cm^{-1}.
HRMS (ESI) *m/z*: $[M+H]^+$ Calcd for $C_{33}H_{39}N_2O$ 479.3062; Found 479.3053.

c) Compound 42. A mixture of **S30** (73.0 mg, 0.15 mmol, 1.00 equiv.) and $Pd(OH)_2$ (20 wt.% on carbon, 128 mg, 0.18 mmol, 1.20 equiv.) in AcOH/EtOH (2/1, 7.6 mL, 0.02 M) was sparged with a balloon of H_2. The mixture was then stirred at room temperature under balloon pressure of H_2 for 3 h. The reaction mixture was then filtered through a plug of celite, and the cake washed with MeOH (3 x 10 mL). The filtrate was concentrated to a residue then dissolved in DCM (20 mL). 1 M NaOH was added until a pH of *ca.* 10 was achieved. The layers were then separated, and the aqueous layer was extracted with DCM (5 x 10 mL). The combined organics were dried over Na_2SO_4, filtered, and concentrated to a residue that was purified by flash column chromatography (silica gel, 2–5% MeOH in DCM) to give the product as a pale yellow residue (19.0 mg, 42%).

^{1}H NMR (400 MHz, $CDCl_3$): δ 7.51 (d, *J* = 7.6 Hz, 1H), 7.31 – 7.27 (m, 1H), 7.14 (ddd, *J* = 8.1, 7.0, 1.2 Hz, 1H), 7.08 (ddd, *J* = 8.0, 7.1, 1.1 Hz, 1H), 4.79 (s, 1H), 3.83 (t, *J* = 6.4 Hz, 2H), 3.08 – 3.00 (m, 2H), 2.97 – 2.90 (m, 2H), 2.87 – 2.77 (m, 2H), 2.51 (td, *J* = 13.1, 6.7 Hz, 1H), 1.91 – 1.87 (m, 3H), 1.76 – 1.66 (m, 1H), 1.59 (dq, *J* = 15.0, 7.6 Hz, 1H), 1.55 – 1.44 (m, 3H), 1.22 – 1.17 (m, 1H), 0.88 (t, *J* = 7.5 Hz, 3H).

^{13}C NMR (126 MHz, $CDCl_3$): δ 135.5, 132.9, 129.2, 120.7, 119.7, 118.2, 108.4, 106.2, 71.6, 62.7, 45.7, 35.2, 34.1, 28.8, 27.8, 21.7, 21.6, 18.7, 7.2.

υ_{max} (solid): 3303, 2926, 2853, 1460, 1308, 1043, 737 cm^{-1}.

HRMS (ESI) *m/z*: $[M+H]^+$ Calcd for $C_{19}H_{27}N_2O$ 299.2123; Found 299.2110.

Spectral data in agreement with literature values.[15]

5. X-Ray Crystallography Data

CCDC 2133112 (**Compound 3**) and 2149746 (Compound **4**) contain the supplementary crystallographic data for this study. The data can be obtained free of charge from the Cambridge Crystallographic Data Centre via www.ccdc.cam.ac.uk/structures.

Compound 3

X-ray quality crystals isolated by liquid-liquid diffusion at room temperature by dissolving the sample in MeCN and layering with Et_2O.

Table 1 Crystal data and structure refinement for 3

Identification code	3
Empirical formula	$C_{14}H_{15}BN_2O_4$
Formula weight	286.09
Temperature/K	125
Crystal system	monoclinic
Space group	$P2_1/c$
a/Å	8.91842(12)
b/Å	11.69930(14)
c/Å	13.55730(17)
α/°	90.0000
β/°	103.0910(14)

γ/°	90.0000
Volume/Å^3	1377.80(3)
Z	4
ρ_{calc}g/cm^3	1.379
μ/mm^{-1}	0.836
F(000)	600.0
Crystal size/mm^3	0.180 × 0.120 × 0.030
Radiation	Cu Kα (λ = 1.54184)
2Θ range for data collection/°	10.102 to 151.238
Index ranges	$-11 \leq h \leq 11$, $-14 \leq k \leq 14$, $-16 \leq l \leq 16$
Reflections collected	14684
Independent reflections	2771 [R_{int} = 0.0189, R_{sigma} = 0.0082]
Data/restraints/parameters	2771/1/196
Goodness-of-fit on F^2	1.055
Final R indexes [I>=2σ (I)]	R_1 = 0.0409, wR_2 = 0.1072
Final R indexes [all data]	R_1 = 0.0413, wR_2 = 0.1081
Largest diff. peak/hole / e Å^{-3}	0.35/-0.22

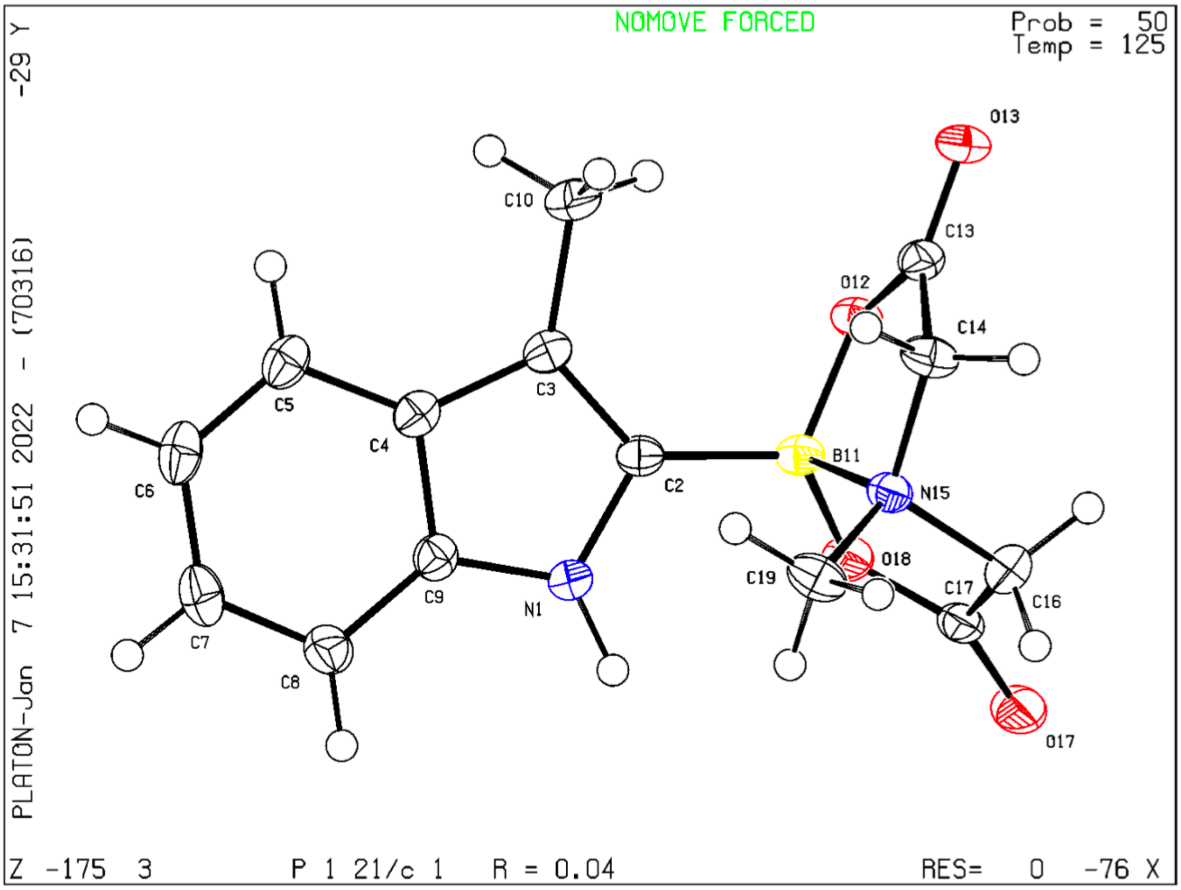

Compound 4

X-ray quality crystals isolated by liquid-liquid diffusion at room temperature by dissolving the sample in MeCN and layering with Et_2O.

Table 1 Crystal data and structure refinement for 4

Identification code	4
Empirical formula	$C_{21}H_{19}BN_2O_5$
Formula weight	390.20
Temperature/K	173
Crystal system	monoclinic
Space group	$P2_1/c$
a/Å	14.7636(15)
b/Å	12.0831(12)
c/Å	10.7076(11)
α/°	90.0000

$\beta/°$	97.887(2)
$\gamma/°$	90.0000
Volume/$Å^3$	1892.1(3)
Z	4
$\rho_{calc}g/cm^3$	1.370
μ/mm^{-1}	0.097
F(000)	816.0
Crystal size/mm^3	0.100 × 0.100 × 0.020
Radiation	Mo Kα (λ = 0.71075)
2Θ range for data collection/°	5.562 to 50.718
Index ranges	$-17 \leq h \leq 17$, $-14 \leq k \leq 14$, $-12 \leq l \leq 12$
Reflections collected	35689
Independent reflections	3469 [R_{int} = 0.1314, R_{sigma} = 0.0503]
Data/restraints/parameters	3469/0/264
Goodness-of-fit on F^2	1.053
Final R indexes [I>=2σ (I)]	R_1 = 0.0525, wR_2 = 0.1332
Final R indexes [all data]	R_1 = 0.0590, wR_2 = 0.1415
Largest diff. peak/hole / e $Å^{-3}$	0.39/-0.23

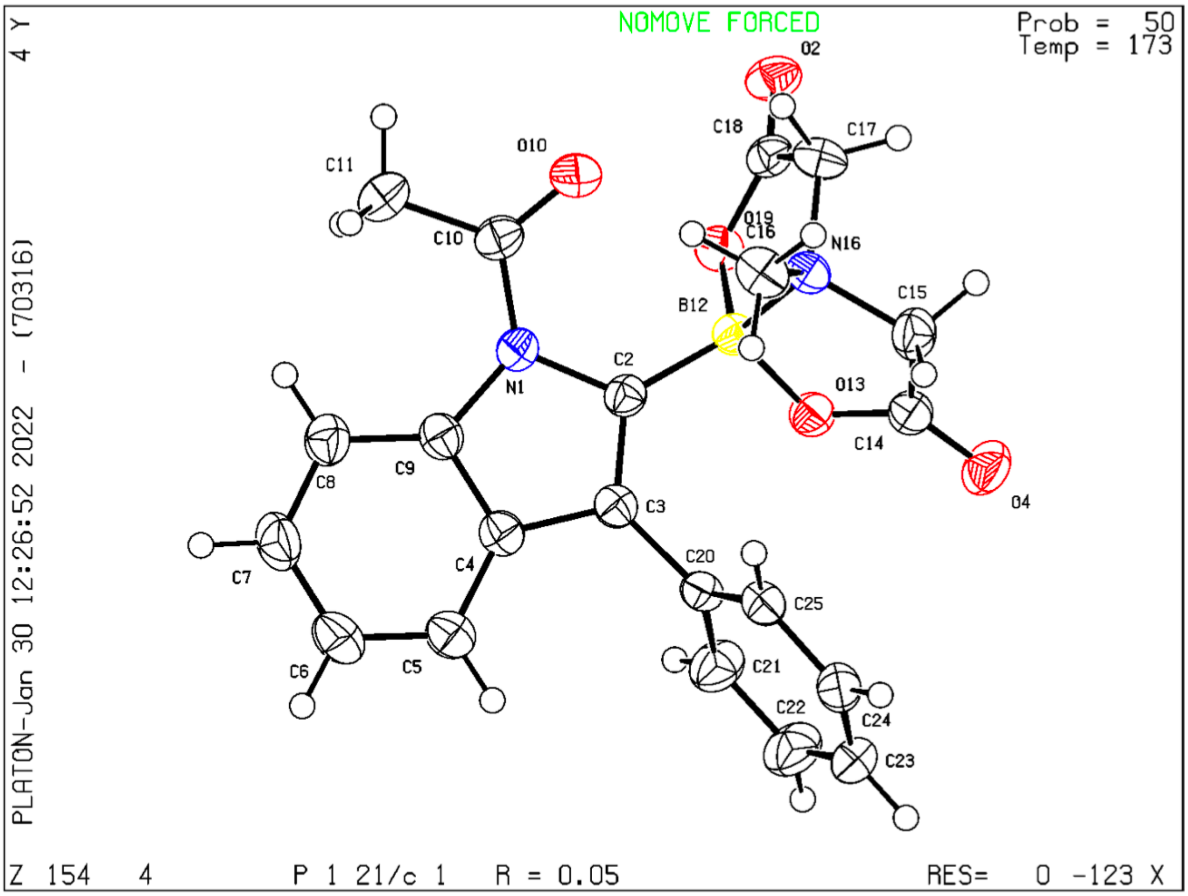

6. BMIDA Volume Calculation

Maximum Tolman cone angle measured mathematically to be 152.04º from SCXRD of compound **3** (assuming perfect free rotation, constraining C–B to 2.28 Å and r_{vdw}(H) = 1.1 Å. *Cf.* calculated $\Theta_{max(Ph)}$ = 129º in gas phase iodobenzene I-C(Ph) = 2.113 Å).[16] We attempted to model exact cone angle and solid angle using the methods described by Aggarwal and coworkers[17] using the scripts developed by Allen and coworkers[18] using the program Mathematica;[19] however, the software was unable to calculate a real value for either cone angle or solid angle.

7. References

1. Armarego, W. L. F. *Purification of Laboratory Chemicals*, 8th Ed., Elsevier: Amsterdam, 2017.
2. Fra, L.; Millán, A.; Souto, J. A.; Muñiz, K. *Angew. Chem. Int. Ed.* **2014**, *53*, 7349–7353.
3. Ikeda, A.; Omote, M.; Kusumoto, K.; Komori, M.; Tarui, A.; Sato, K.; Ando, A. *Org. Biomol. Chem.* **2016**, *14*, 2127–2133.
4. Politanskaya, L. V.; Chuikov, I. P.; Tretyakov, E. V.; Shteingarts, V. D.; Ovchinnikova, L. P.; Zakharova, O. D.; Nevinsky, G. A. *J. Fluor. Chem.* **2015**, *178*, 142–153
5. Mejía-Oneto, J. M.; Padwa, A. *Org. Lett.* **2006**, *8*, 3275–3278.
6. (a) Ueoka, R.; Bortfeld-Miller, M.; Morinaka, B. I.; Vorholt, J. A.; Piel, J. *Angew. Chem. Int. Ed.* **2018**, *57*, 977–981. (b) Schmidt, J.; Eschgfäller, B.; Benner, S. A. *Helv. Chim. Acta* **2003**, *86*, 2937–2958.
7. Weinstain, R.; Sagi, A.; Karton, N.; Shabat, D. *Chem. Eur. J.* **2008**, *14*, 6857–6861.
8. Bruch, A.; Fröhlich, R.; Grimme, S.; Studer, A.; Curran, D. P. *J. Am. Chem. Soc.* **2011**, *133*, 16270–16276.
9. Kathiravan, S.; Nicholls, I. A. *Chem. Eur. J.* **2017**, *23*, 7031–7036.
10. Chaisan, N.; Kaewsri, W.; Thongsornkleeb, C.; Tummatorn, J.; Ruchirawat, S. *Tetrahedron Lett.* **2018**, *59*, 675–680.
11. Morisset, E.; Chardon, A.; Rouden, J.; Blanchet, J. *Eur. J. Org. Chem.* **2020**, *2020*, 388–392.
12. Leitner, C.; Gaich, T. *Chem. Commun.* **2017**, *53*, 7451–7453.

13. Wen, Q.; Jin, J.; Mei, Y.; Lu, P.; Wang, Y. *Eur. J. Org. Chem.* **2013**, 4032–4036.
14. Wilson, K. L.; Kennedy, A. R.; Murray, J.; Greatrex, B.; Jamieson, C.; Watson, A. J. B. *Beilstein J. Org. Chem.* **2016**, *12*, 2005–2011.
15. M. V. Mijangos, L. D. Miranda, *Org. Biomol. Chem.* **2018**, *16*, 9409–9419.
16. Schulz, A. Z. *Anorg. Allg. Chem.* **2014**, *640*, 2183–2192.
17. Fasano, V.; McFord, A. W.; Butts, C. P.; Collins, B. S. L.; Fey, N.; Alder, R. W.; Aggarwal, V. K. *Angew. Chem. Int. Ed.* **2020**, *59*, 22403–22407.
18. (a) Bilbrey, J. A.; Kazez, A. H.; Locklin, J.; Allen, W. D. *J. Chem. Theory Comp.* **2013**, *9*, 5734–5744. (b) Bilbrey, J. A.; Kazez, A. H.; Locklin, J.; Allen, W. D. *J. Comp. Chem.* **2013**, *34*, 1189–1197.
19. Mathematica 8.0; Wolfram Research, Inc.: Champaign, IL, 2010.
