# Supplementary material for: Synthesis of 2-BMIDA Indoles via Heteroannulation: Applications in Drug Scaffold and Natural Product Synthesis
Source: Org Lett. 2022 Apr 15;24(16):3024–7. doi: 10.1021/acs.orglett.2c00959 (PMC9062883; doi:10.1021/acs.orglett.2c00959)
Supplement: Supplementary file 2 — ol2c00959_si_002.pdf [file ol2c00959_si_002.pdf]

Supporting Information

Synthesis of 2-BMIDA indoles via heteroannulation: Applications in drug scaffold and natural product synthesis

George E. Bell,[a] James W. B. Fyfe,[a] Eva M. Israel,[a] Alexandra M. Z. Slawin,[a] Matthew Campbell,[b] and Allan J. B. Watson*[a]

[a] EaStCHEM, School of Chemistry, University of St Andrews, North Haugh, St Andrews, Fife, KY16 9ST, U.K.
[b] GlaxoSmithKline, Medicines Research Centre, Gunnels Wood Road, Stevenage, SG1 2NY, U.K.

*Email: aw260@st-andrews.ac.uk

Contents

NMR Spectra S2

Compound S1:

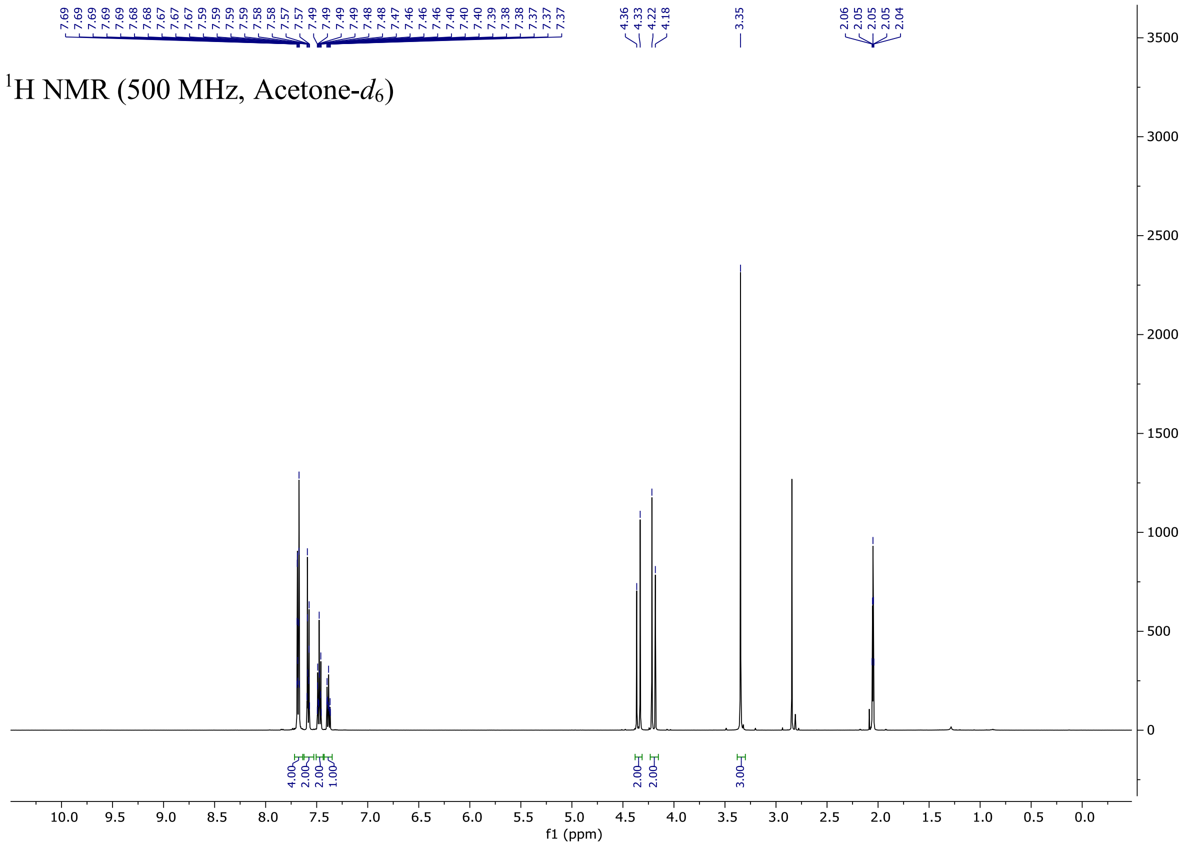

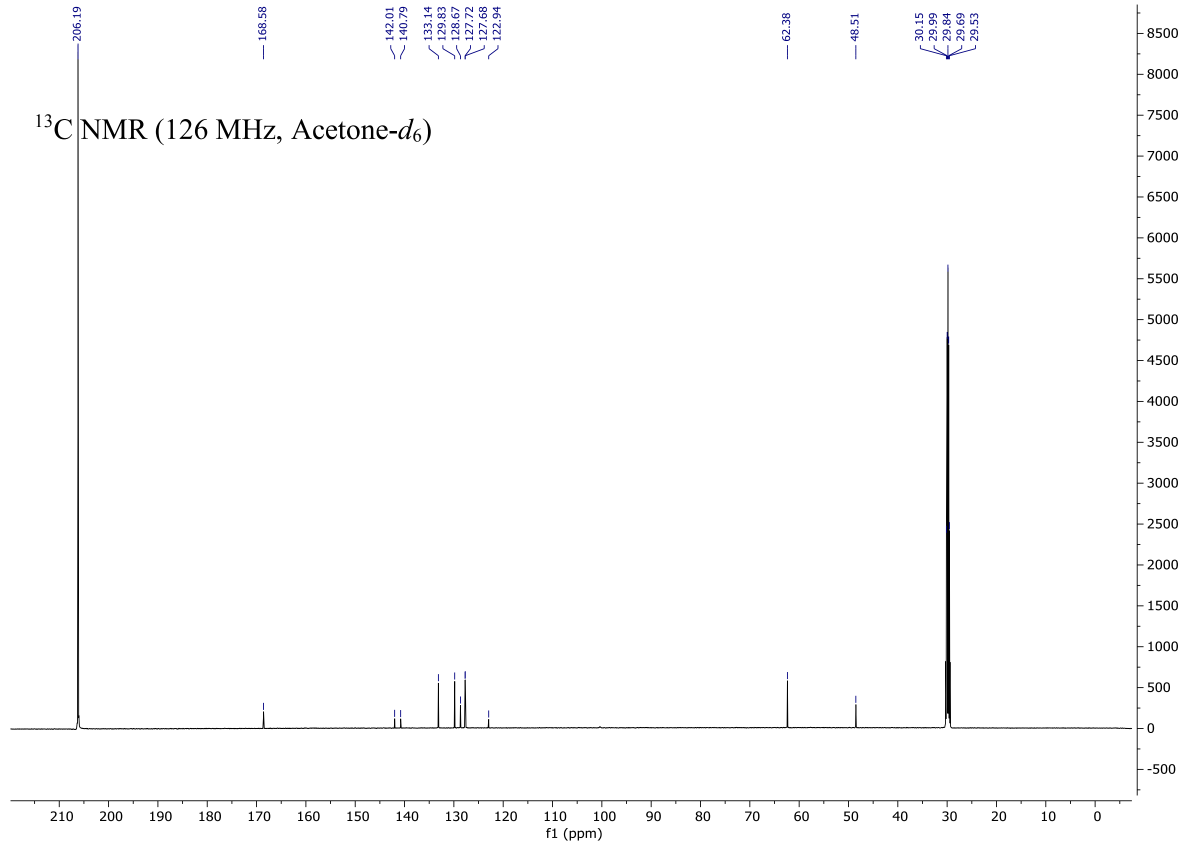

Compound S2:

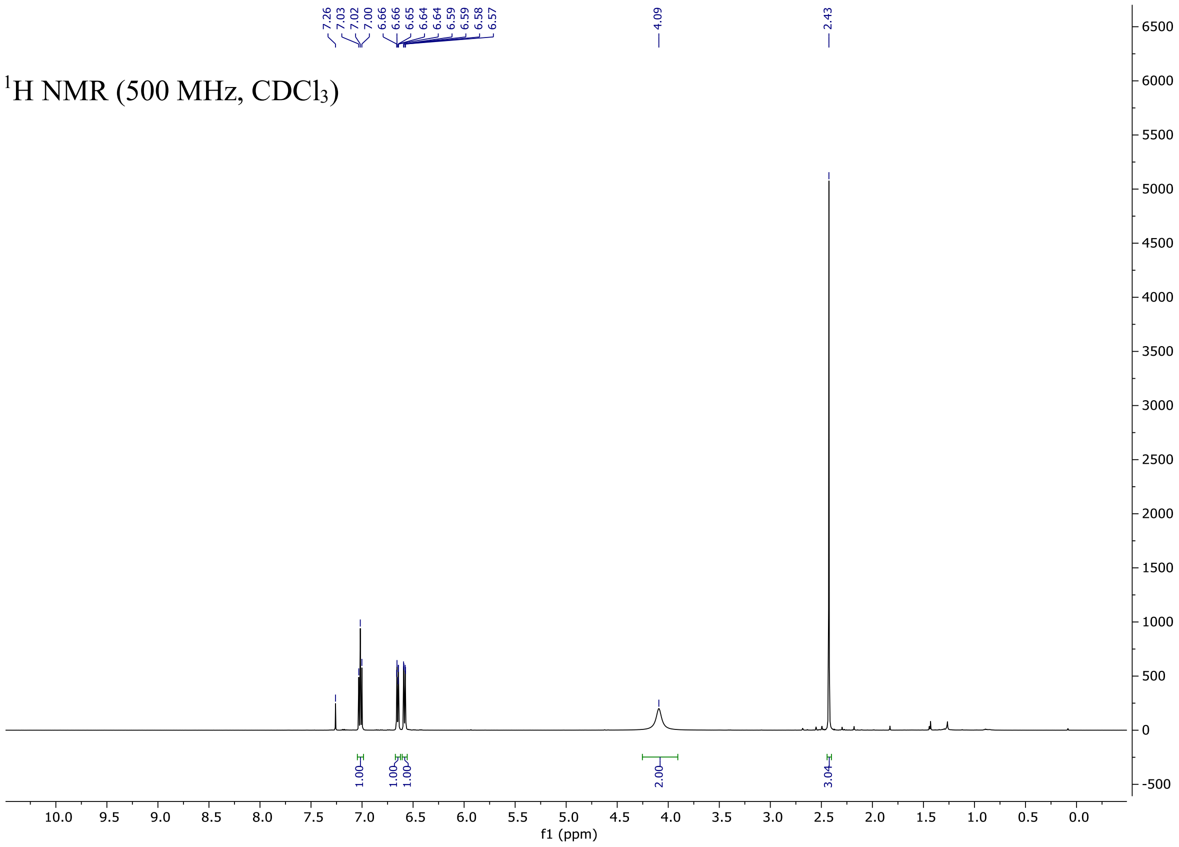

7.26
7.03
7.02
7.00
6.66
6.65
6.64
6.64
6.59
6.59
6.58
6.57
4.09
2.43
1H NMR (500 MHz, CDCl3)
1.00
1.00
1.00
2.00
3.04
f1 (ppm)

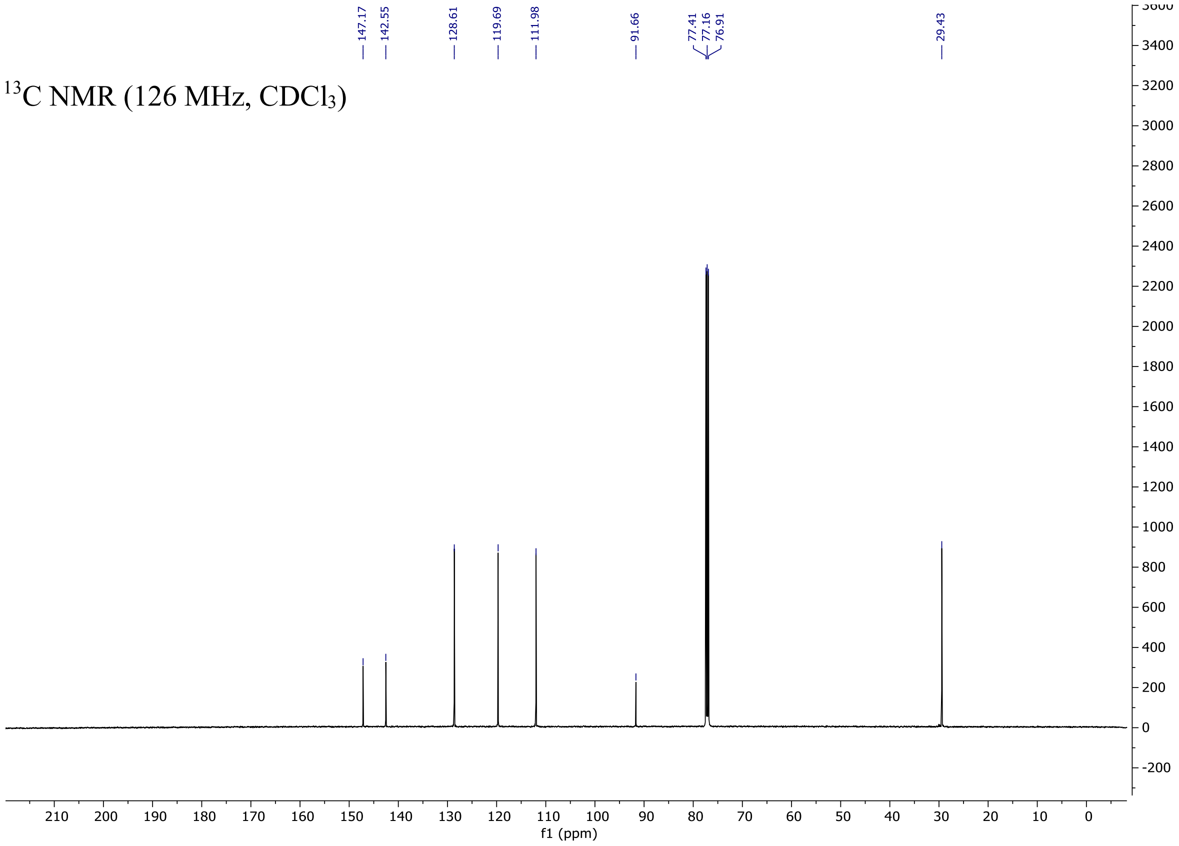

147.17
142.55
128.61
119.69
111.98
91.66
77.41
77.16
76.91
29.43
13C NMR (126 MHz, CDCl3)
f1 (ppm)

Compound S3:

^{1}H NMR (500 MHz, DMSO-d_6)

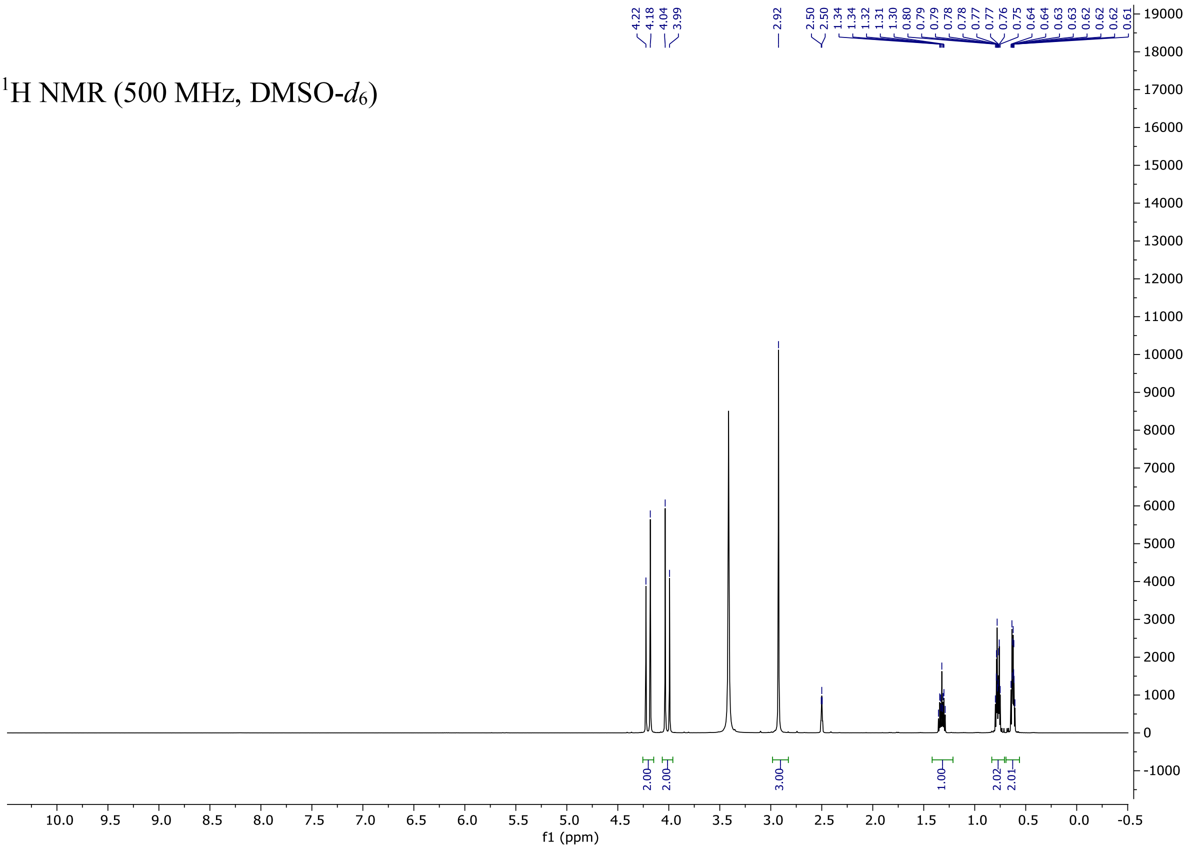

^{13}C NMR (126 MHz, DMSO-d_6)

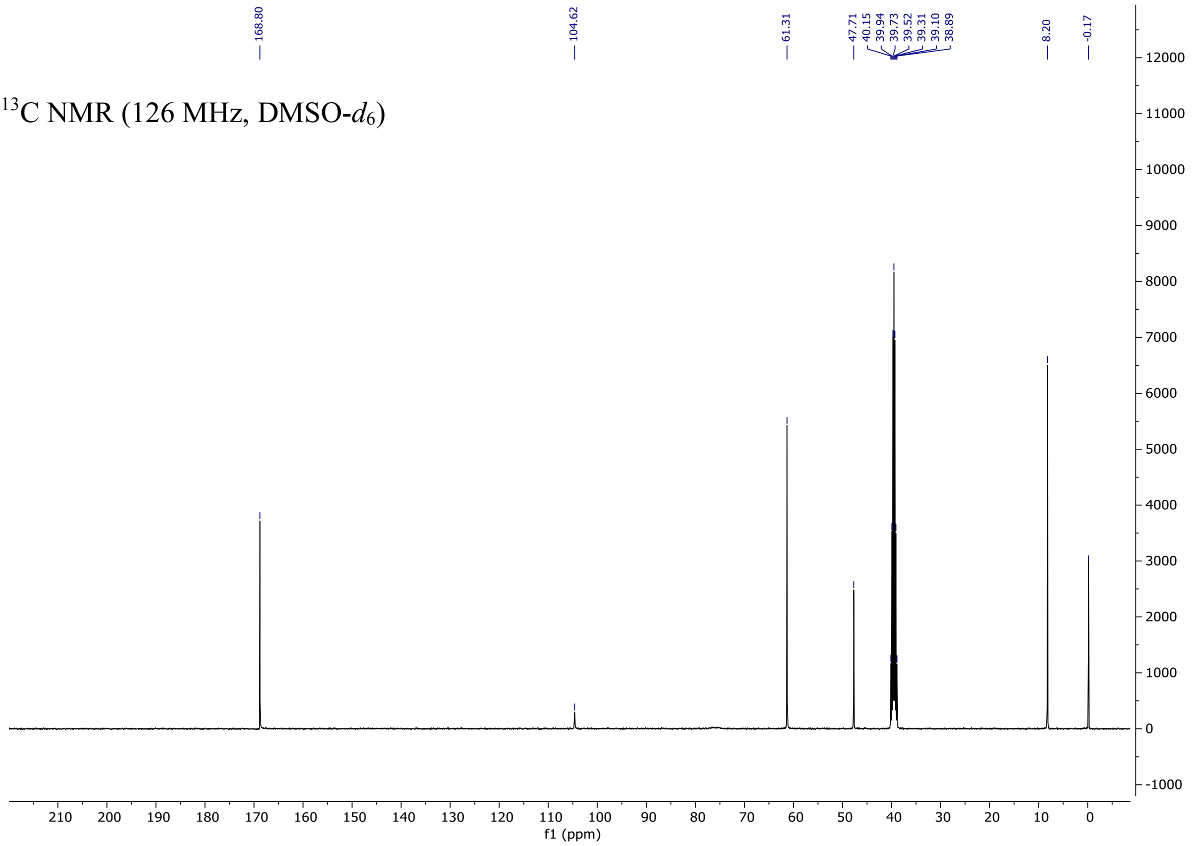

Compound S4:

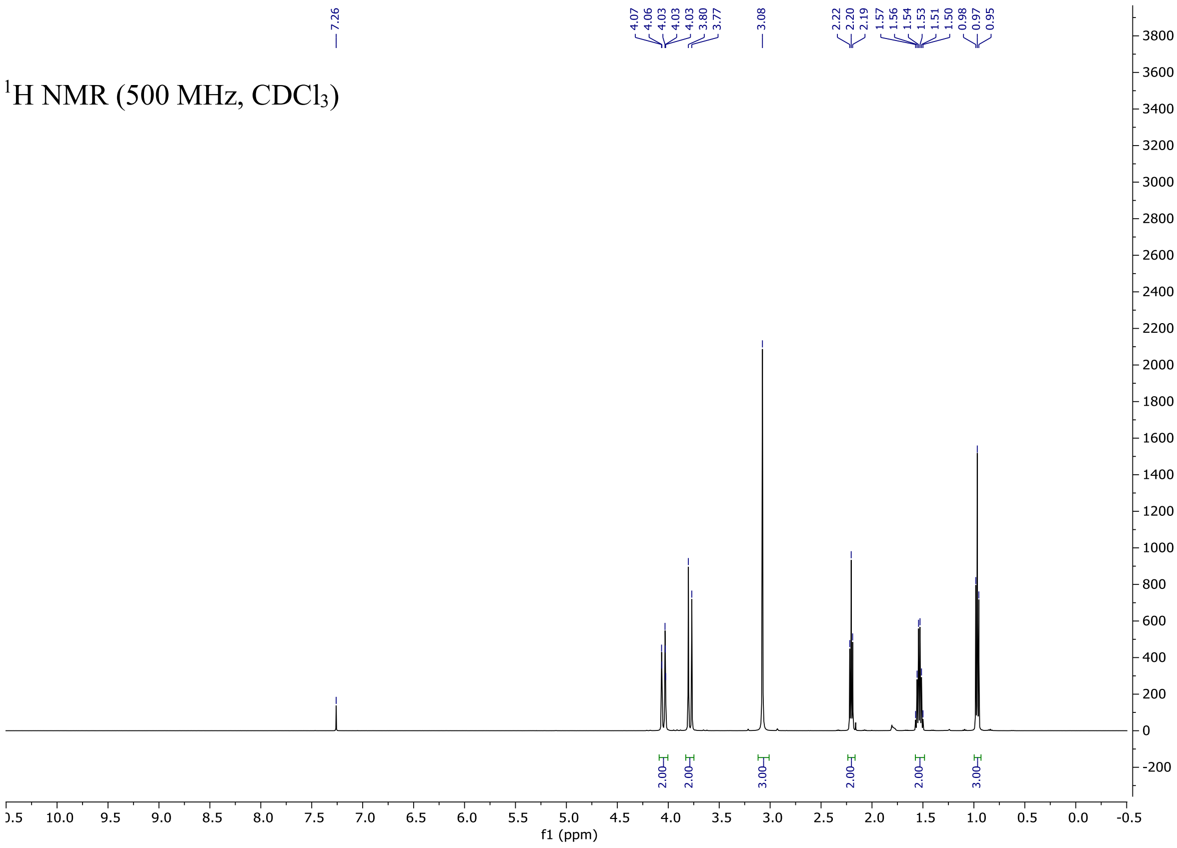

1H NMR (500 MHz, CDCl3)
7.26
4.07
4.06
4.03
4.03
4.03
3.80
3.77
3.08
2.22
2.20
2.19
1.57
1.56
1.54
1.53
1.51
1.50
0.98
0.97
0.95
2.00
2.00
3.00
2.00
2.00
3.00
f1 (ppm)

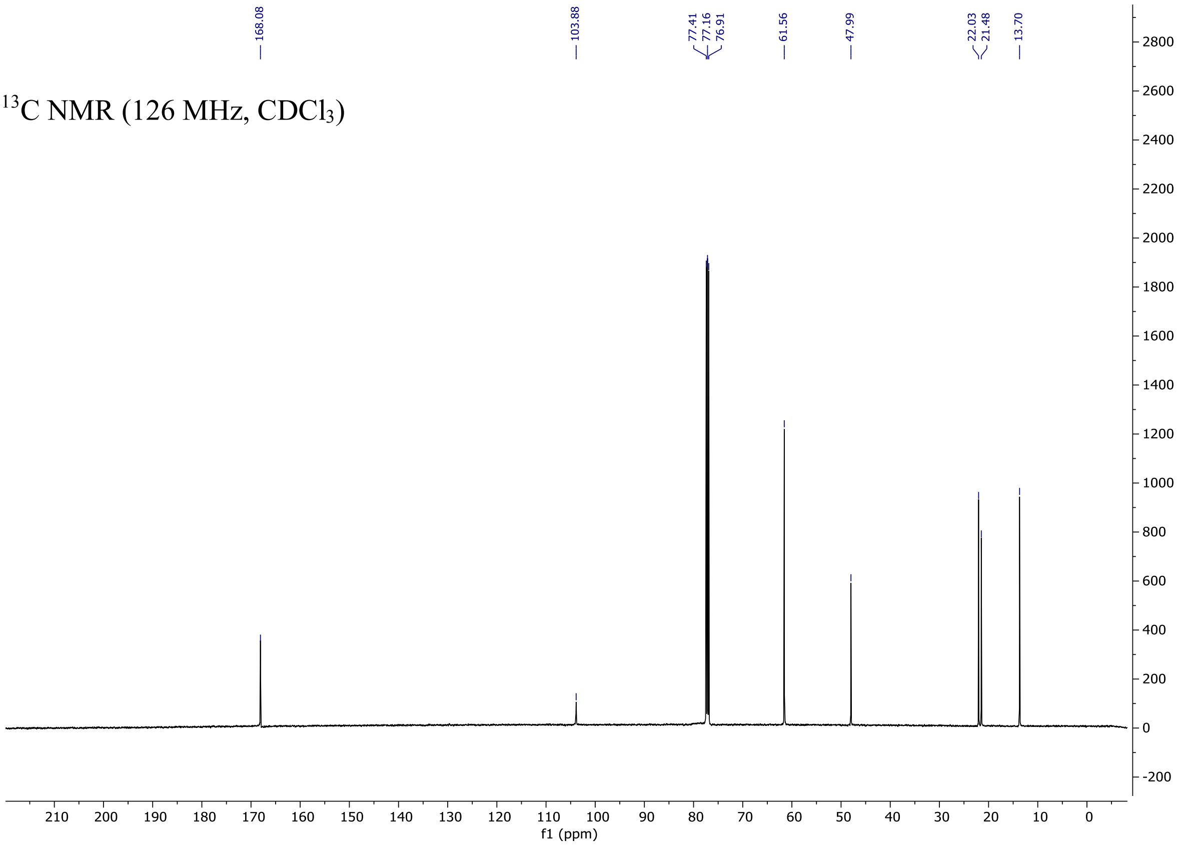

13C NMR (126 MHz, CDCl3)
168.08
103.88
77.41
77.16
76.91
61.56
47.99
22.03
21.48
13.70
f1 (ppm)

Compound S5:

^{1}H NMR (500 MHz, DMSO-d_6)

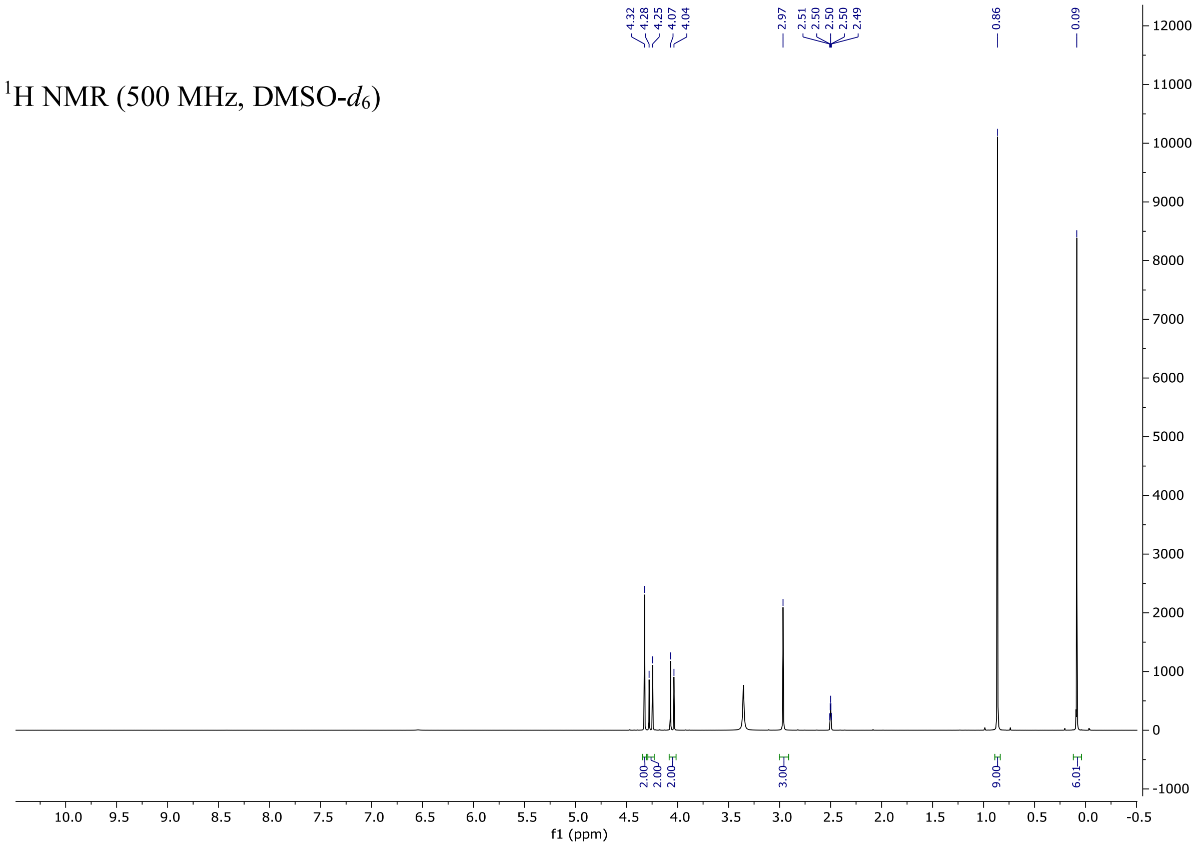

^{13}C NMR (126 MHz, DMSO-d_6)

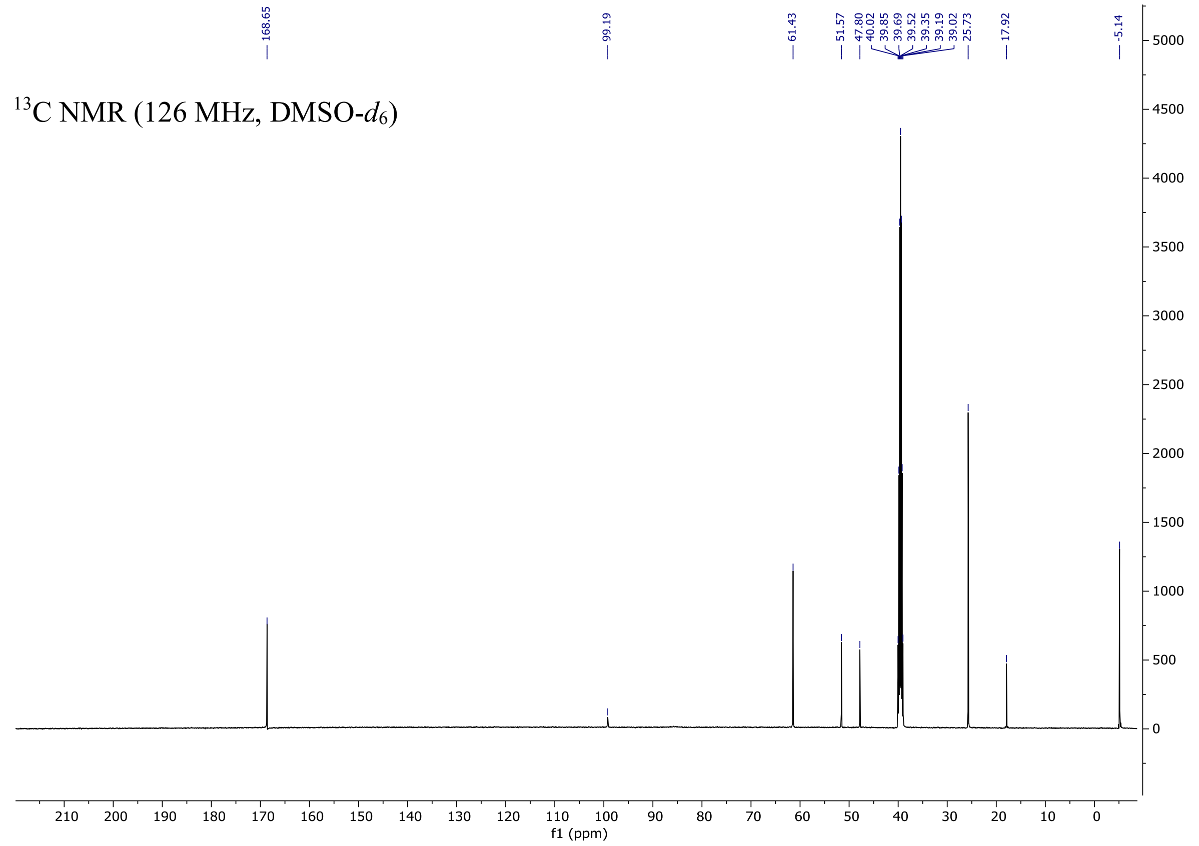

Compound S6:

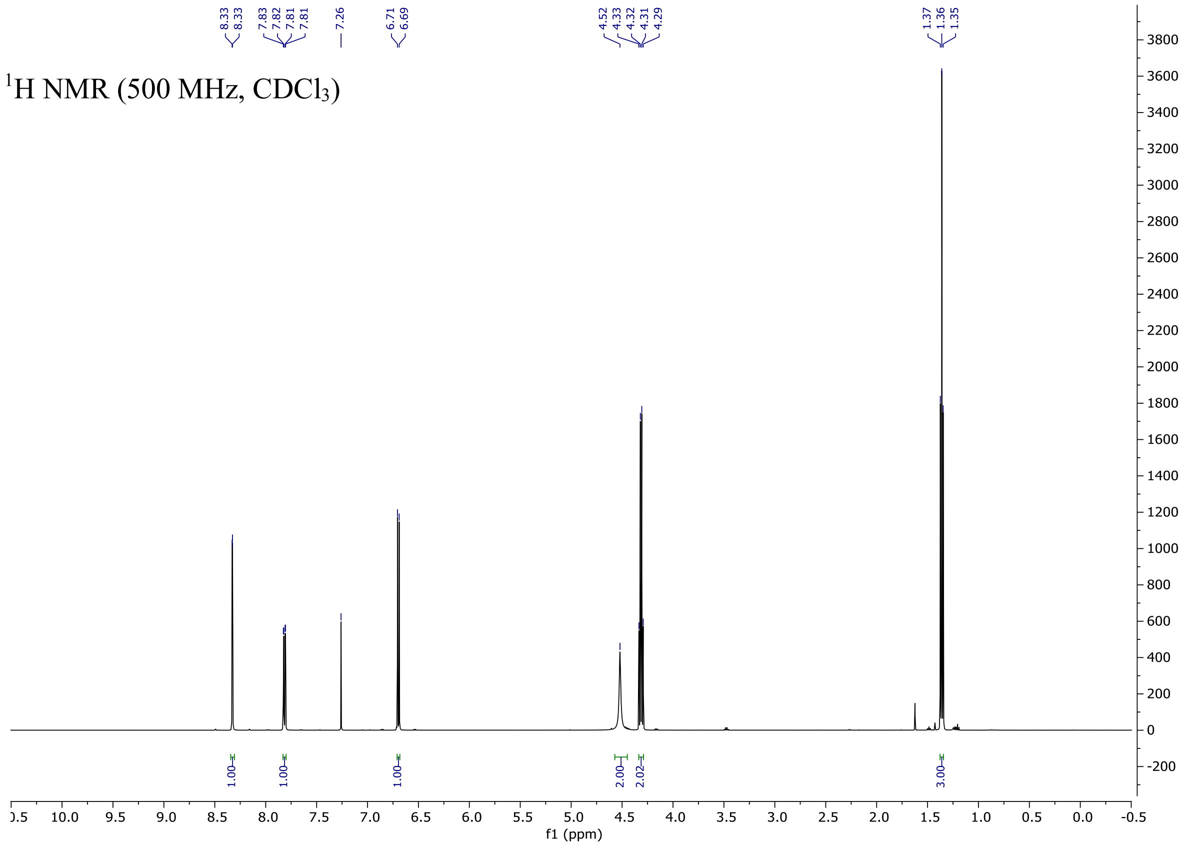

8.33
8.33
7.83
7.82
7.81
7.81
7.26
6.71
6.69
4.52
4.33
4.32
4.31
4.29
1.37
1.36
1.35
1H NMR (500 MHz, CDCl3)
1.00
1.00
1.00
2.00
2.02
3.00
f1 (ppm)

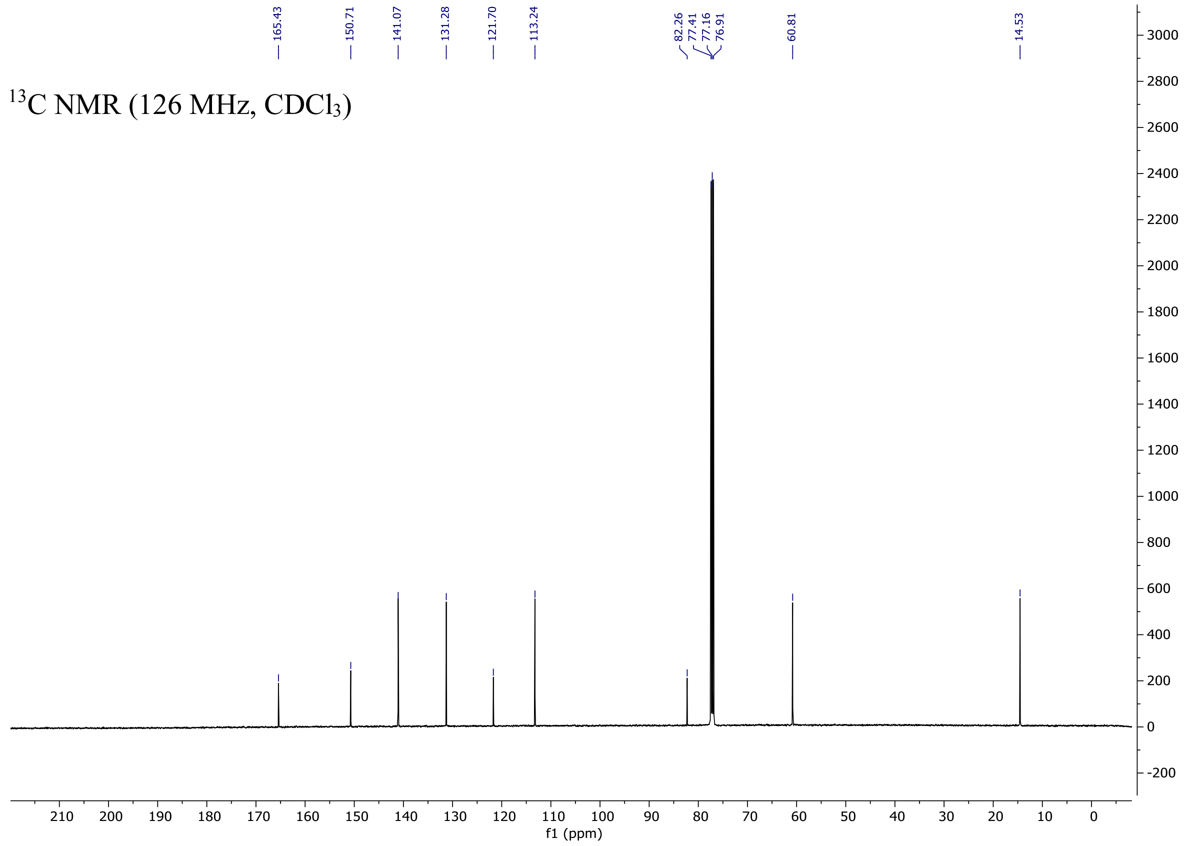

165.43
150.71
141.07
131.28
121.70
113.24
82.26
77.41
77.16
76.91
60.81
14.53
13C NMR (126 MHz, CDCl3)
f1 (ppm)

Compound S7:

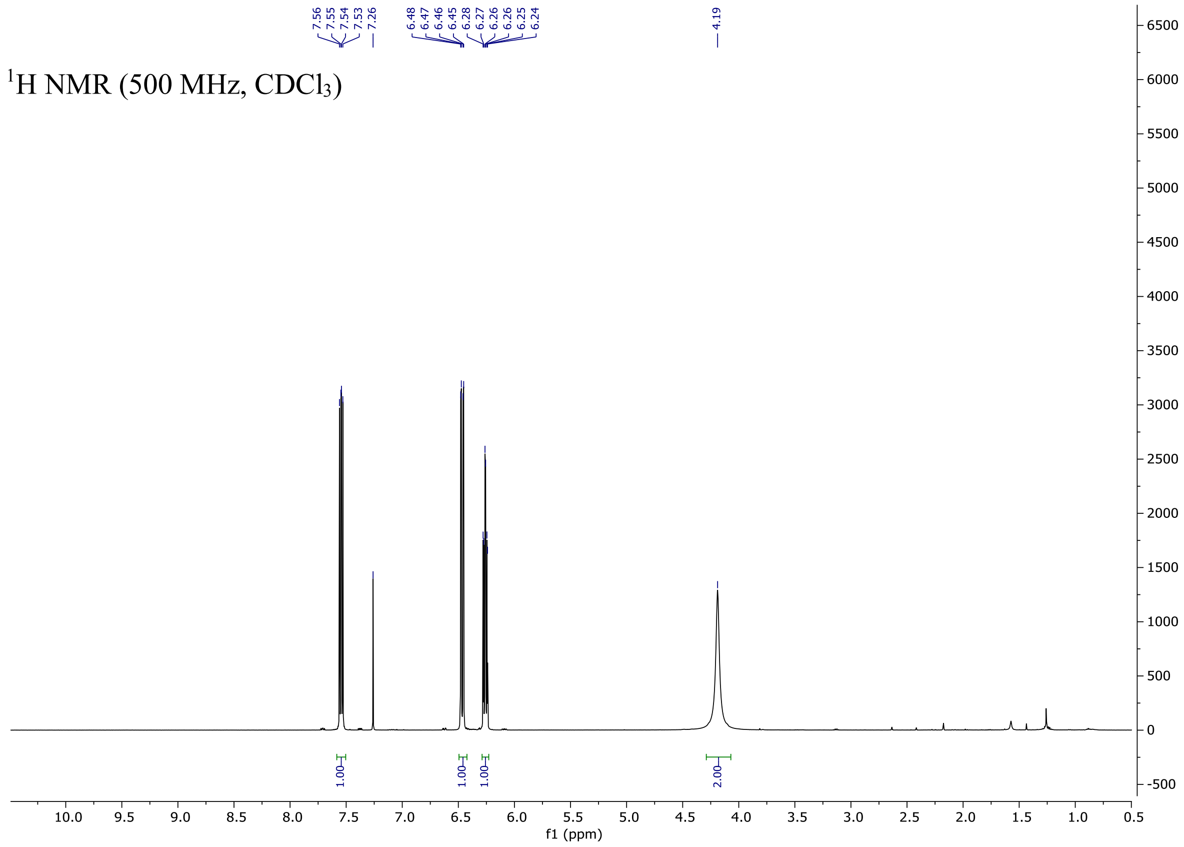
7.56
7.55
7.54
7.53
7.26
6.48
6.47
6.46
6.45
6.28
6.27
6.26
6.26
6.25
6.24
4.19
¹H NMR (500 MHz, CDCl₃)
1.00
1.00
1.00
2.00
f1 (ppm)

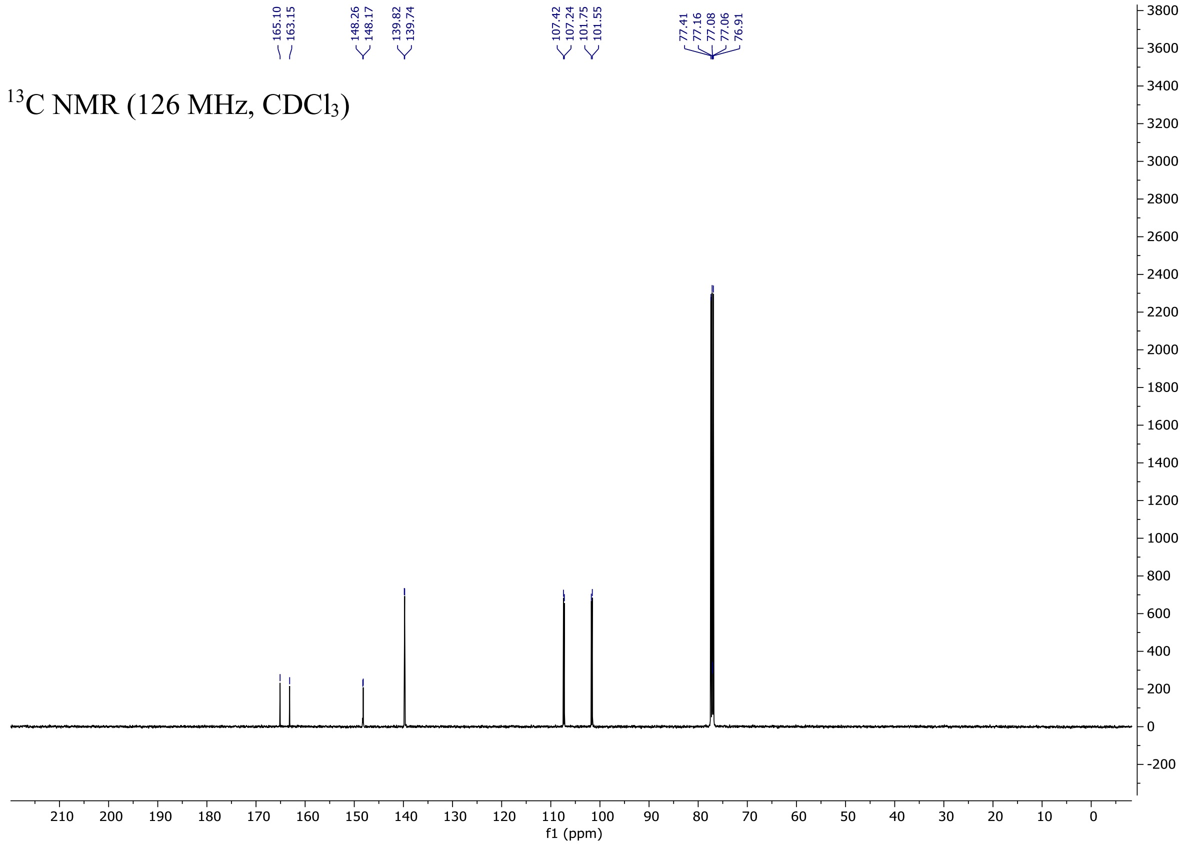
165.10
163.15
148.26
148.17
139.82
139.74
107.42
107.24
101.75
101.55
77.41
77.16
77.08
77.06
76.91
¹³C NMR (126 MHz, CDCl₃)
f1 (ppm)

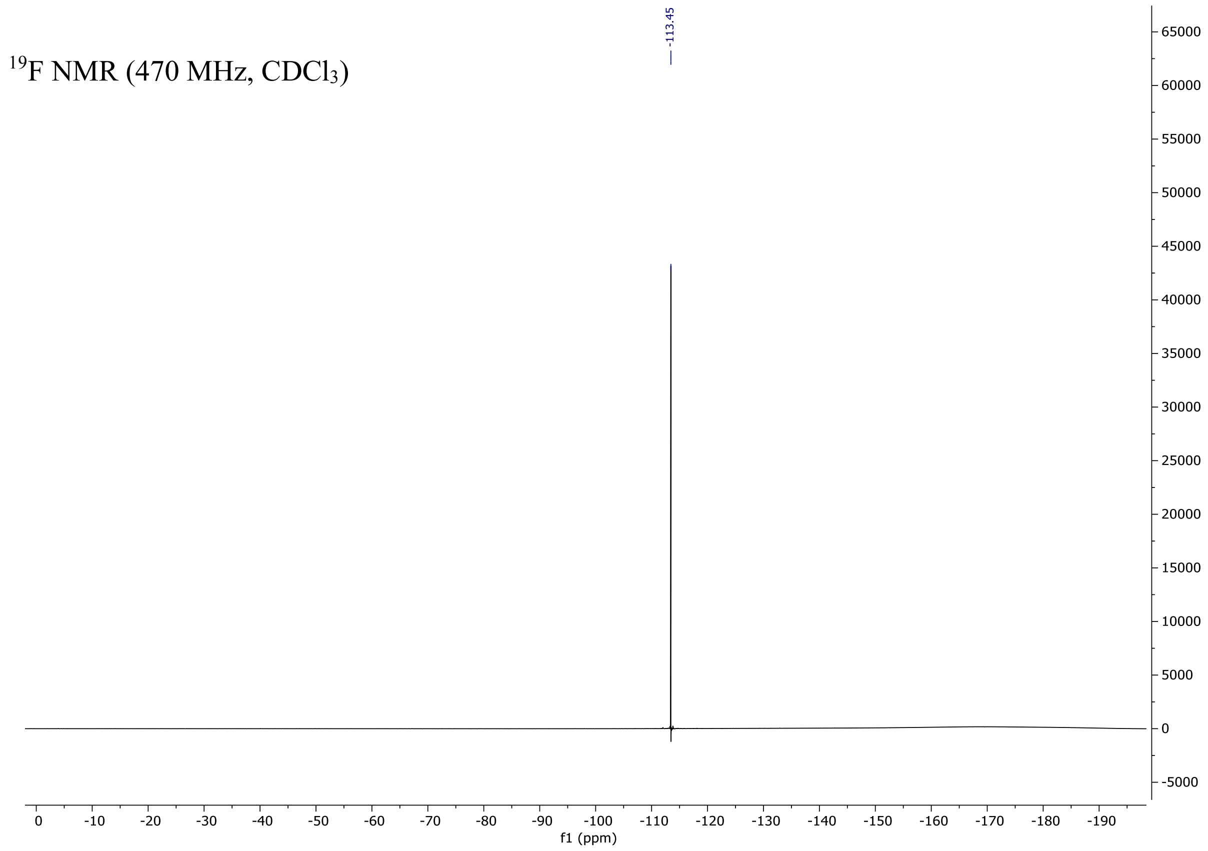

¹⁹F NMR (470 MHz, CDCl₃)
-113.45
65000
60000
55000
50000
45000
40000
35000
30000
25000
20000
15000
10000
5000
0
-5000
0
-10
-20
-30
-40
-50
-60
-70
-80
-90
-100
-110
-120
-130
-140
-150
-160
-170
-180
-190
f1 (ppm)

Compound S8:

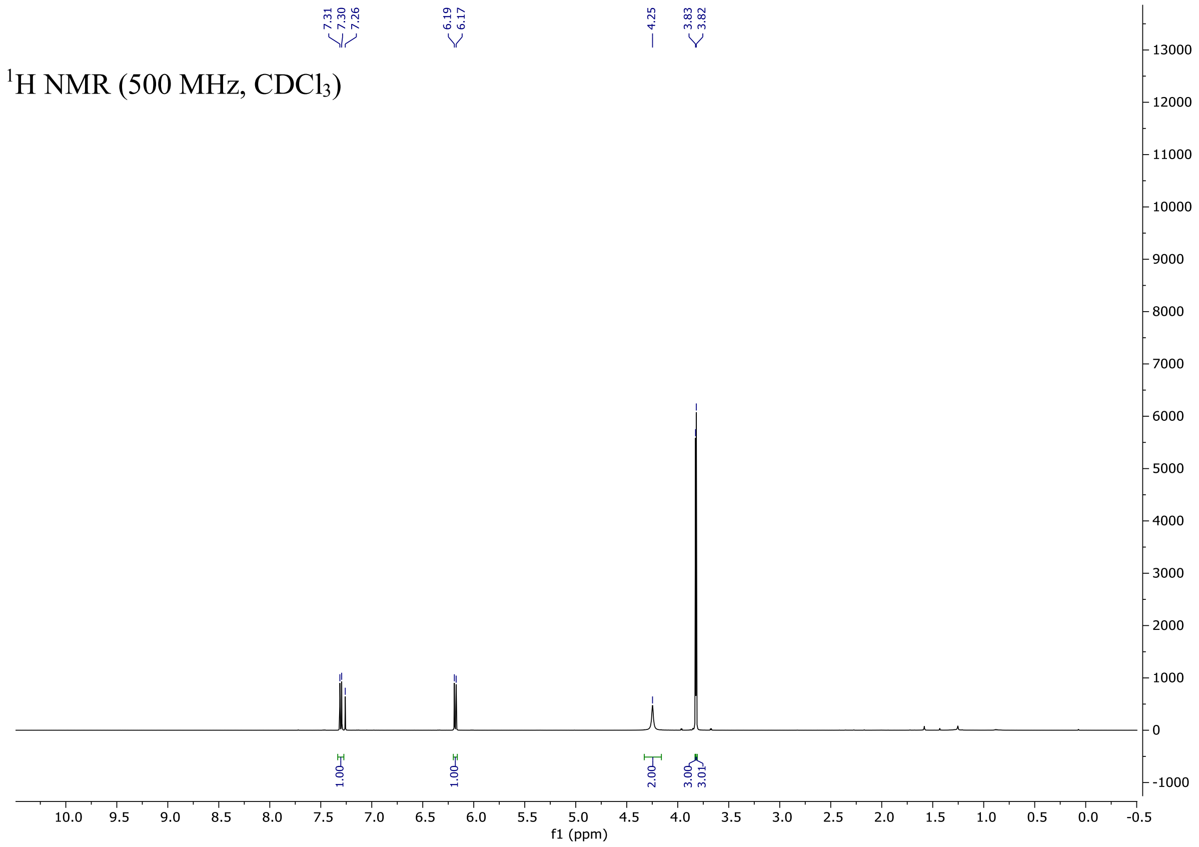

7.31
7.30
7.26
6.19
6.17
4.25
3.83
3.82
^{1}H NMR (500 MHz, $CDCl_3$)
1.00
1.00
2.00
3.00
3.01
f1 (ppm)

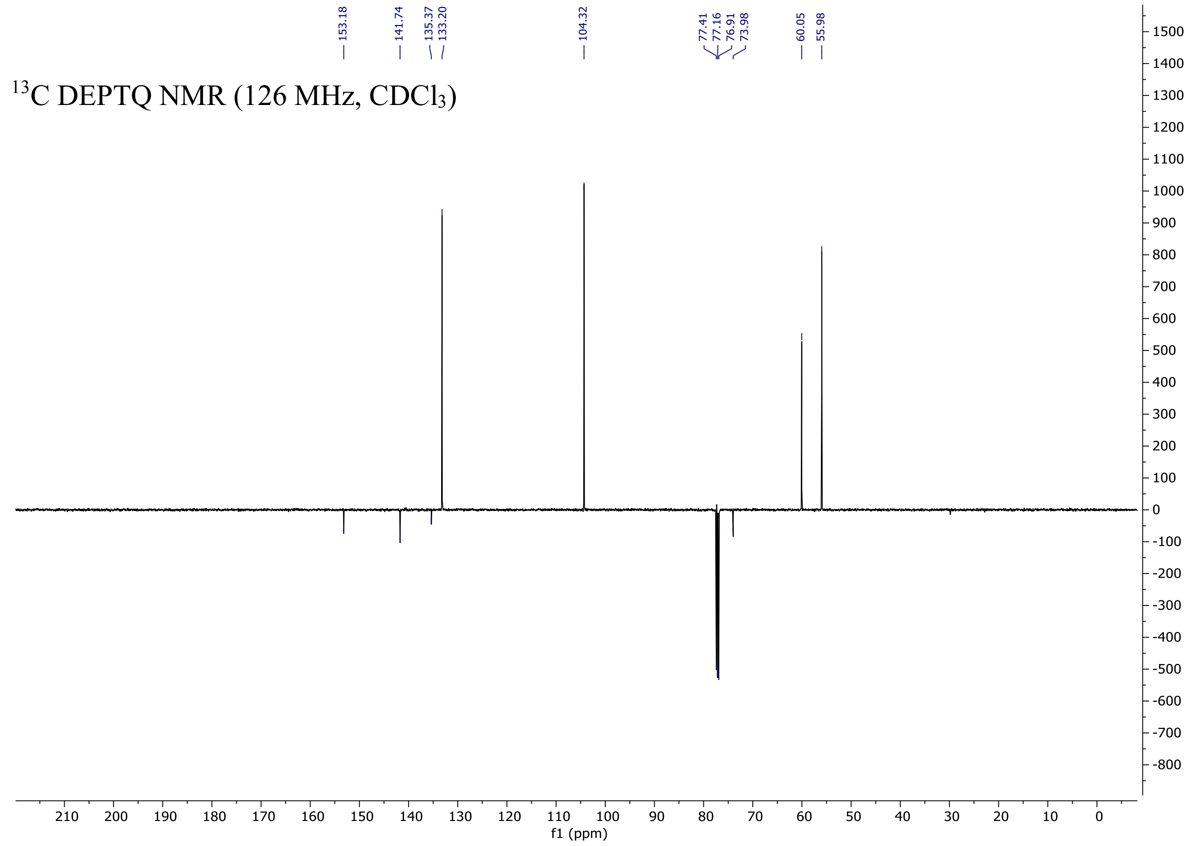

153.18
141.74
135.37
133.20
104.32
77.41
77.16
76.91
73.98
60.05
55.98
^{13}C DEPTQ NMR (126 MHz, $CDCl_3$)
f1 (ppm)

Compound S9:

^{1}H NMR (500 MHz, DMSO-d_6)

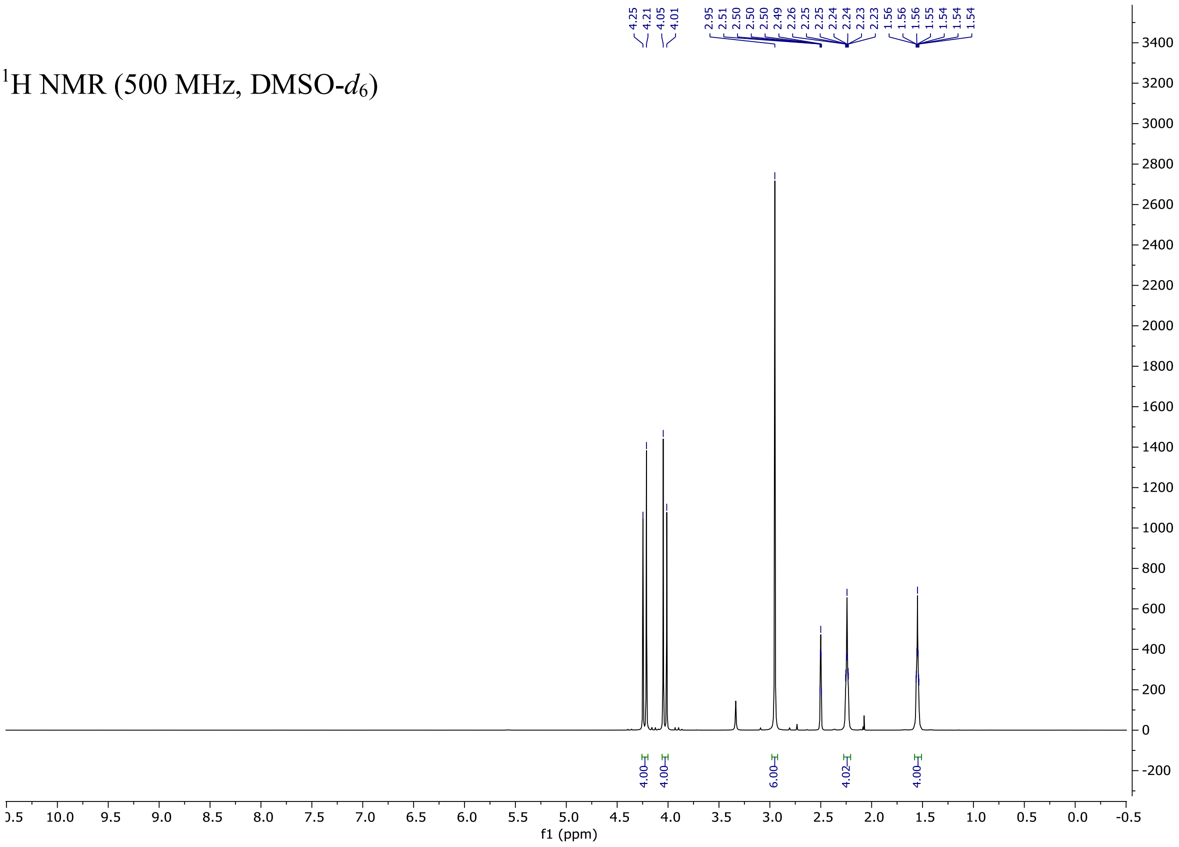

^{13}C NMR (126 MHz, DMSO-d_6)

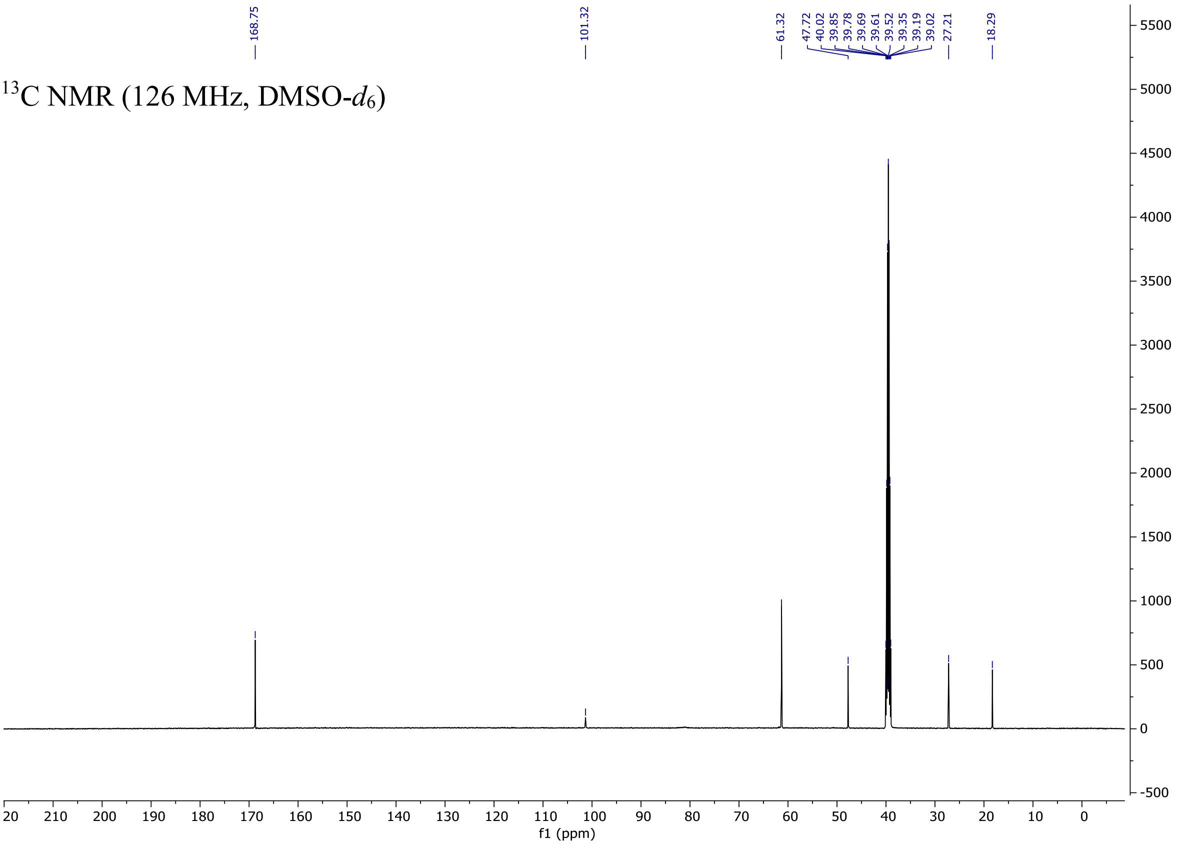

Compound S10:

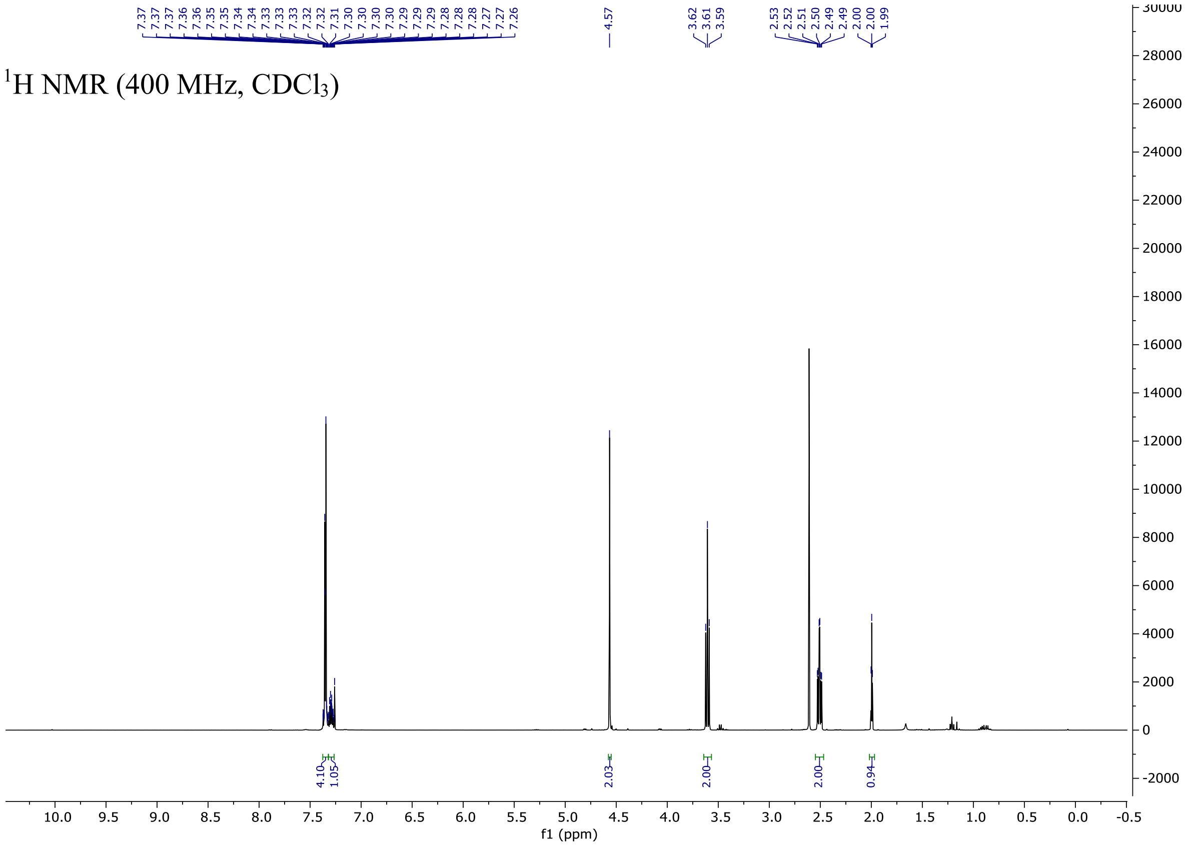

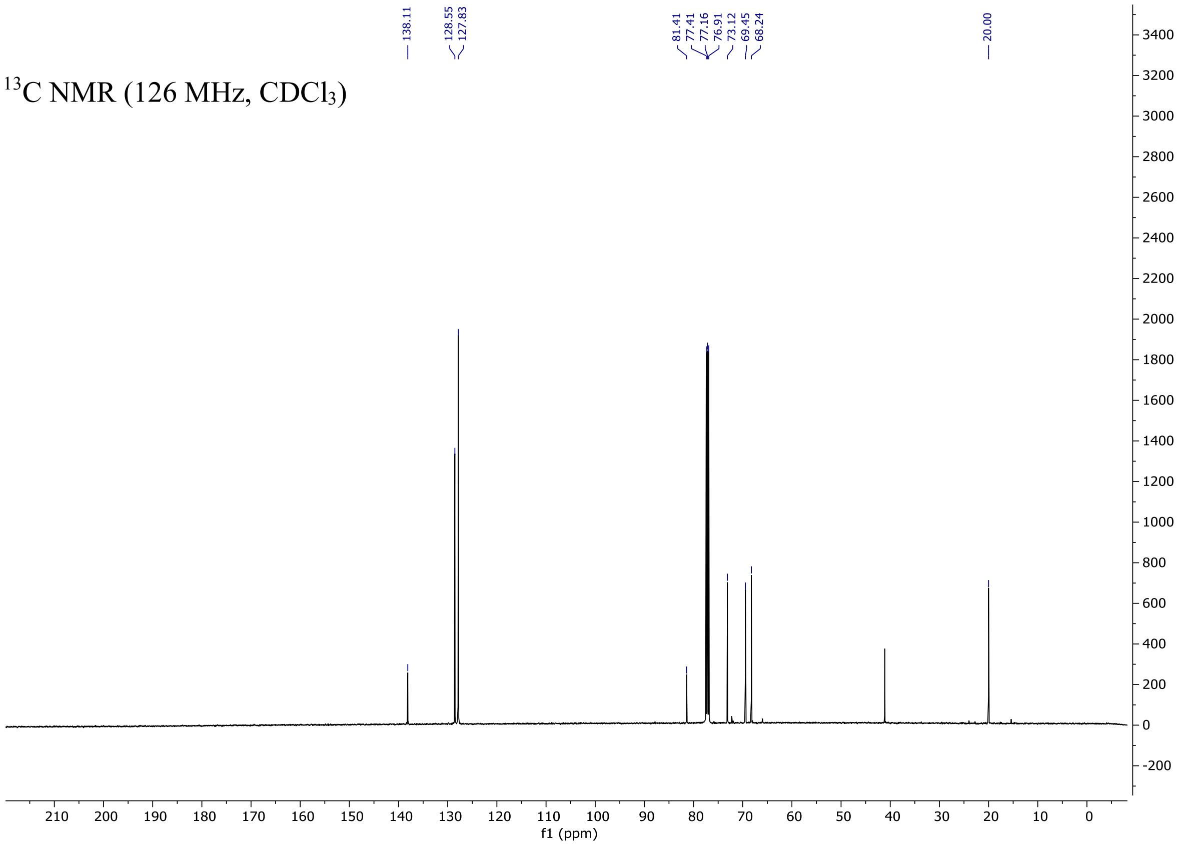

Compound S11:

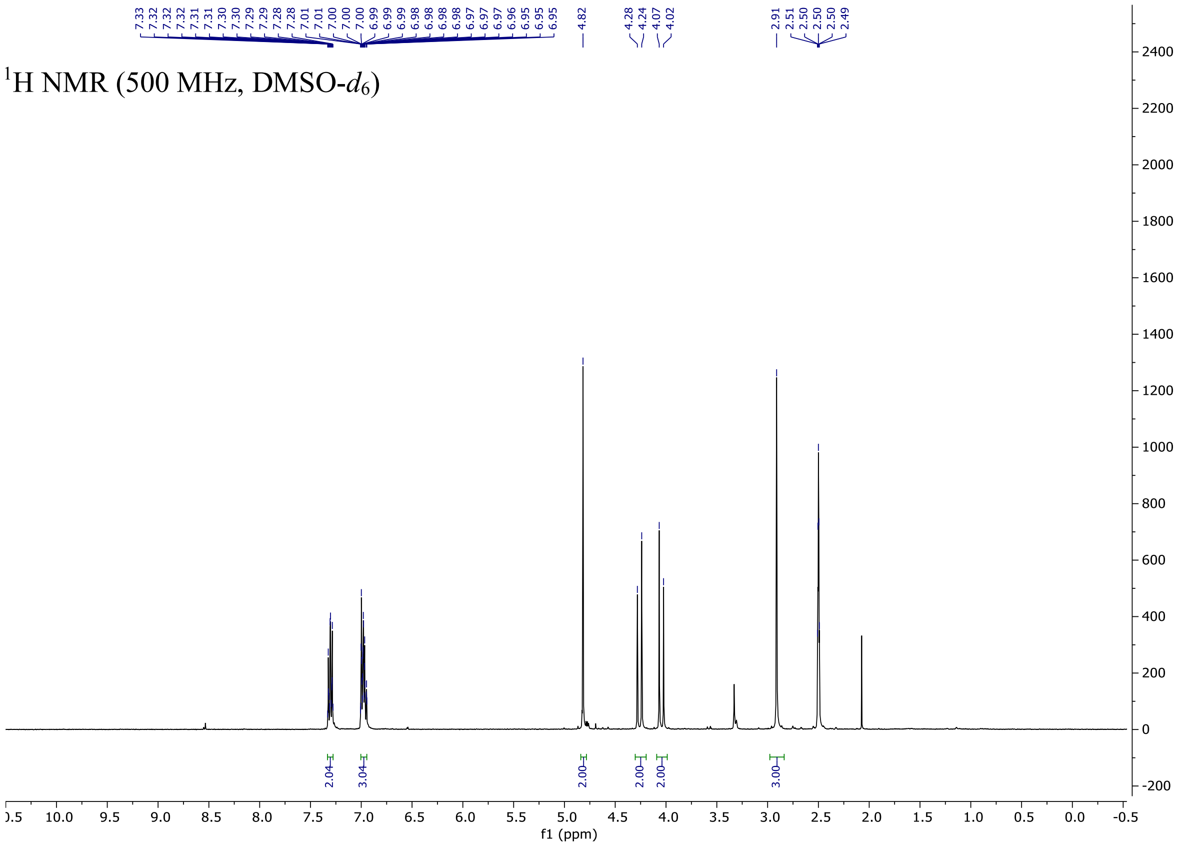

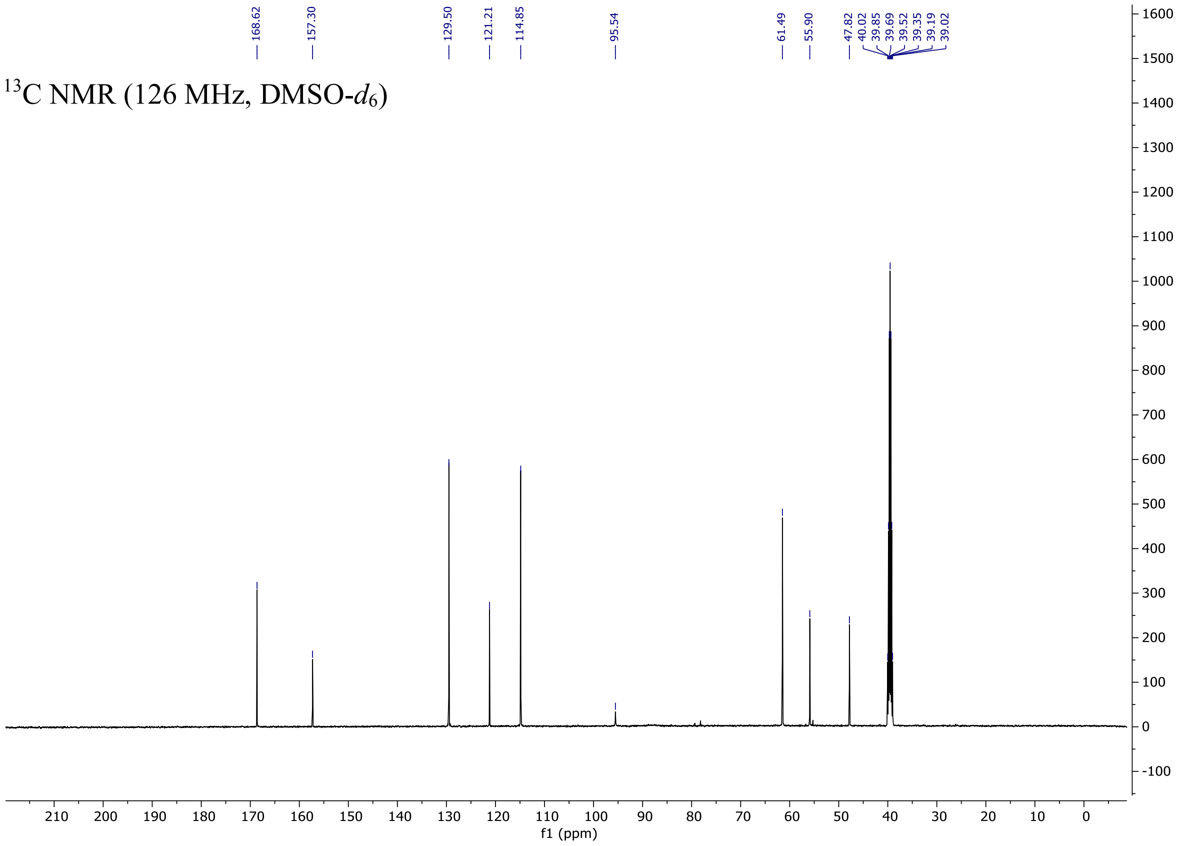

Compound S12:

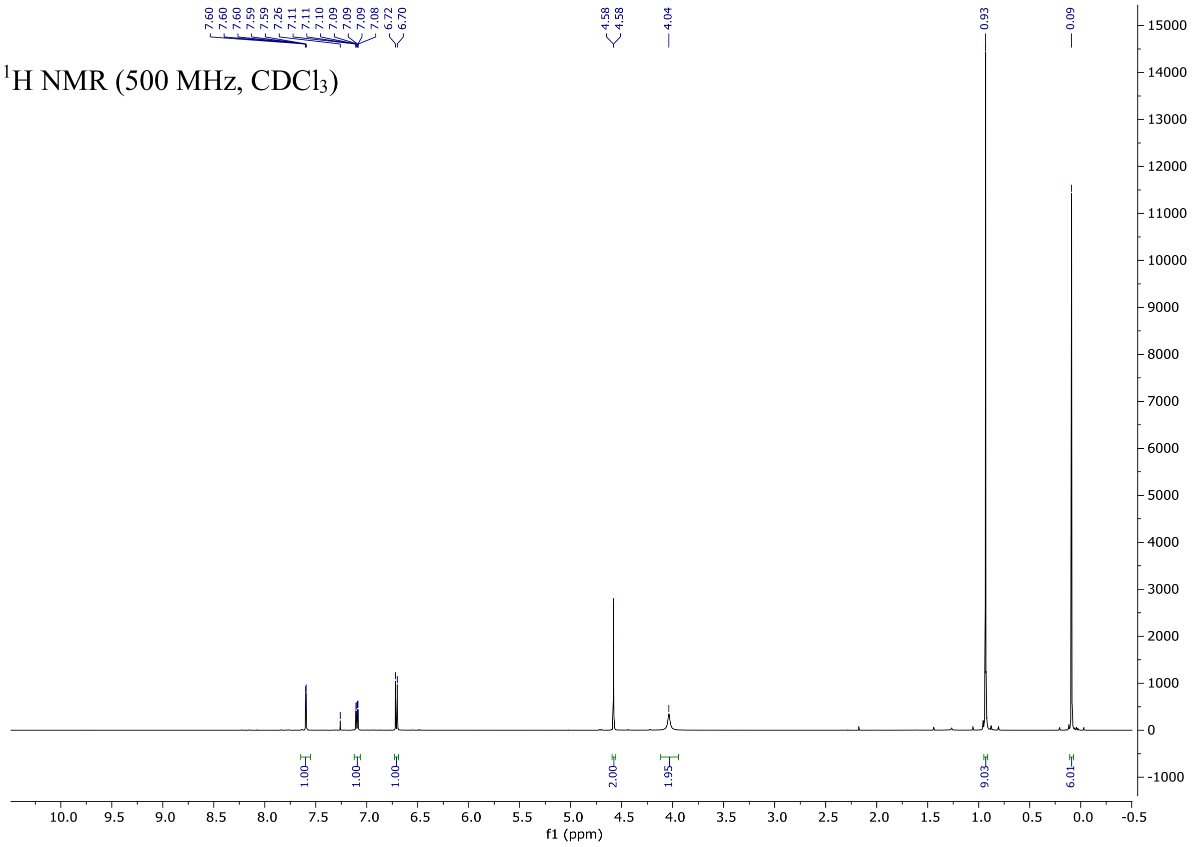

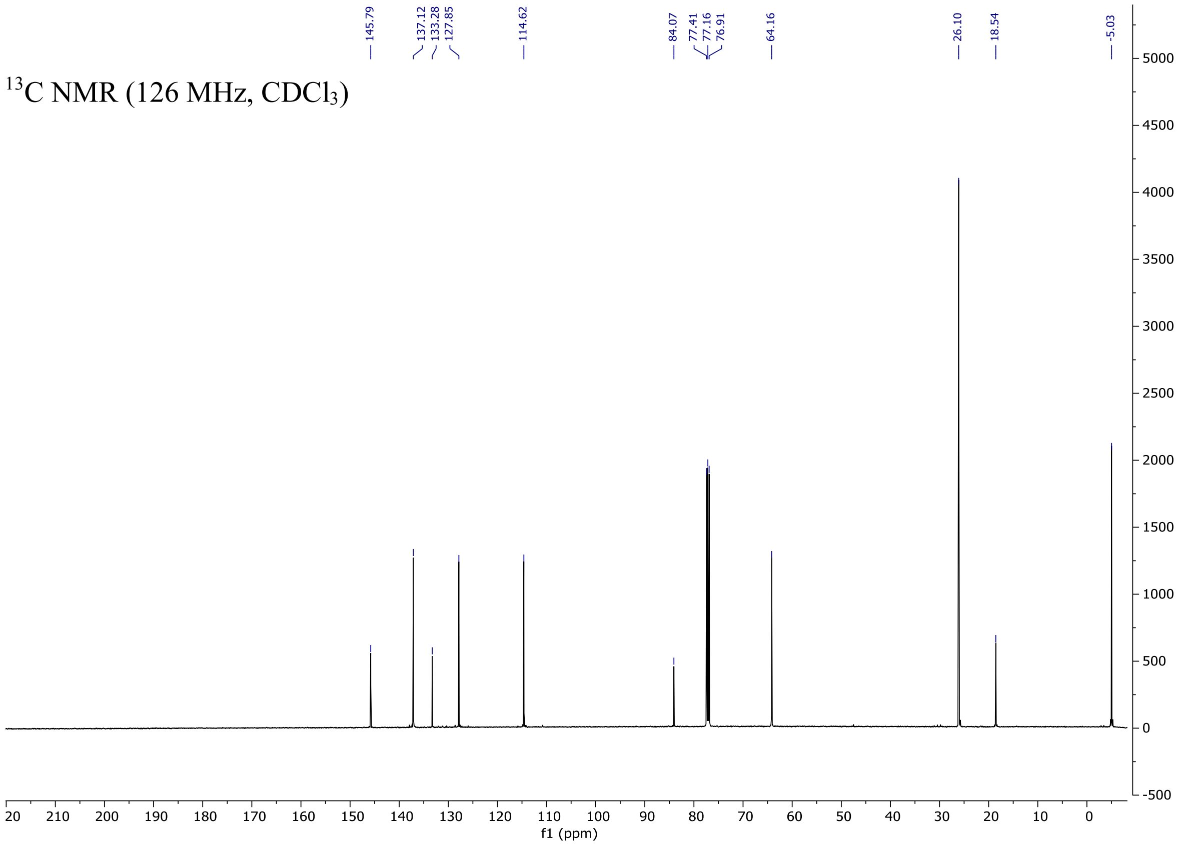

Compound S13:

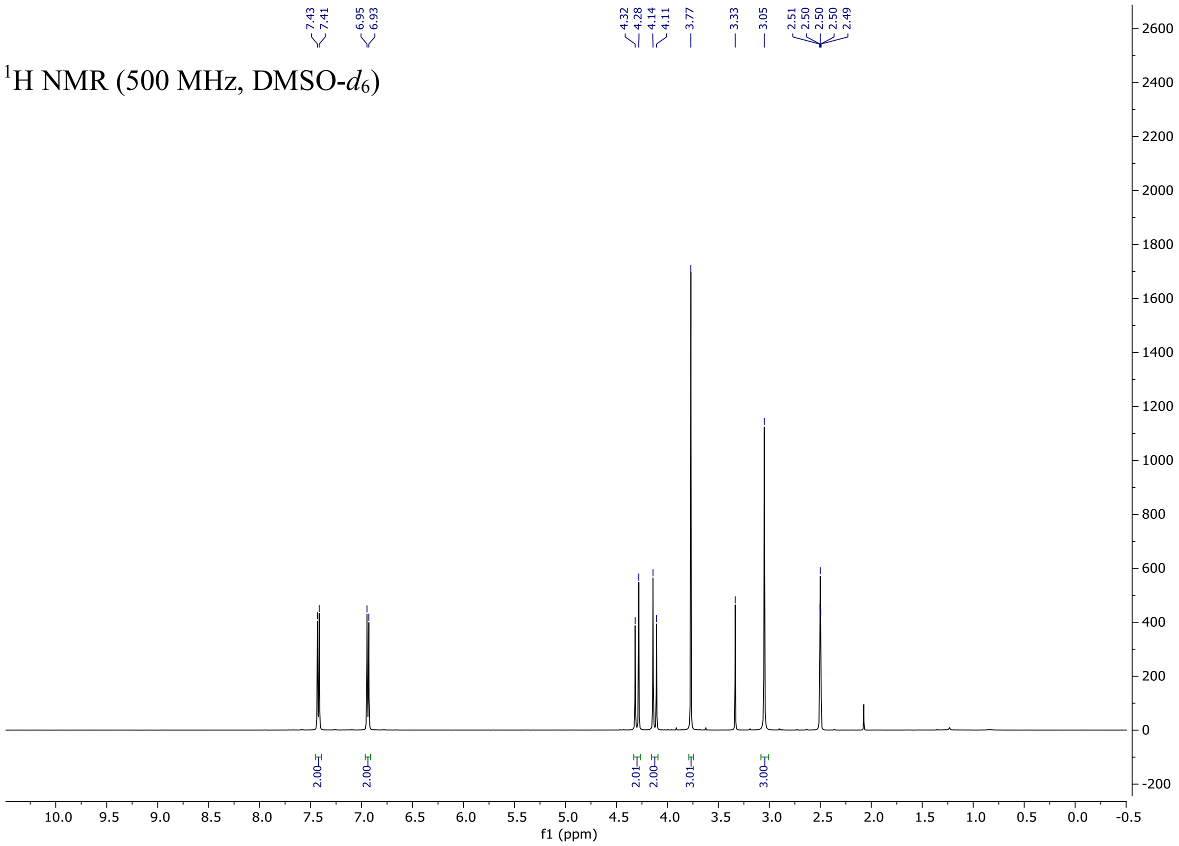

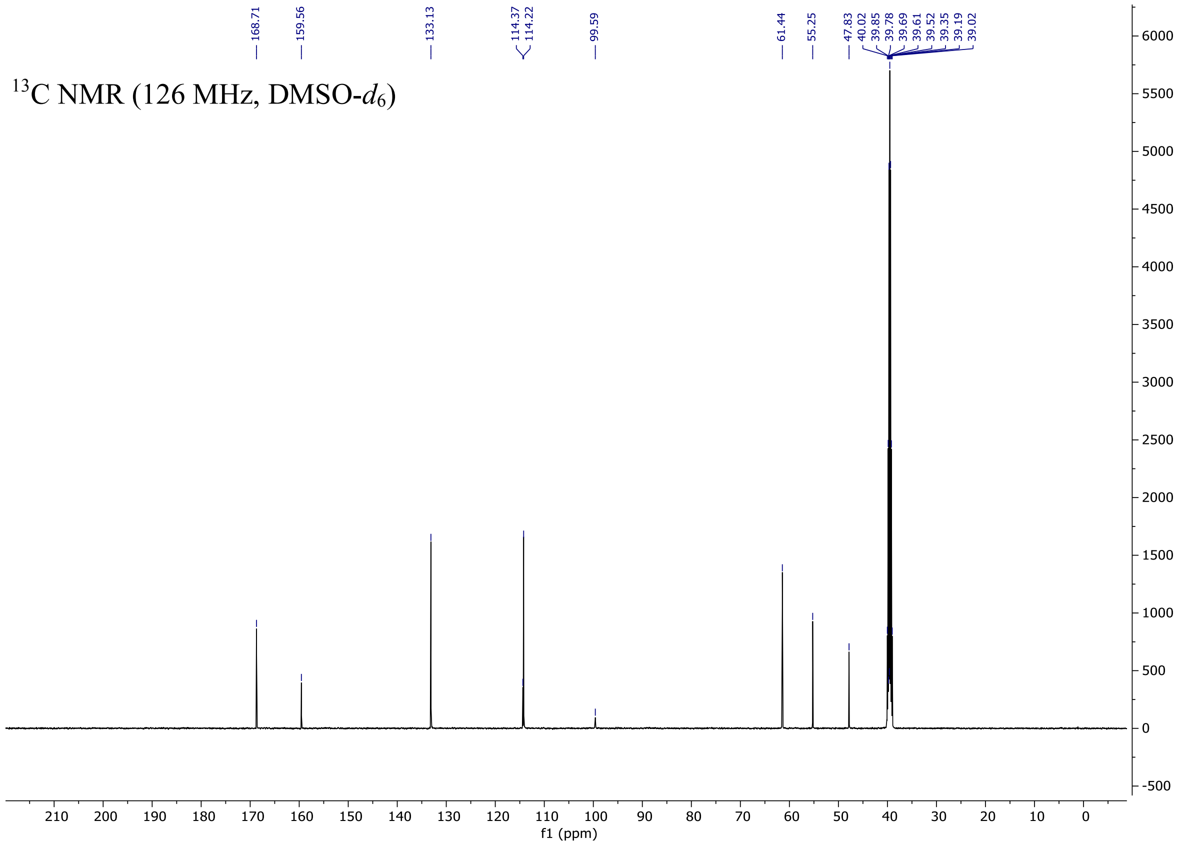

Compound S14:

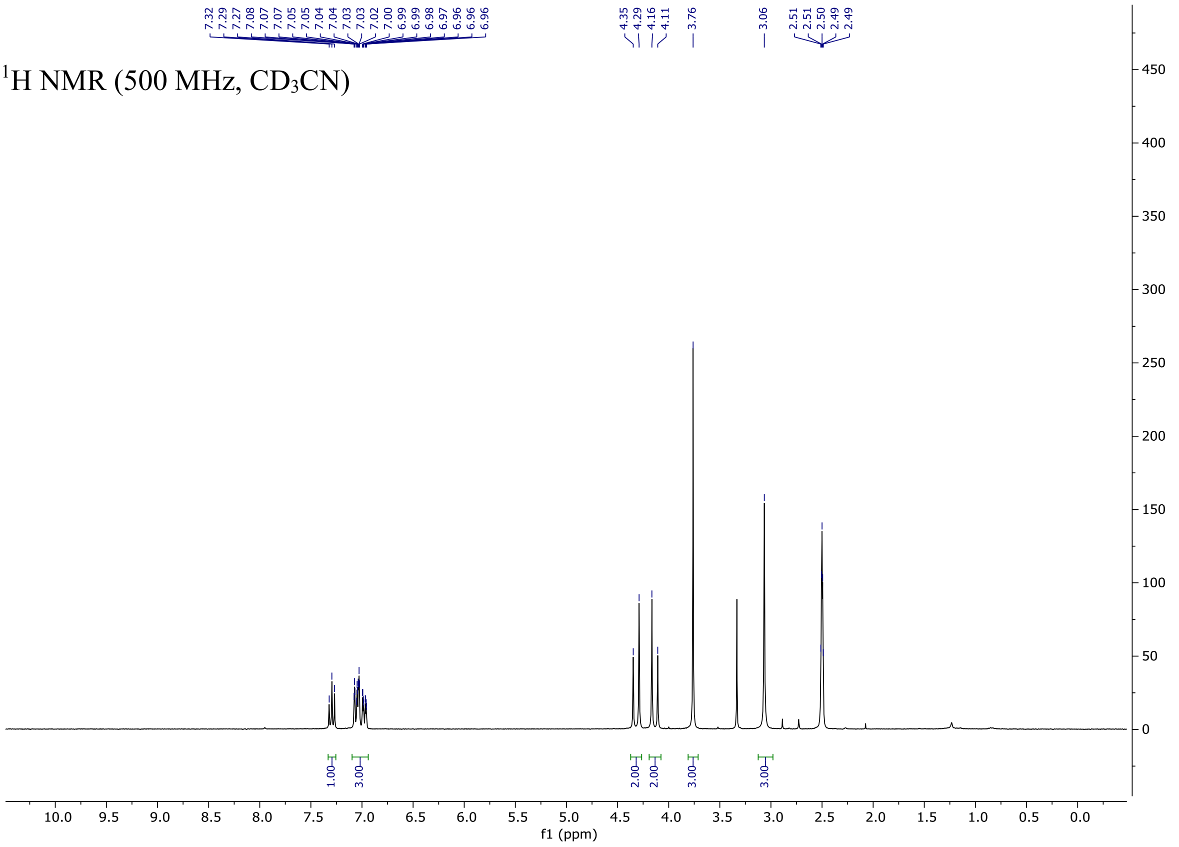

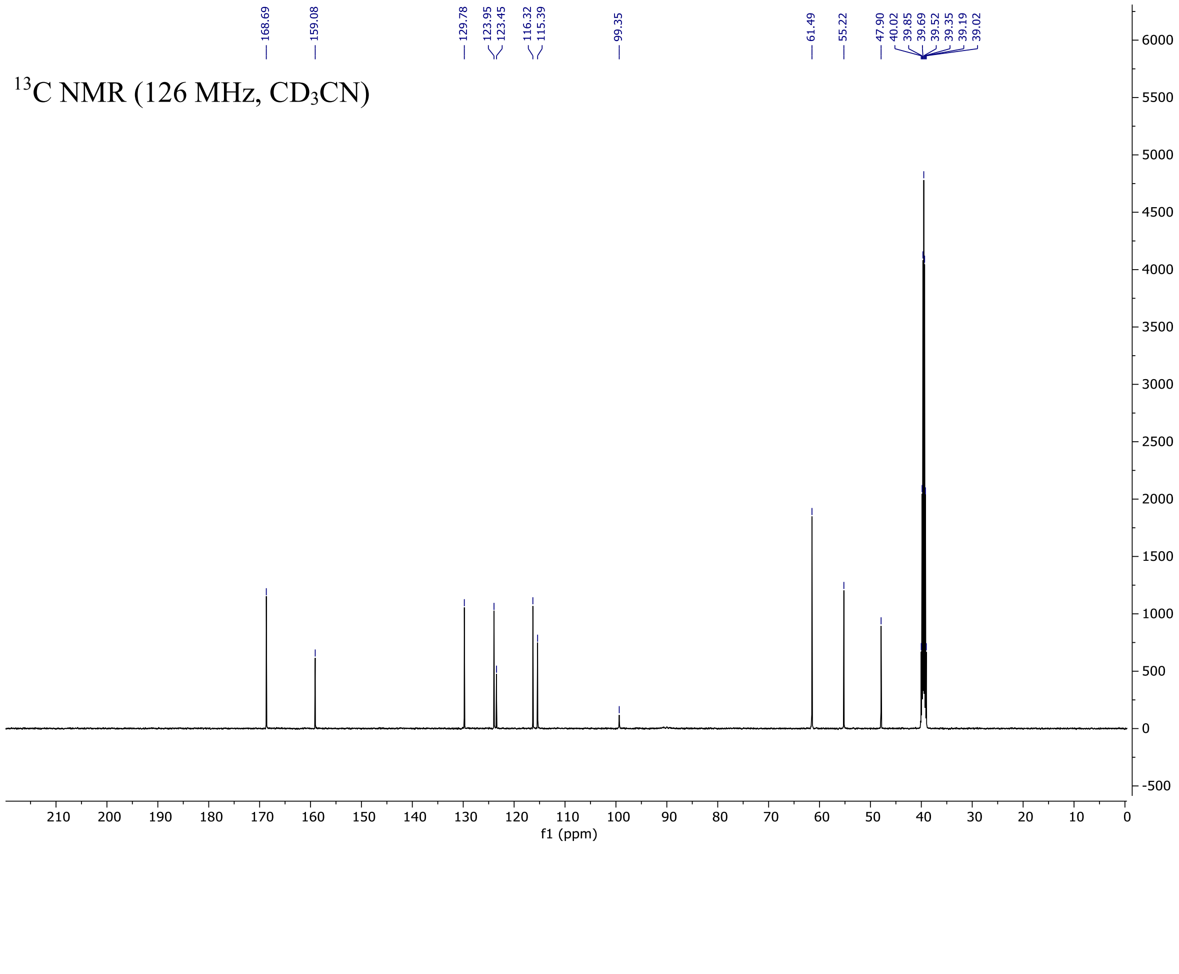

Compound S15:

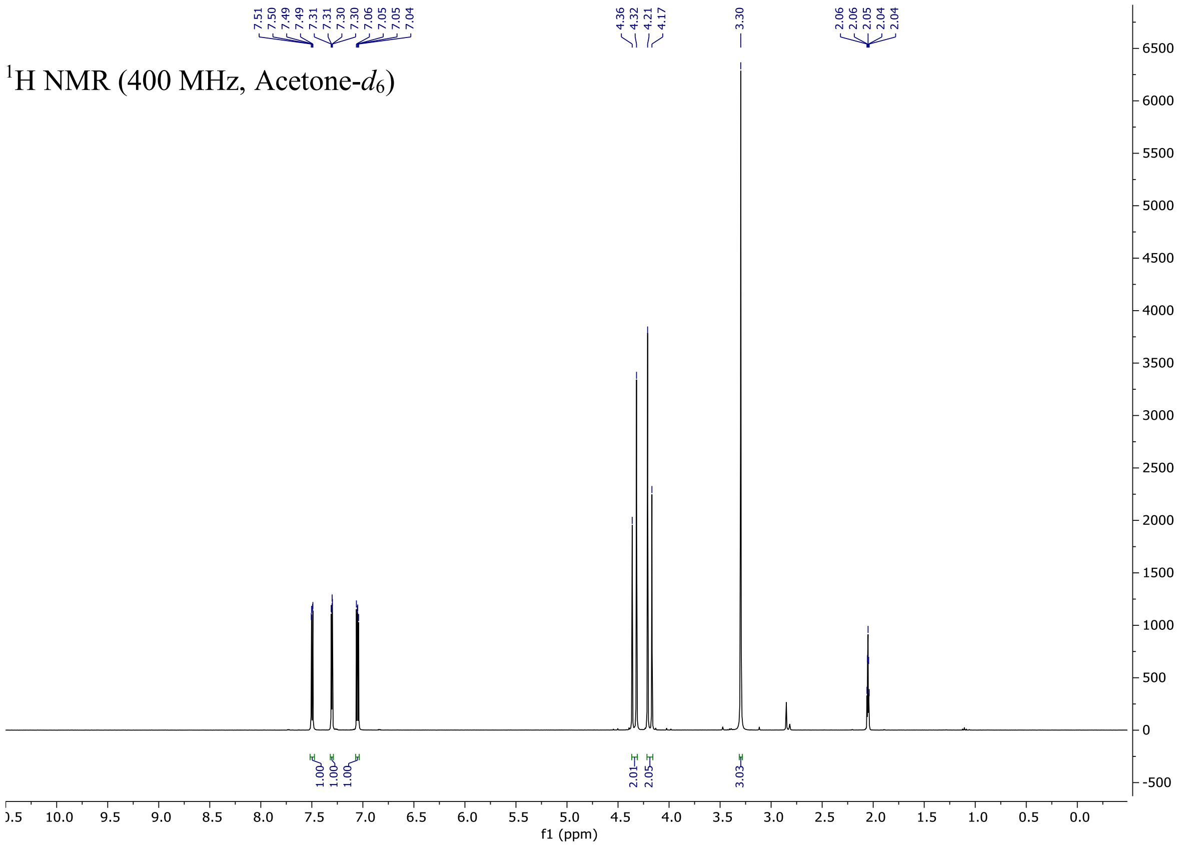

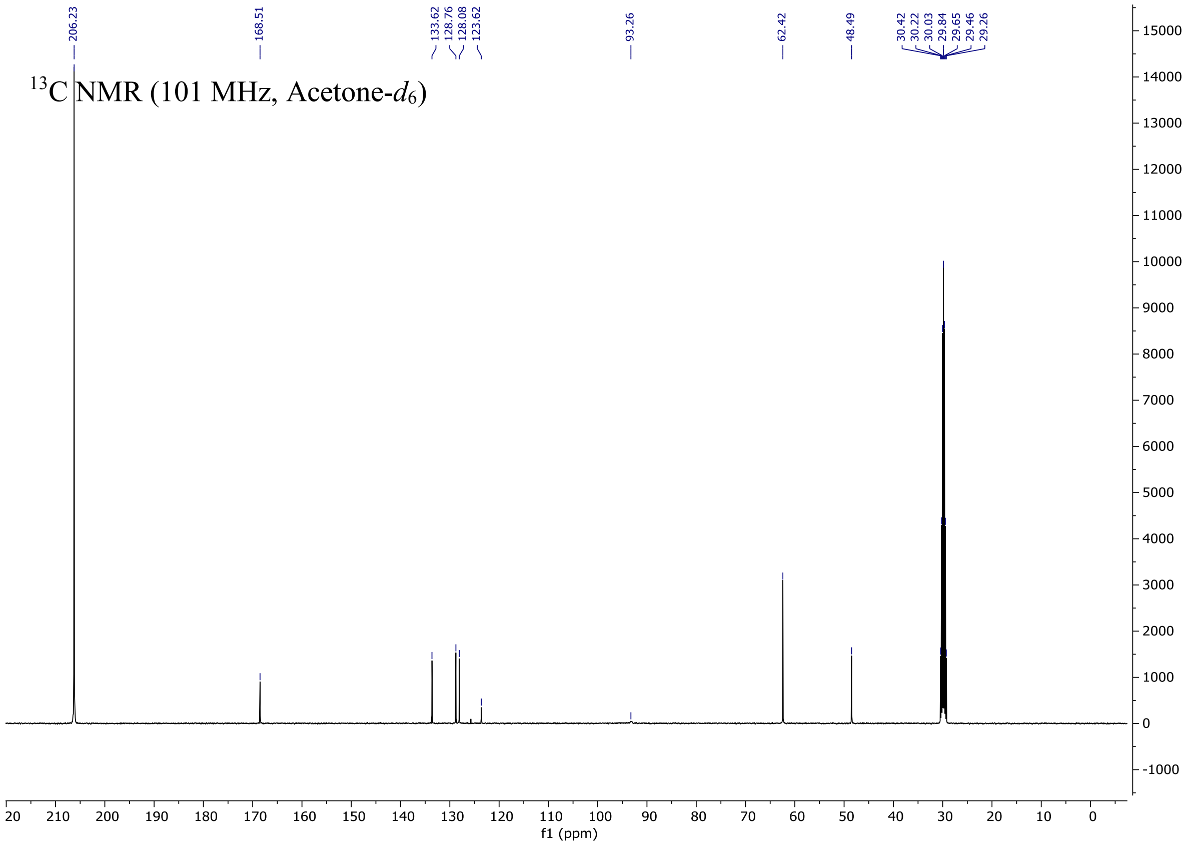

Compound S16:

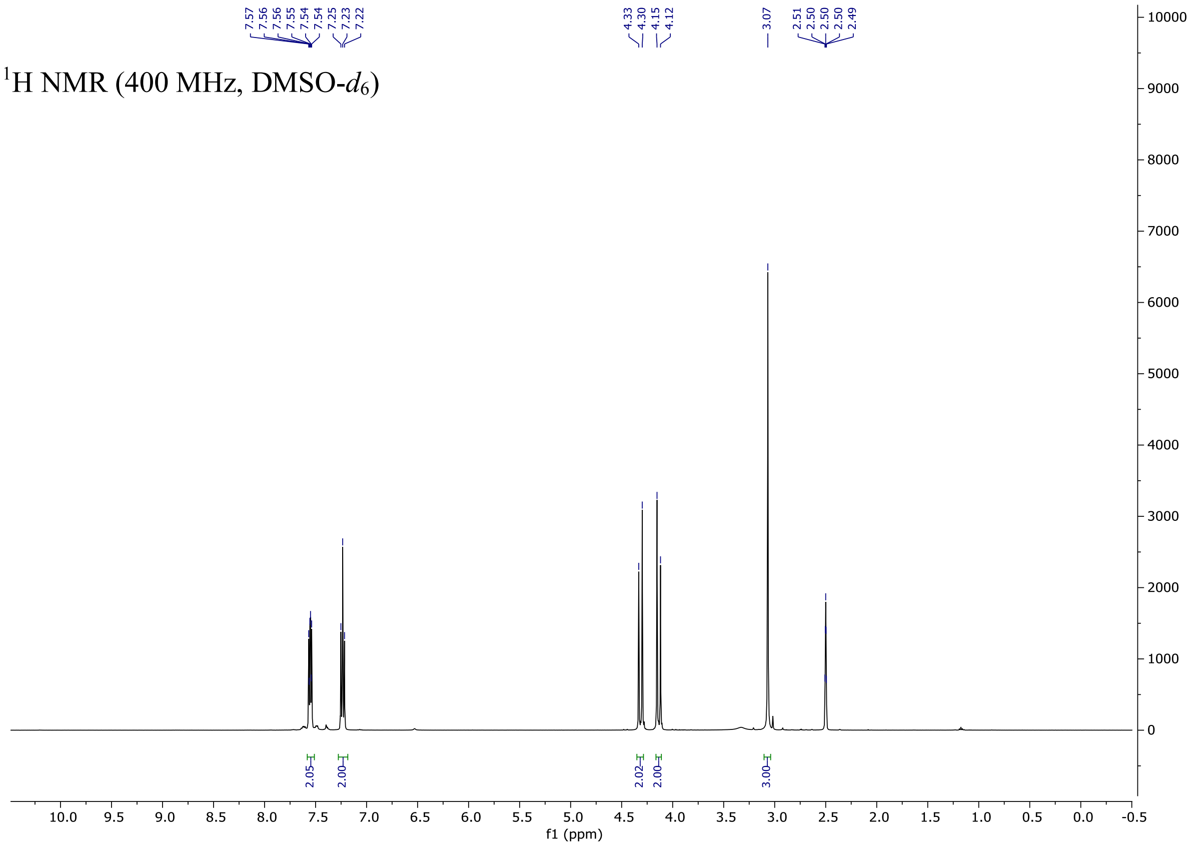

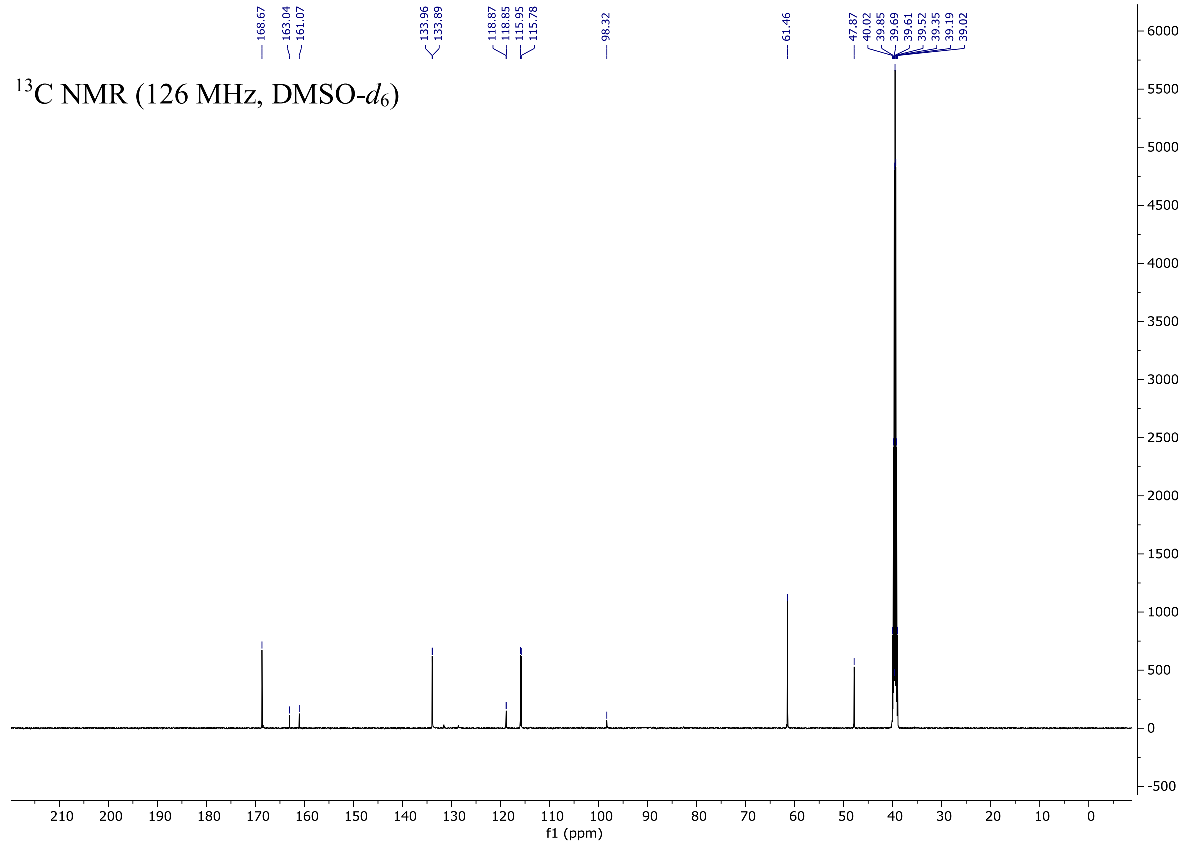

^{19}F NMR (376 MHz, DMSO-d_6)

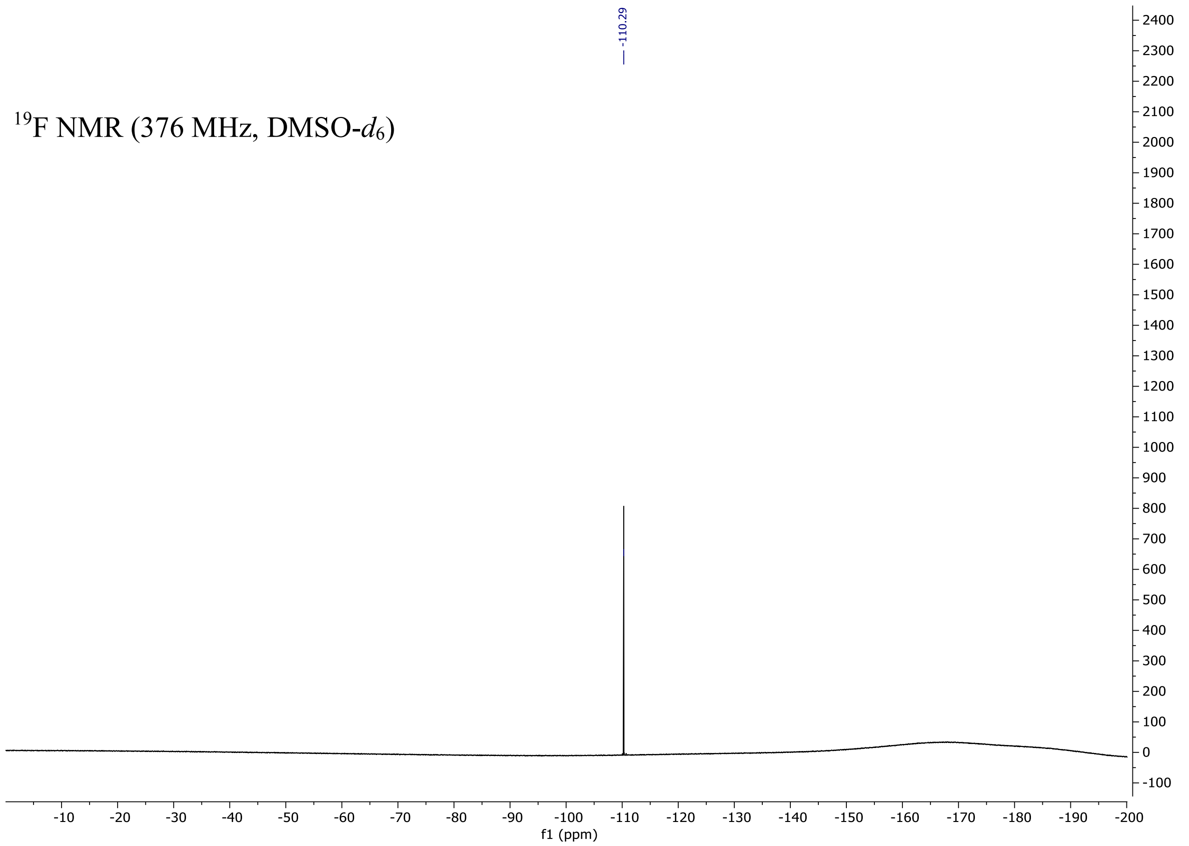

Compound S17:

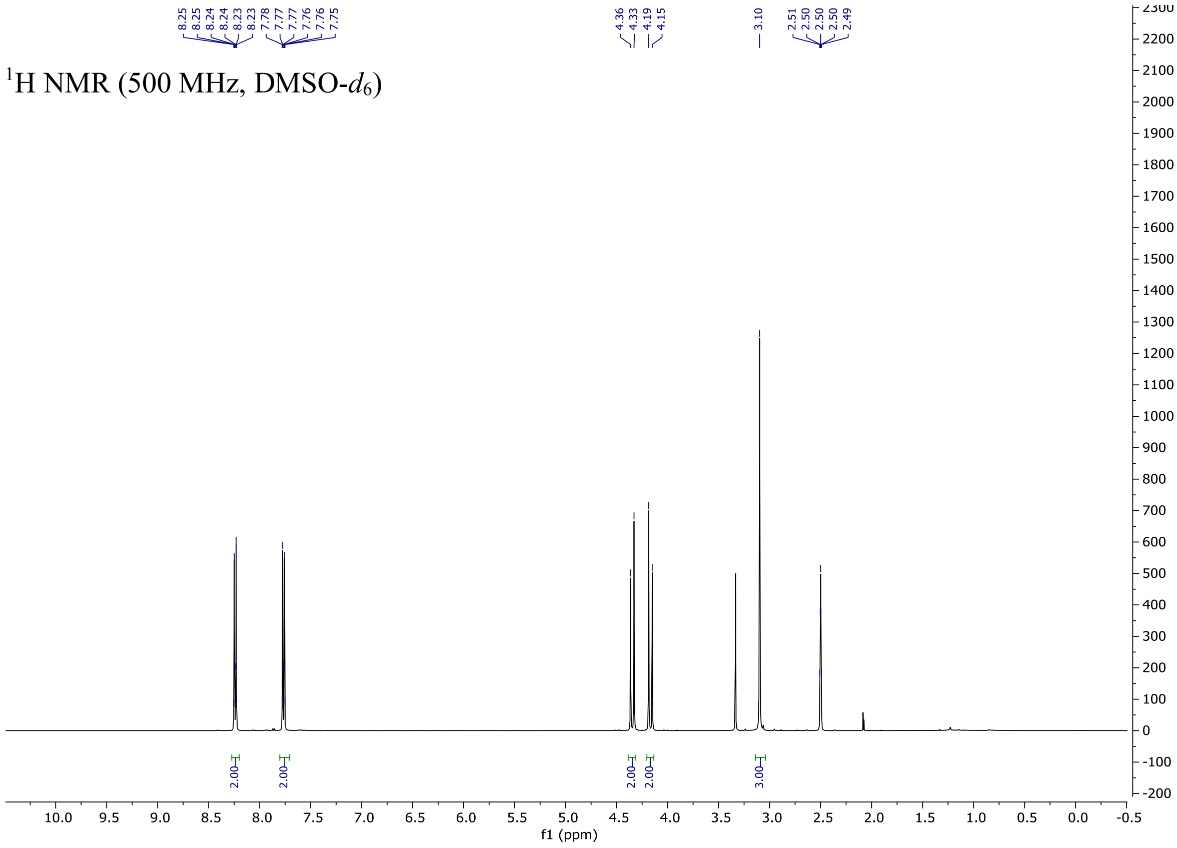

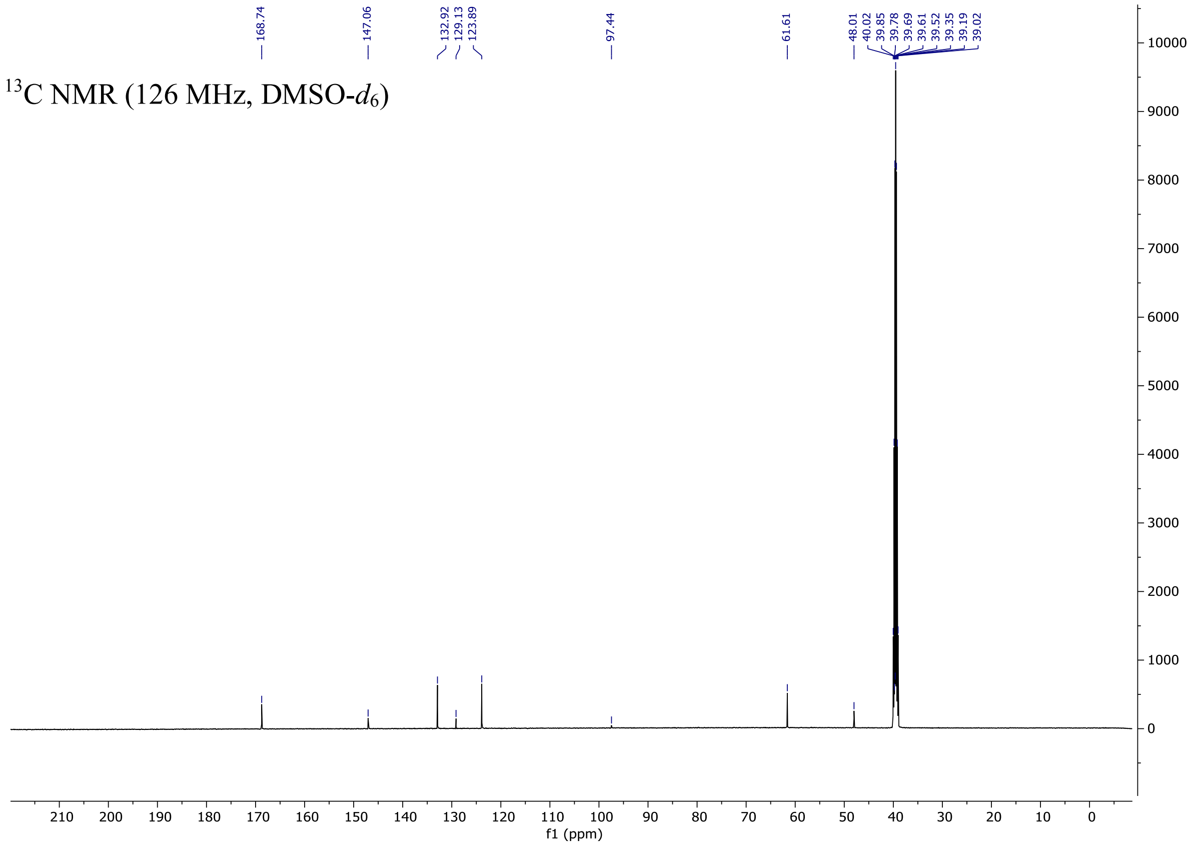

Compound S18:

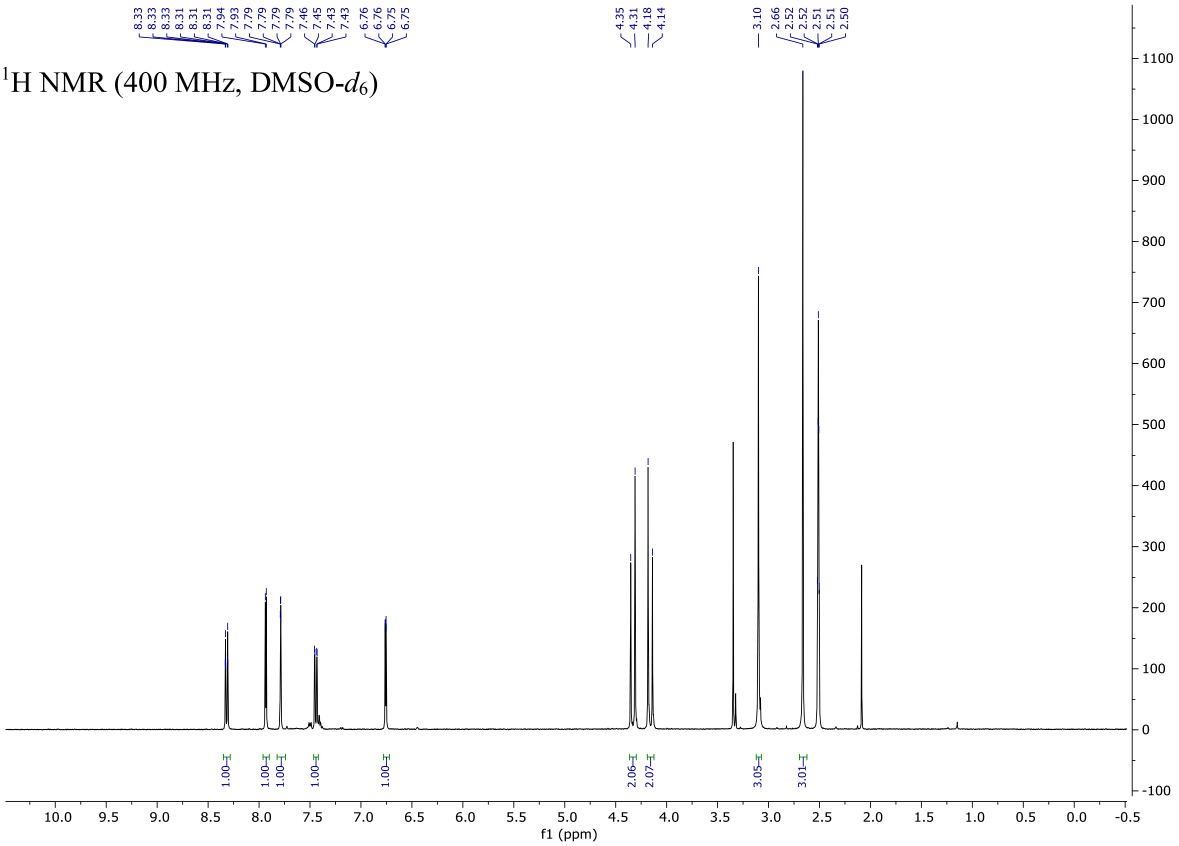

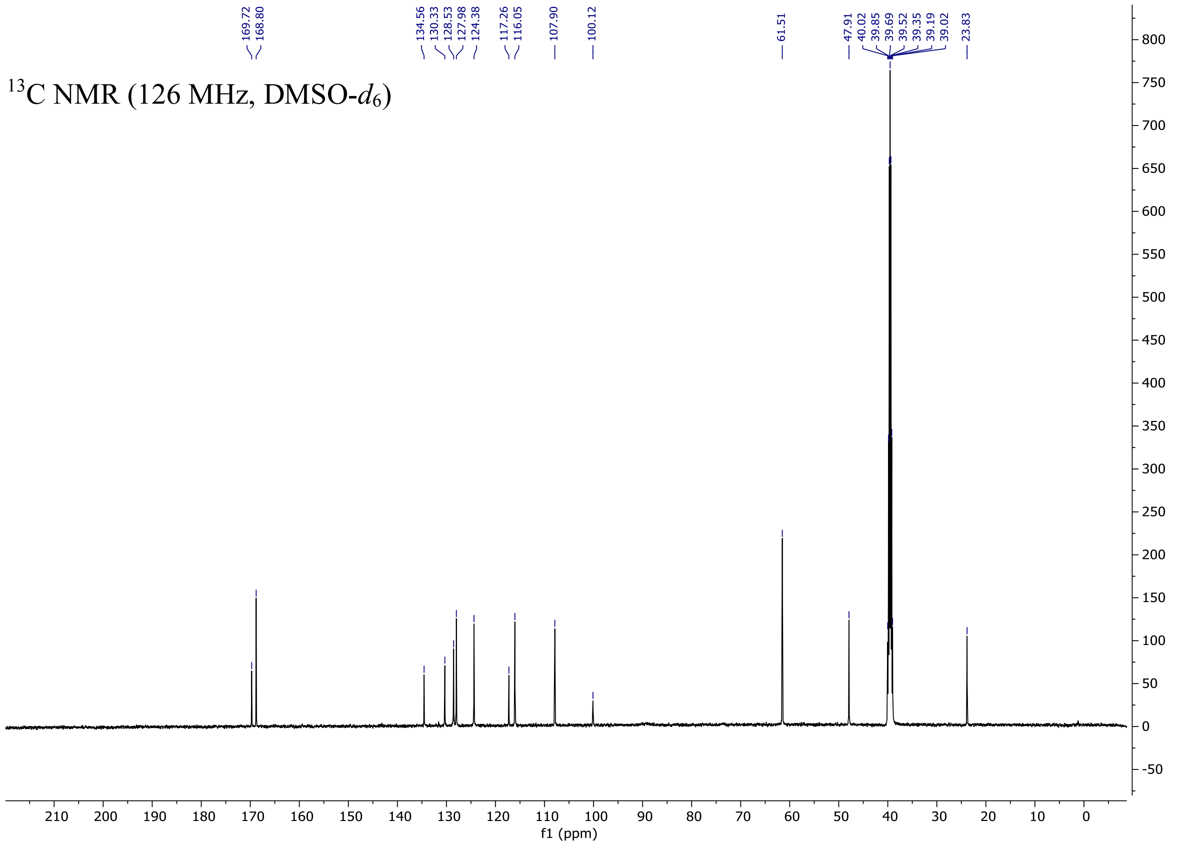

Compound S19:

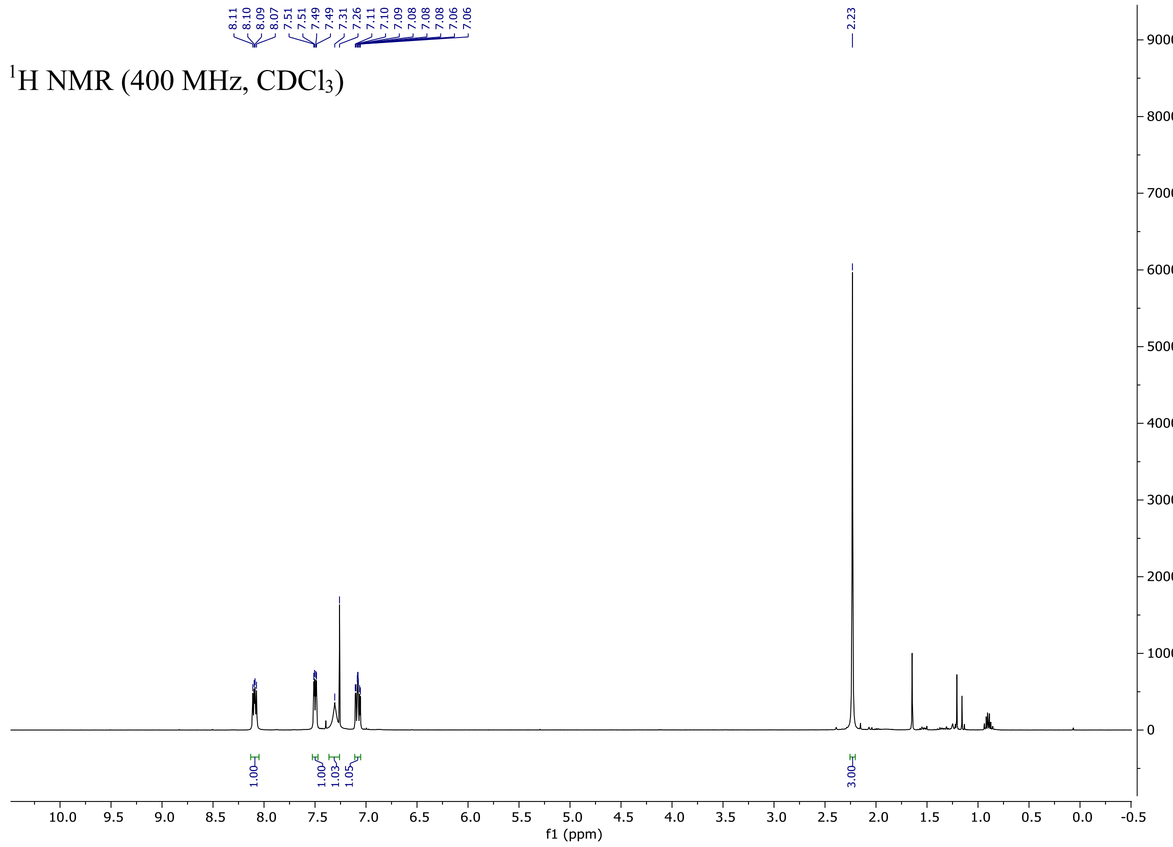

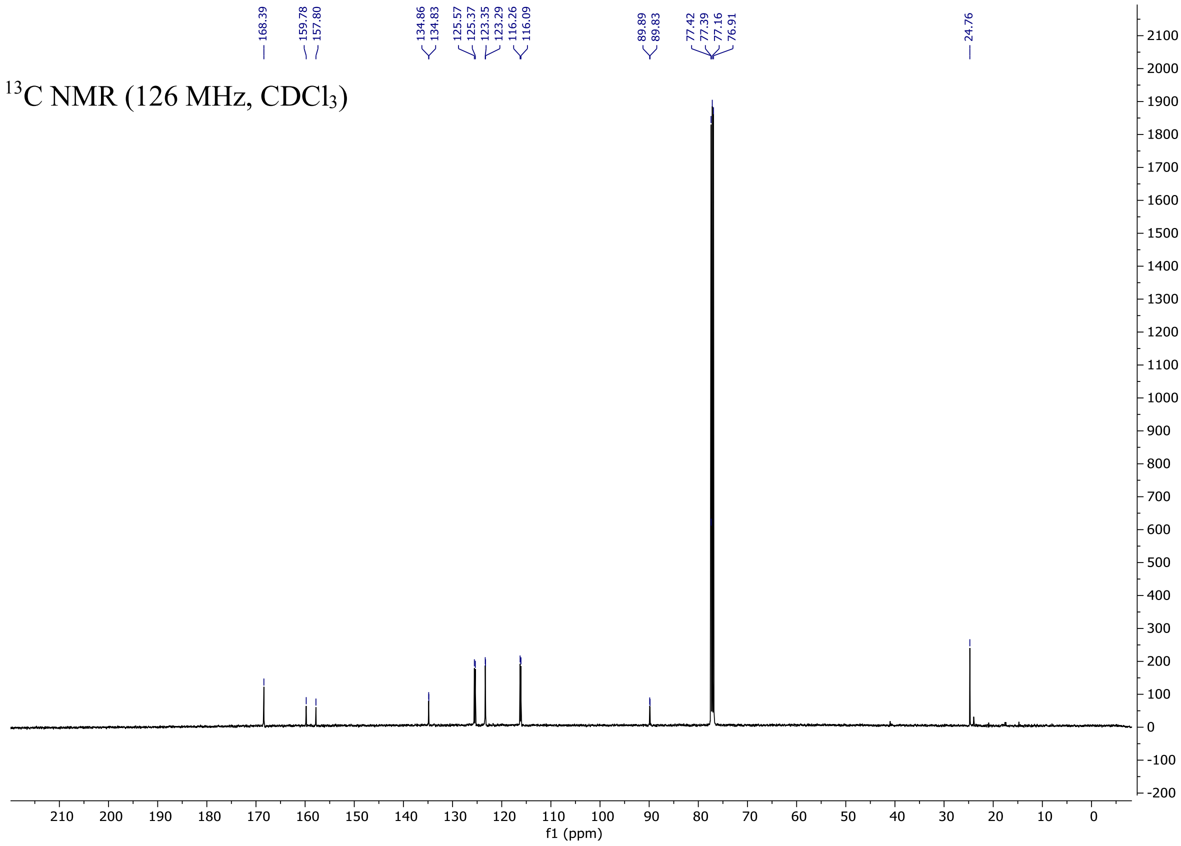

^{19}F NMR (471 MHz, $CDCl_3$)

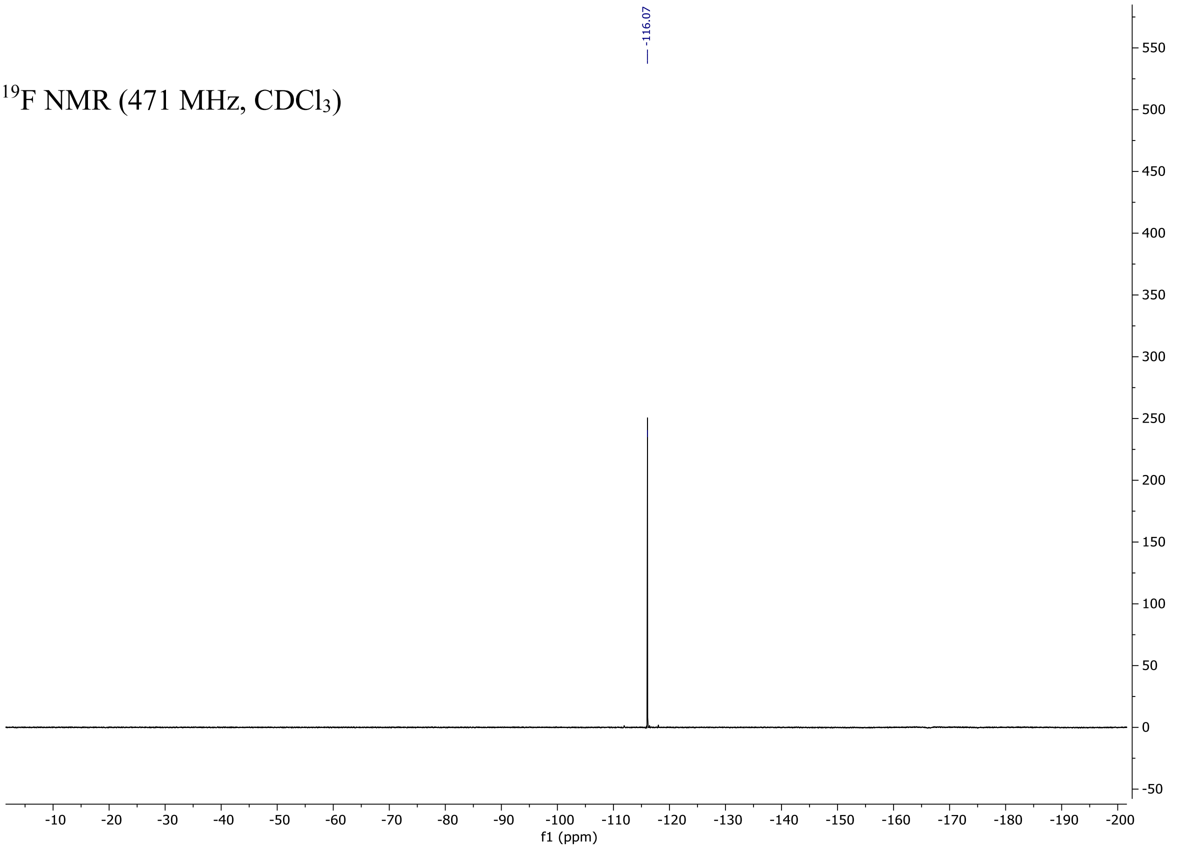

Compound S20:

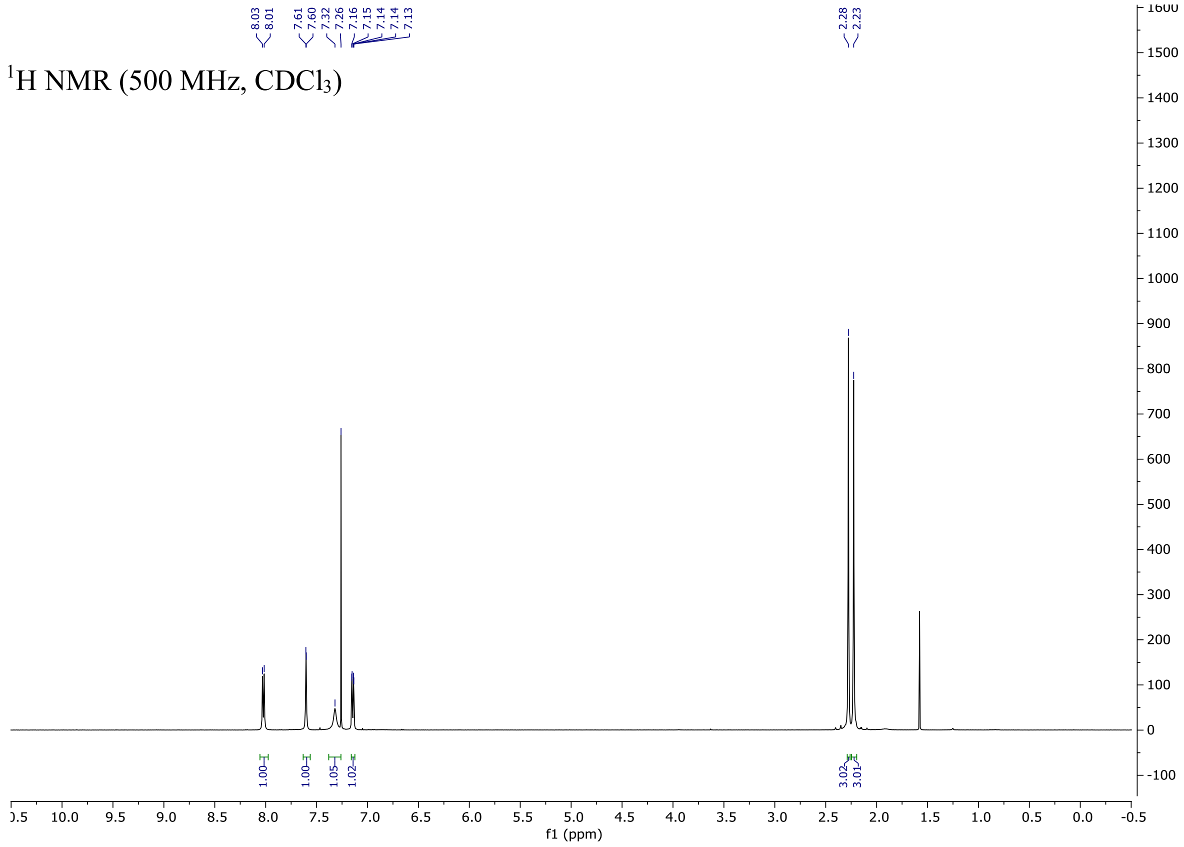

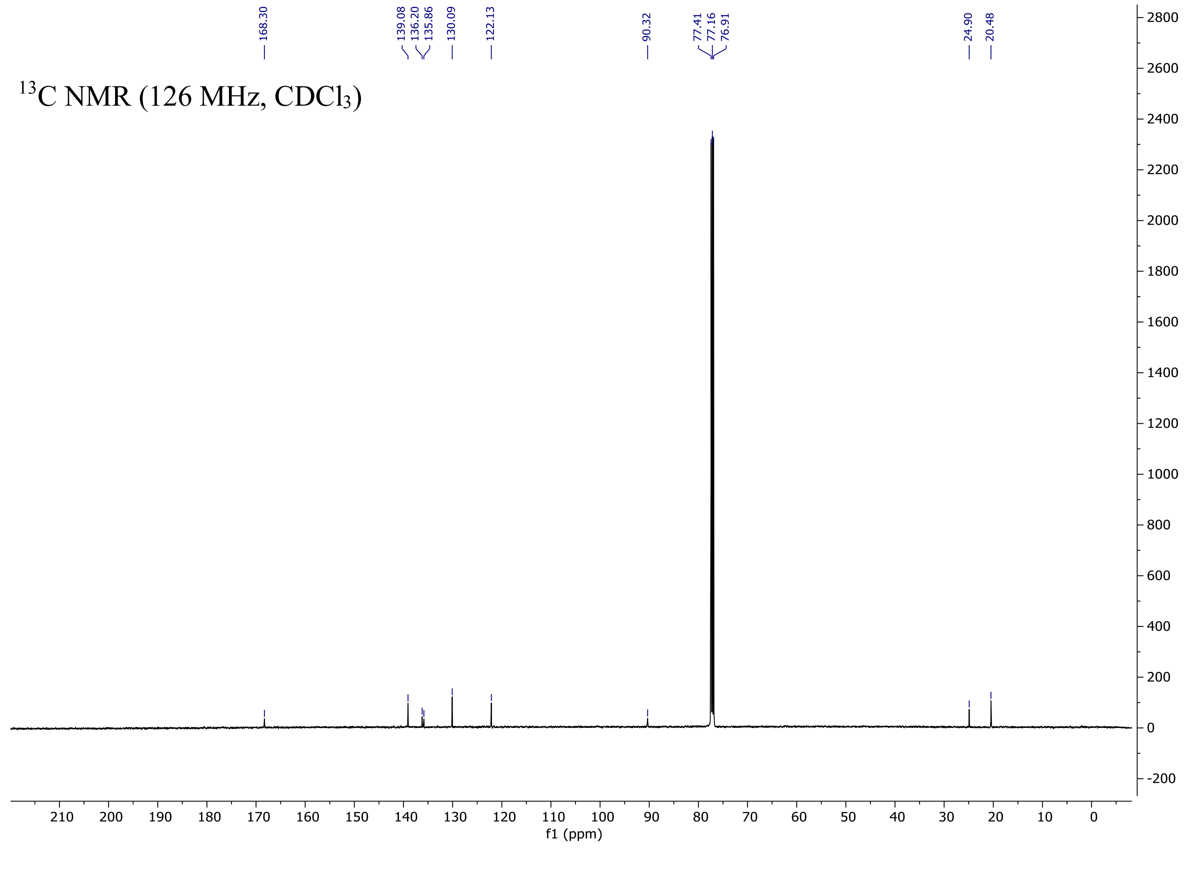

Compound S21:

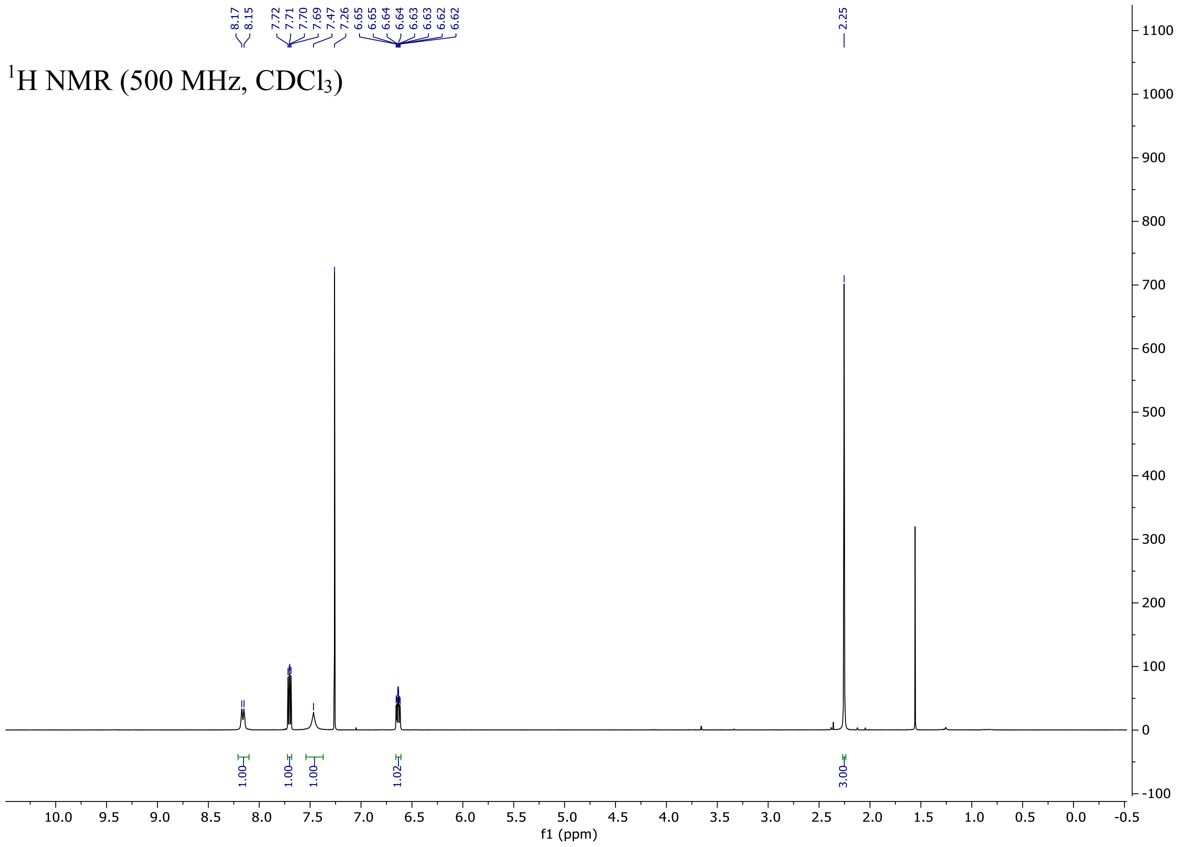

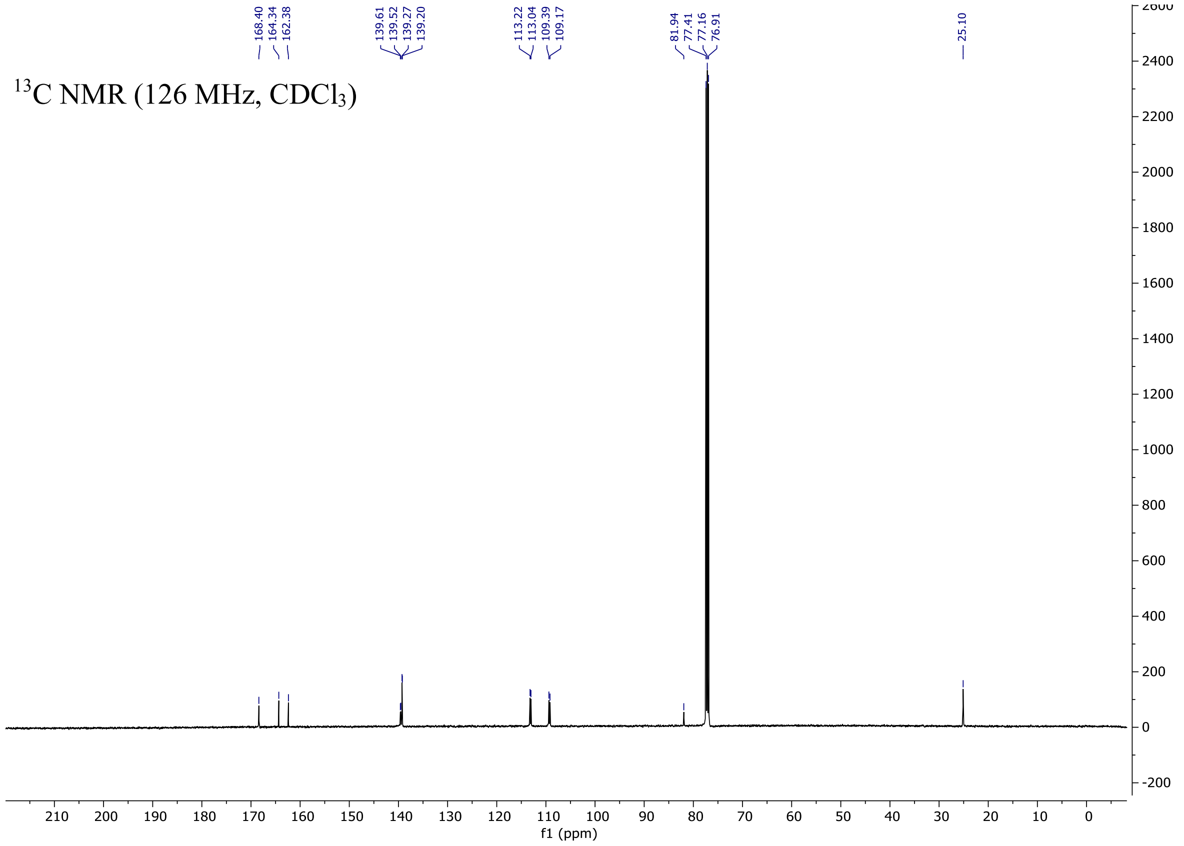

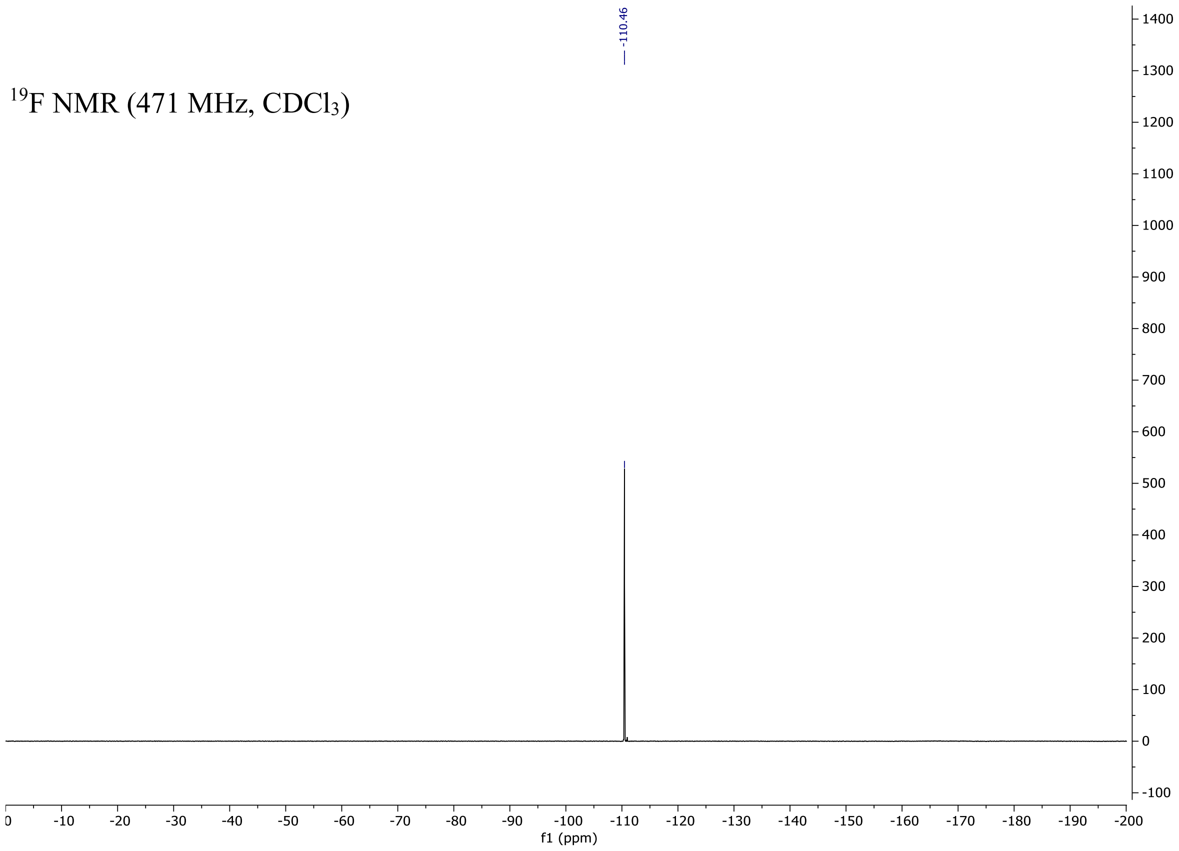

-110.46
19F NMR (471 MHz, CDCl3)
1400
1300
1200
1100
1000
900
800
700
600
500
400
300
200
100
0
-100
0
-10
-20
-30
-40
-50
-60
-70
-80
-90
-100
-110
-120
-130
-140
-150
-160
-170
-180
-190
-200
f1 (ppm)

Compound S22:

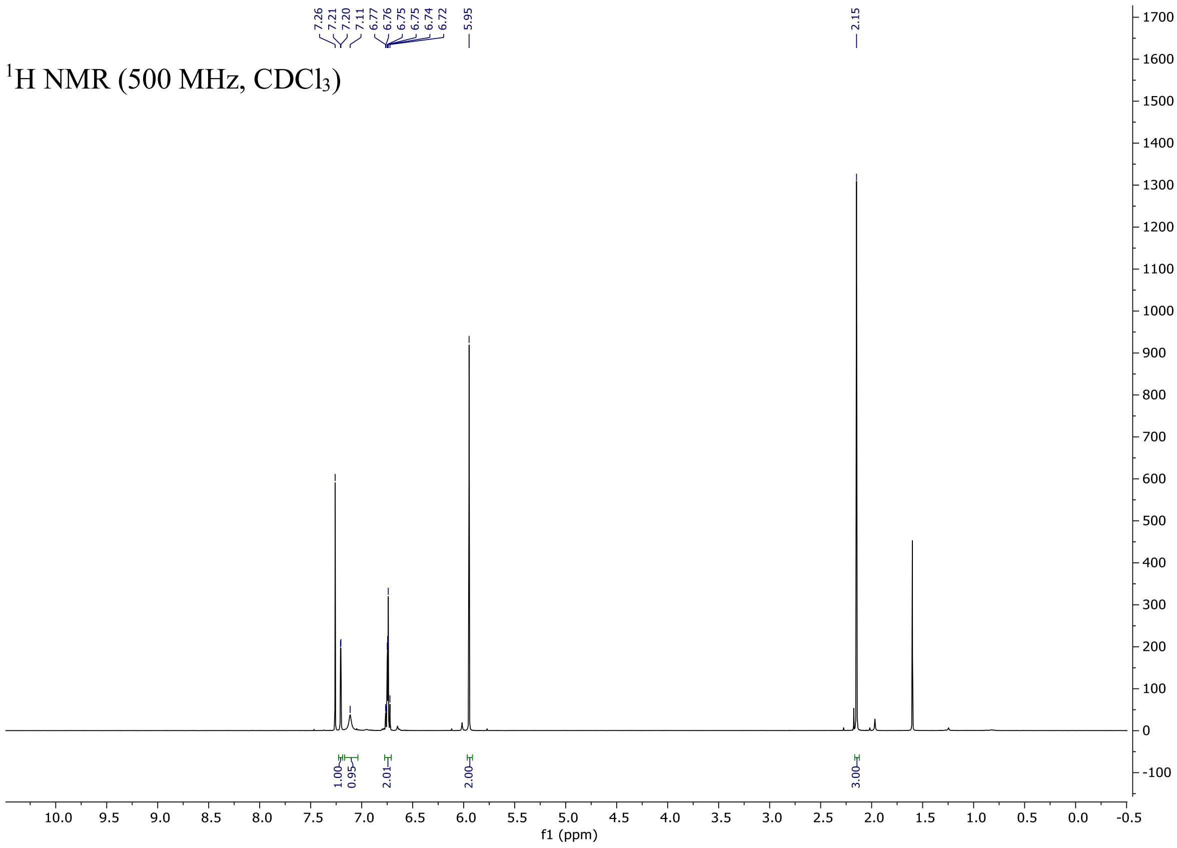

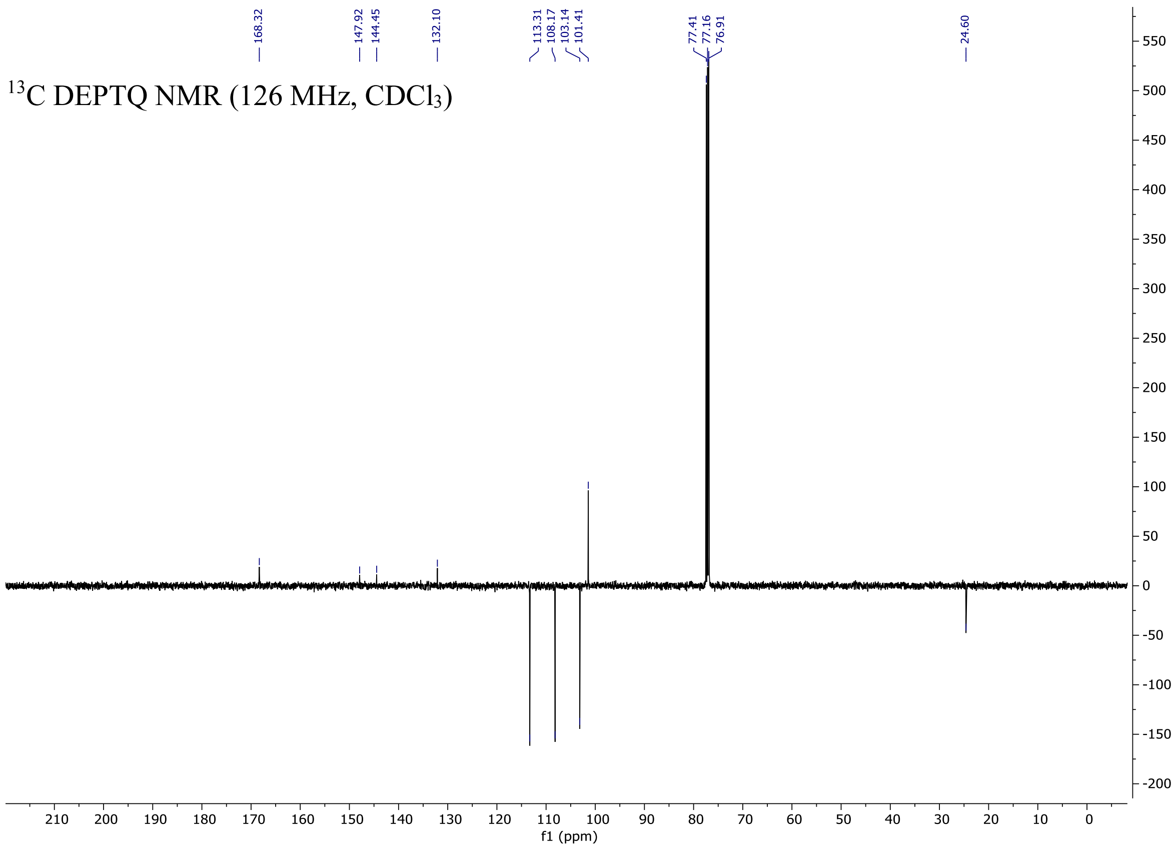

Compound S23

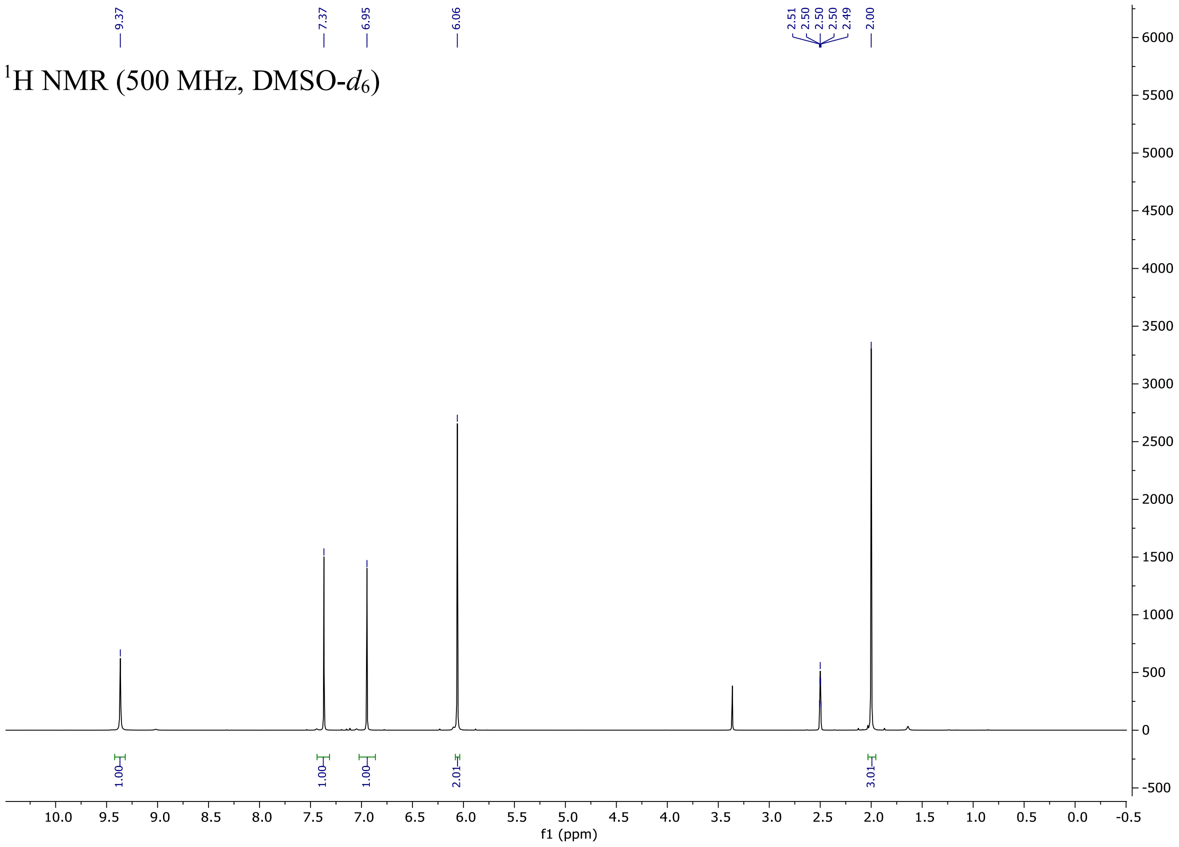

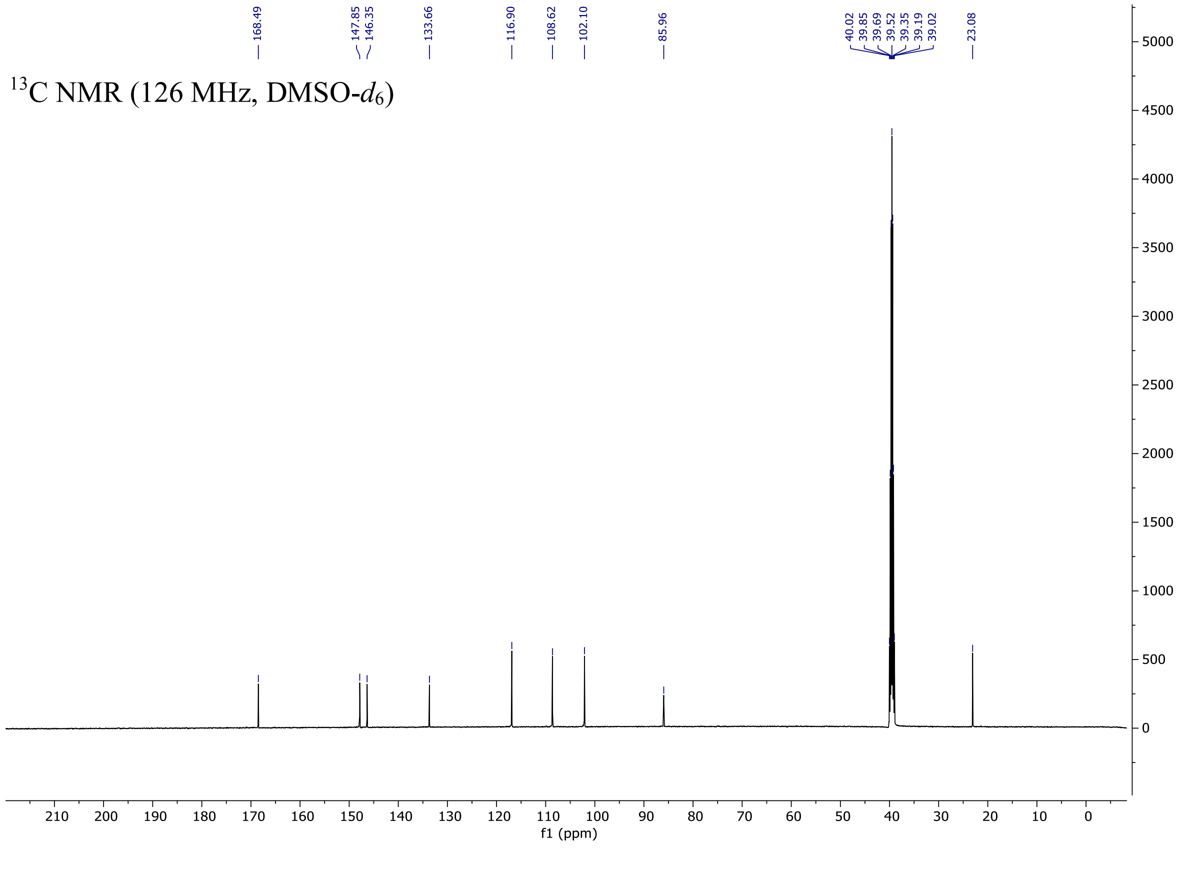

Compound S24:

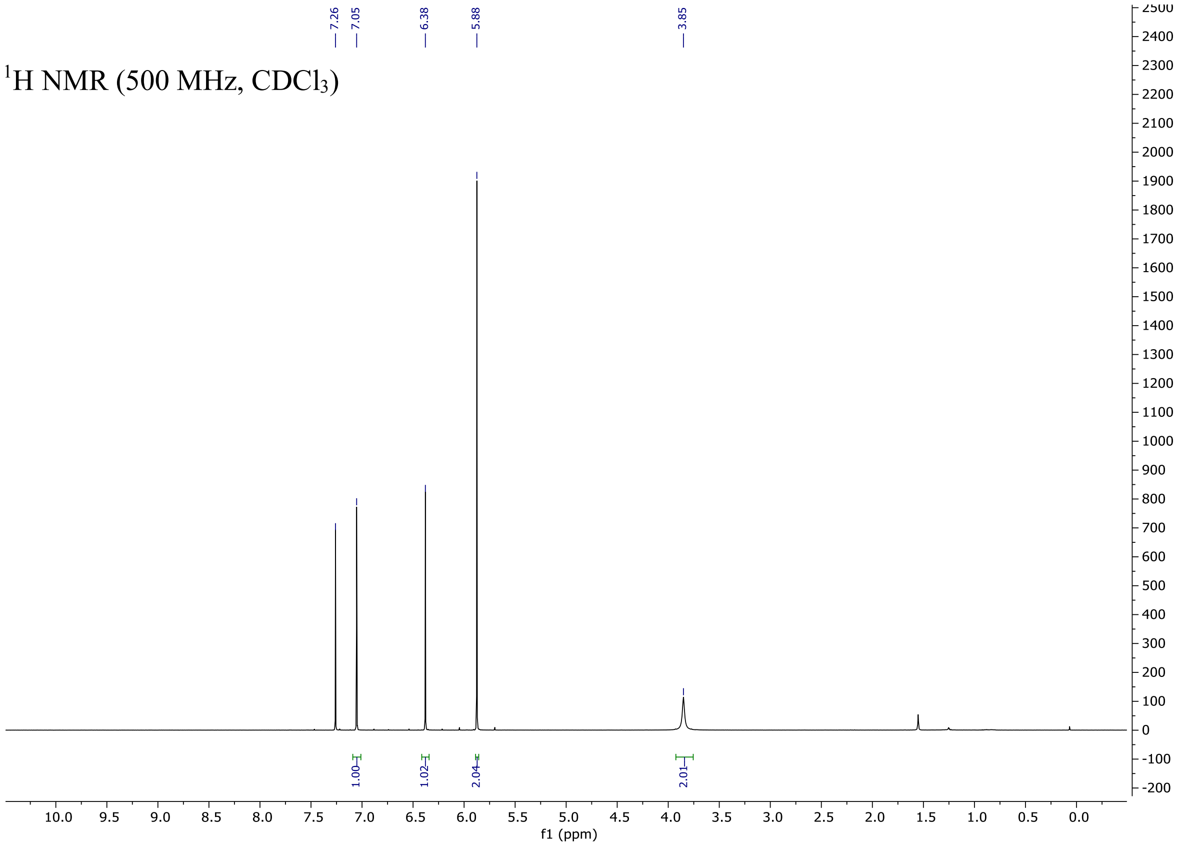

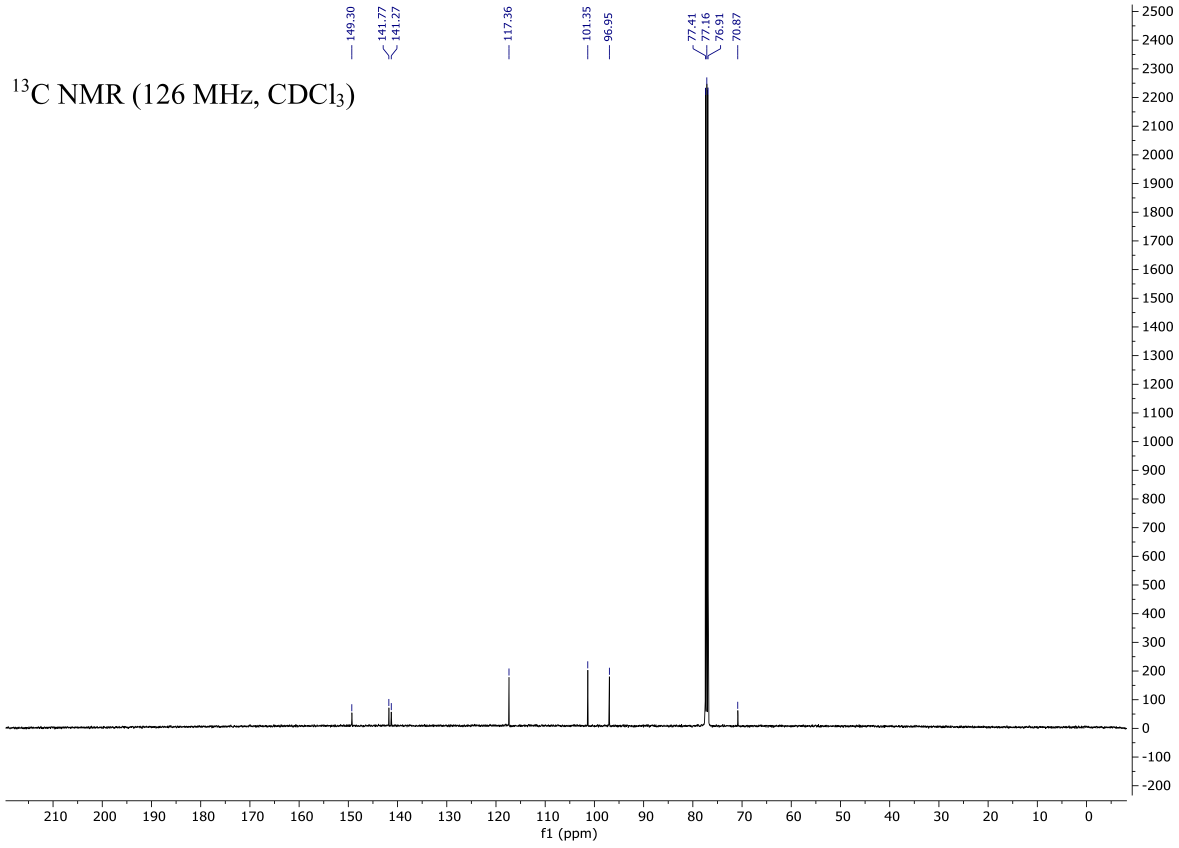

Compound S25

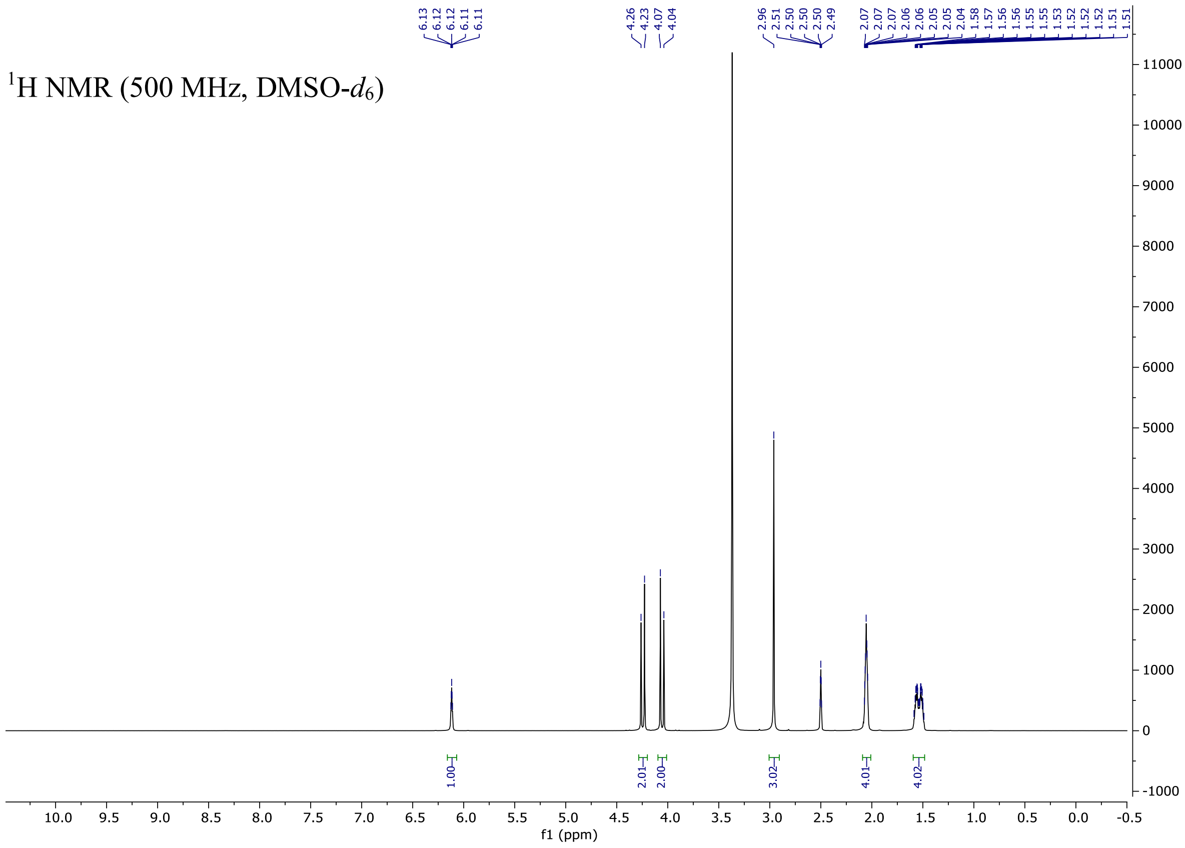

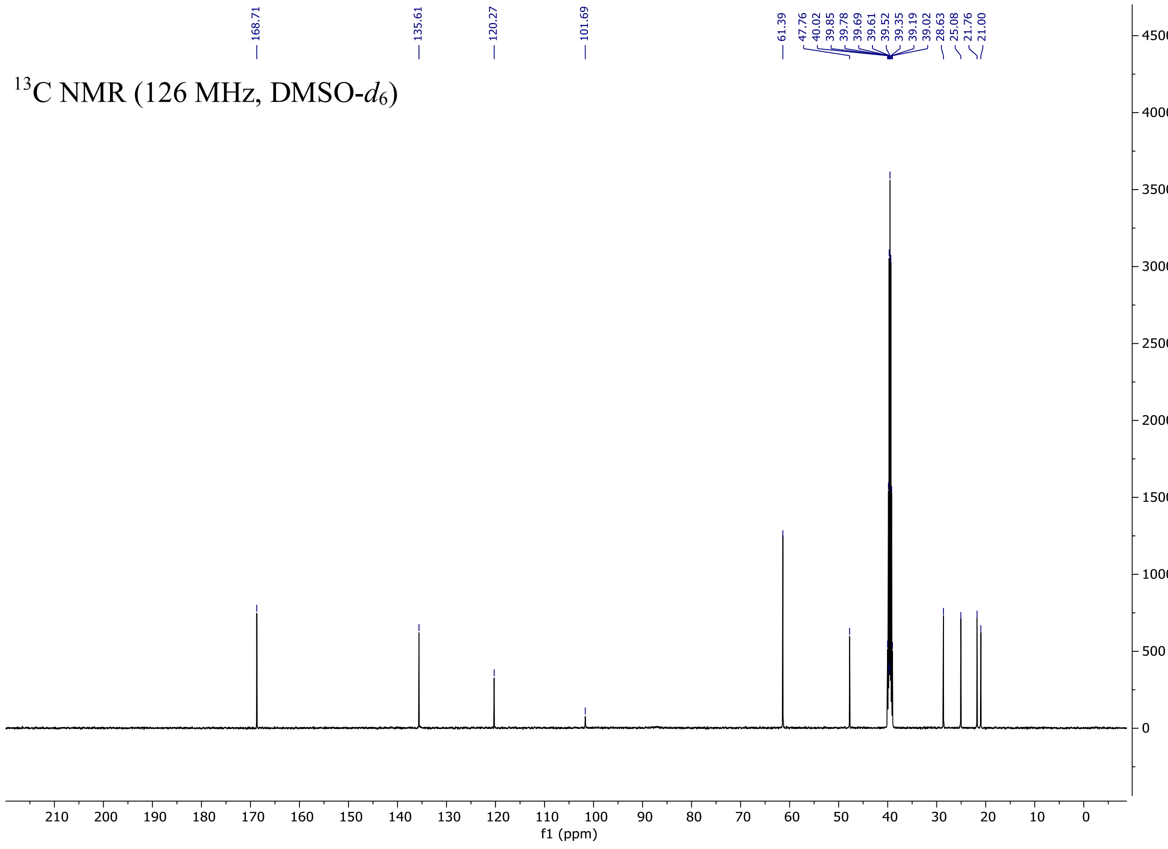

Compound S26:

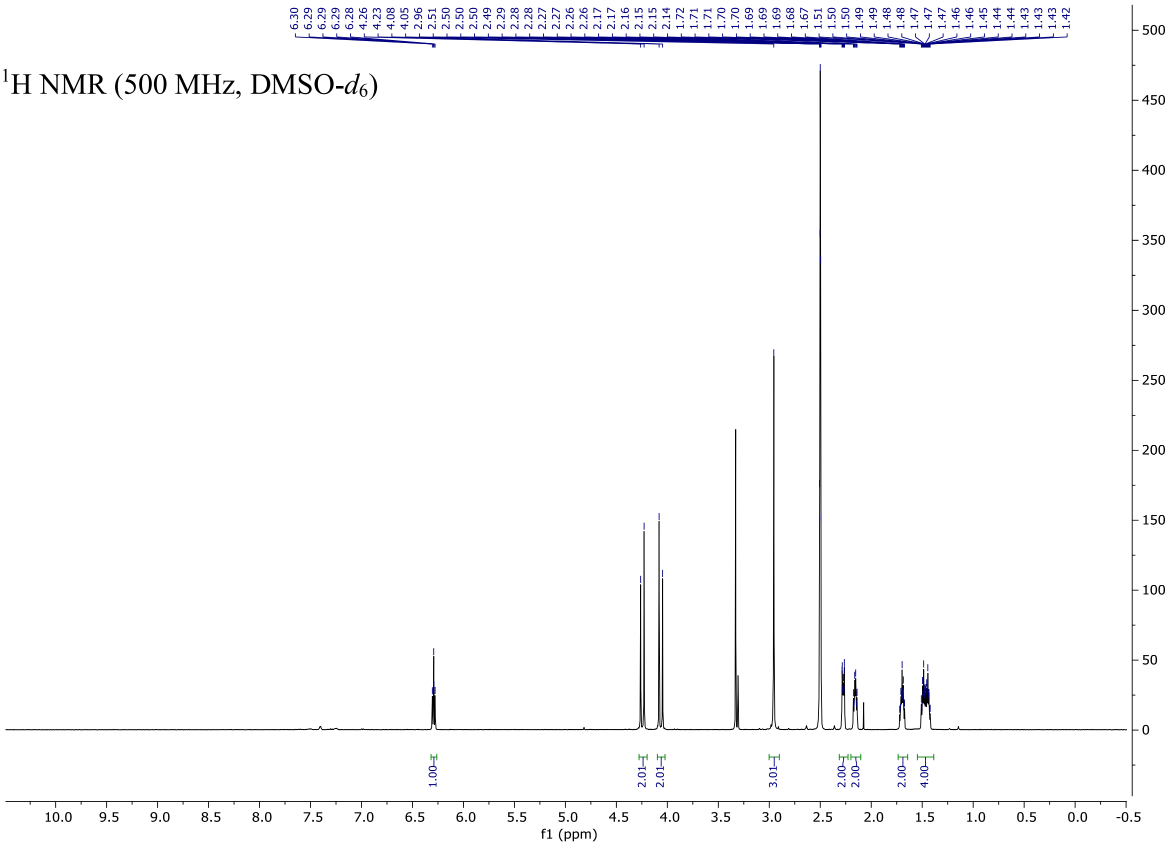

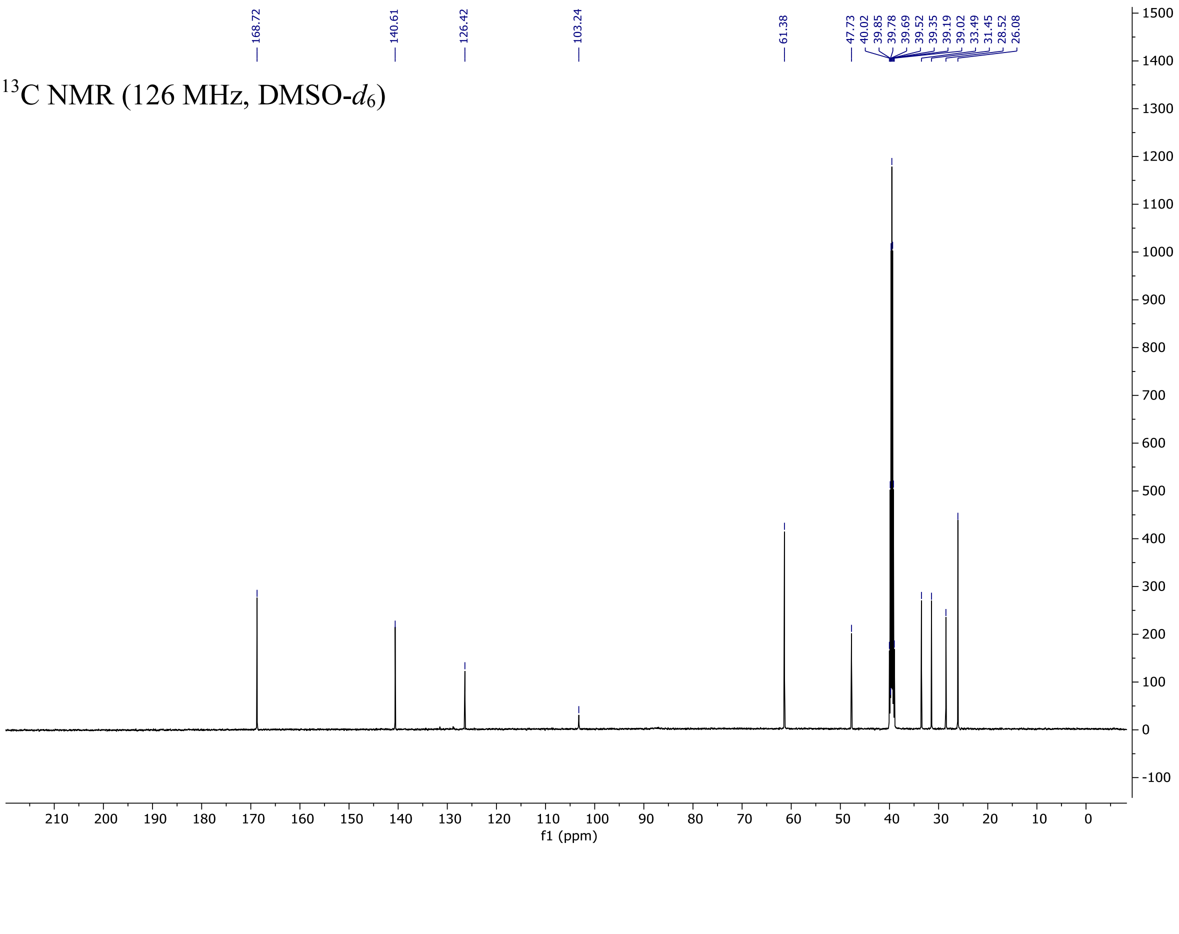

Compound S27:

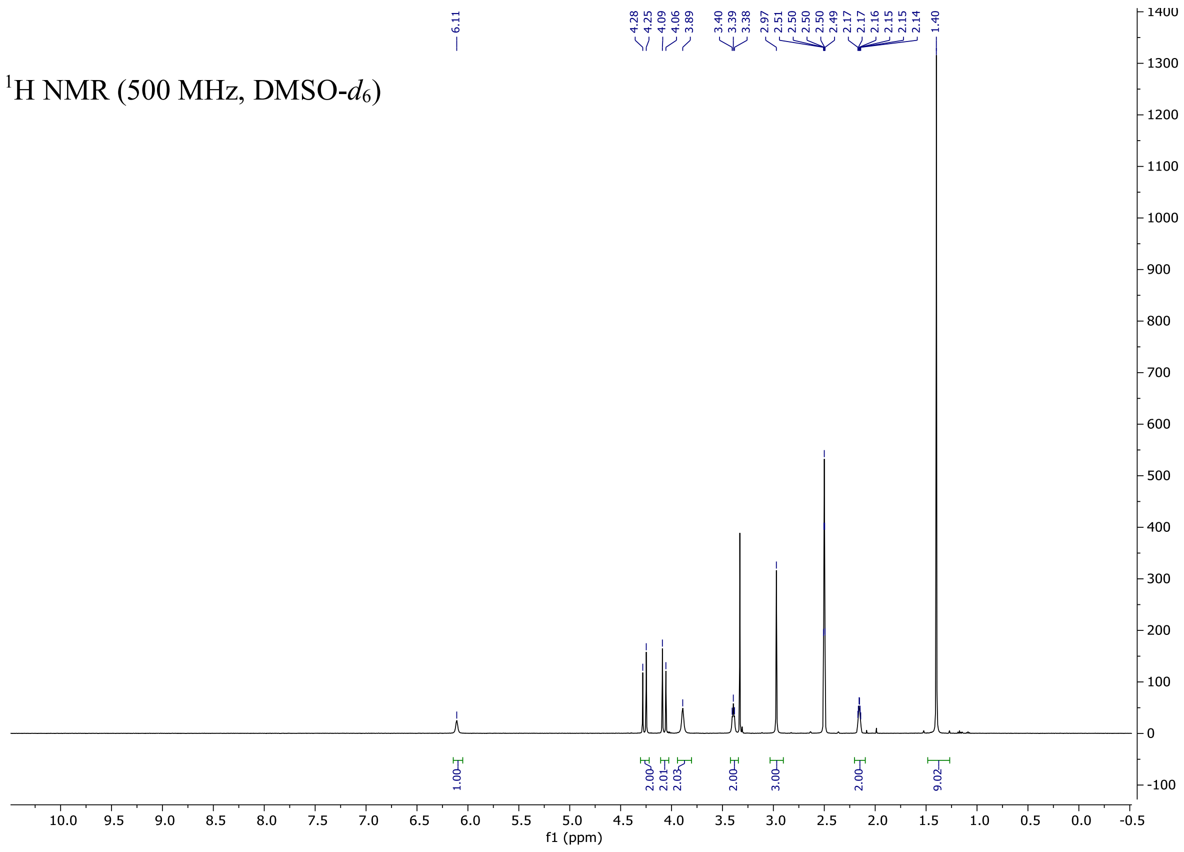

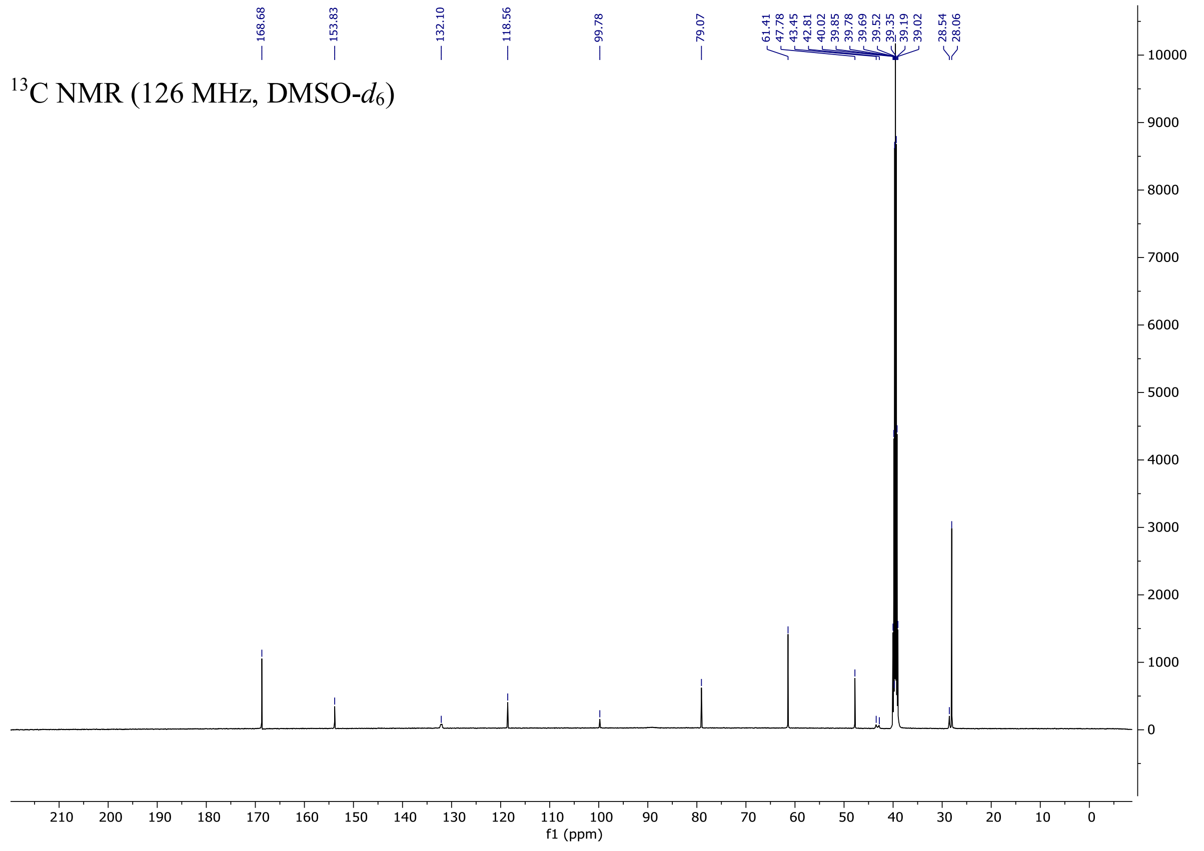

Compound S28:

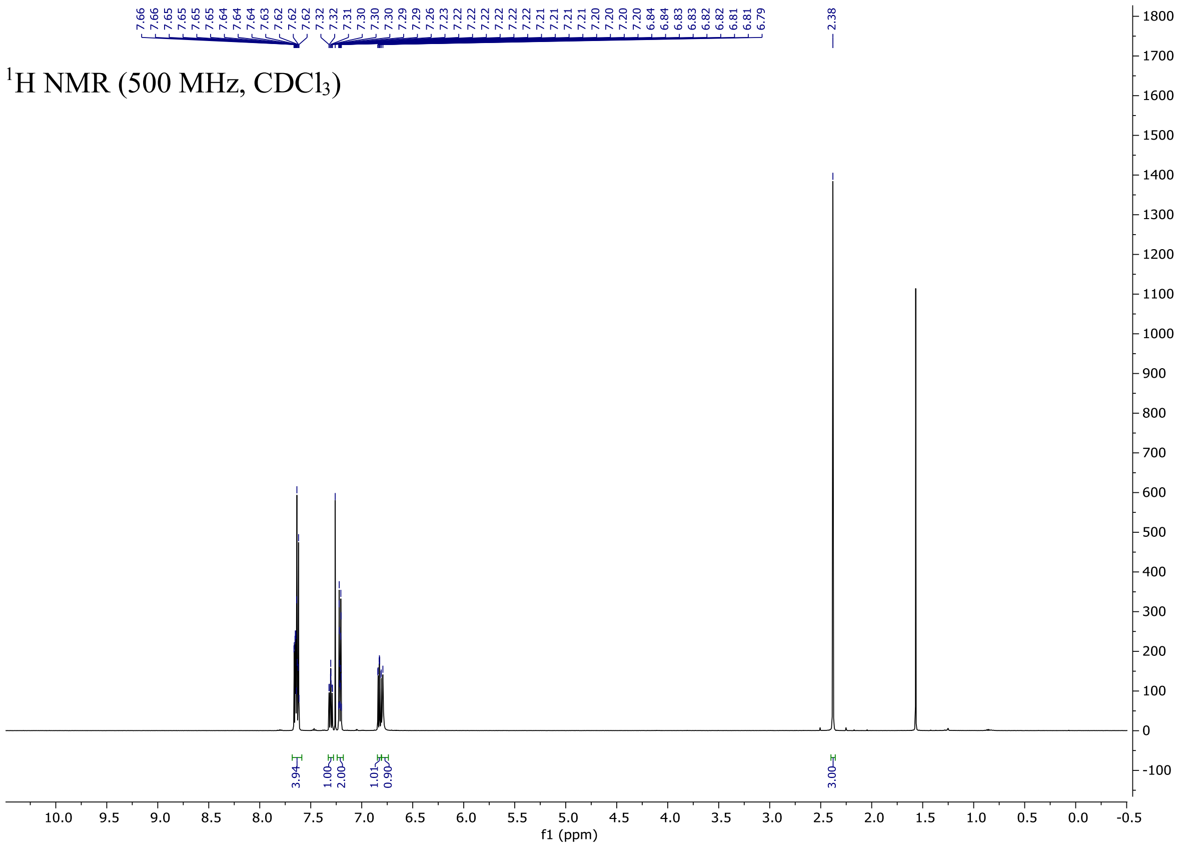

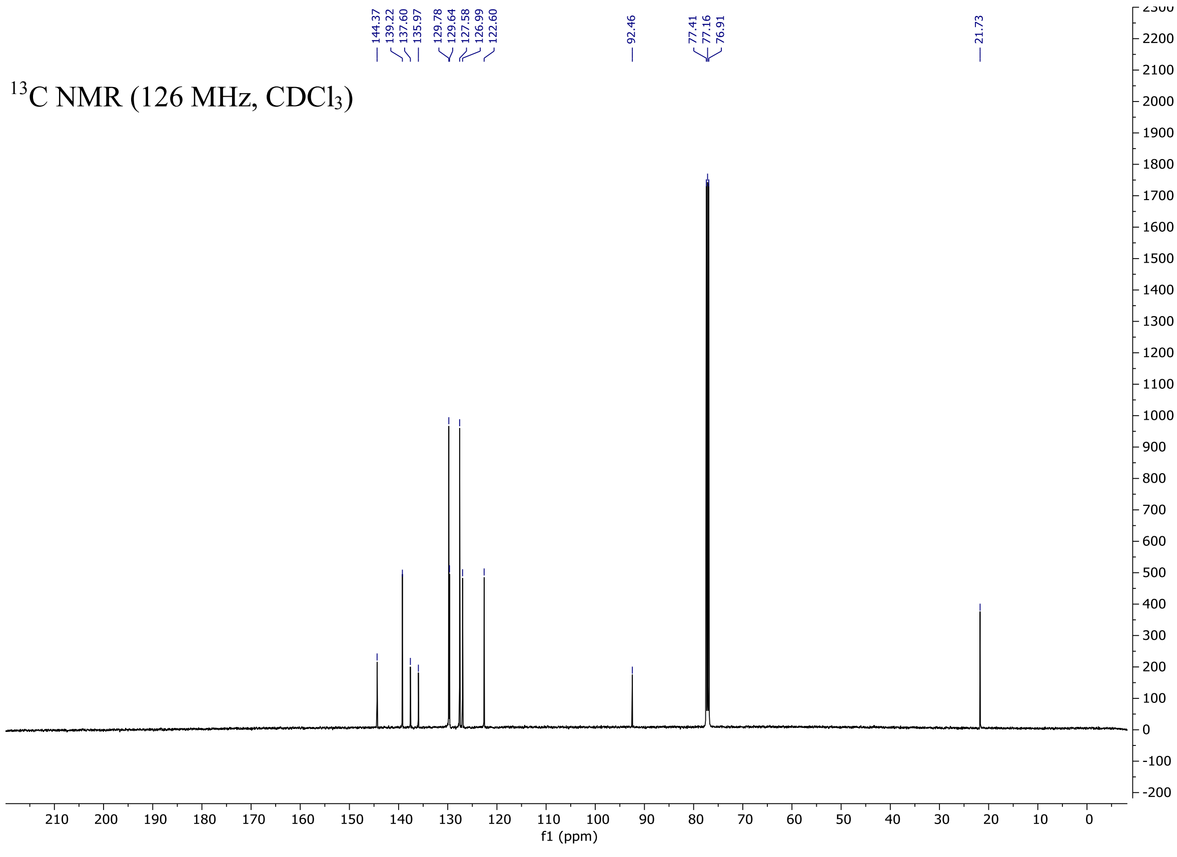

Compound S29:

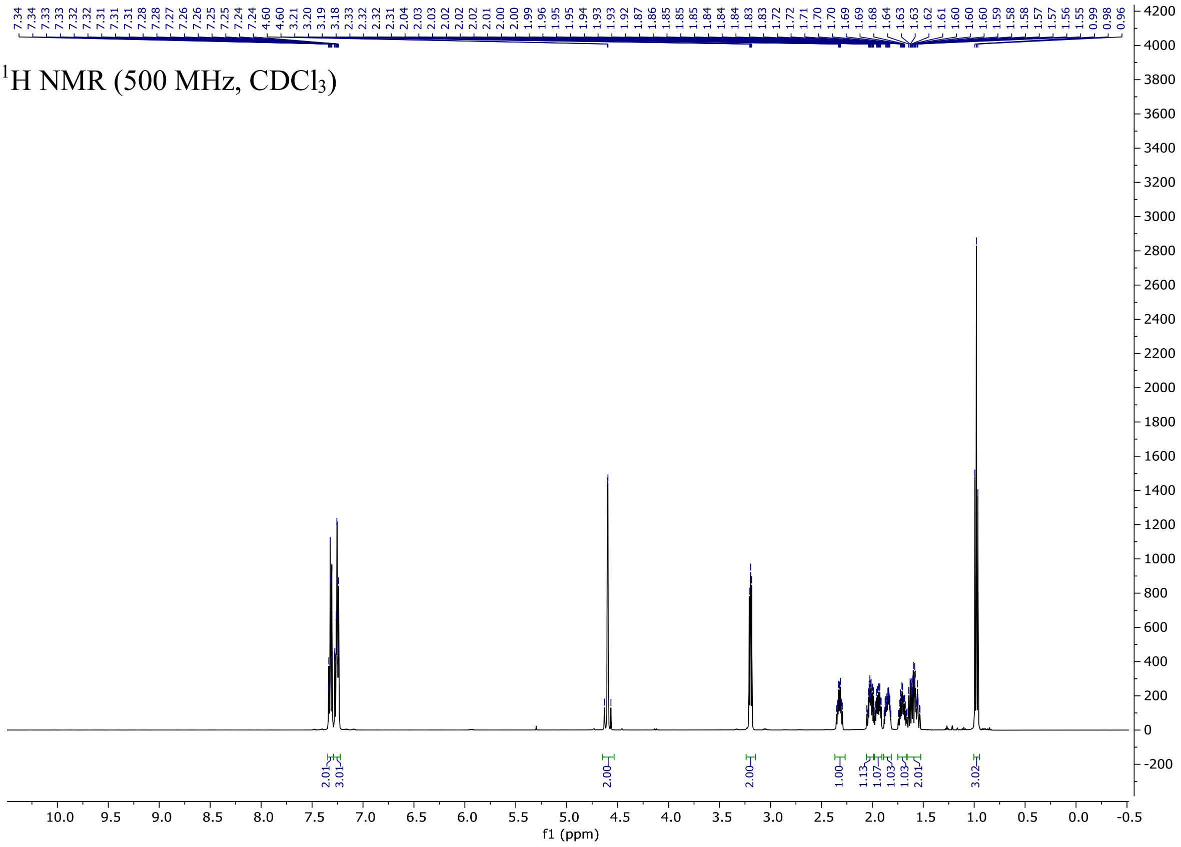

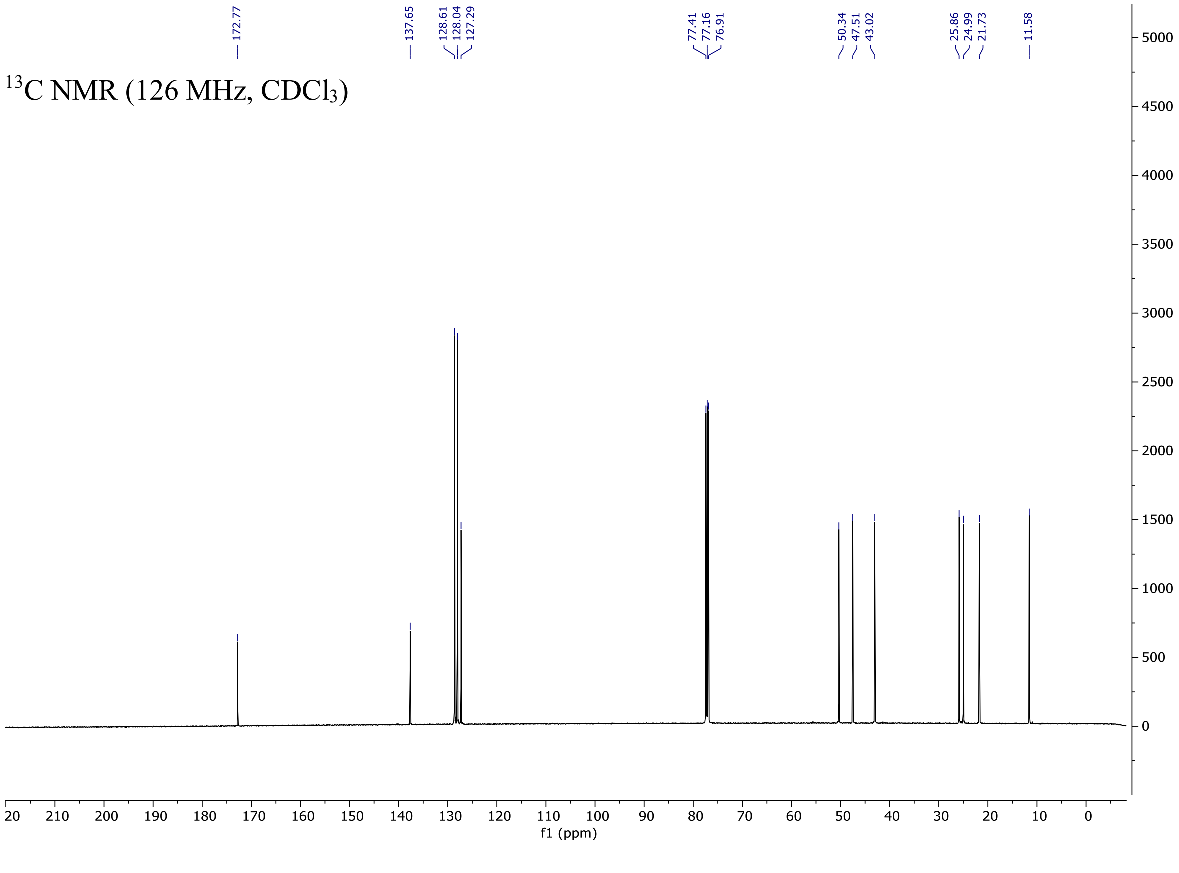

Compound S30:

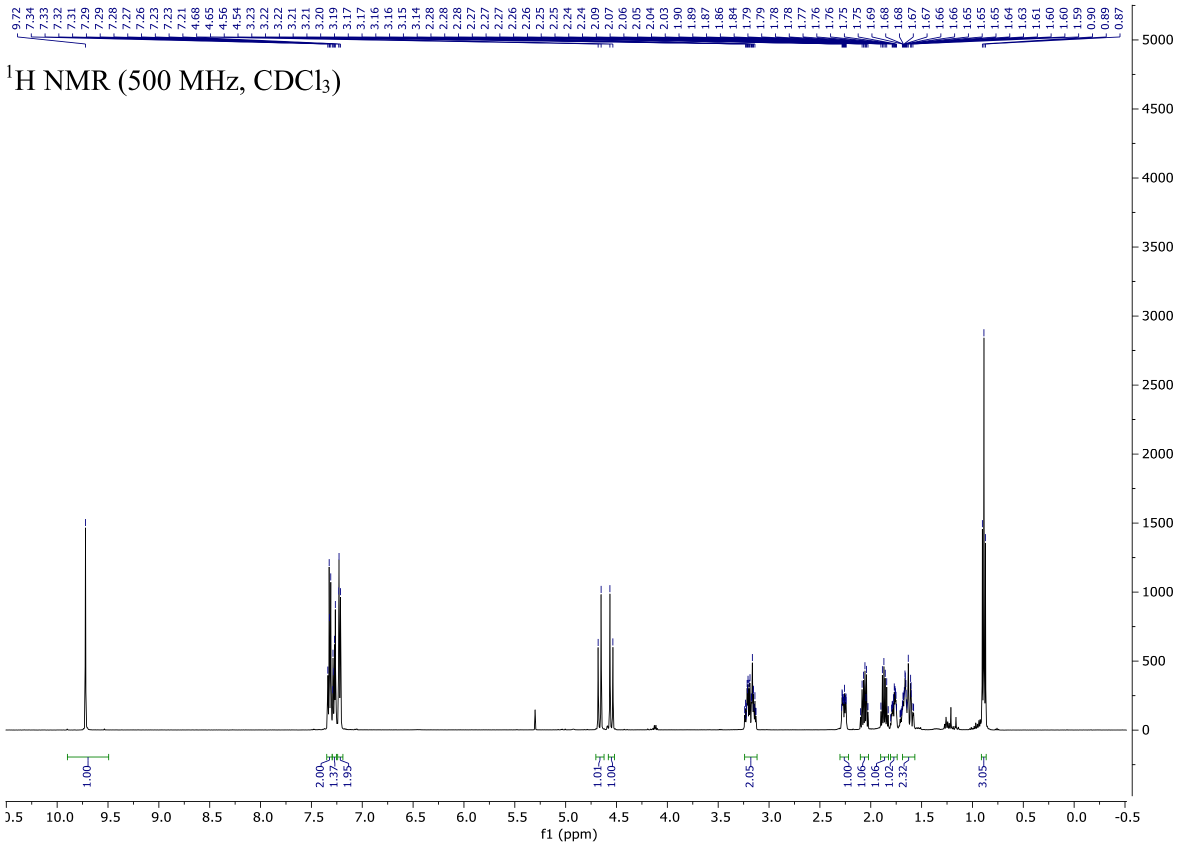

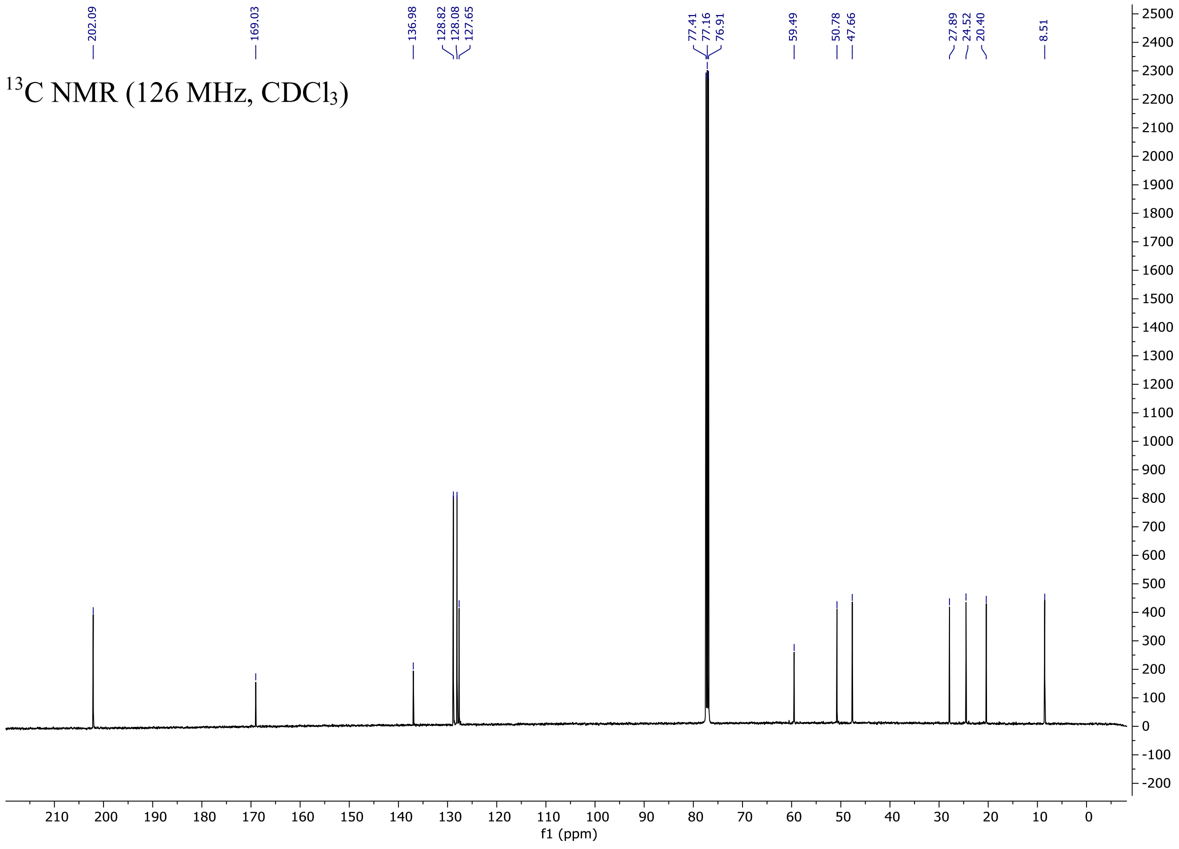

Compound S31:

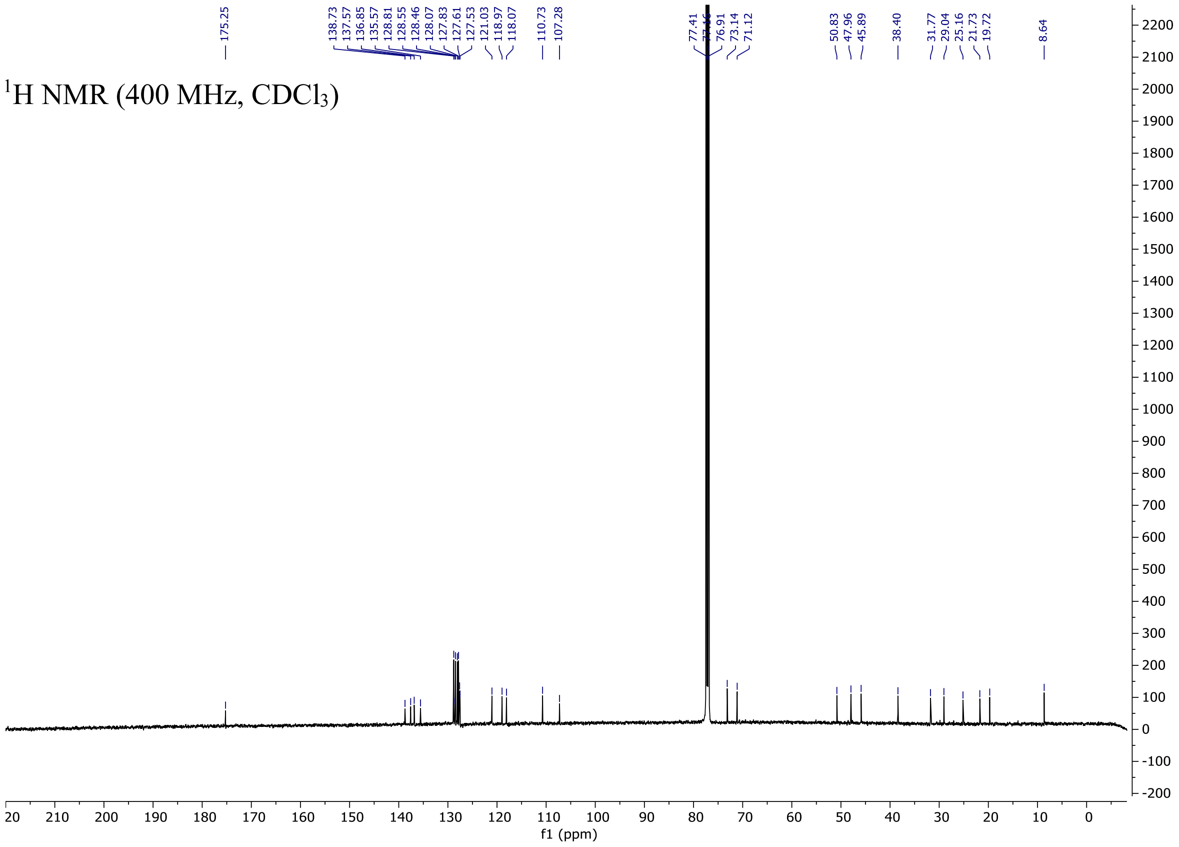

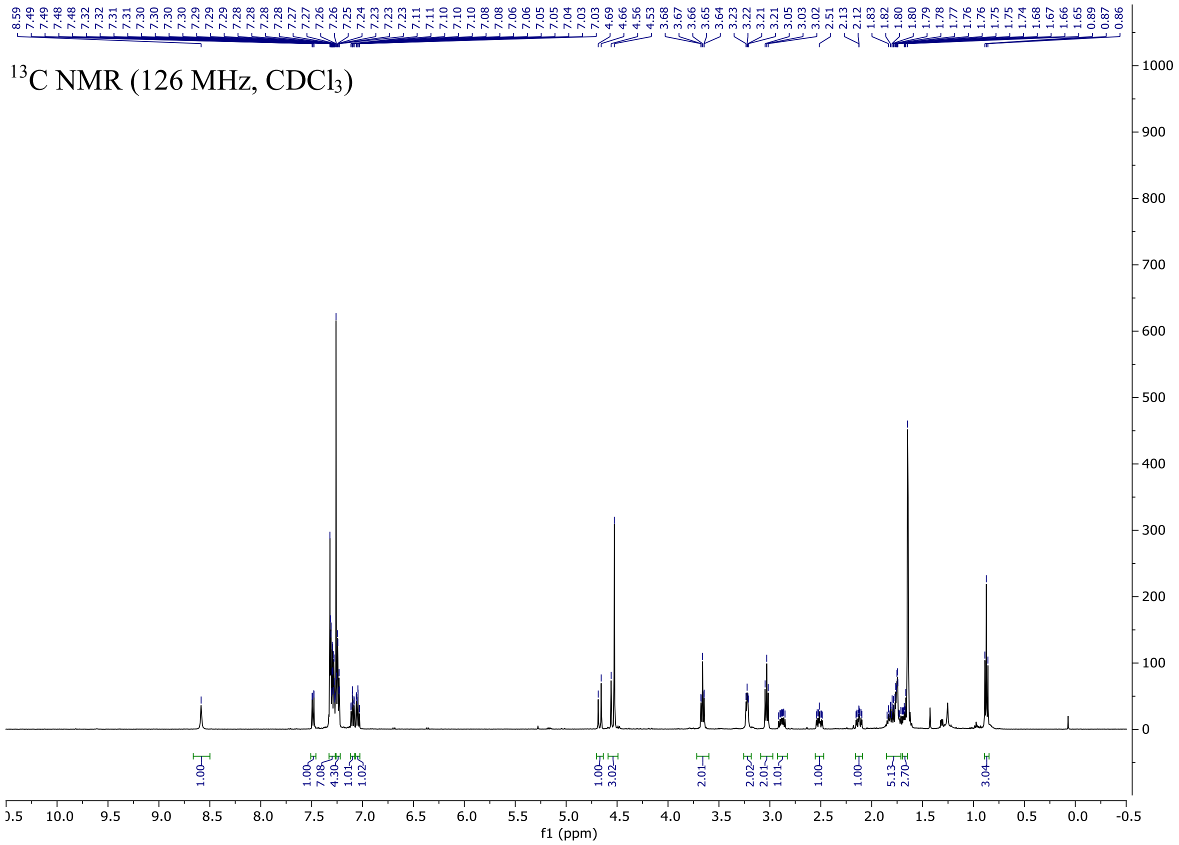

Compound S32:

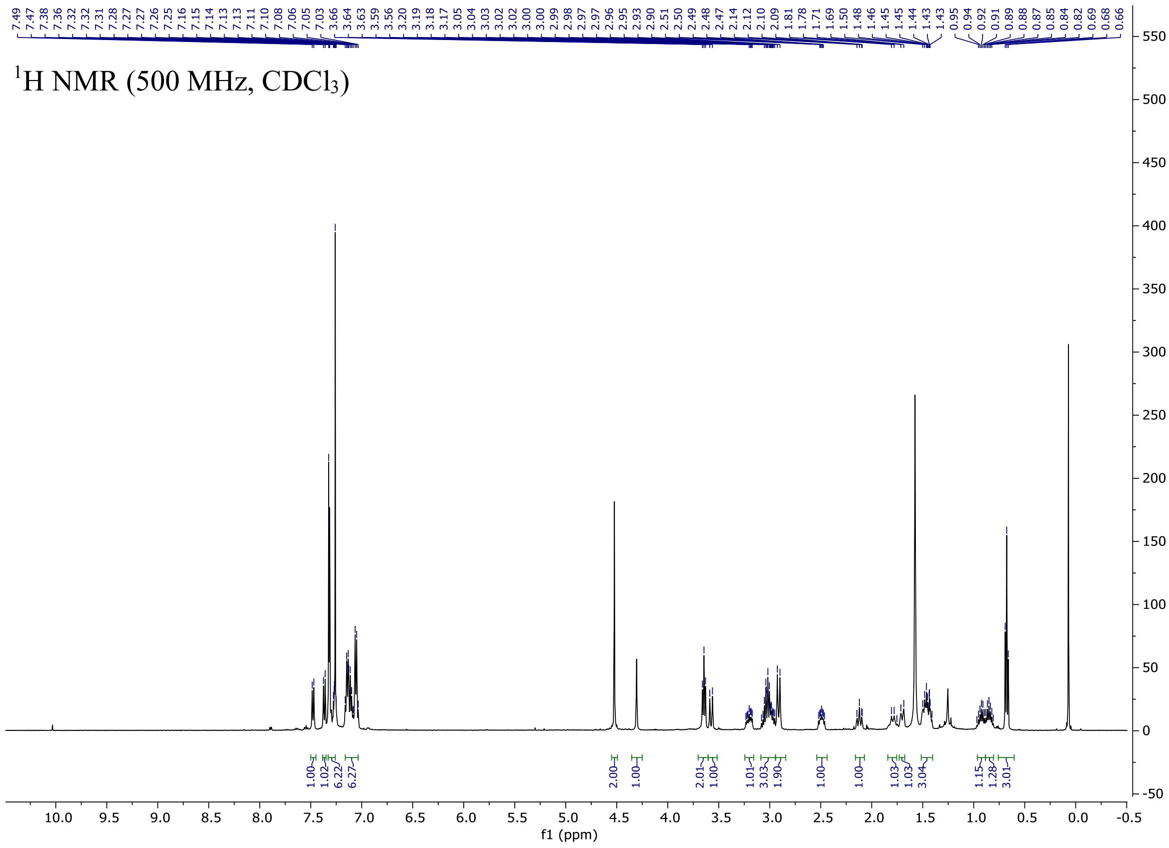

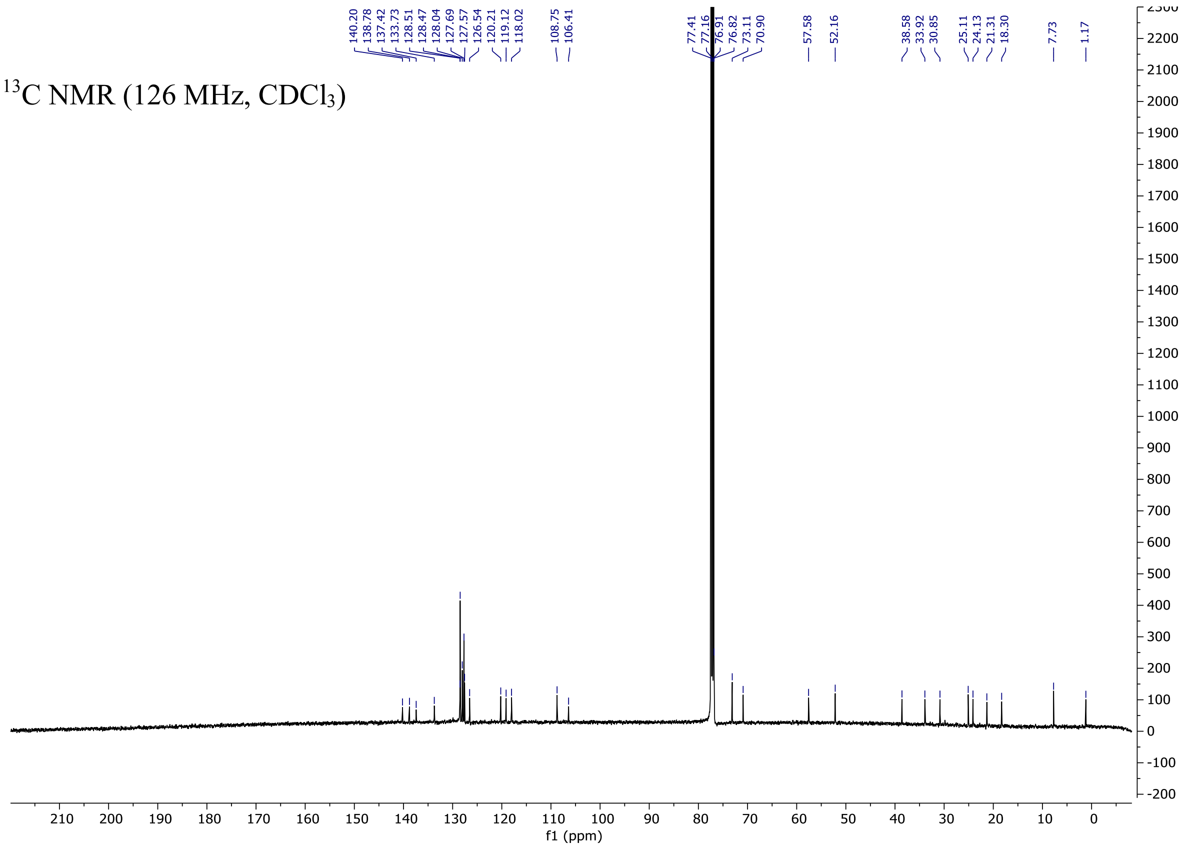

Compound 1b:

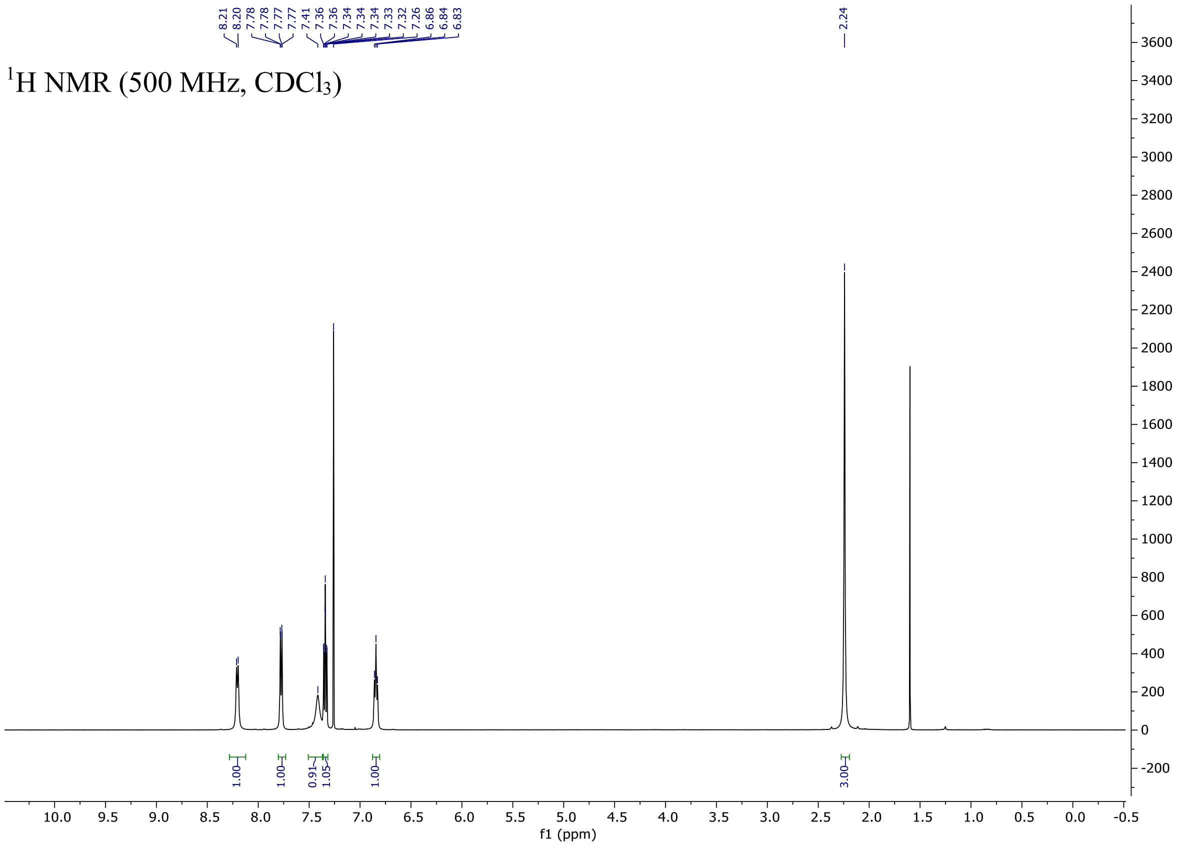
8.21
8.20
7.78
7.78
7.77
7.77
7.41
7.36
7.36
7.34
7.34
7.34
7.34
7.33
7.32
7.26
6.86
6.84
6.83
2.24
1H NMR (500 MHz, CDCl3)
1.00
1.00
0.91
1.05
1.00
3.00
f1 (ppm)

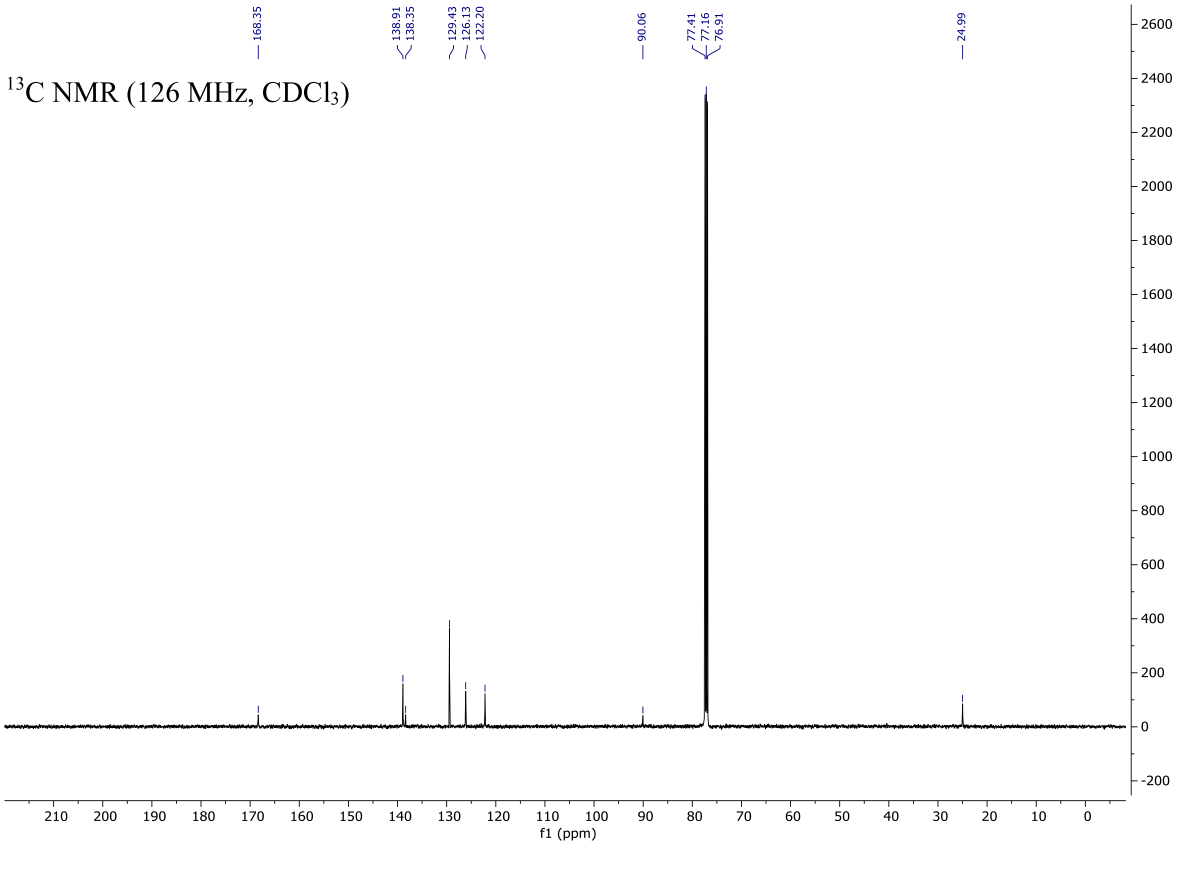
168.35
138.91
138.35
129.43
126.13
122.20
90.06
77.41
77.16
76.91
24.99
13C NMR (126 MHz, CDCl3)
f1 (ppm)

Compound 2b:

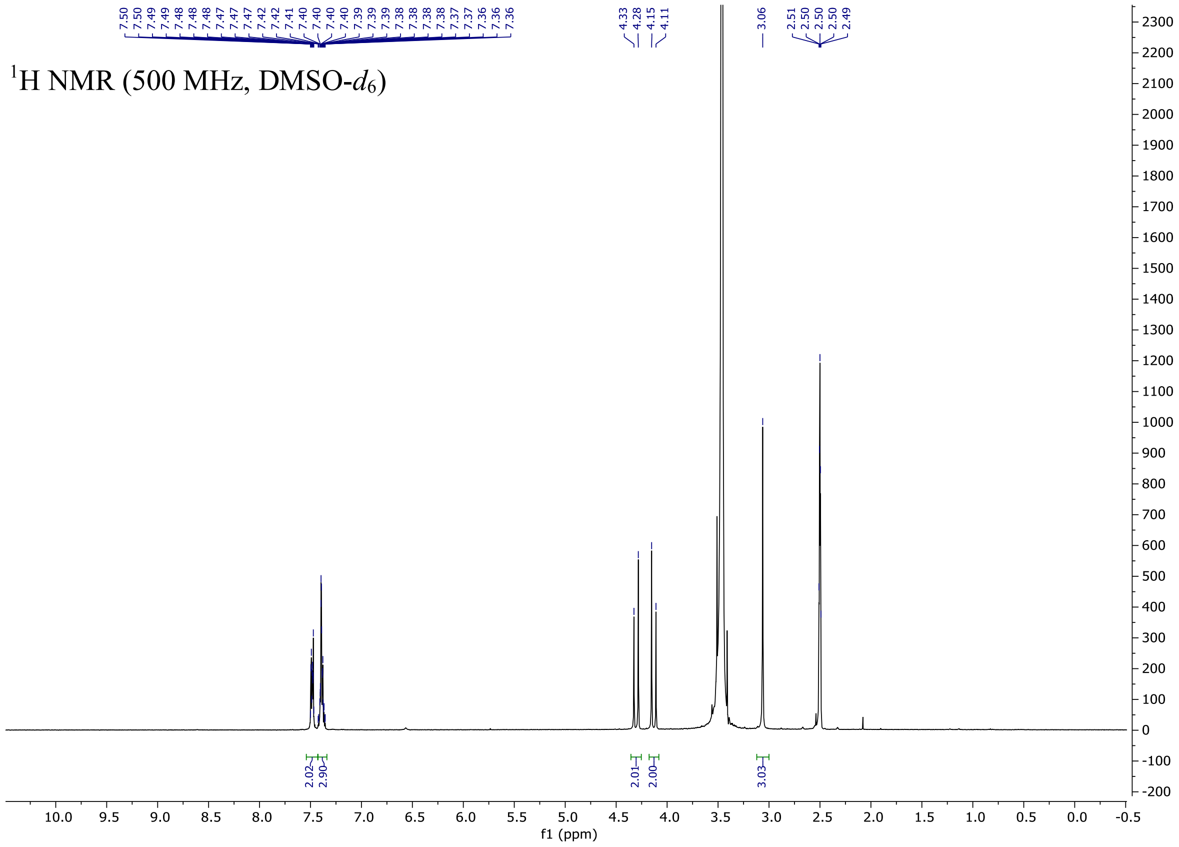

7.50
7.50
7.49
7.48
7.48
7.48
7.47
7.47
7.47
7.42
7.42
7.41
7.40
7.40
7.40
7.40
7.39
7.39
7.39
7.38
7.38
7.38
7.38
7.37
7.37
7.36
7.36
7.36
4.33
4.28
4.15
4.11
3.06
2.51
2.50
2.50
2.50
2.49
^{1}H NMR (500 MHz, DMSO-d_6)
2.02
2.90
2.01
2.00
3.03
f1 (ppm)

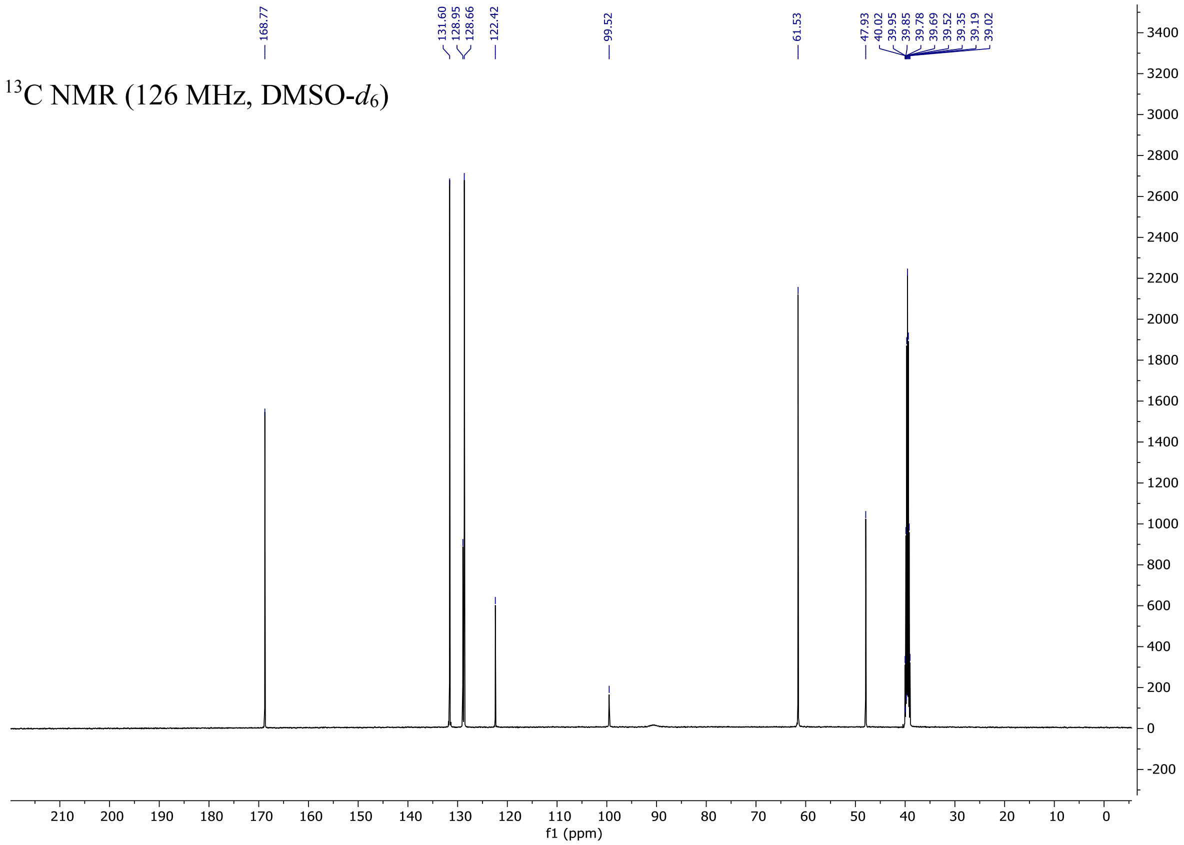

168.77
131.60
128.95
128.66
122.42
99.52
61.53
47.93
40.02
39.95
39.85
39.78
39.69
39.52
39.35
39.19
39.02
^{13}C NMR (126 MHz, DMSO-d_6)
f1 (ppm)

Compound 3:

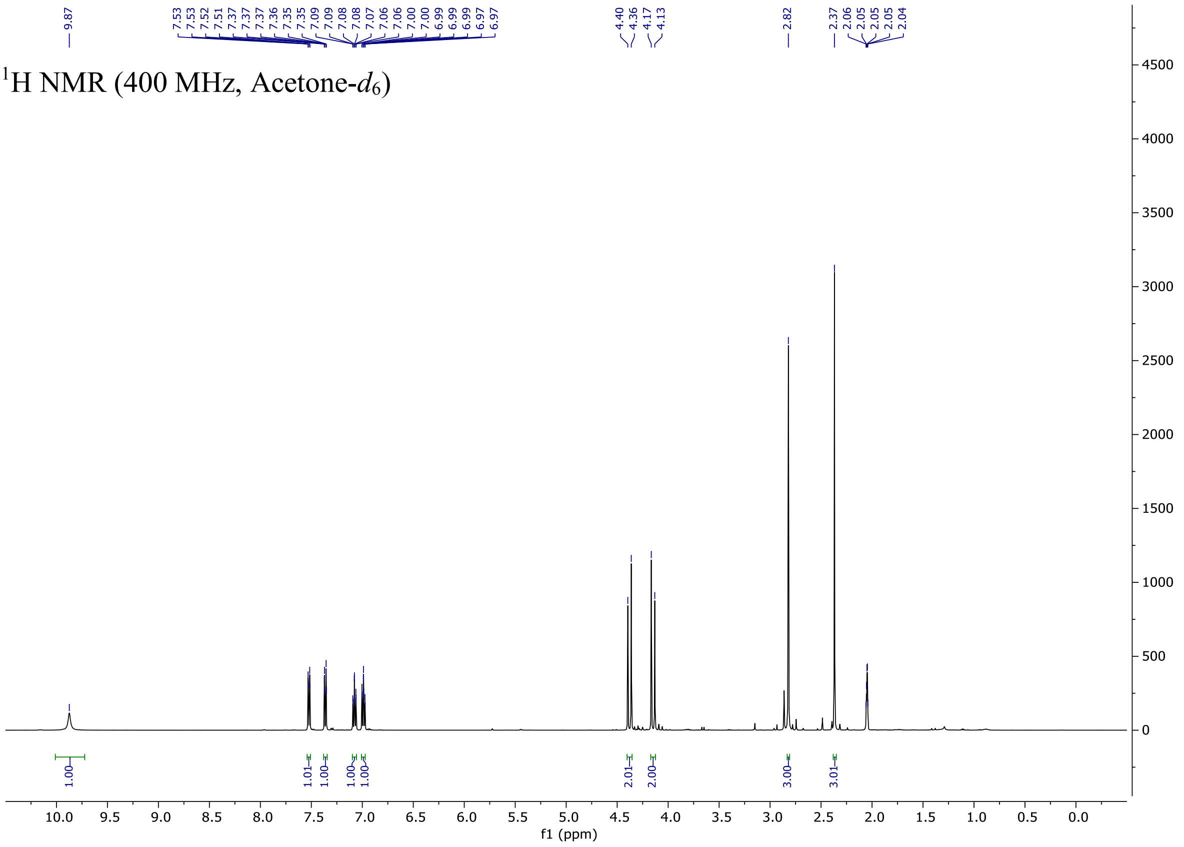

^{1}H NMR (400 MHz, Acetone-d_6)
9.87
7.53 7.53 7.52 7.51 7.37 7.37 7.36 7.35 7.35 7.09 7.09 7.08 7.07 7.06 7.06 7.00 7.00 6.99 6.99 6.99 6.97 6.97
4.40 4.36 4.17 4.13
2.82
2.37 2.06 2.05 2.05 2.05 2.04
1.00 1.01 1.00 1.00 1.00 2.01 2.00 3.00 3.01
f1 (ppm)

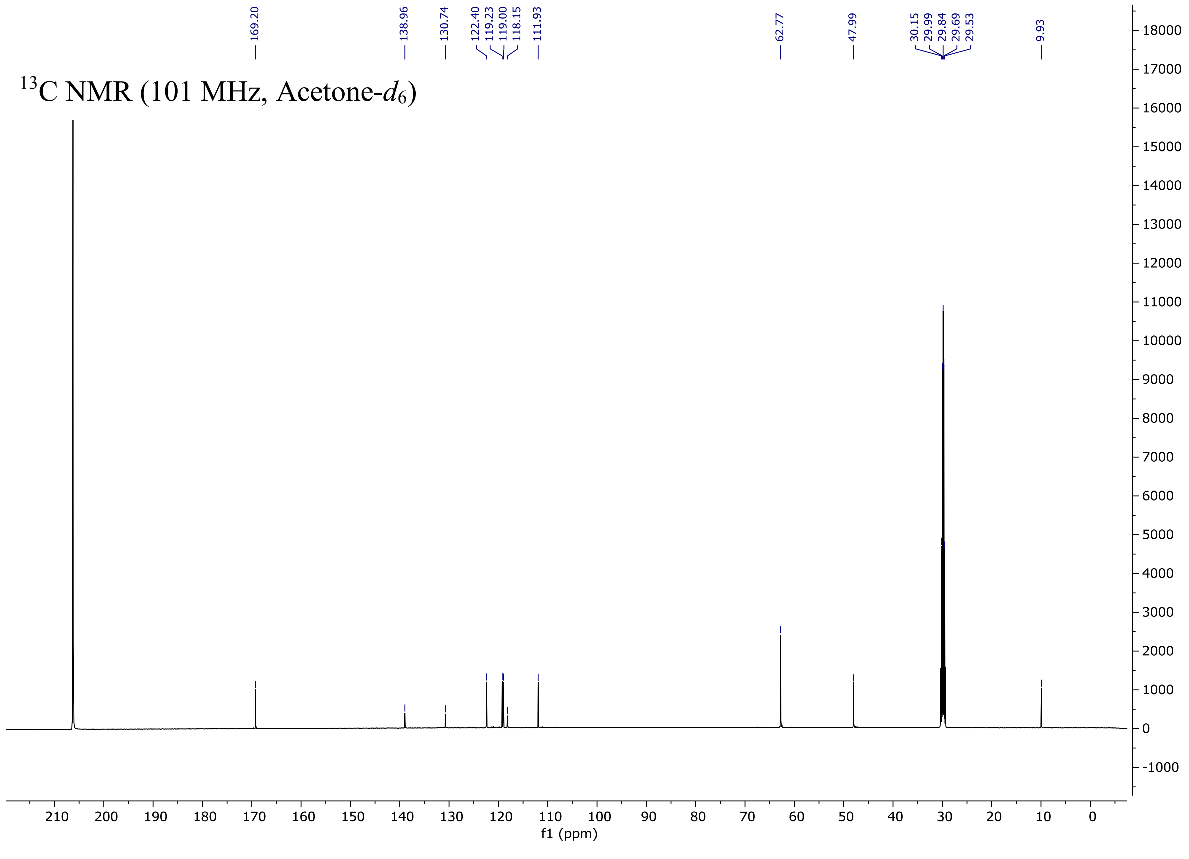

^{13}C NMR (101 MHz, Acetone-d_6)
169.20
138.96
130.74
122.40 119.23 119.00 118.15
111.93
62.77
47.99
30.15 29.99 29.84 29.69 29.53
9.93
f1 (ppm)

Compound 4:

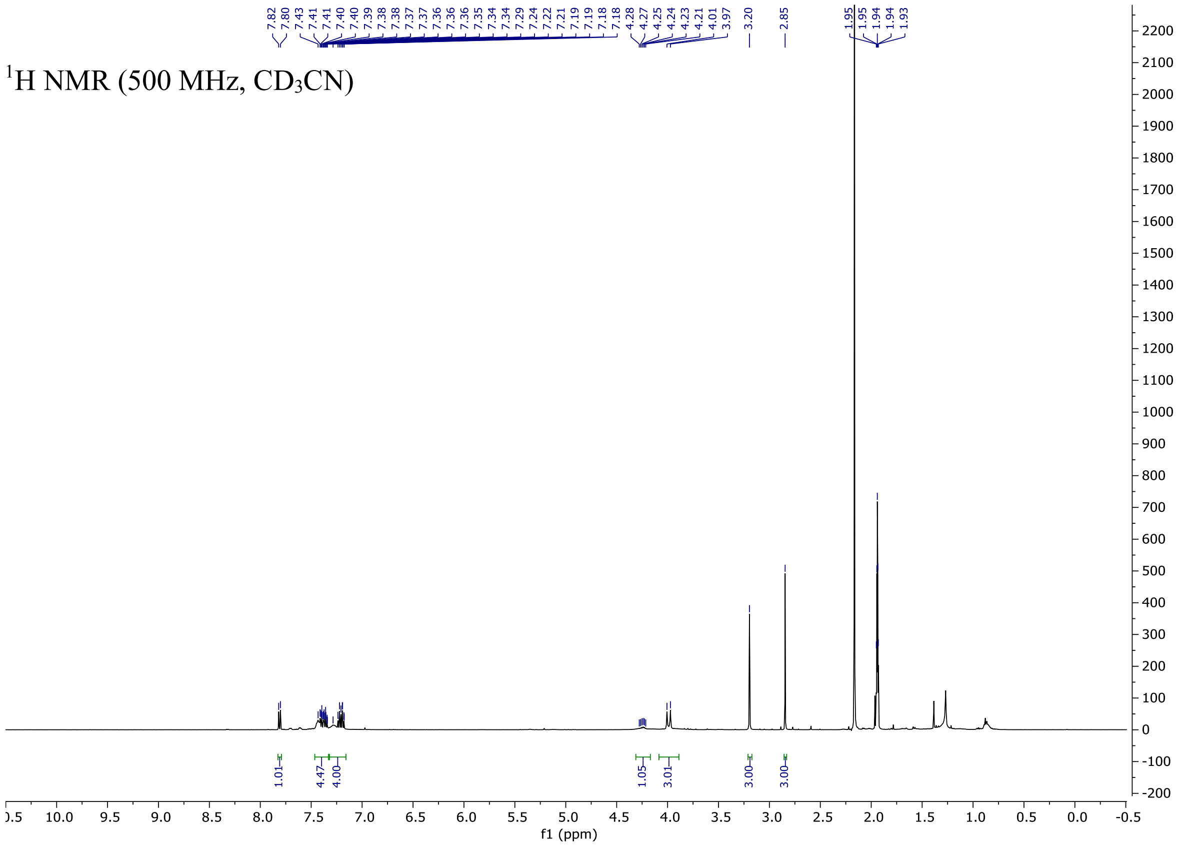

^{1}H NMR (500 MHz, CD$_3$CN)
7.82
7.80
7.43
7.41
7.41
7.40
7.39
7.38
7.38
7.37
7.37
7.36
7.36
7.36
7.35
7.34
7.34
7.29
7.24
7.22
7.21
7.19
7.19
7.18
4.28
4.27
4.25
4.24
4.23
4.21
4.01
3.97
3.20
2.85
1.95
1.95
1.94
1.94
1.93
1.01
4.47
4.00
1.05
3.01
3.00
3.00
f1 (ppm)

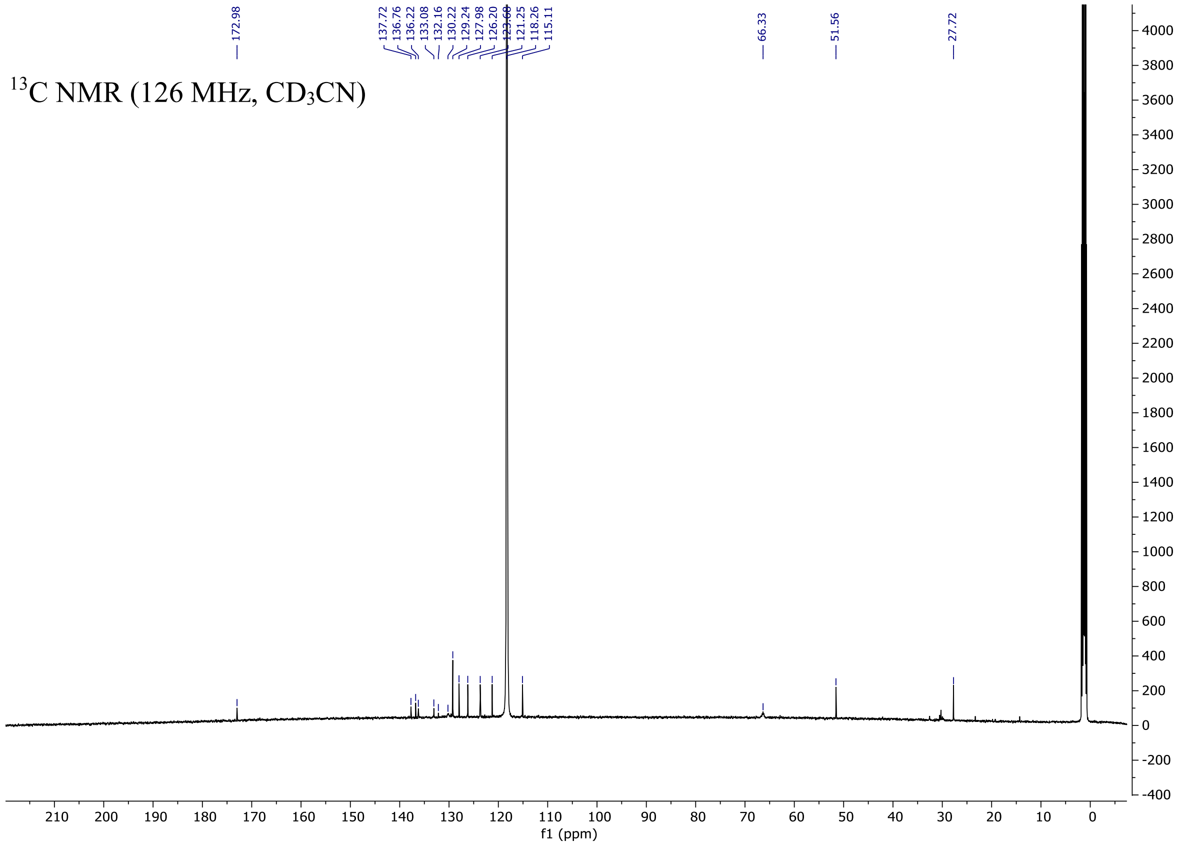

^{13}C NMR (126 MHz, CD$_3$CN)
172.98
137.72
136.76
136.22
133.08
132.16
130.22
129.24
127.98
126.20
123.69
121.25
118.26
115.11
66.33
51.56
27.72
f1 (ppm)

Compound 5:

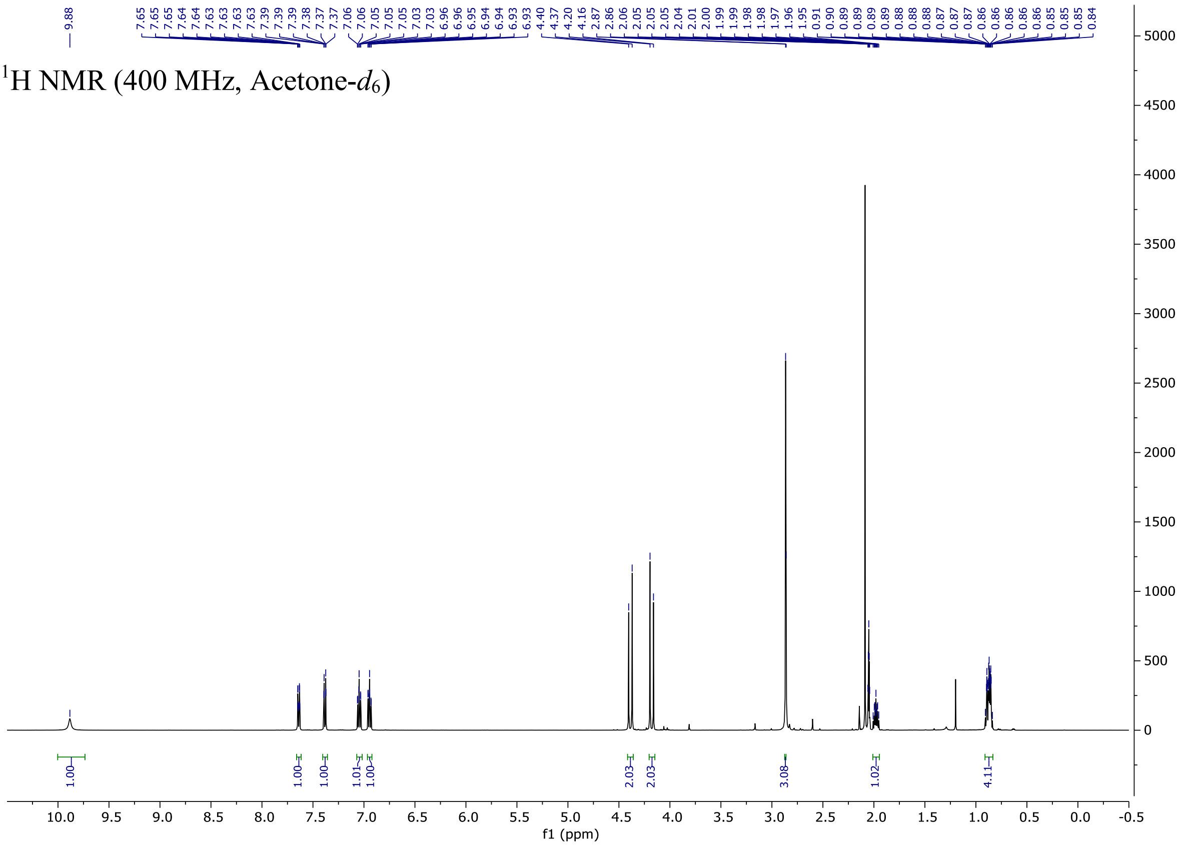

^{1}H NMR (400 MHz, Acetone-d_6)
f1 (ppm)

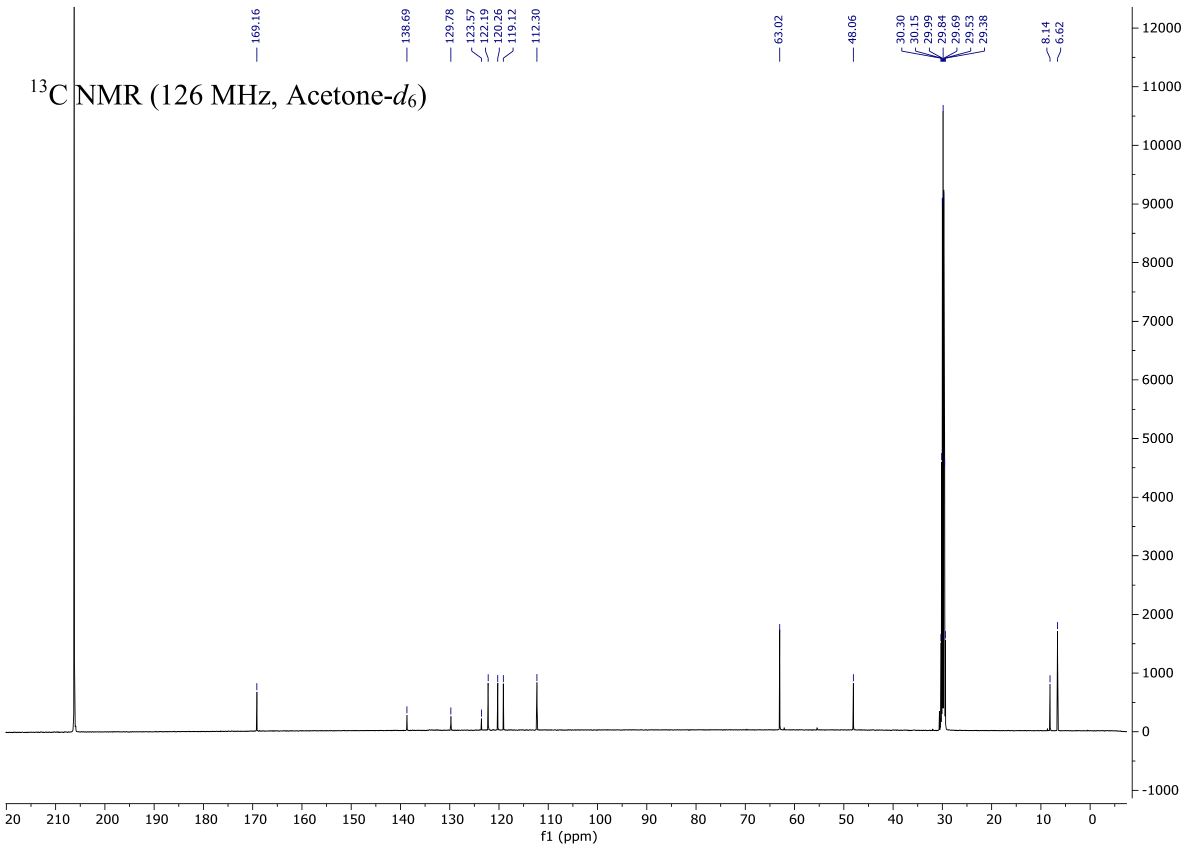

^{13}C NMR (126 MHz, Acetone-d_6)
169.16
138.69
129.78
123.57
122.19
120.26
119.12
112.30
63.02
48.06
30.30
30.15
29.99
29.84
29.69
29.53
29.38
8.14
6.62
f1 (ppm)

Compound 6:

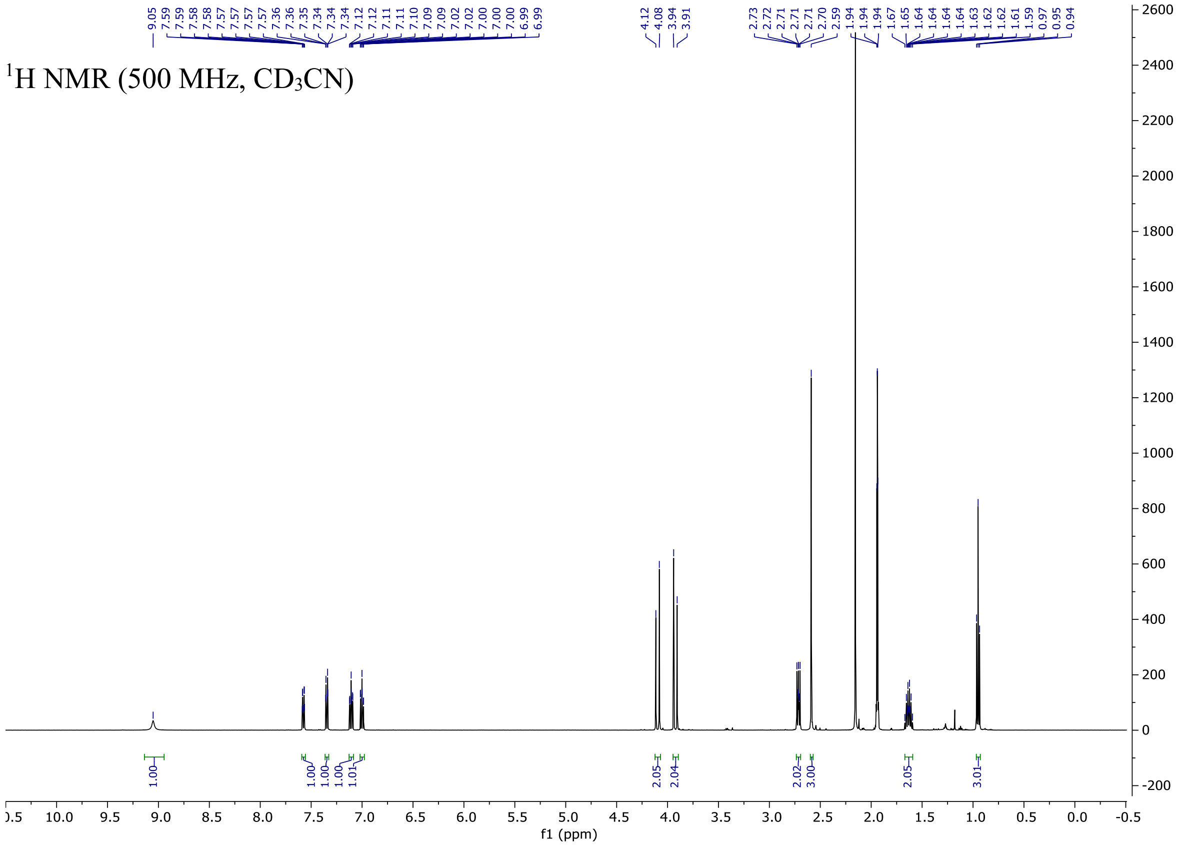

^{1}H NMR (500 MHz, CD3CN)
f1 (ppm)

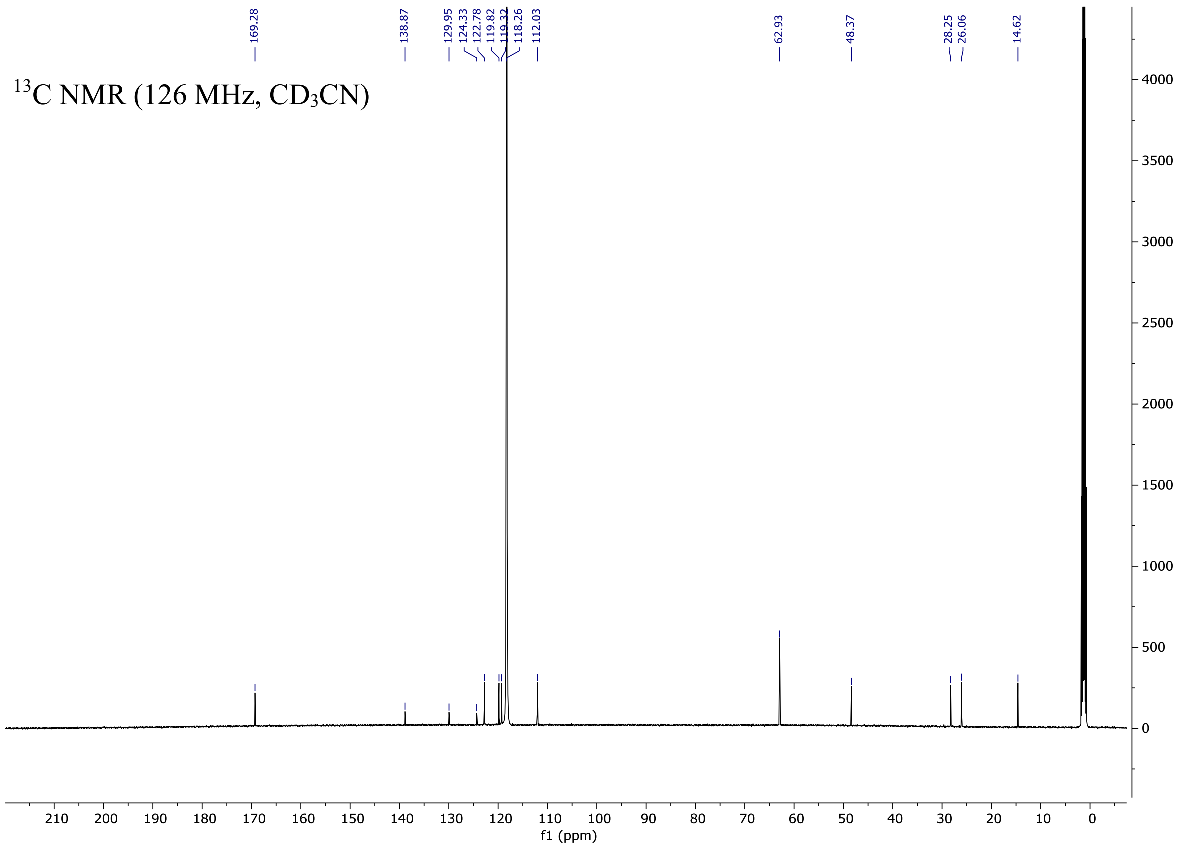

^{13}C NMR (126 MHz, CD3CN)
169.28
138.87
129.95
124.33
122.78
119.82
119.32
118.26
112.03
62.93
48.37
28.25
26.06
14.62
f1 (ppm)

Compound 7:

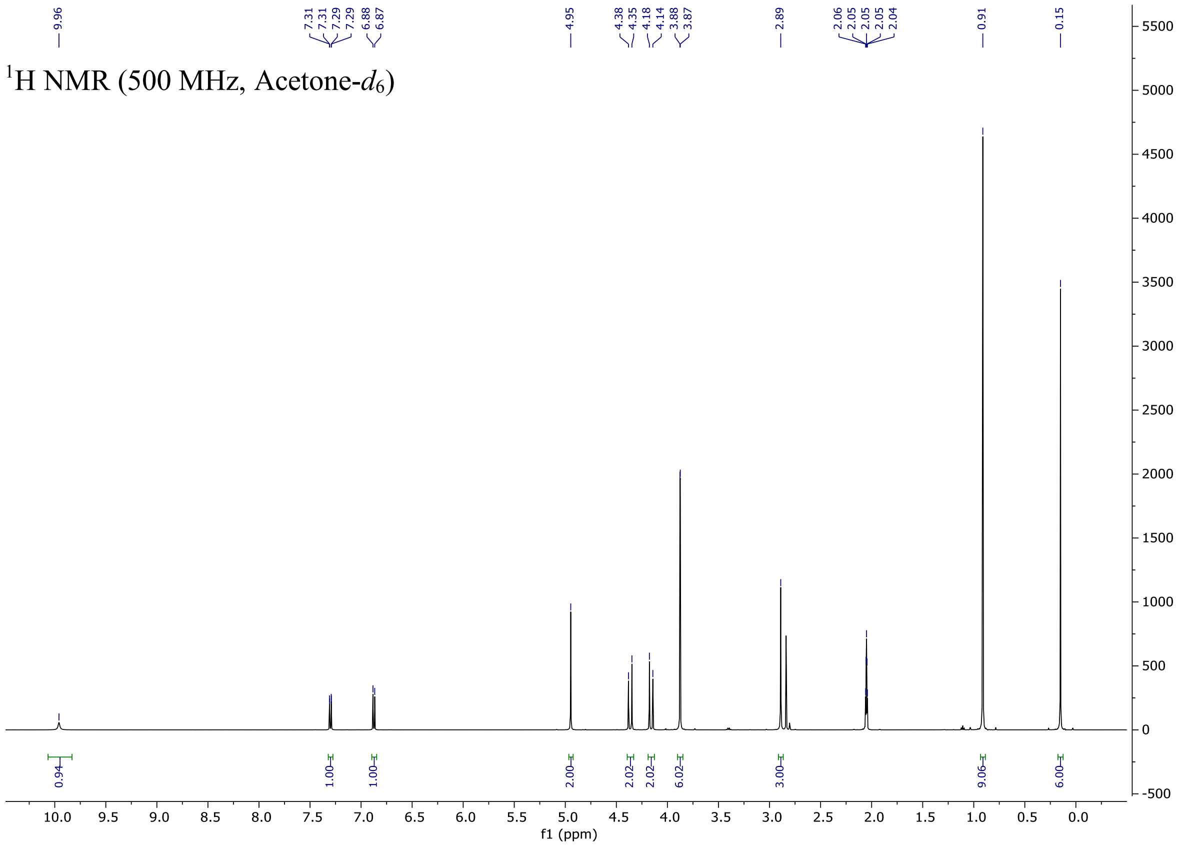

¹H NMR (500 MHz, Acetone-d6)
9.96
7.31
7.31
7.29
7.29
6.88
6.87
4.95
4.38
4.35
4.18
4.14
3.88
3.87
2.89
2.06
2.05
2.05
2.05
2.04
0.91
0.15
0.94
1.00
1.00
2.00
2.02
2.02
6.02
3.00
9.06
6.00
f1 (ppm)

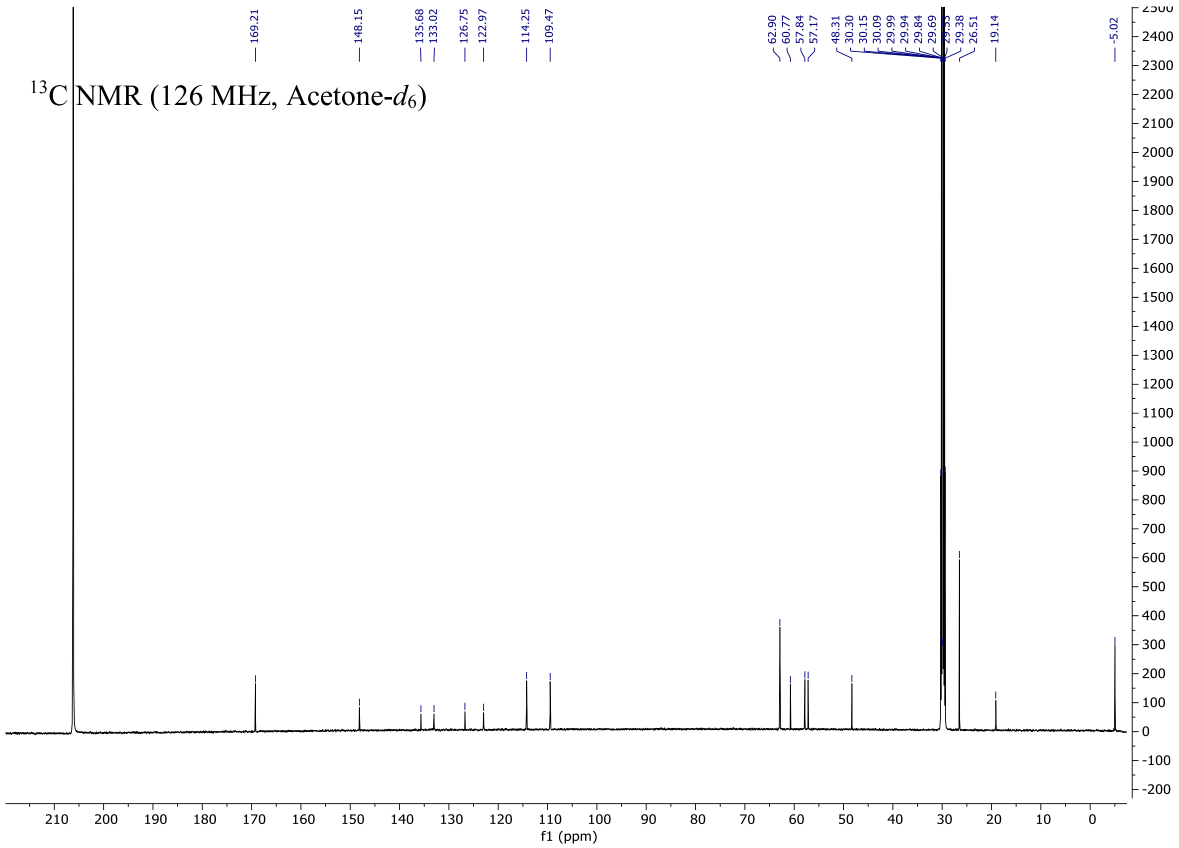

¹³C NMR (126 MHz, Acetone-d6)
169.21
148.15
135.68
133.02
126.75
122.97
114.25
109.47
62.90
60.77
57.84
57.17
48.31
30.30
30.15
30.09
29.99
29.94
29.84
29.69
29.53
29.38
26.51
19.14
-5.02
f1 (ppm)

Compound 8:

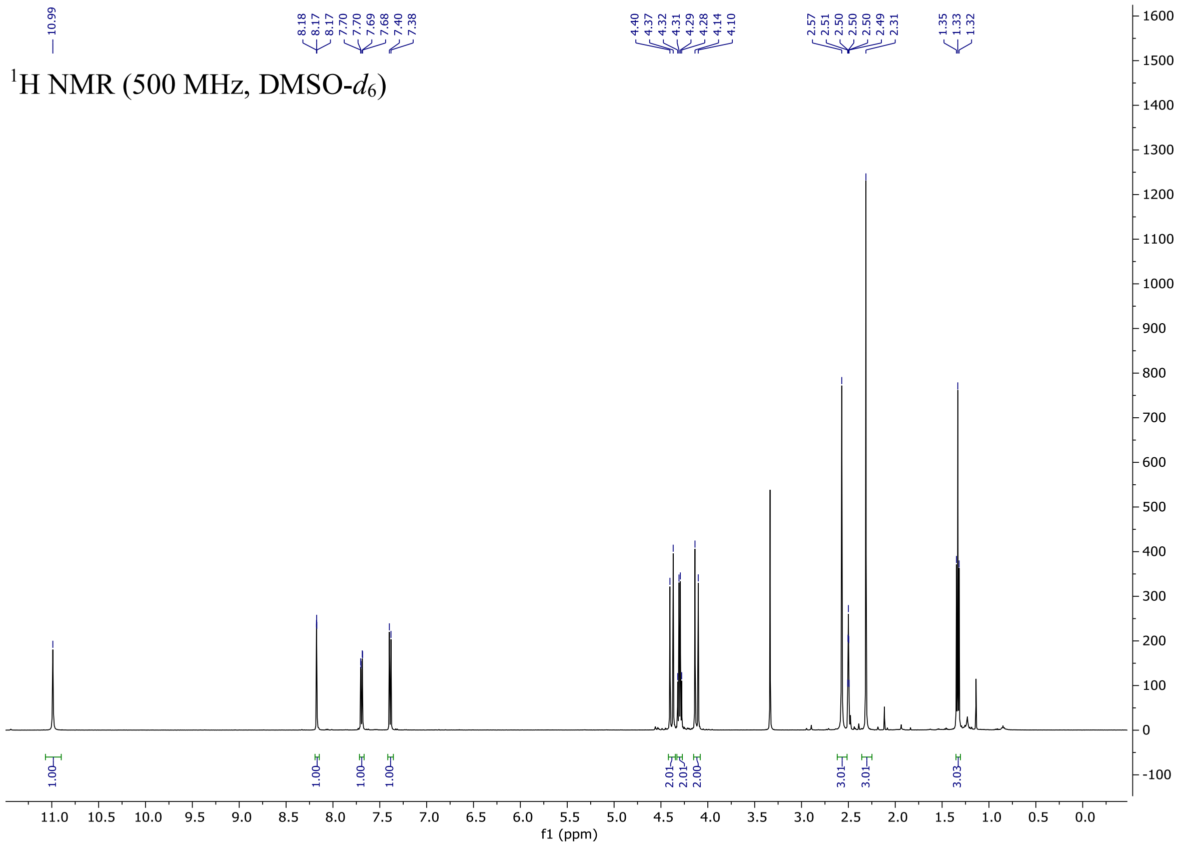

^{1}H NMR (500 MHz, DMSO-d_6)
10.99
8.18
8.17
8.17
7.70
7.70
7.69
7.68
7.40
7.38
4.40
4.37
4.32
4.31
4.29
4.28
4.14
4.10
2.57
2.51
2.50
2.50
2.50
2.49
2.31
1.35
1.33
1.32
1.00
1.00
1.00
1.00
2.01
2.01
2.00
3.01
3.01
3.03
f1 (ppm)

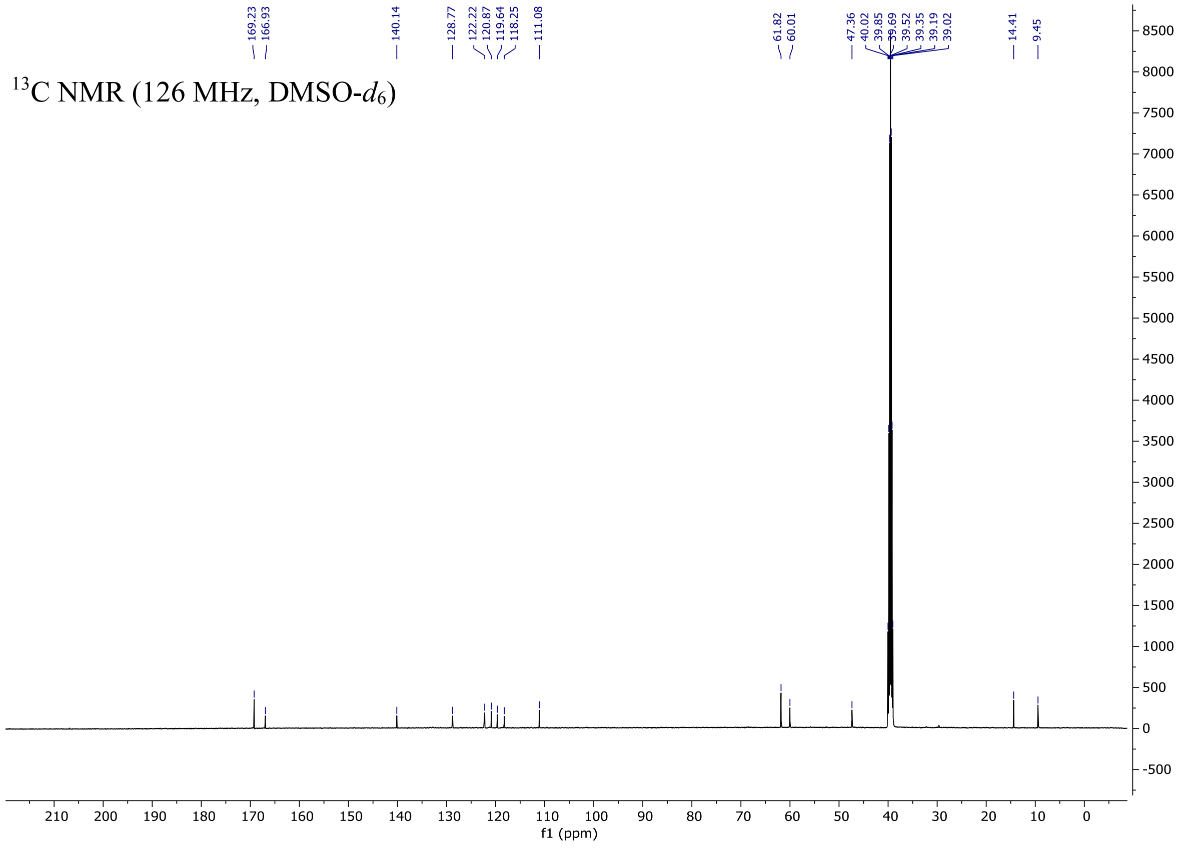

^{13}C NMR (126 MHz, DMSO-d_6)
169.23
166.93
140.14
128.77
122.22
120.87
119.64
118.25
111.08
61.82
60.01
47.36
40.02
39.85
39.69
39.52
39.35
39.19
39.02
14.41
9.45
f1 (ppm)

Compound 9:

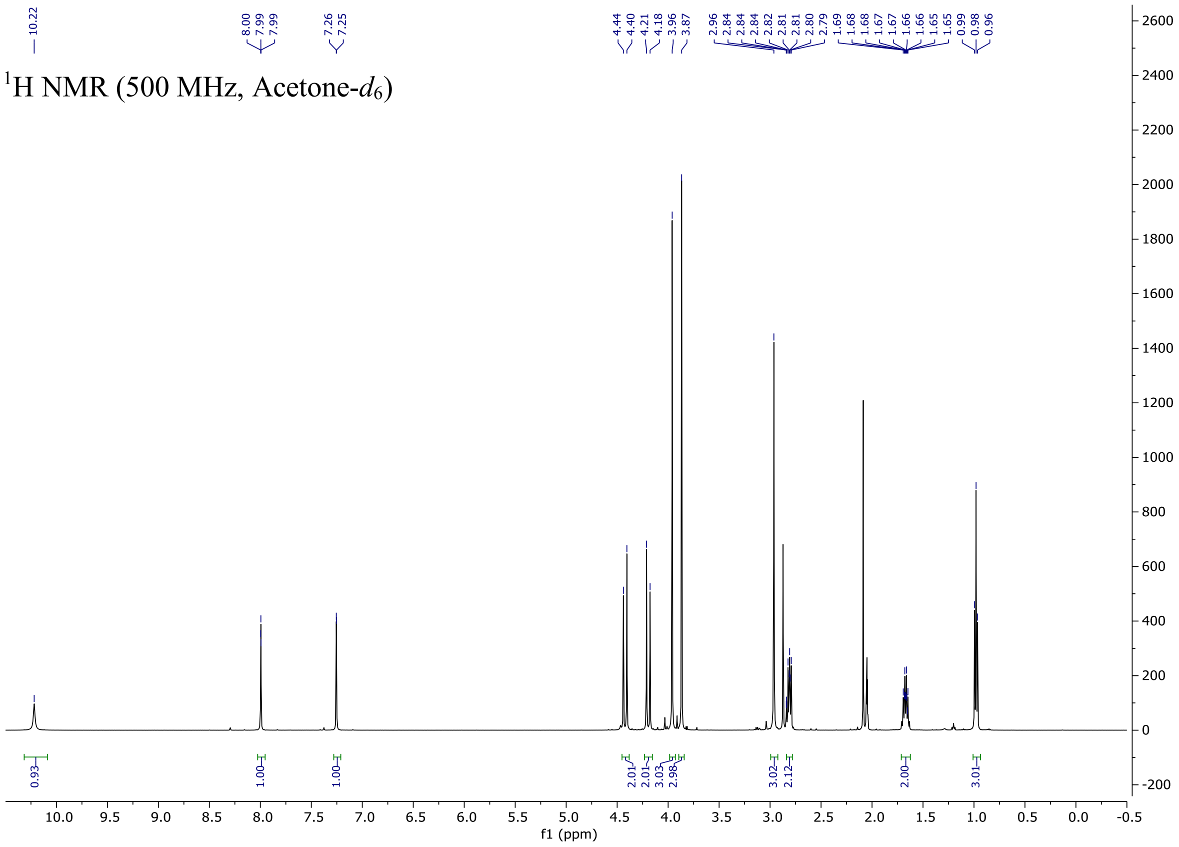

10.22
8.00
7.99
7.99
7.26
7.25
4.44
4.40
4.21
4.18
3.96
3.87
2.96
2.84
2.84
2.84
2.82
2.81
2.81
2.80
2.79
1.69
1.68
1.68
1.67
1.67
1.66
1.66
1.65
1.65
0.99
0.98
0.96
^{1}H NMR (500 MHz, Acetone-d_6)
0.93
1.00
1.00
2.01
2.01
3.03
2.98
3.02
2.12
2.00
3.01
f1 (ppm)

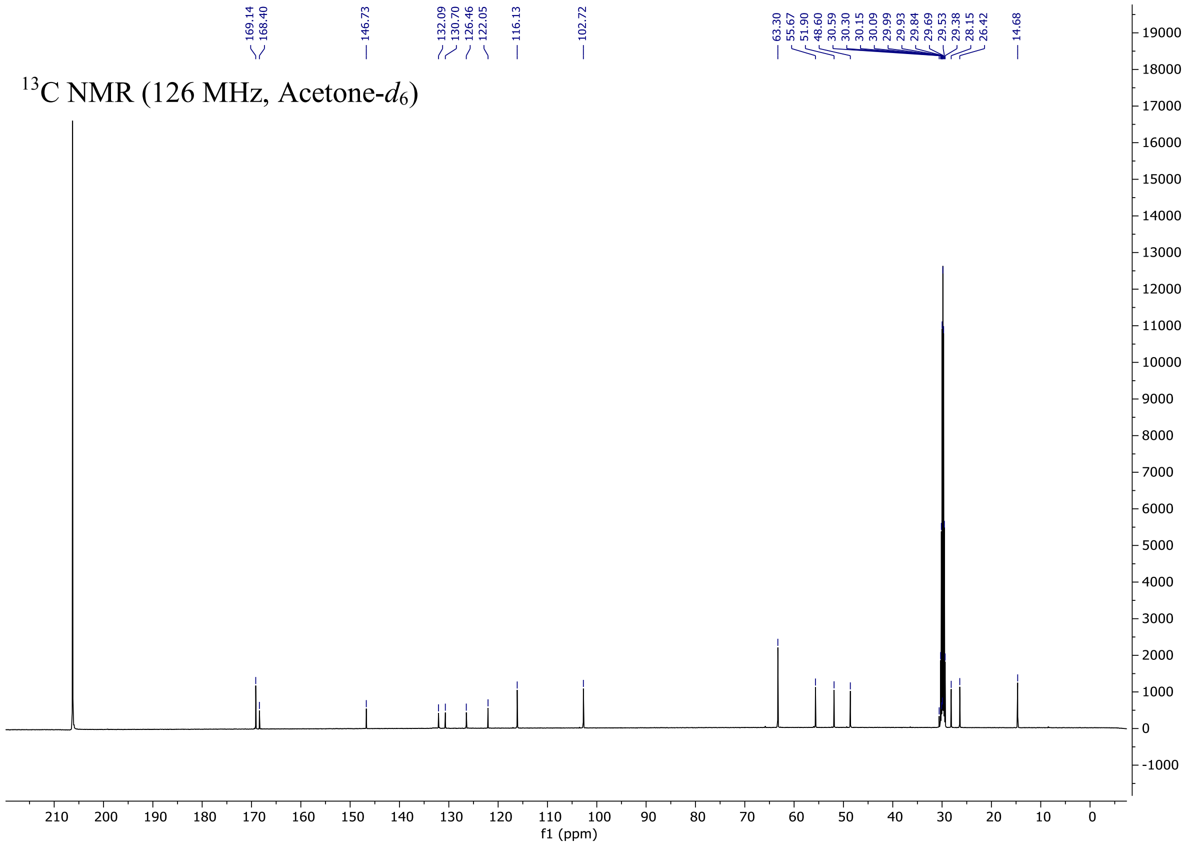

169.14
168.40
146.73
132.09
130.70
126.46
122.05
116.13
102.72
63.30
55.67
51.90
48.60
30.59
30.30
30.15
30.09
29.99
29.93
29.84
29.69
29.53
29.38
28.15
26.42
14.68
^{13}C NMR (126 MHz, Acetone-d_6)
f1 (ppm)

Compound 10:

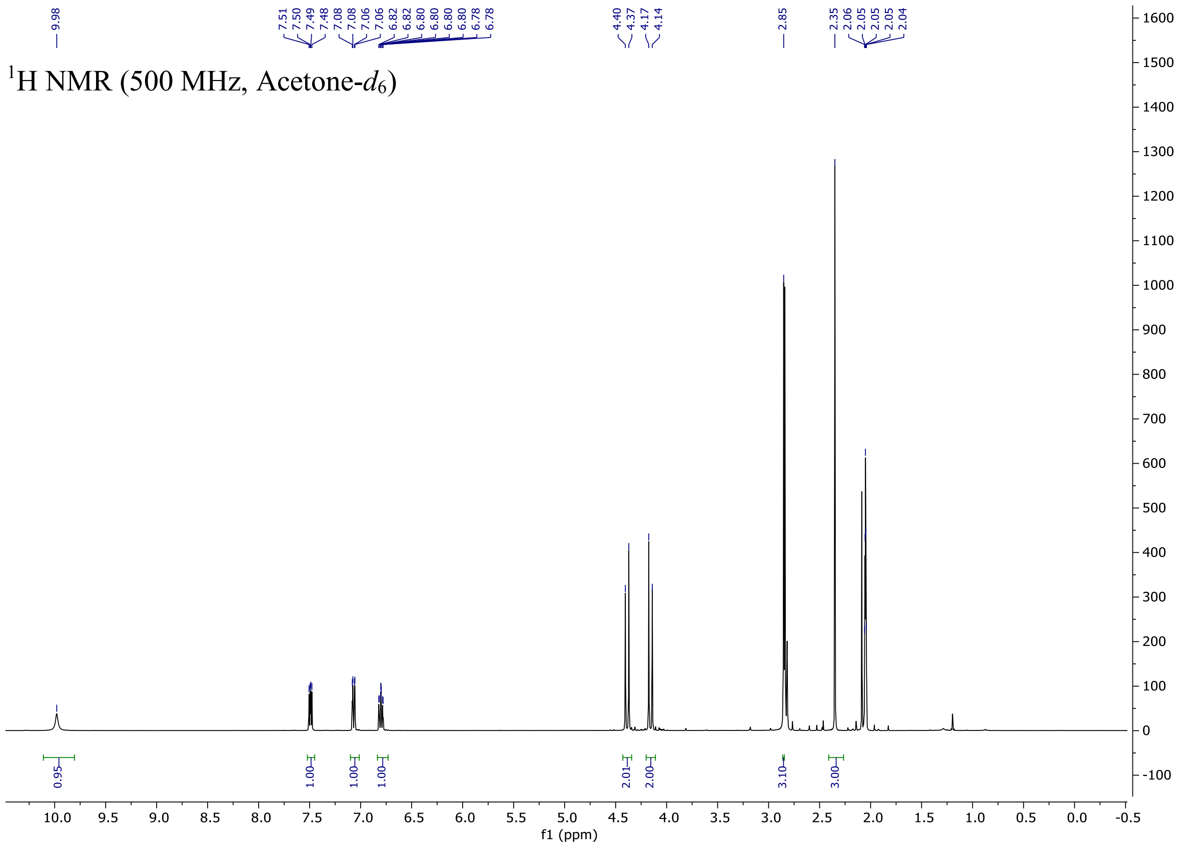

^{1}H NMR (500 MHz, Acetone-d_6)
9.98
7.51 7.50 7.49 7.48 7.08 7.08 7.06 7.06 6.82 6.82 6.80 6.80 6.80 6.80 6.78 6.78
4.40 4.37 4.17 4.14
2.85
2.35 2.06 2.05 2.05 2.05 2.04
0.95 1.00 1.00 1.00 2.01 2.00 3.10 3.00
f1 (ppm)

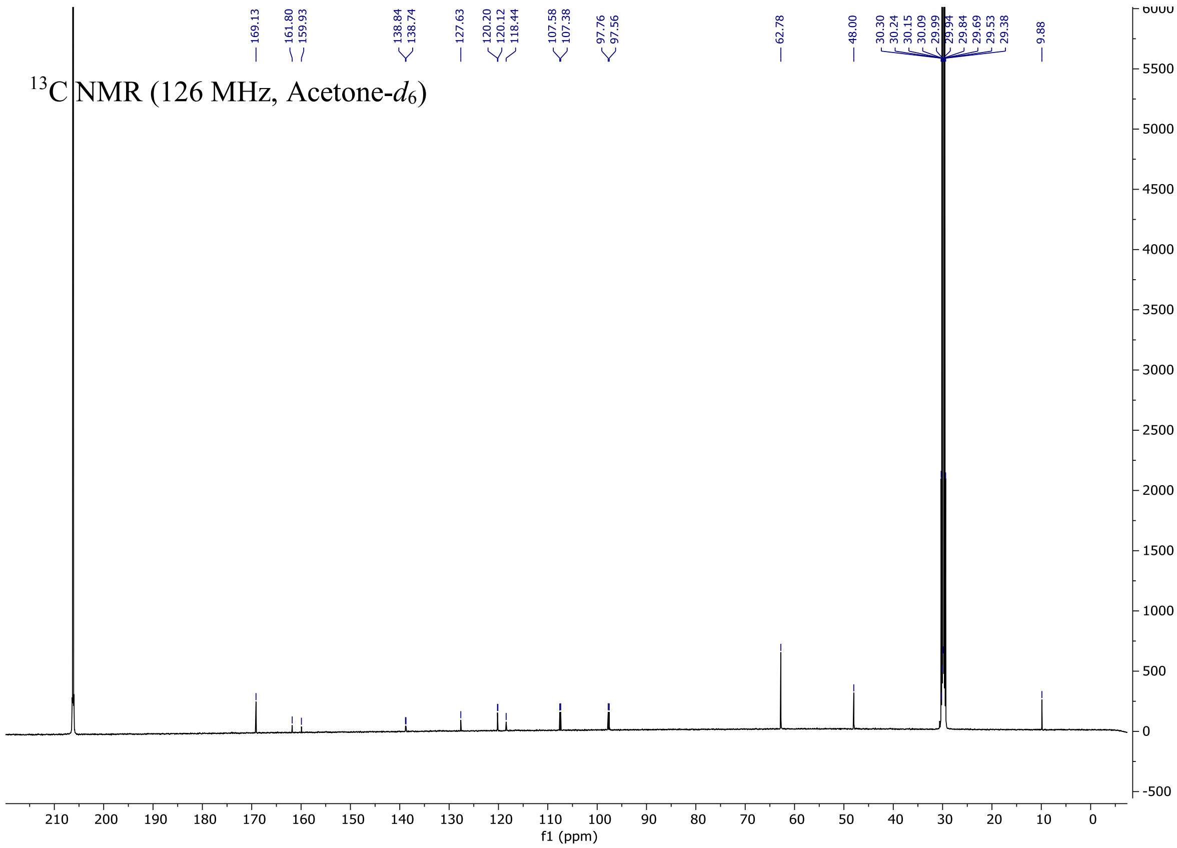

^{13}C NMR (126 MHz, Acetone-d_6)
169.13 161.80 159.93 138.84 138.74 127.63 120.20 120.12 118.44 107.58 107.38 97.76 97.56 62.78 48.00 30.30 30.24 30.15 30.09 29.99 29.94 29.84 29.69 29.53 29.38 9.88
f1 (ppm)

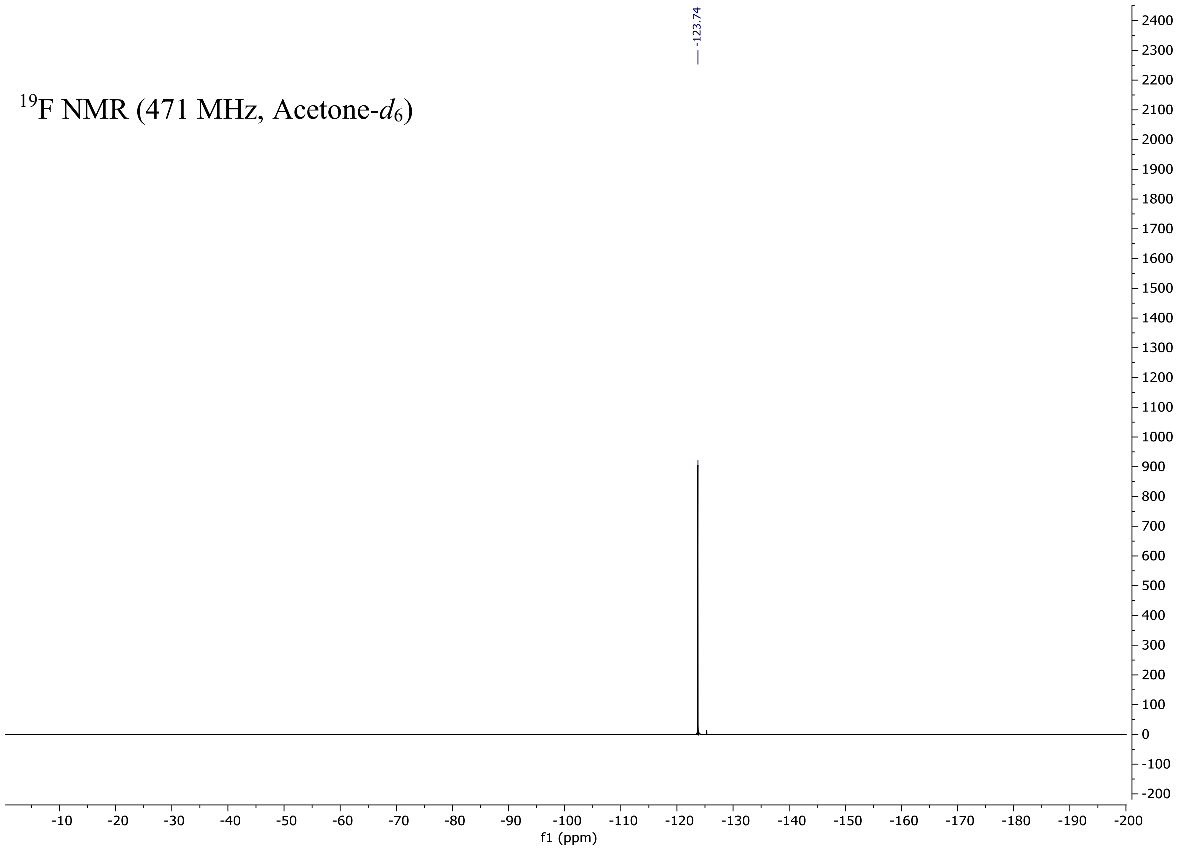

19F NMR (471 MHz, Acetone-d6)
-123.74
f1 (ppm)

Compound 11:

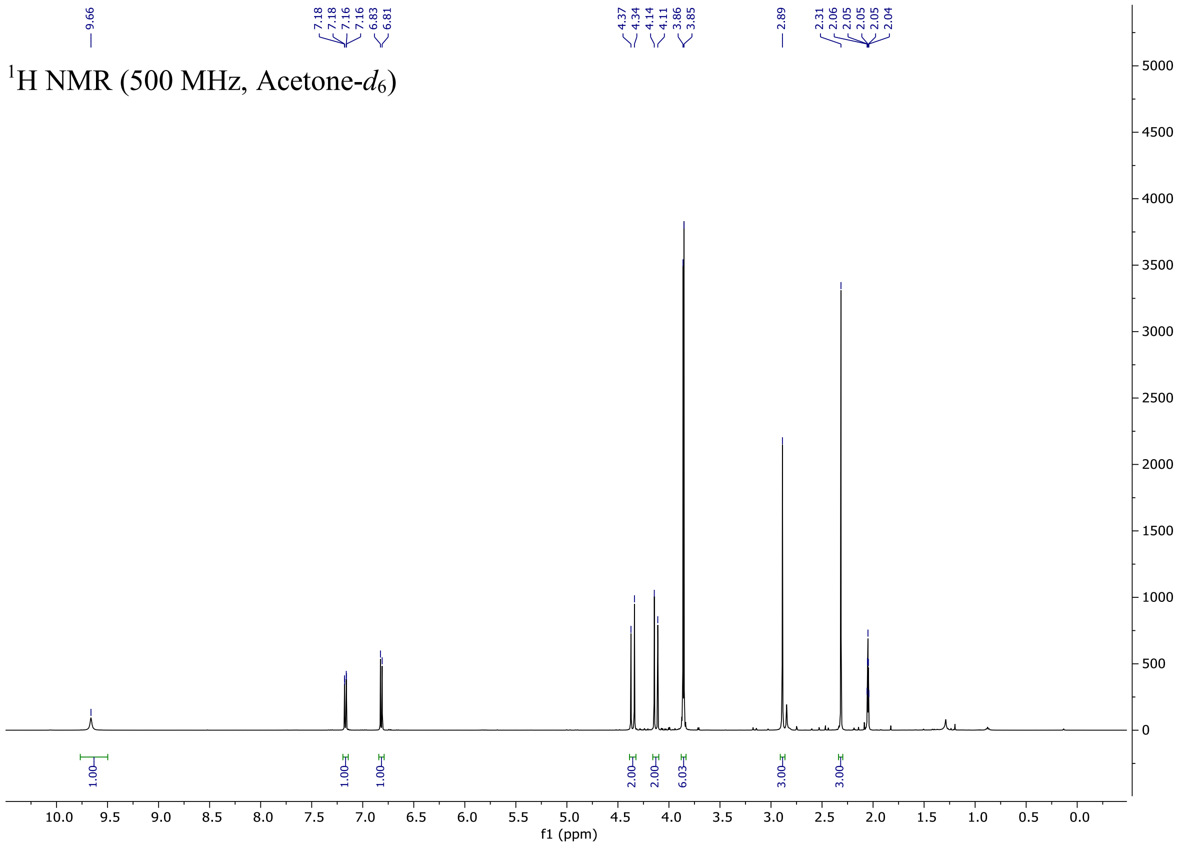
1H NMR (500 MHz, Acetone-d6)
9.66
7.18 7.18 7.16 7.16 6.83 6.81
4.37 4.34 4.14 4.11 3.86 3.85
2.89
2.31 2.06 2.05 2.05 2.05 2.04
1.00 1.00 1.00 2.00 2.00 6.03 3.00 3.00
f1 (ppm)

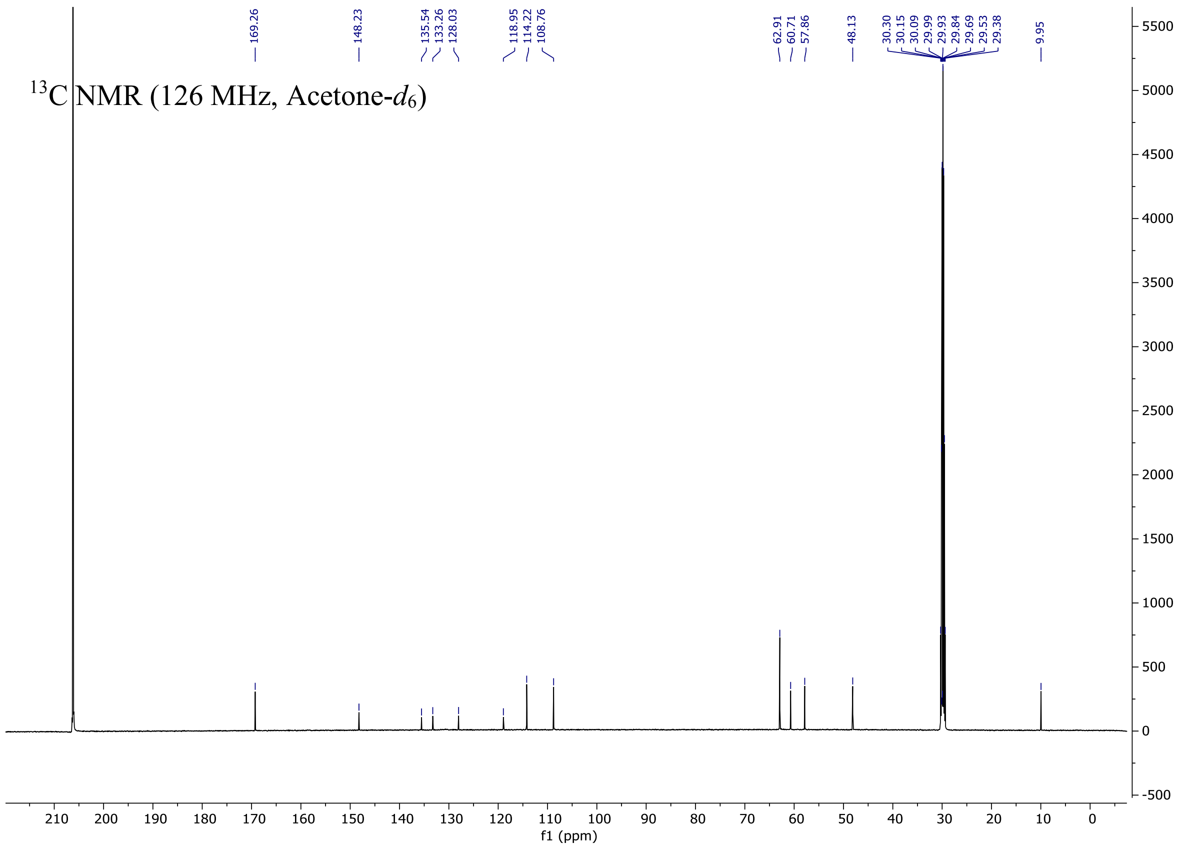
13C NMR (126 MHz, Acetone-d6)
169.26
148.23
135.54 133.26 128.03
118.95 114.22 108.76
62.91 60.71 57.86
48.13
30.30 30.15 30.09 29.99 29.93 29.84 29.69 29.53 29.38
9.95
f1 (ppm)

Compound 12:

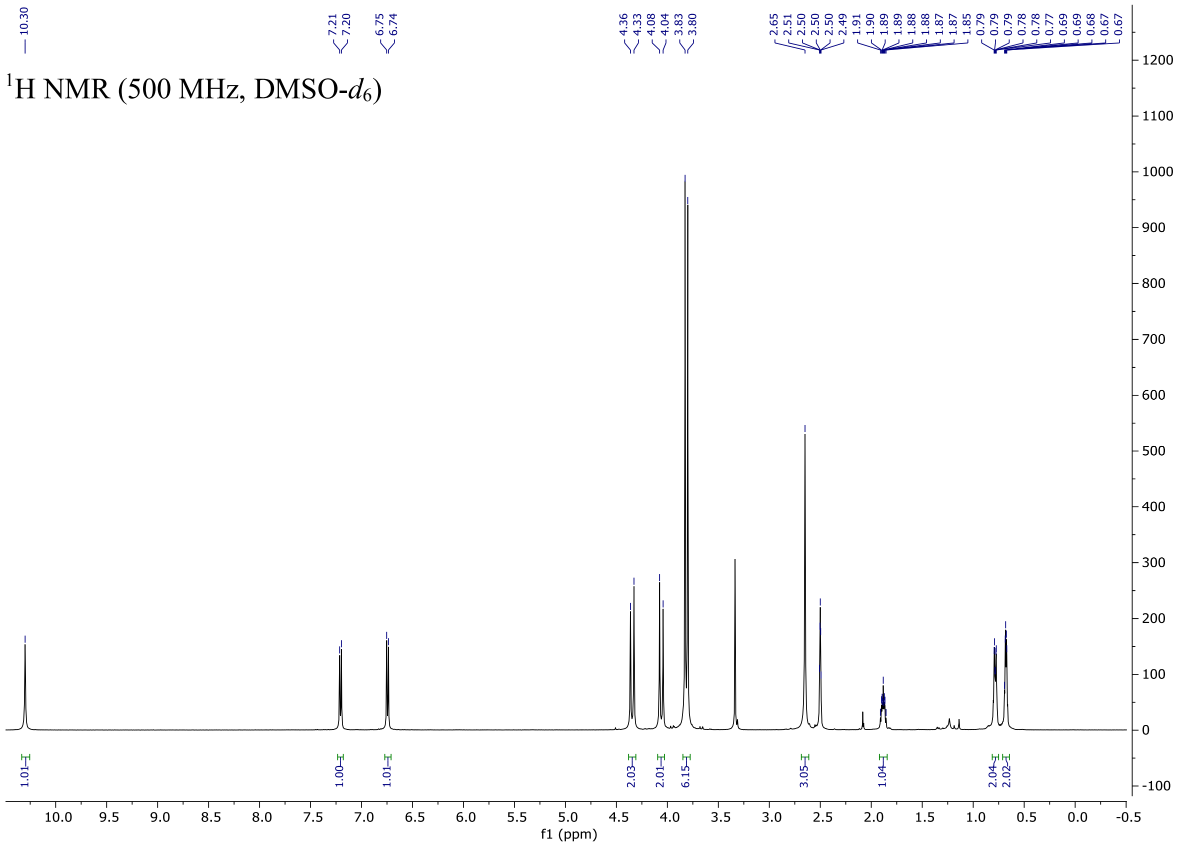

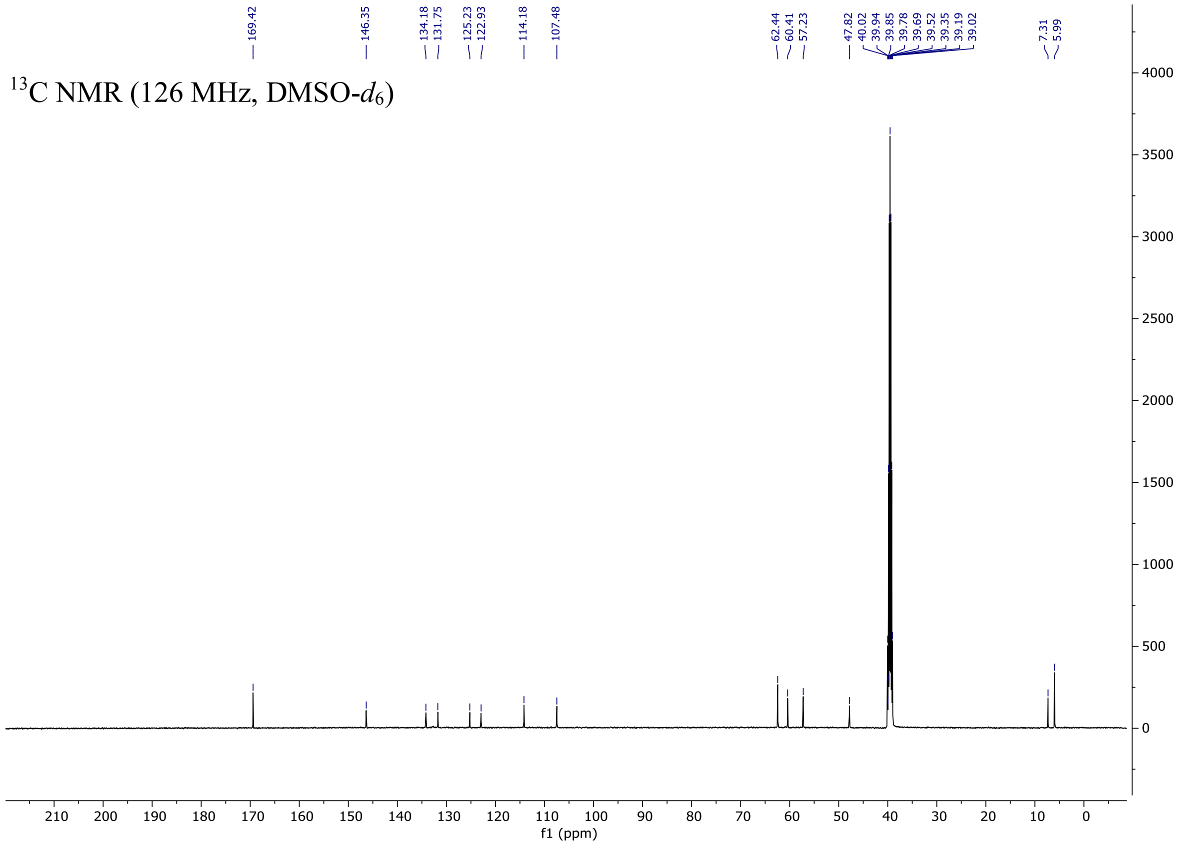

Compound 13:

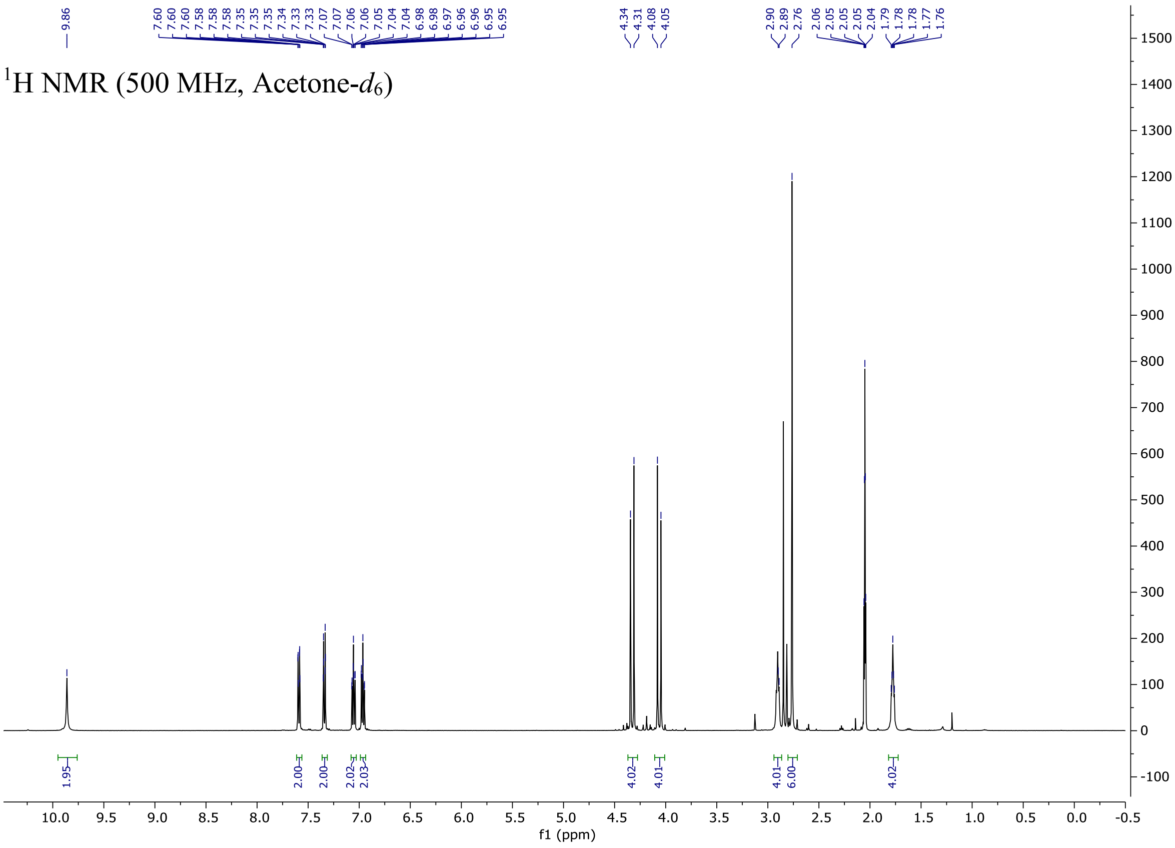

^{1}H NMR (500 MHz, Acetone-d_6)
f1 (ppm)

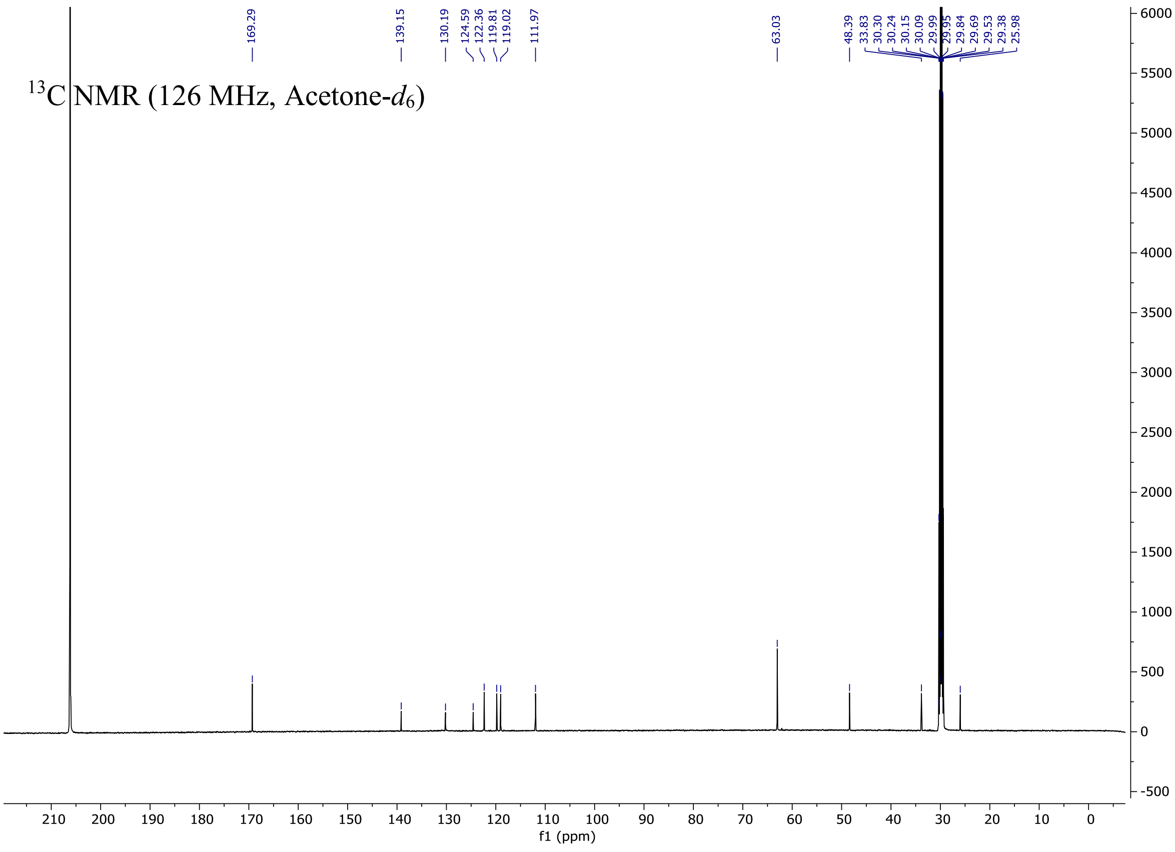

^{13}C NMR (126 MHz, Acetone-d_6)
f1 (ppm)

Compound 14:

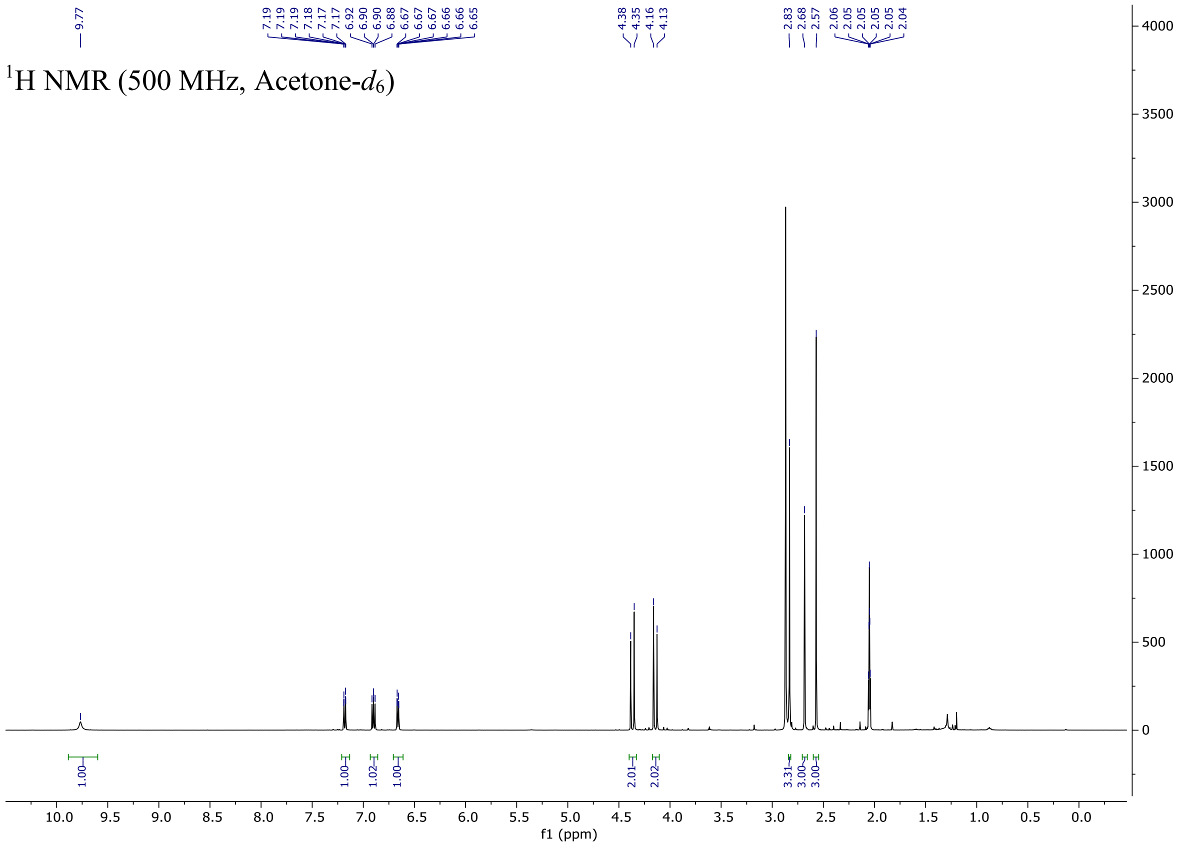

9.77
7.19
7.19
7.19
7.18
7.17
7.17
6.92
6.90
6.90
6.88
6.67
6.67
6.67
6.66
6.66
6.65
4.38
4.35
4.16
4.13
2.83
2.68
2.57
2.06
2.05
2.05
2.05
2.05
2.04
¹H NMR (500 MHz, Acetone-d6)
1.00
1.00
1.02
1.00
2.01
2.02
3.31
3.00
3.00
f1 (ppm)

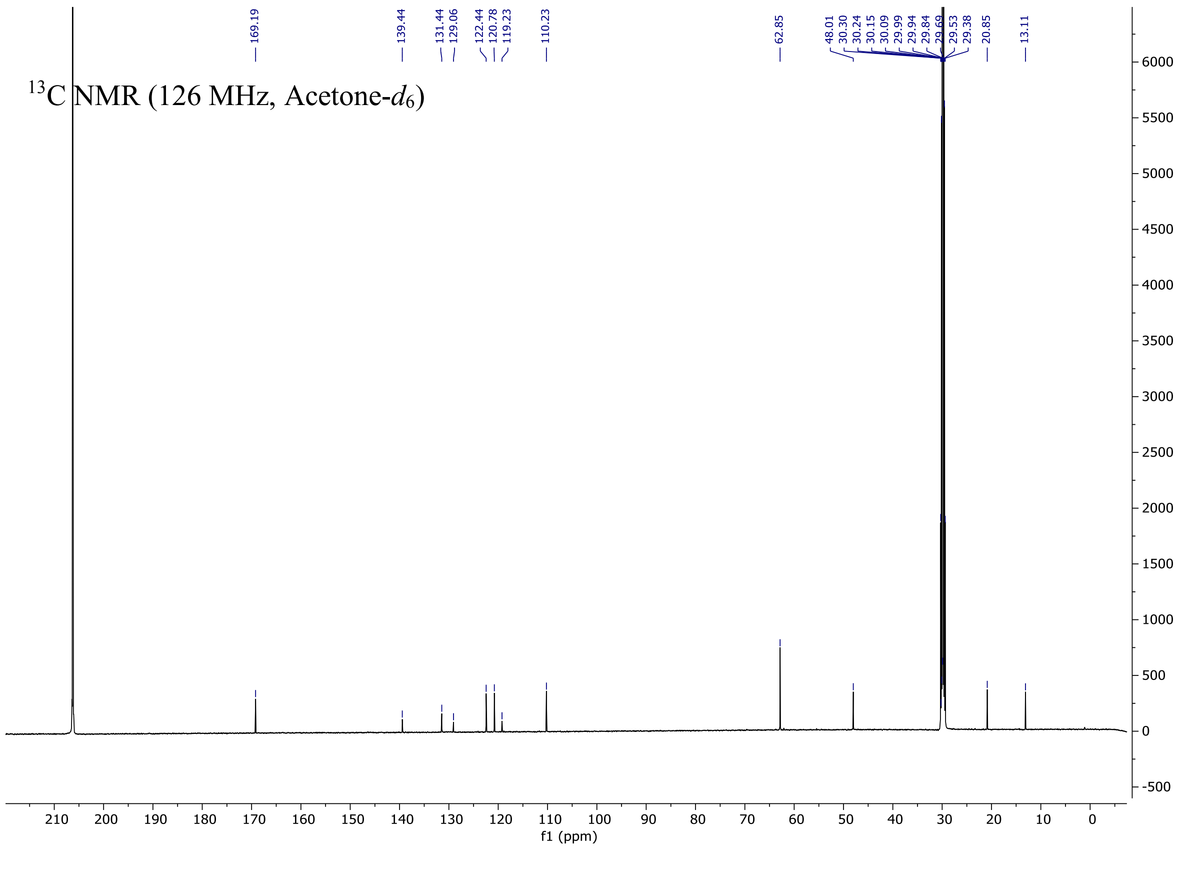

169.19
139.44
131.44
129.06
122.44
120.78
119.23
110.23
62.85
48.01
30.30
30.24
30.15
30.09
29.99
29.94
29.84
29.69
29.53
29.38
20.85
13.11
¹³C NMR (126 MHz, Acetone-d6)
f1 (ppm)

Compound 15:

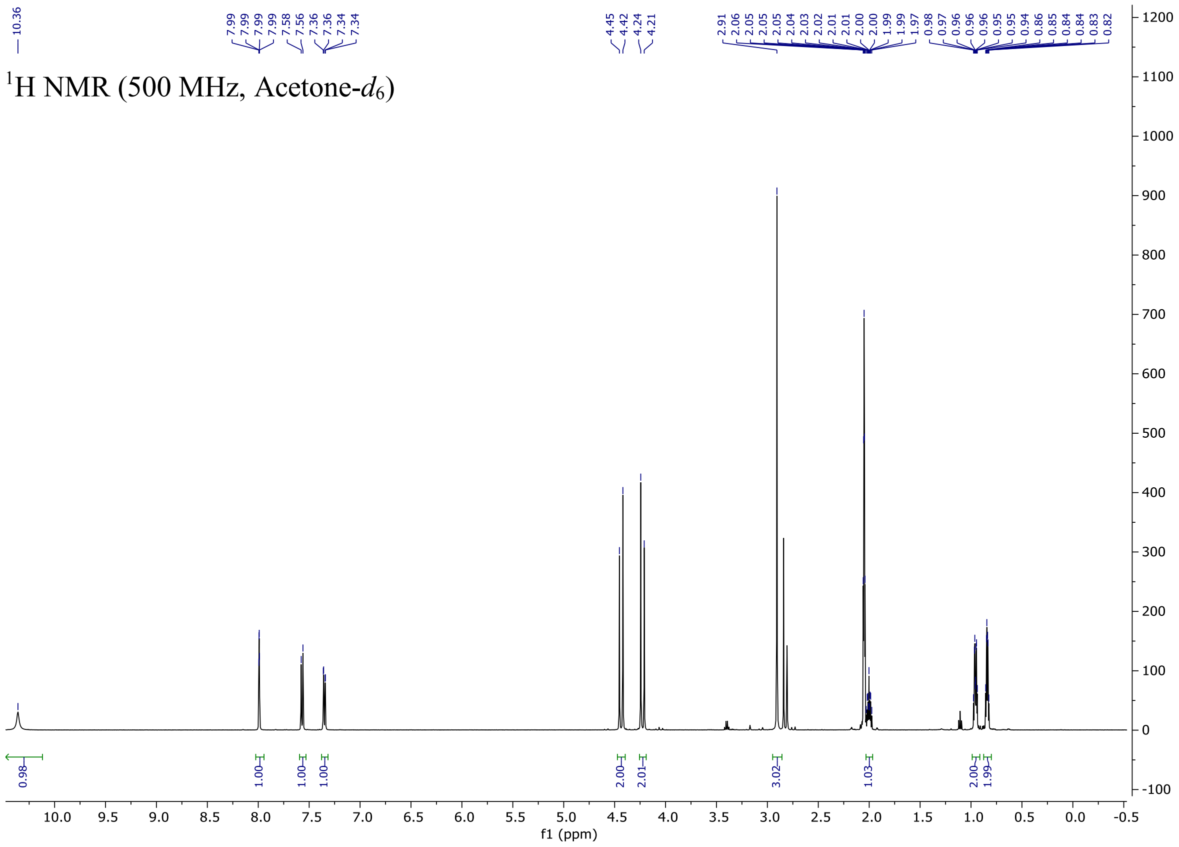
1H NMR (500 MHz, Acetone-d6)
10.36
7.99
7.99
7.99
7.99
7.58
7.56
7.36
7.36
7.34
7.34
4.45
4.42
4.24
4.21
2.91
2.06
2.05
2.05
2.05
2.04
2.03
2.02
2.01
2.01
2.00
2.00
1.99
1.99
1.97
0.98
0.97
0.96
0.96
0.96
0.95
0.95
0.94
0.86
0.85
0.84
0.84
0.83
0.82
0.98
1.00
1.00
1.00
2.00
2.01
3.02
1.03
2.00
1.99
f1 (ppm)

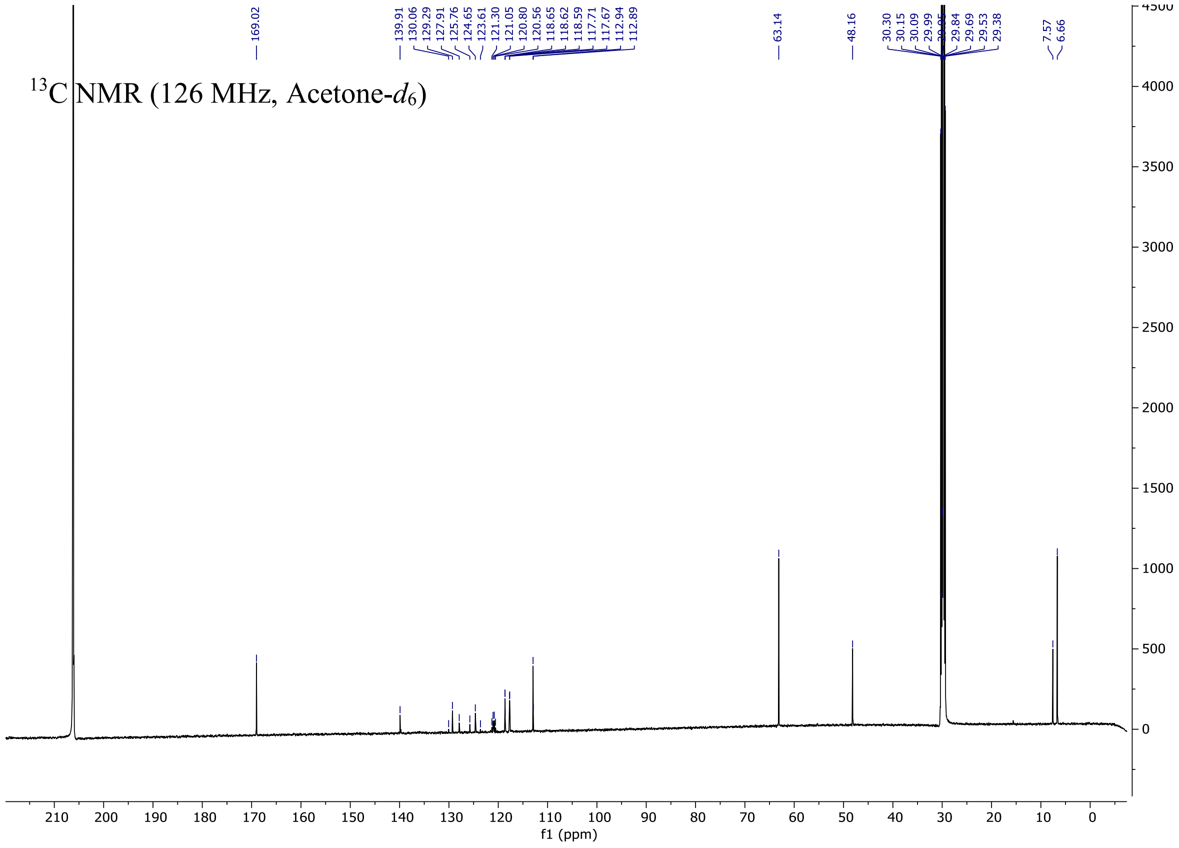
13C NMR (126 MHz, Acetone-d6)
169.02
139.91
130.06
129.29
127.91
125.76
124.65
123.61
121.30
121.05
120.80
120.56
118.65
118.62
118.59
117.71
117.67
112.94
112.89
63.14
48.16
30.30
30.15
30.09
29.99
29.84
29.69
29.53
29.38
7.57
6.66
f1 (ppm)

^{19}F NMR (376 MHz, Acetone-d_6)

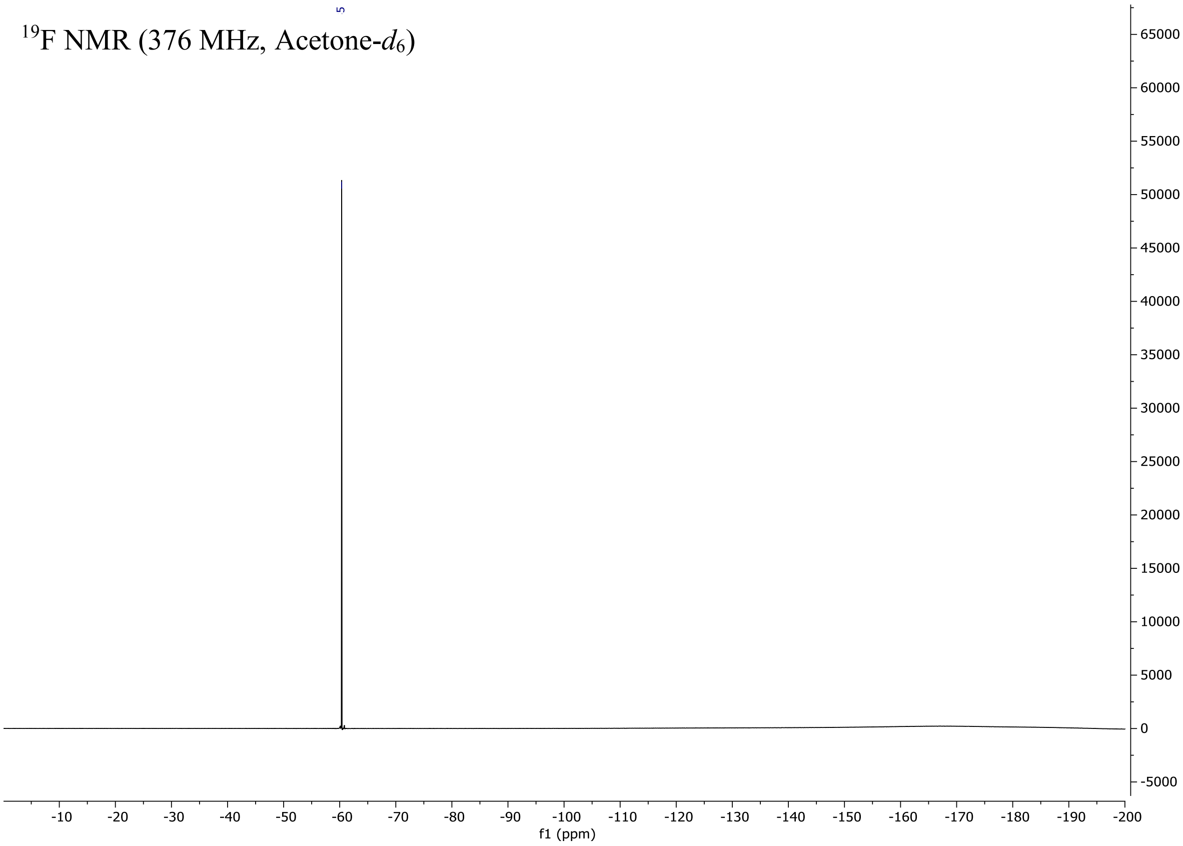

Compound 16:

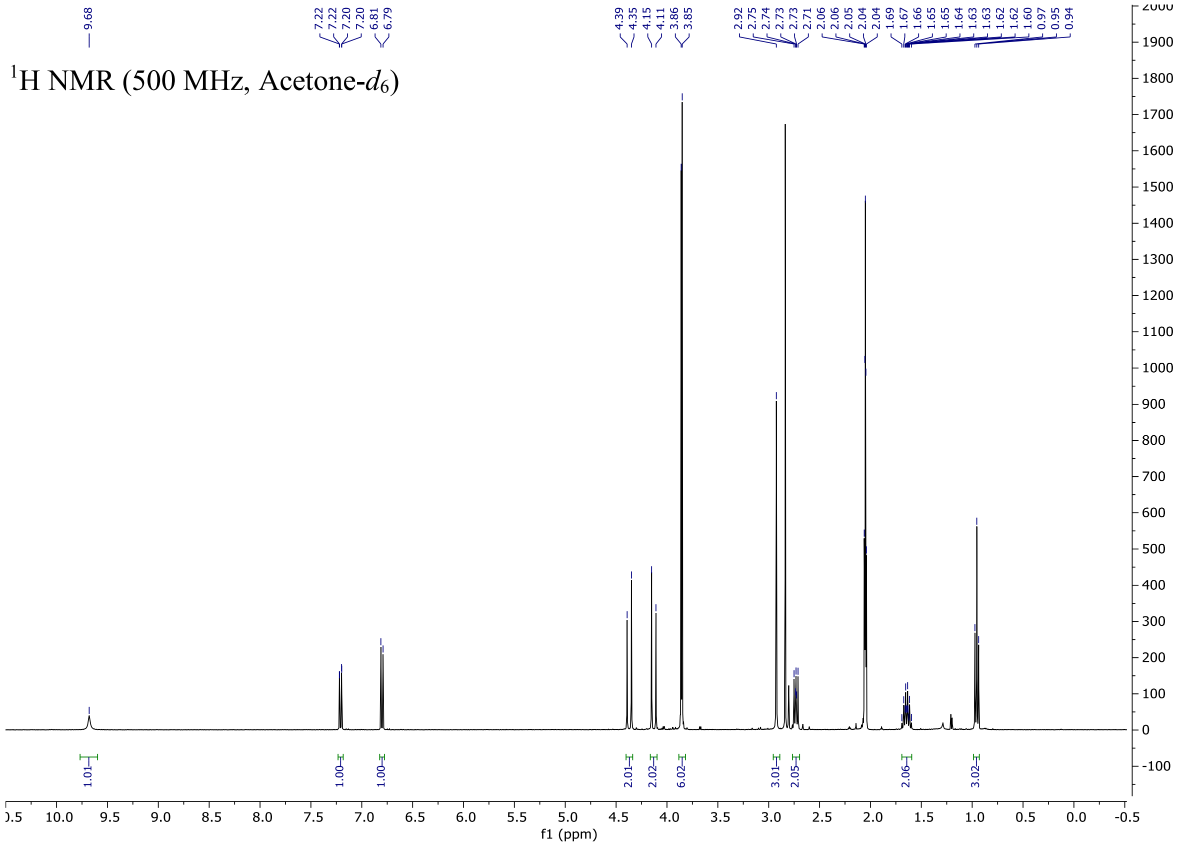

¹H NMR (500 MHz, Acetone-d6)
f1 (ppm)

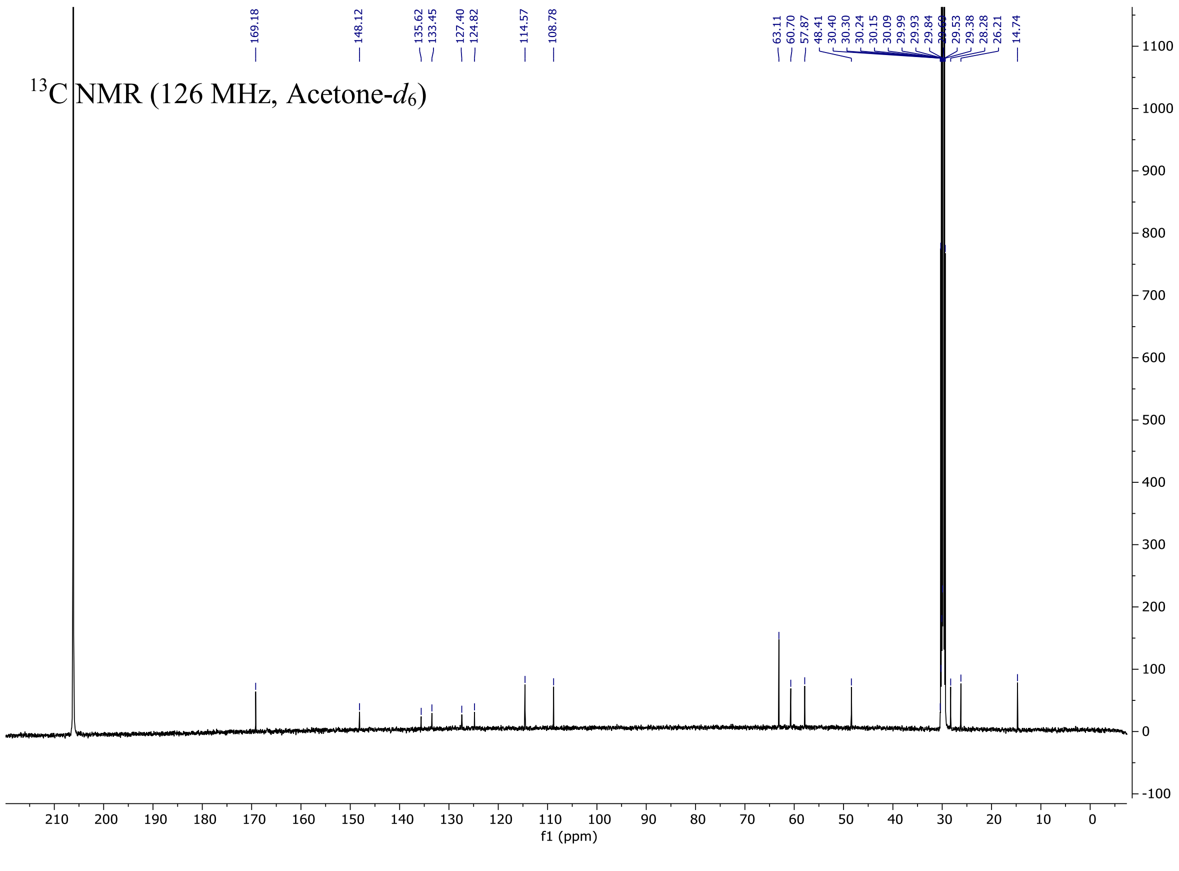

¹³C NMR (126 MHz, Acetone-d6)
f1 (ppm)

Compound 17:

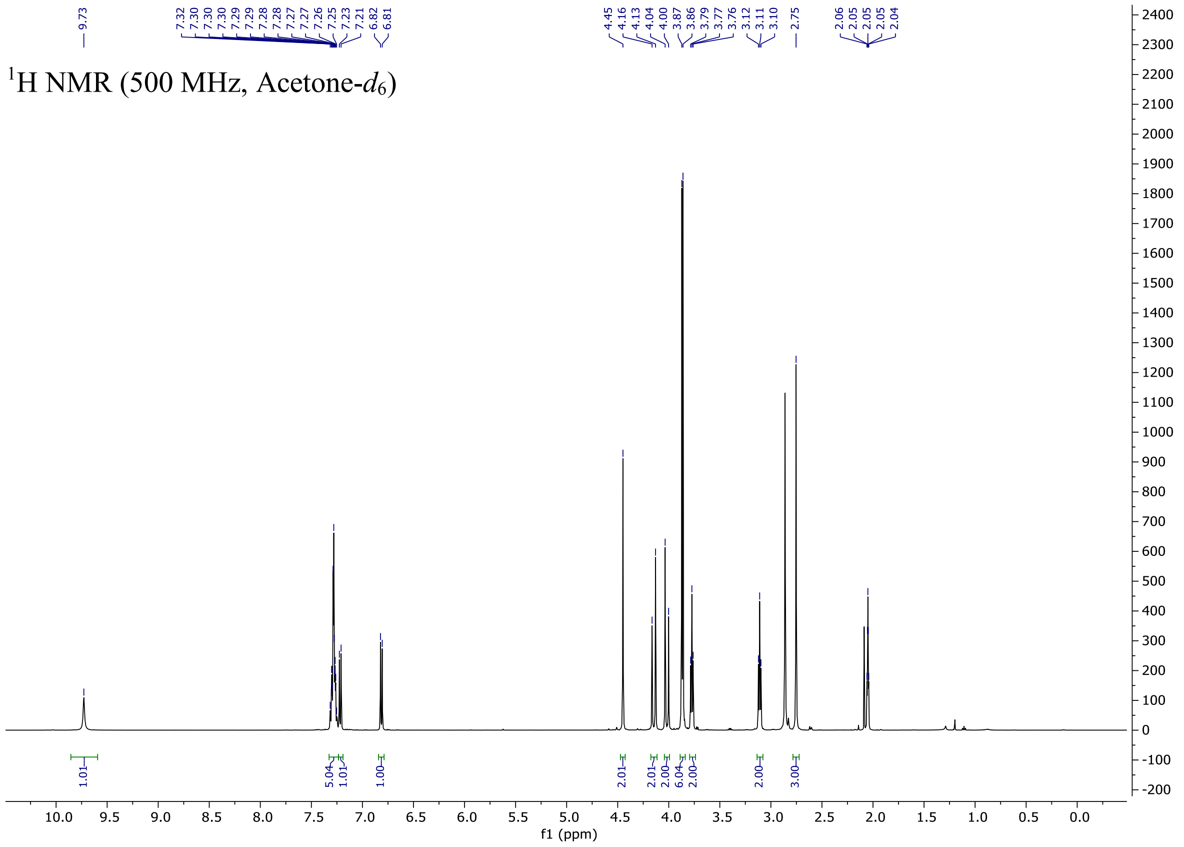

^{1}H NMR (500 MHz, Acetone-d_6)
f1 (ppm)

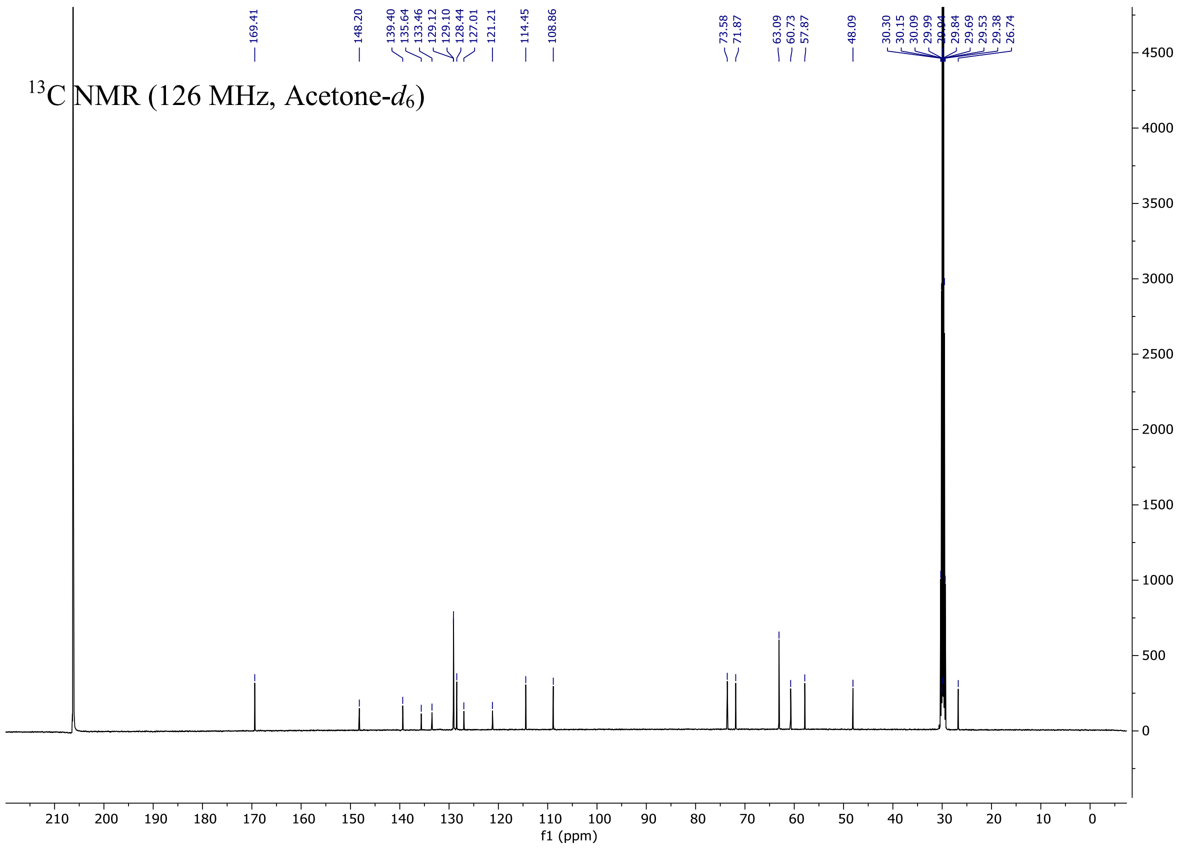

^{13}C NMR (126 MHz, Acetone-d_6)
f1 (ppm)

Compound 18:

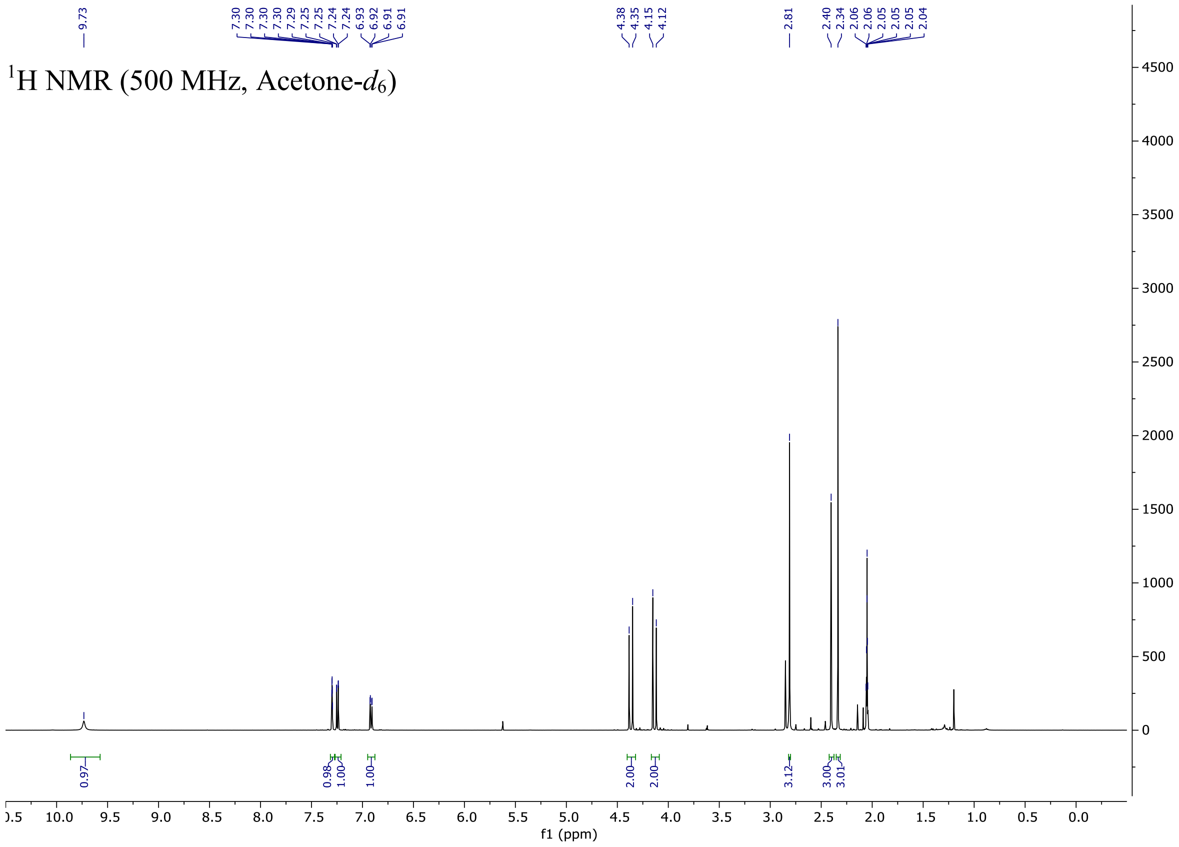

^{1}H NMR (500 MHz, Acetone-d_6)
9.73
7.30 7.30 7.30 7.30 7.29 7.25 7.25 7.24 7.24 6.93 6.92 6.91 6.91
4.38 4.35 4.15 4.12
2.81
2.40 2.34 2.06 2.05 2.05 2.05 2.04
0.97 0.98 1.00 1.00 2.00 2.00 3.12 3.00 3.01
f1 (ppm)

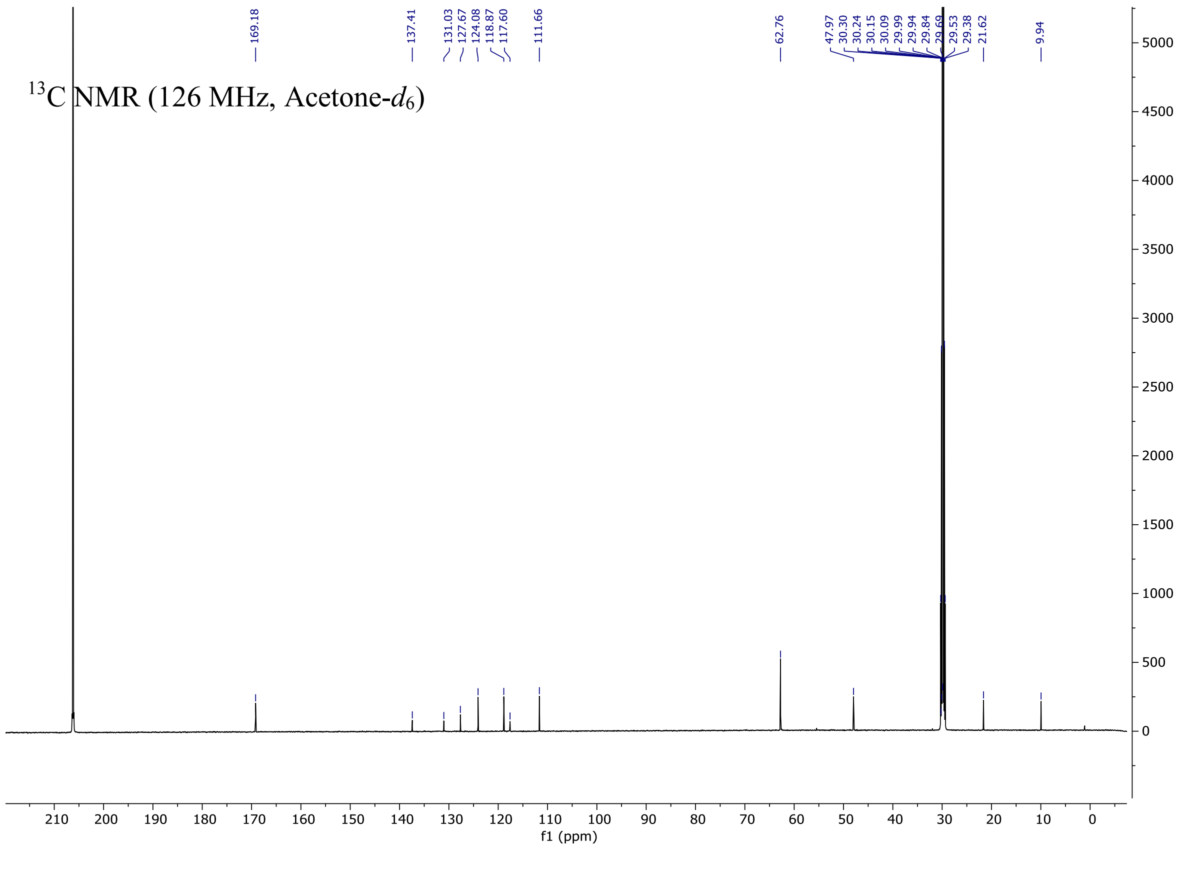

^{13}C NMR (126 MHz, Acetone-d_6)
169.18
137.41
131.03 127.67 124.08 118.87 117.60
111.66
62.76
47.97 30.30 30.24 30.15 30.09 29.99 29.94 29.84 29.69 29.53 29.38 21.62
9.94
f1 (ppm)

Compound 19:

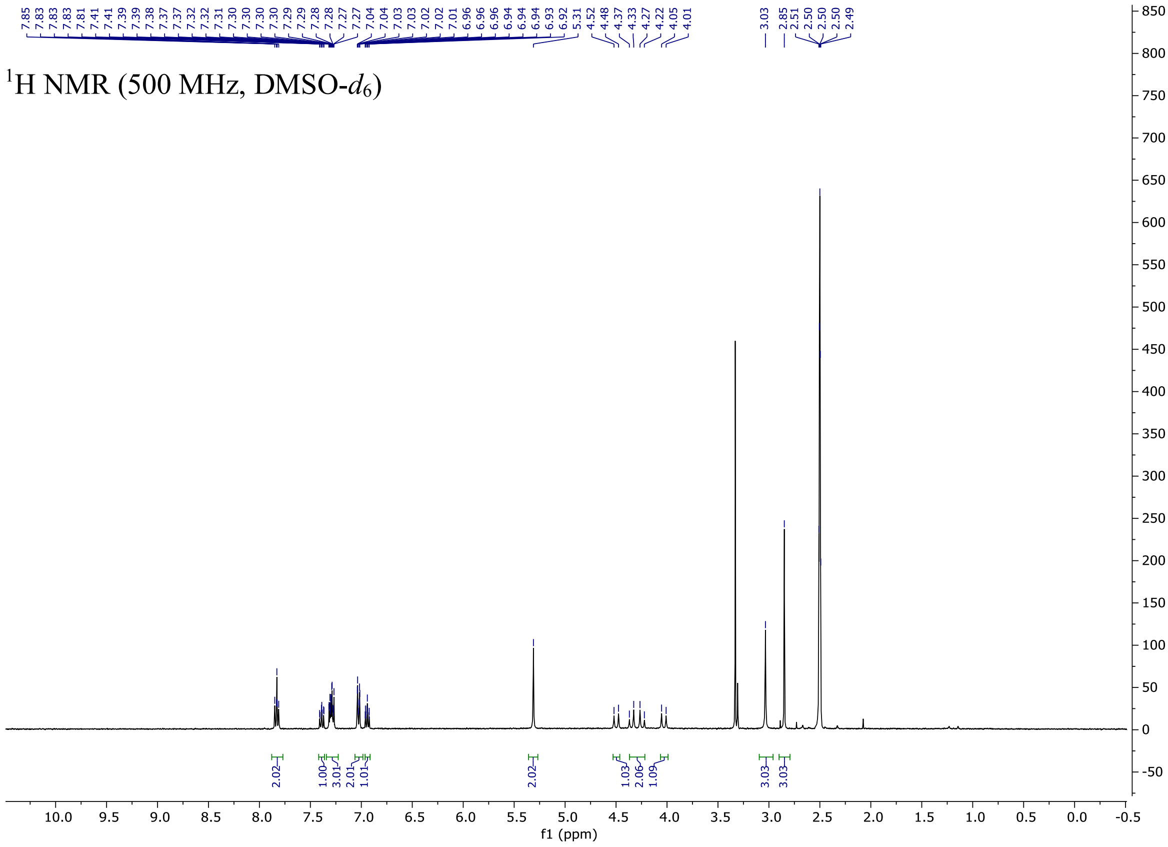

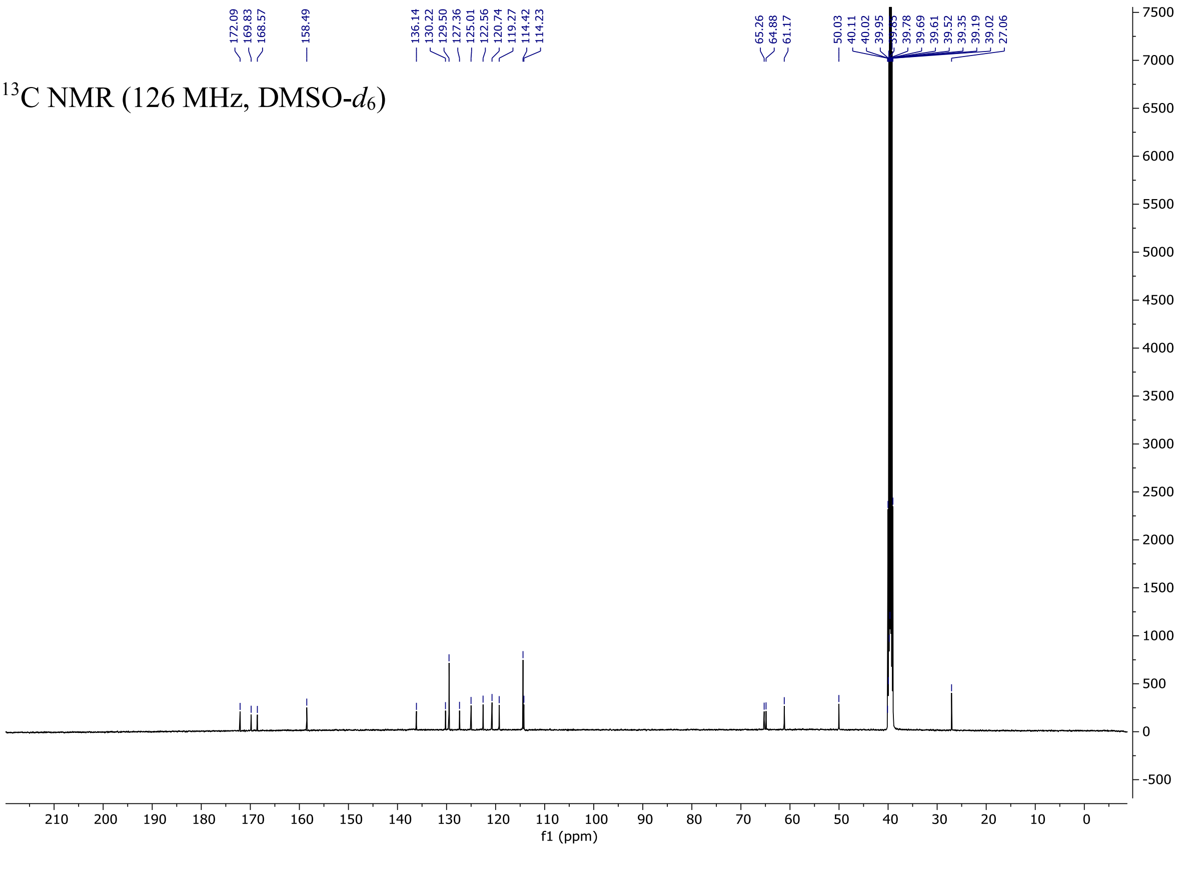

Compound 20:

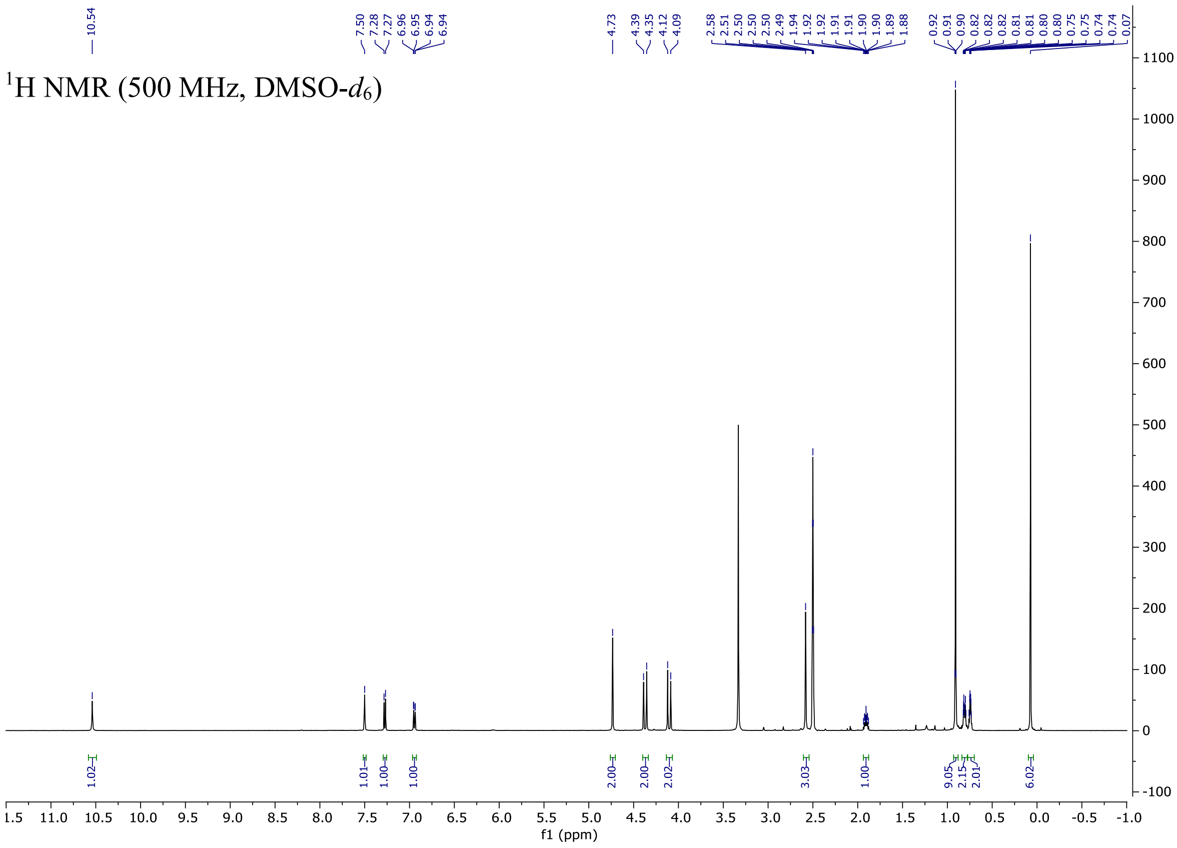

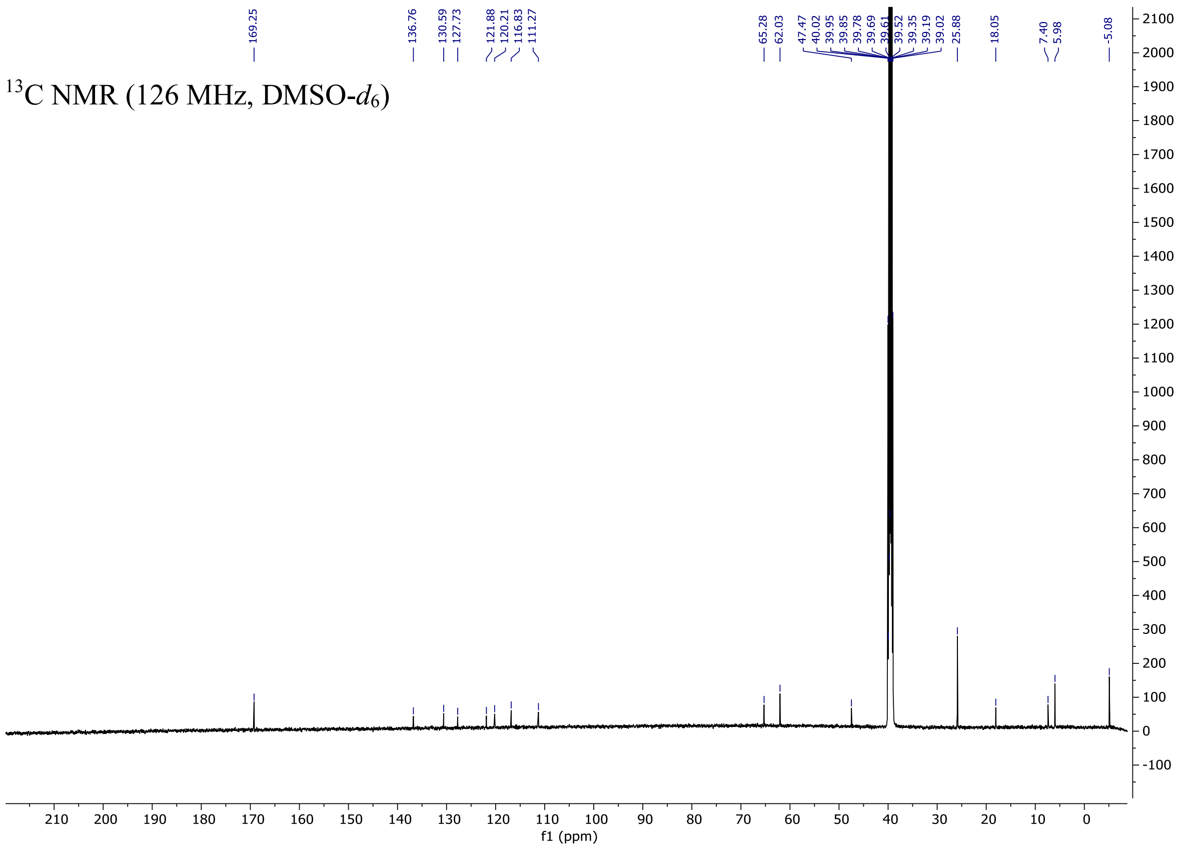

Compound 21:

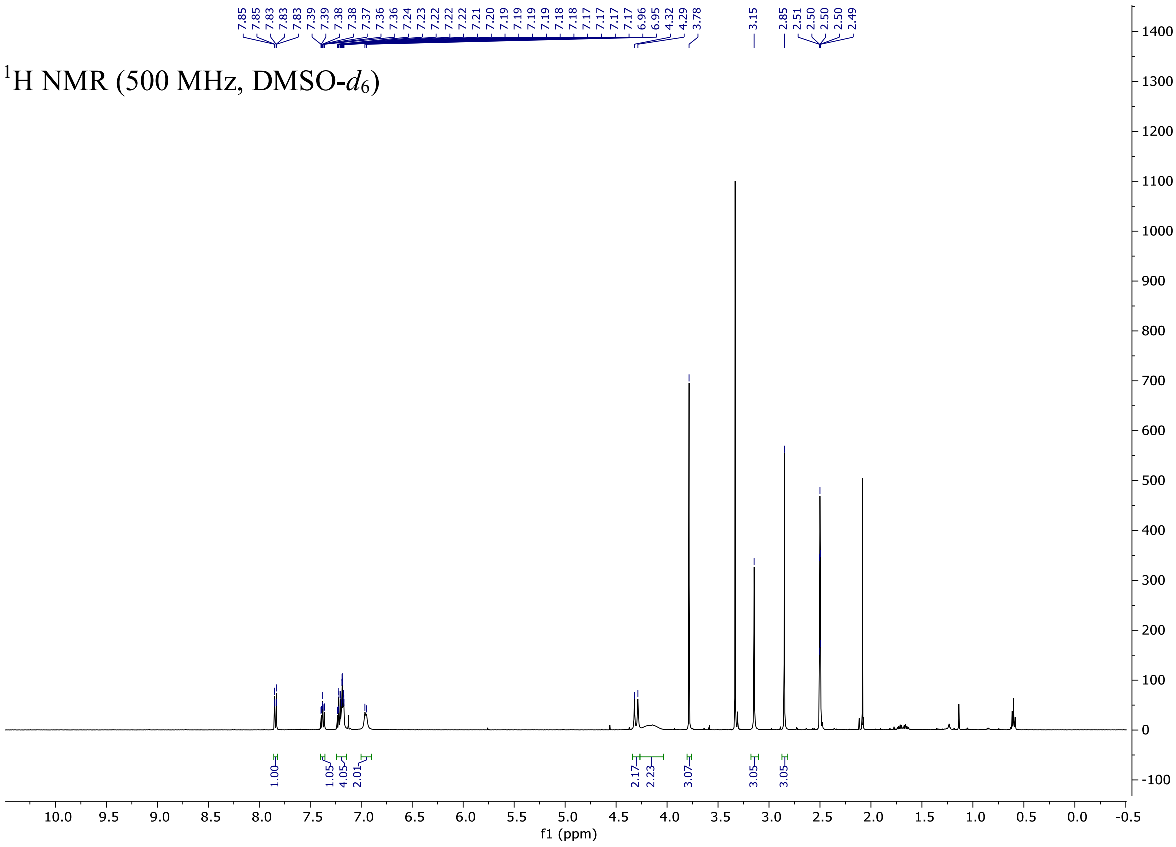

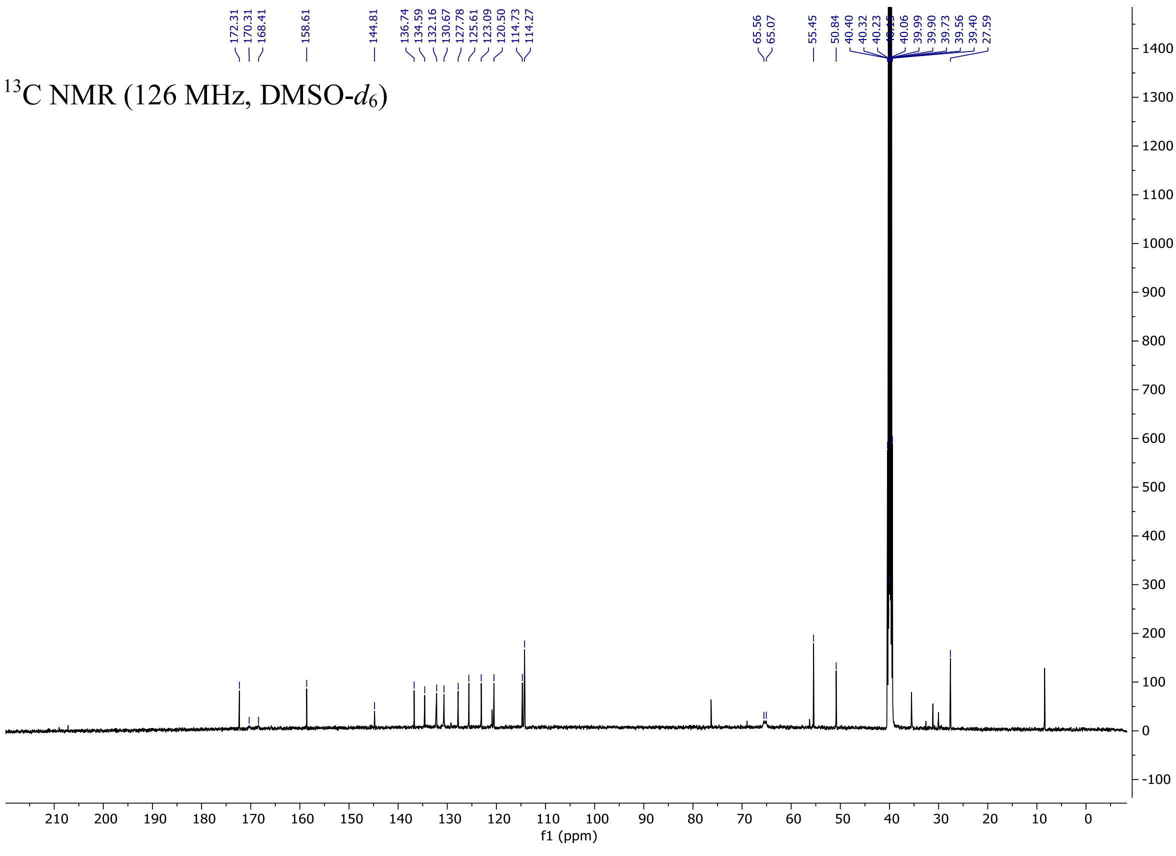

Compound 22:

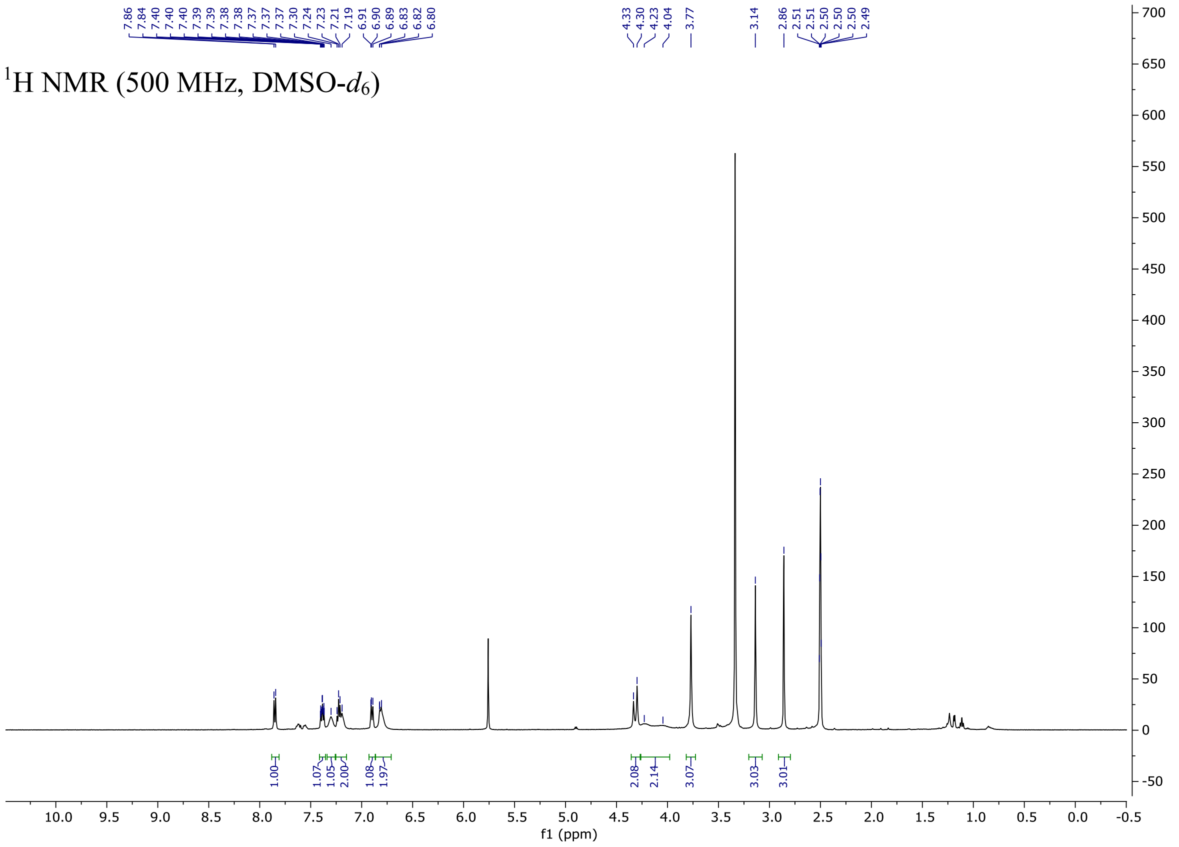

7.86
7.84
7.40
7.40
7.40
7.39
7.39
7.38
7.38
7.37
7.37
7.37
7.30
7.24
7.23
7.21
7.19
6.91
6.90
6.89
6.83
6.82
6.80
4.33
4.30
4.23
4.04
3.77
3.14
2.86
2.51
2.51
2.50
2.50
2.50
2.49
¹H NMR (500 MHz, DMSO-d6)
1.00
1.07
1.05
2.00
1.08
1.97
2.08
2.14
3.07
3.03
3.01
f1 (ppm)

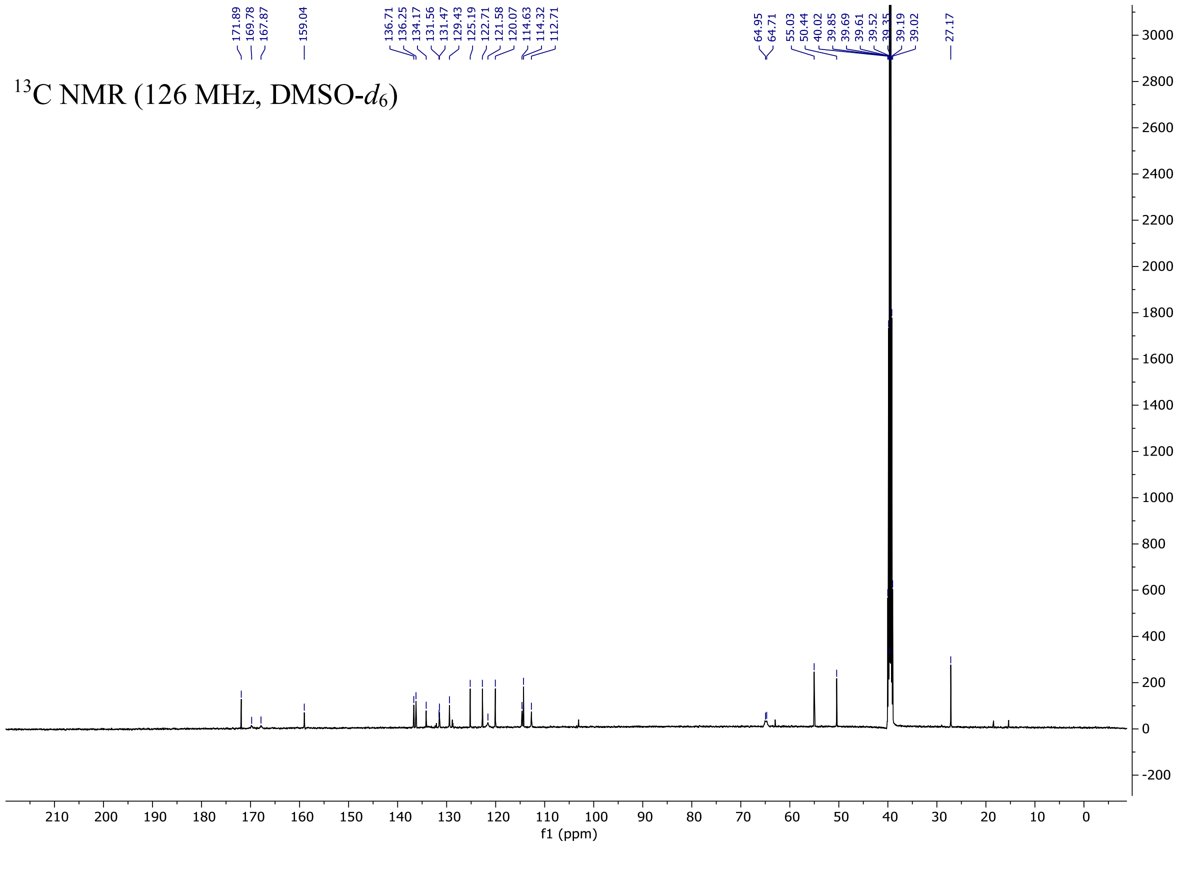

171.89
169.78
167.87
159.04
136.71
136.25
134.17
131.56
131.47
129.43
125.19
122.71
121.58
120.07
114.63
114.32
112.71
64.95
64.71
55.03
50.44
40.02
39.85
39.69
39.61
39.52
39.35
39.19
39.02
27.17
¹³C NMR (126 MHz, DMSO-d6)
f1 (ppm)

Compound 23:

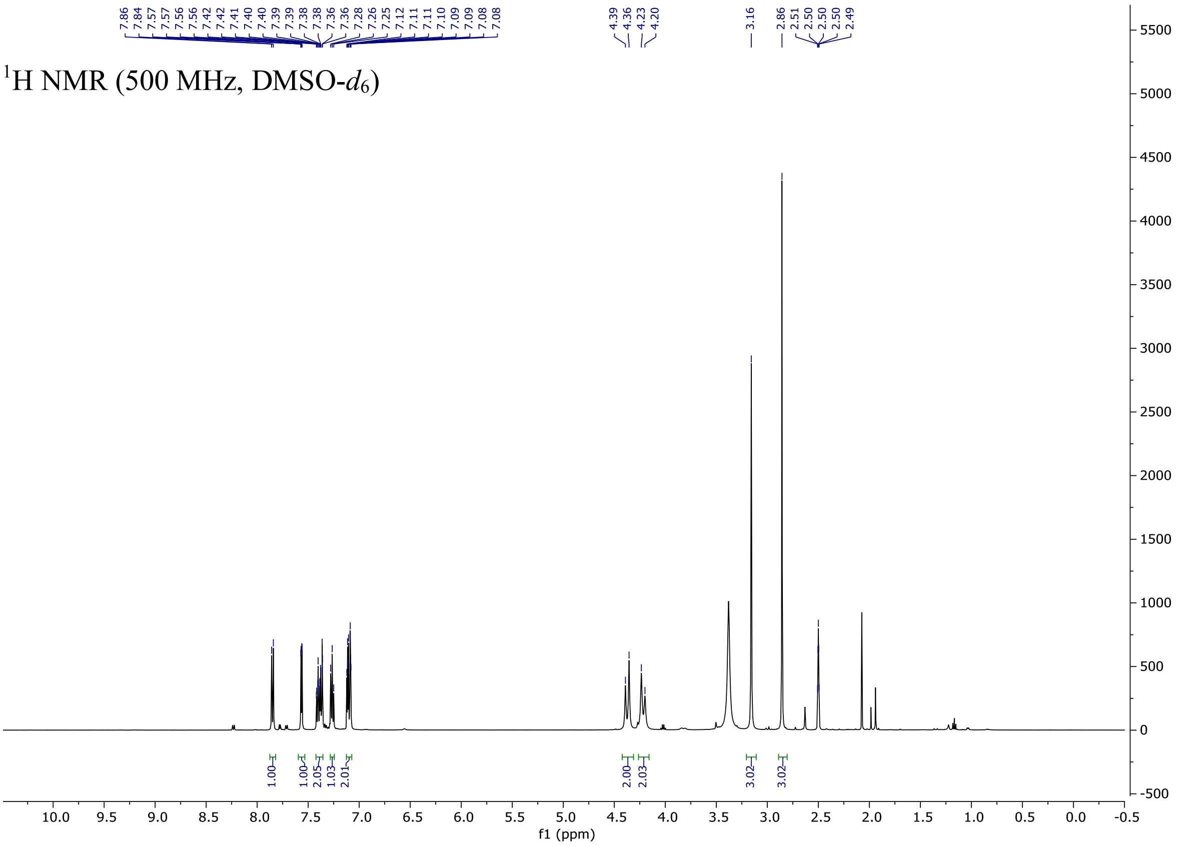

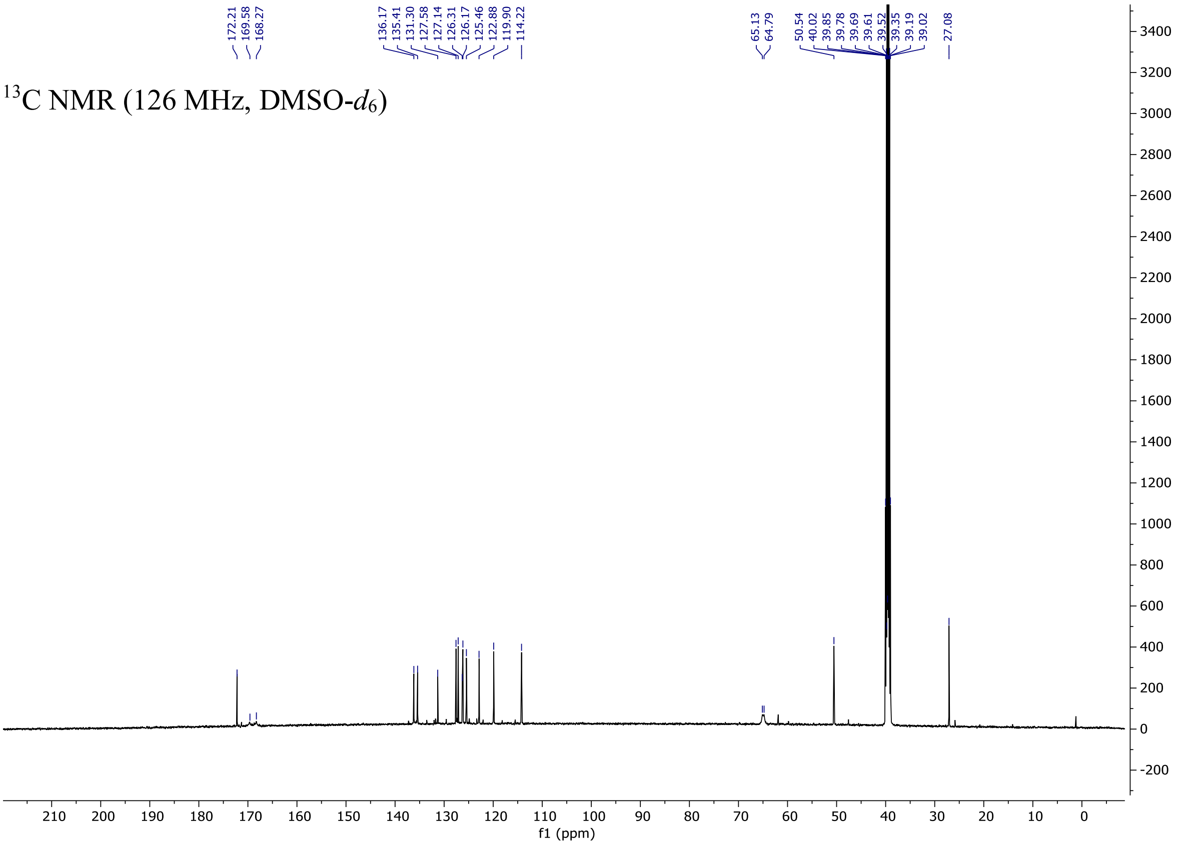

Compound 24:

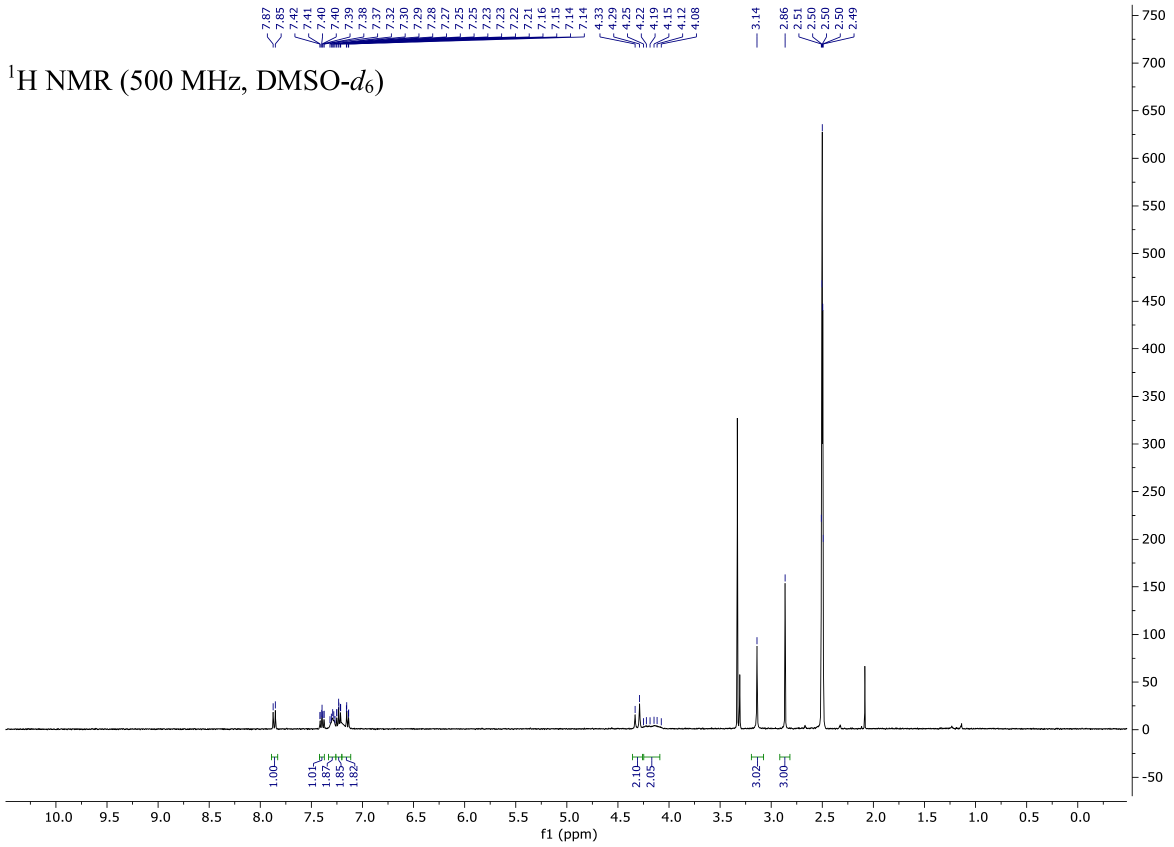

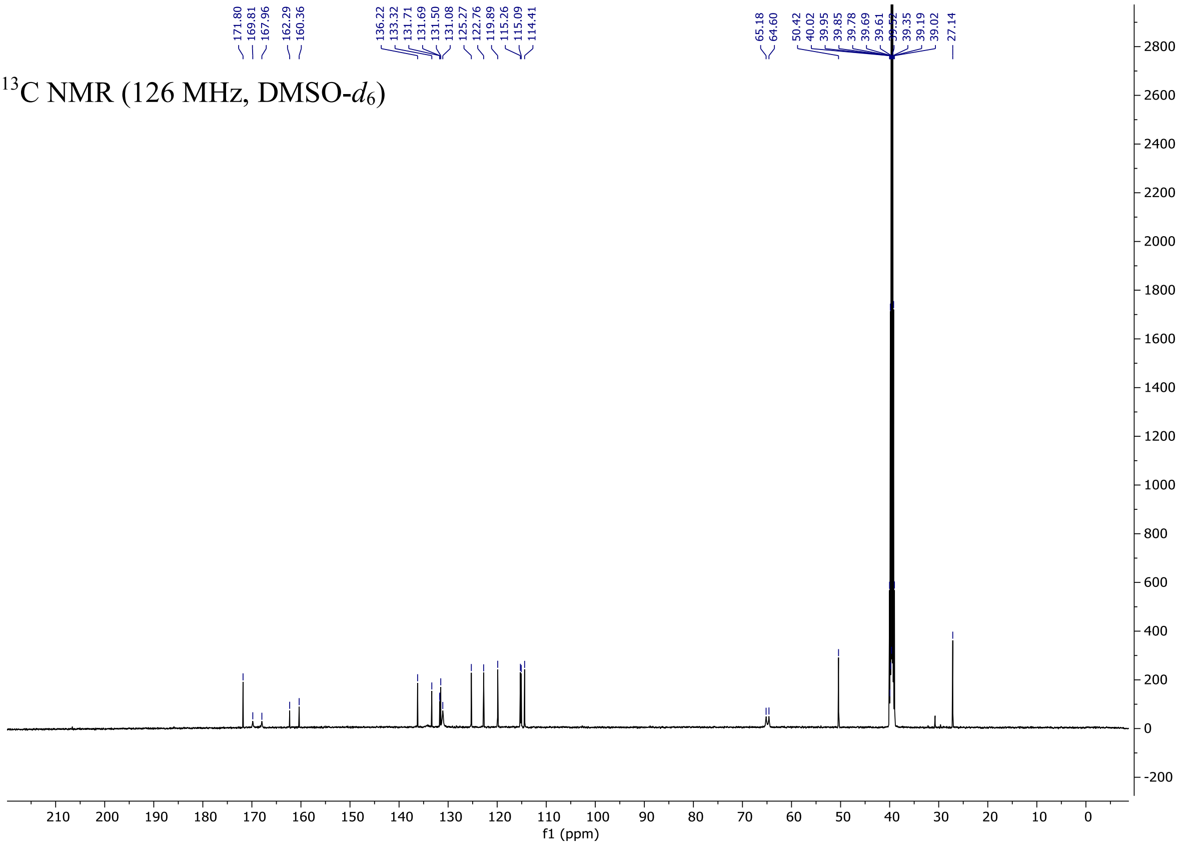

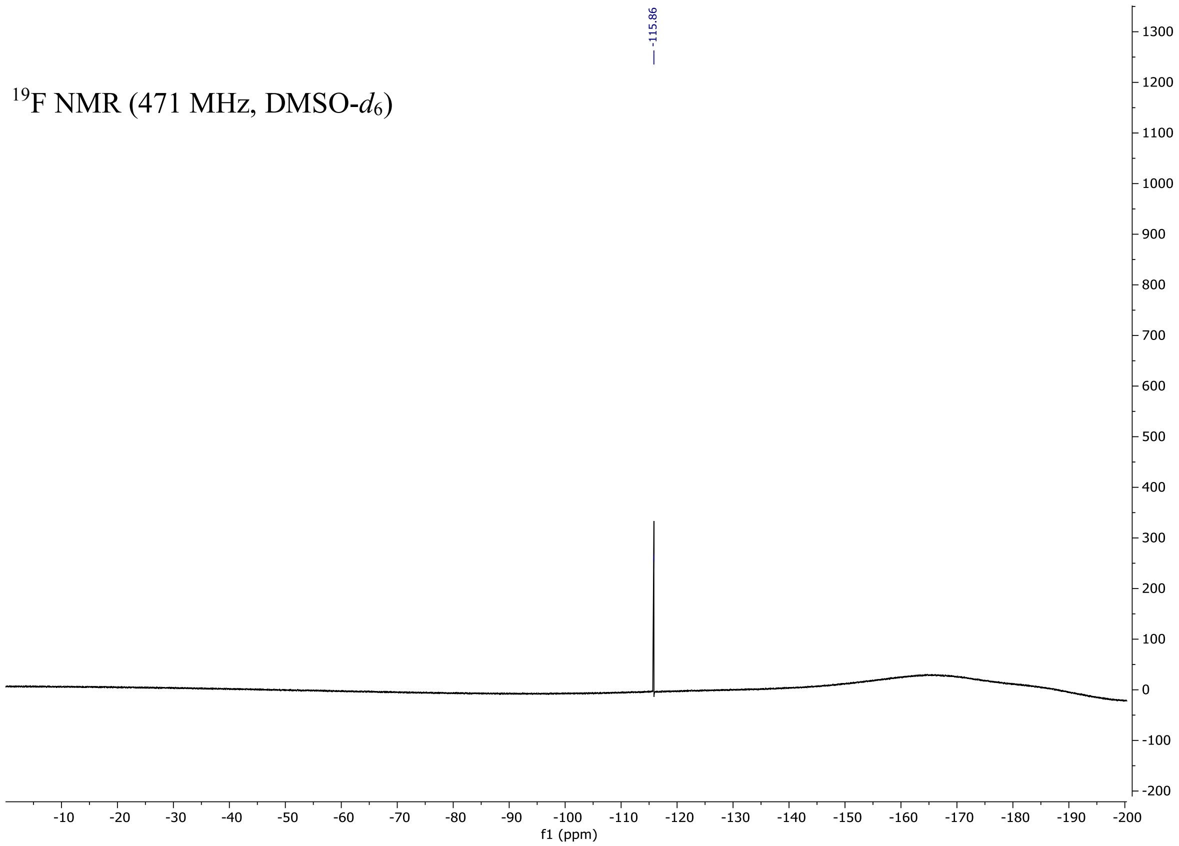

-115.86
19F NMR (471 MHz, DMSO-d6)
f1 (ppm)

Compound 25:

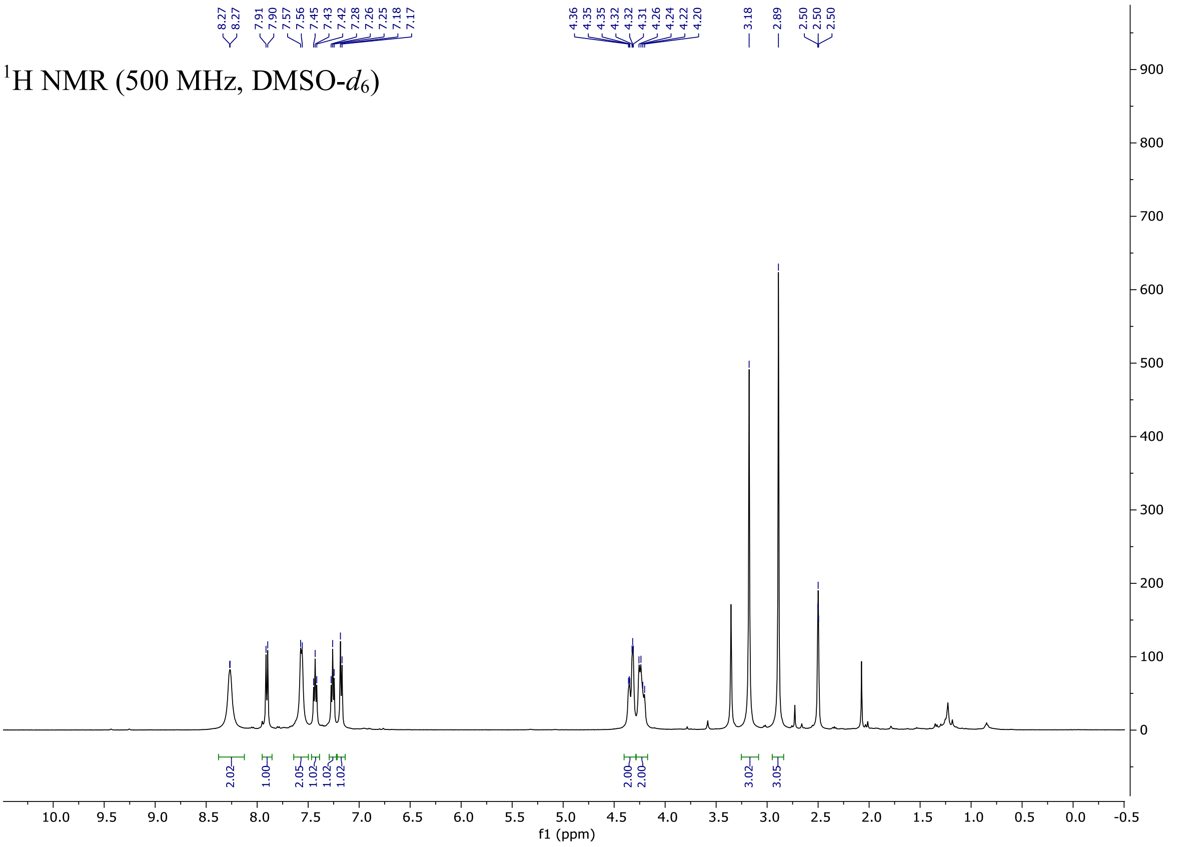

^{1}H NMR (500 MHz, DMSO-d_6)

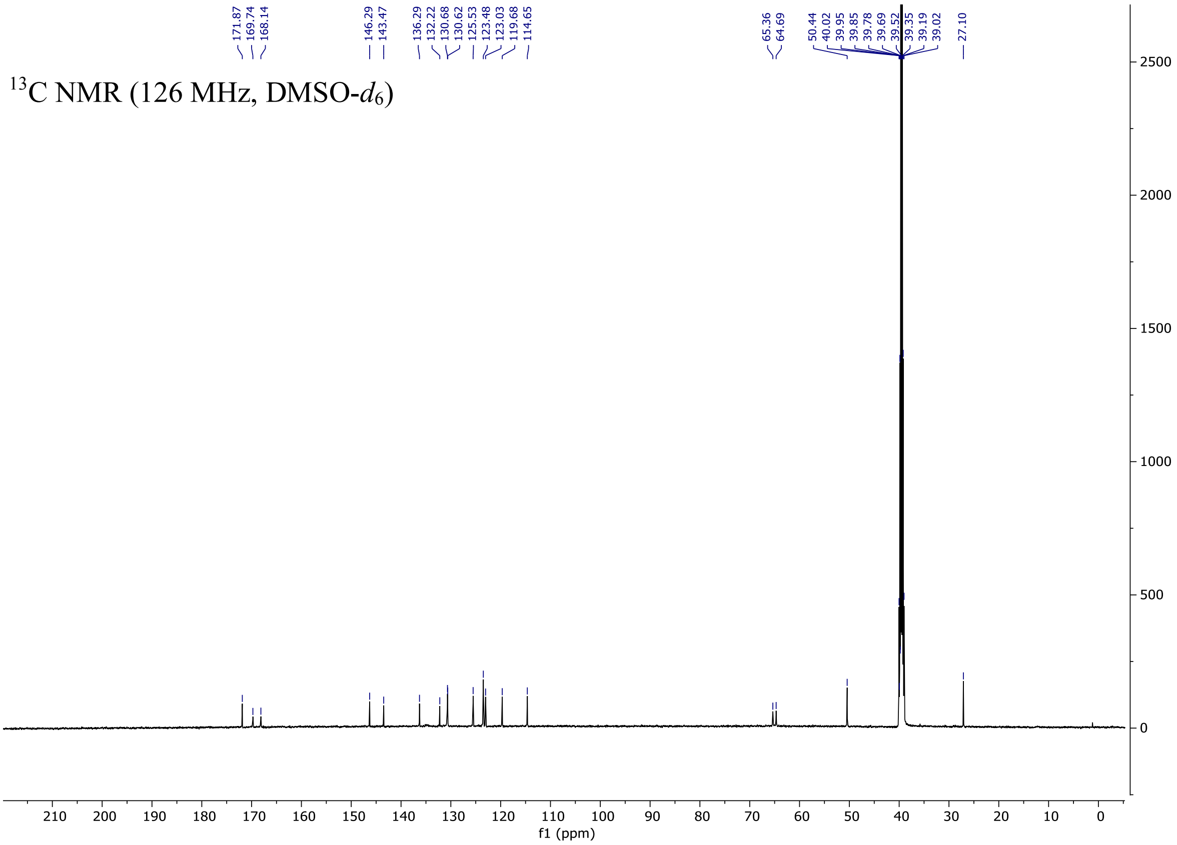

^{13}C NMR (126 MHz, DMSO-d_6)

Compound 26:

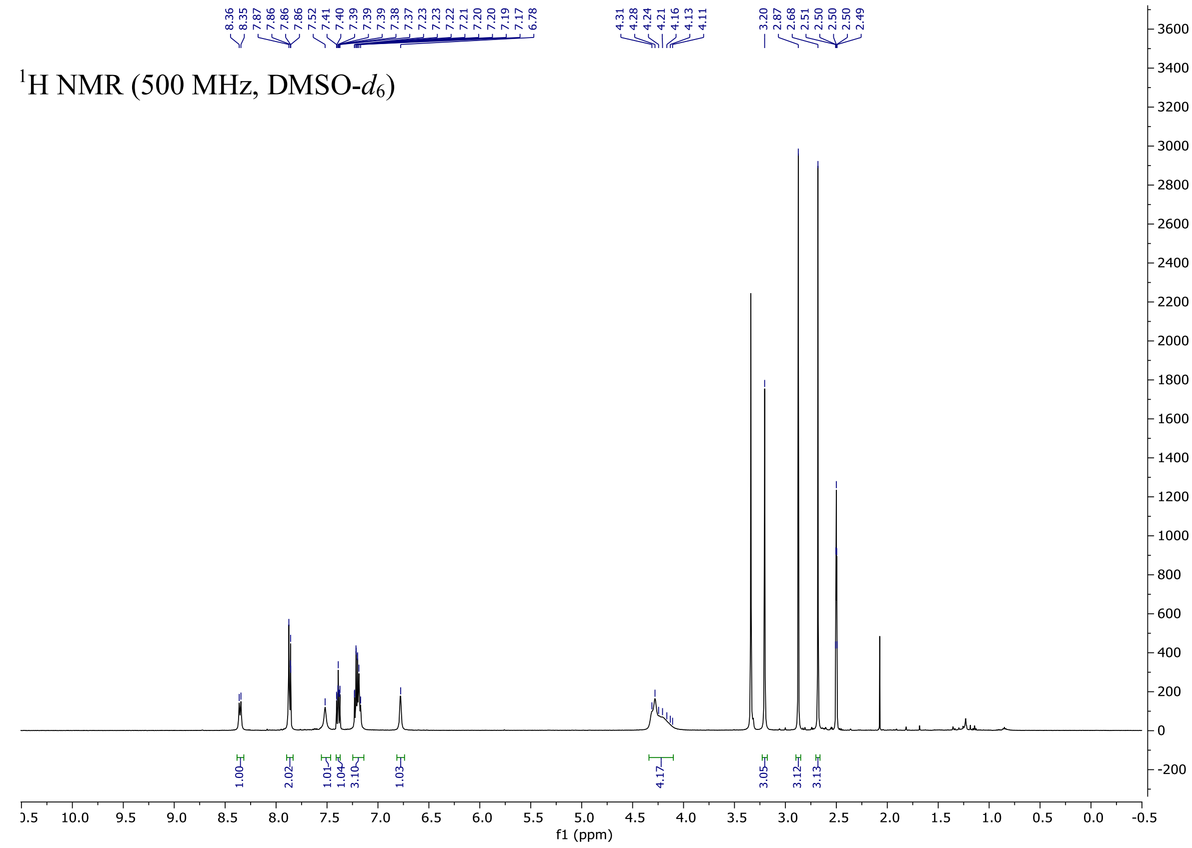
1H NMR (500 MHz, DMSO-d6)
8.36
8.35
7.87
7.86
7.86
7.86
7.52
7.41
7.40
7.39
7.39
7.39
7.38
7.37
7.23
7.23
7.22
7.21
7.20
7.19
7.17
6.78
4.31
4.28
4.24
4.21
4.16
4.13
4.11
3.20
2.87
2.68
2.51
2.50
2.50
2.50
2.49
1.00
2.02
1.01
1.04
3.10
1.03
4.17
3.05
3.12
3.13
f1 (ppm)

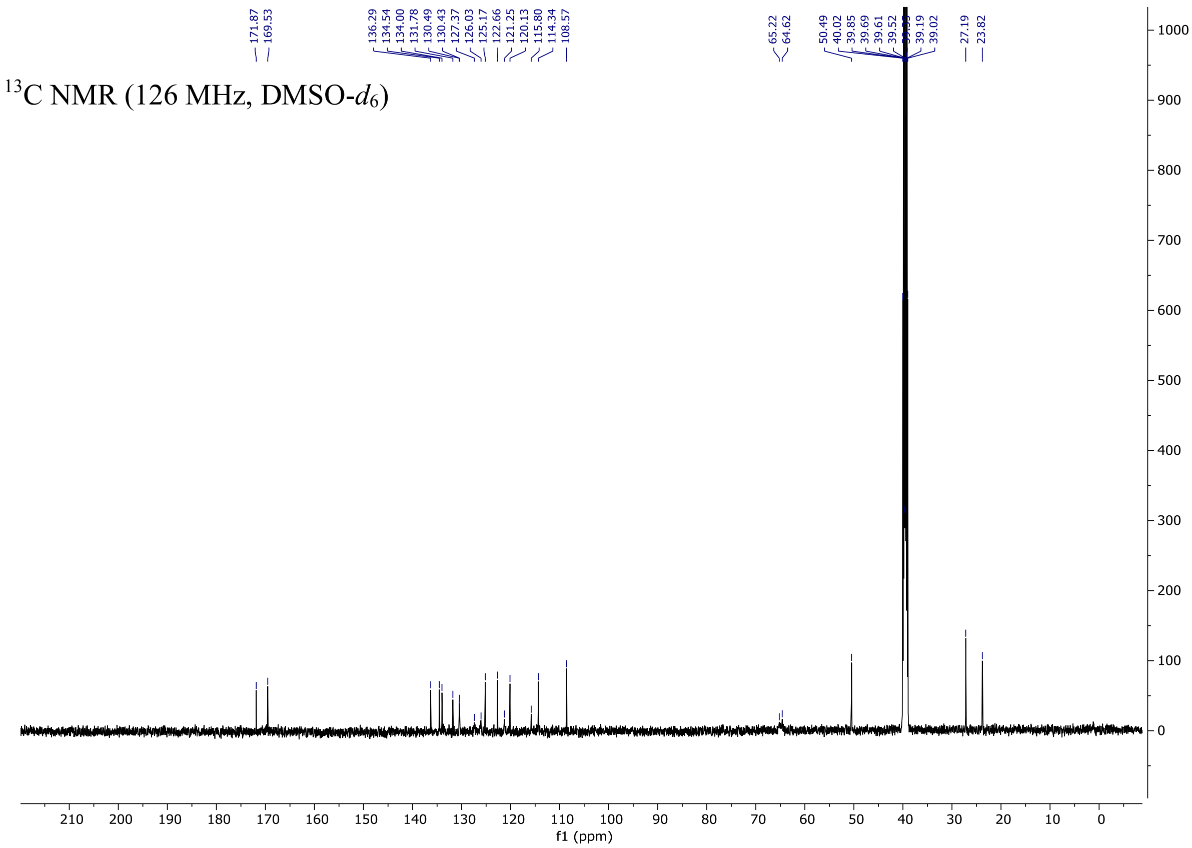
13C NMR (126 MHz, DMSO-d6)
171.87
169.53
136.29
134.54
134.00
131.78
130.49
130.43
127.37
126.03
125.17
122.66
121.25
120.13
115.80
114.34
108.57
65.22
64.62
50.49
40.02
39.85
39.69
39.61
39.52
39.19
39.02
27.19
23.82
f1 (ppm)

Compound 27:

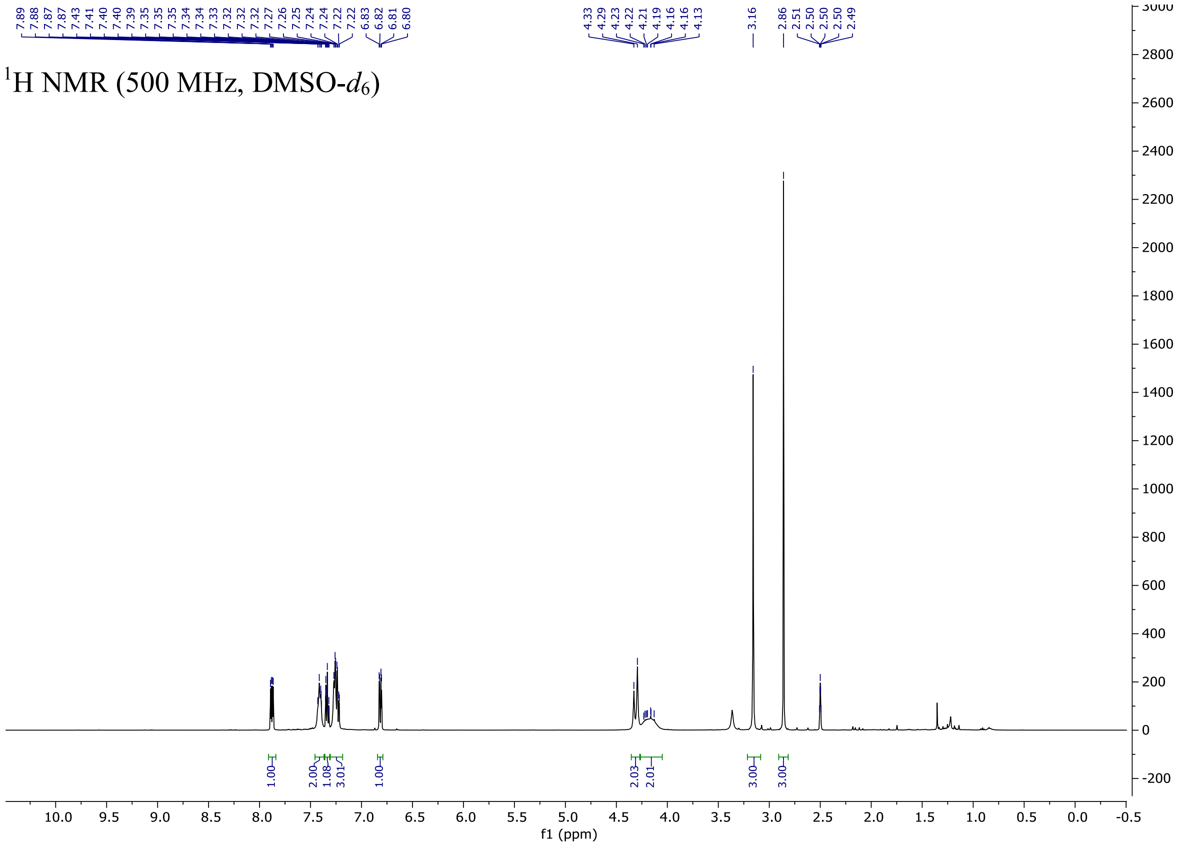

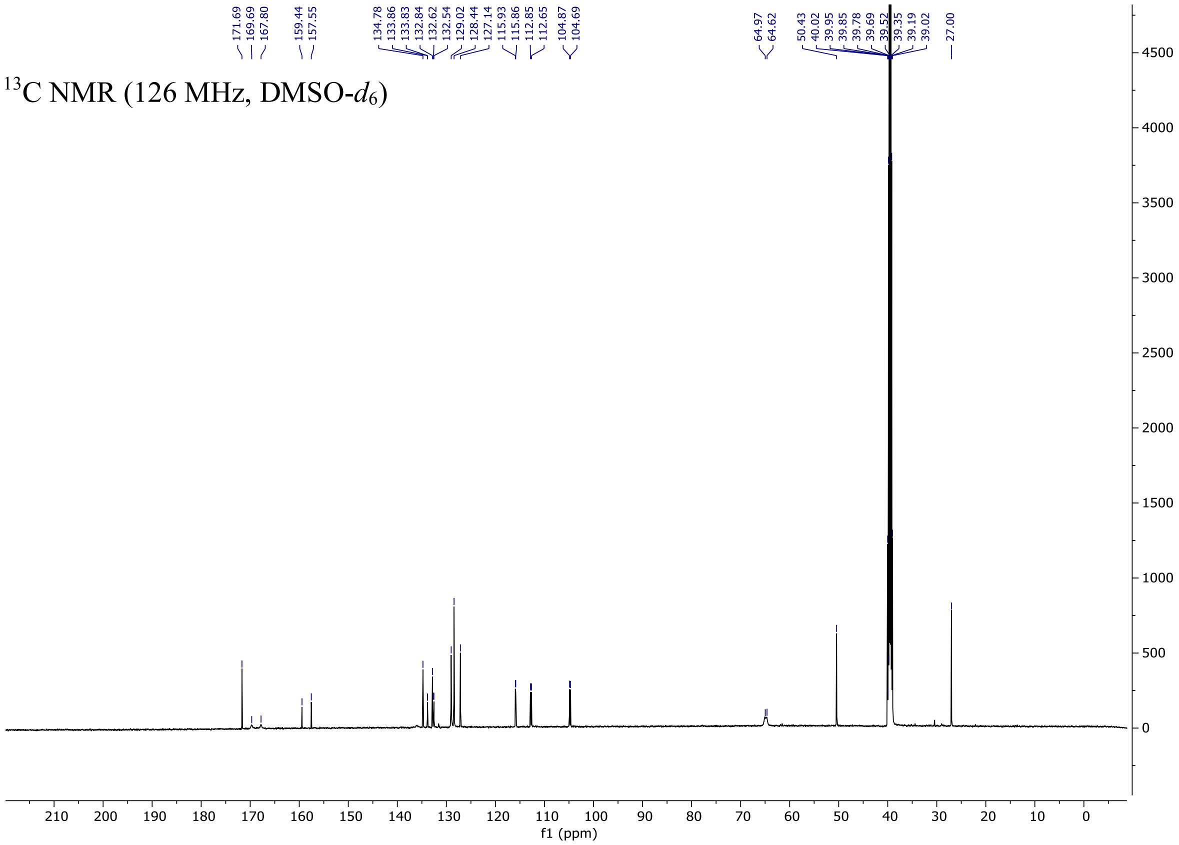

^{19}F NMR (376 MHz, DMSO-d_6)

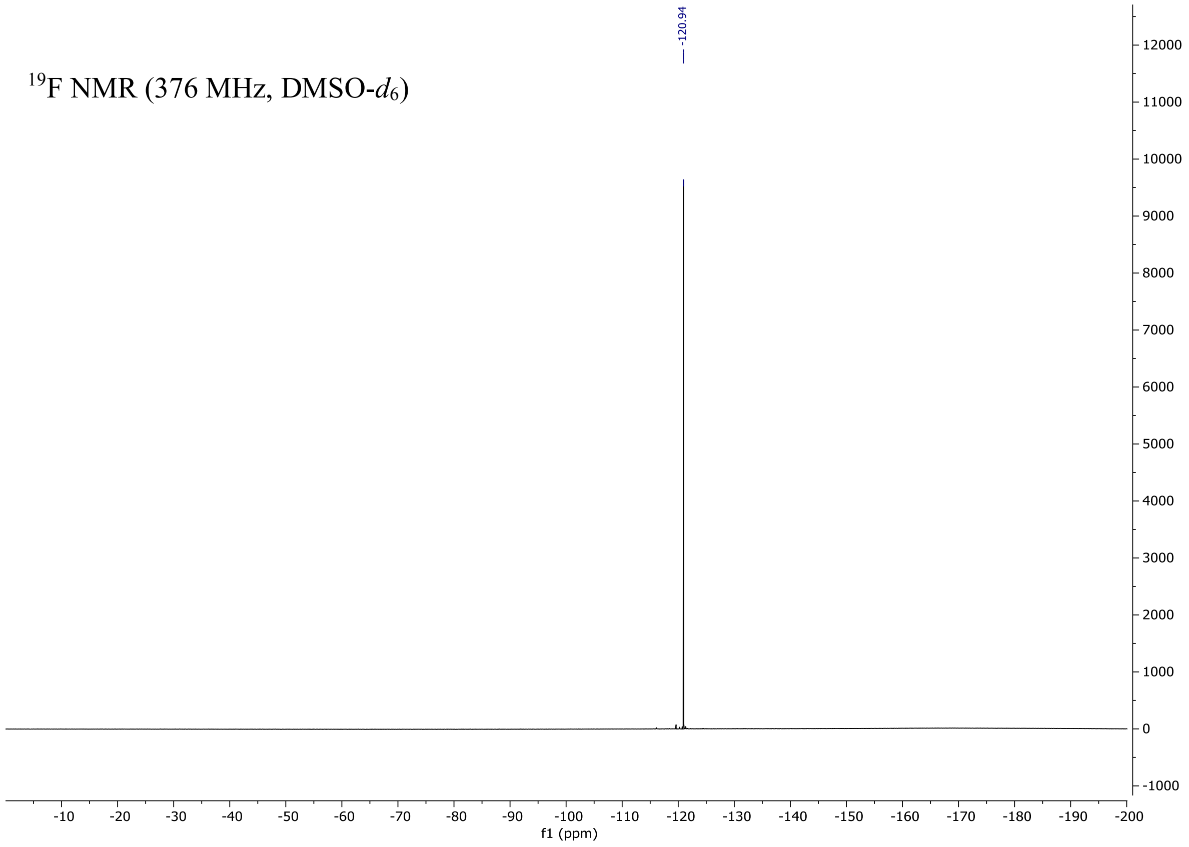

Compound 28:

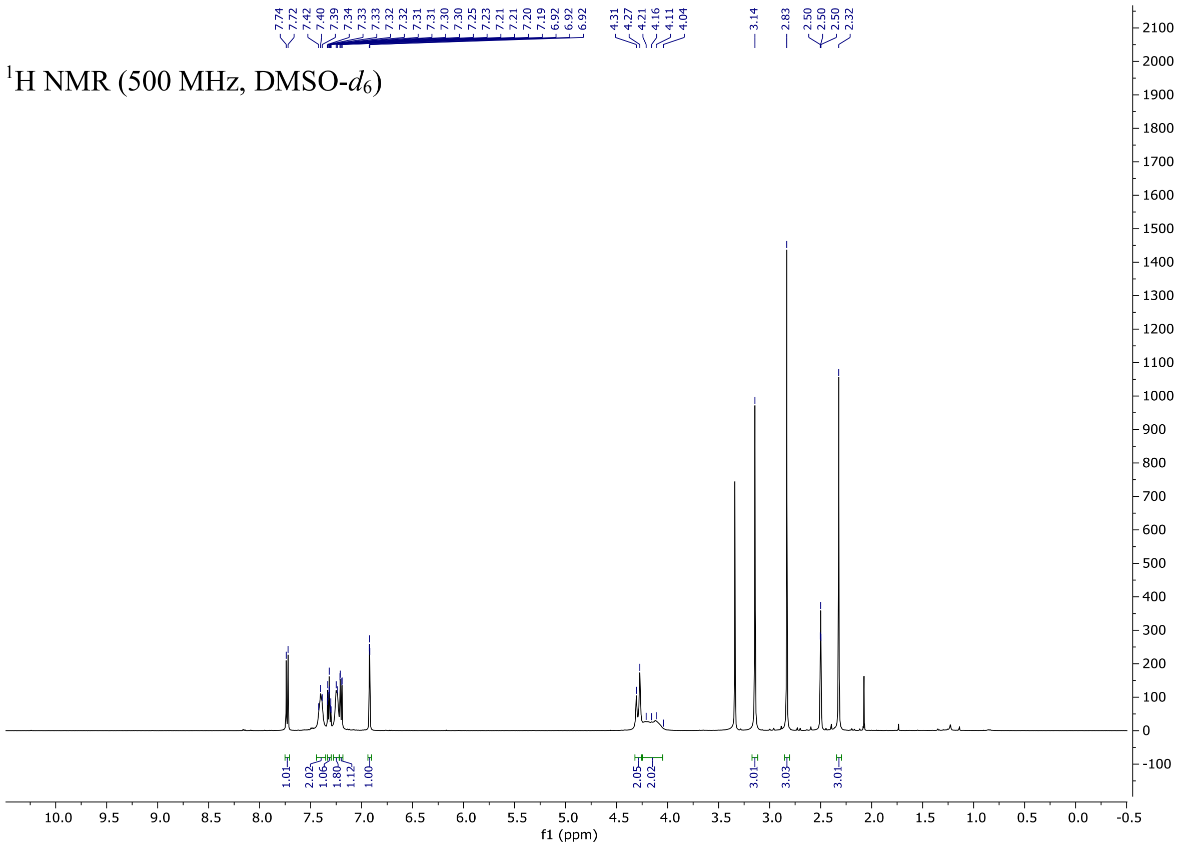

^{1}H NMR (500 MHz, DMSO-d_6)
7.74
7.72
7.42
7.40
7.39
7.34
7.33
7.33
7.32
7.32
7.31
7.31
7.30
7.30
7.25
7.23
7.21
7.21
7.20
7.19
6.92
6.92
6.92
4.31
4.27
4.21
4.16
4.11
4.04
3.14
2.83
2.50
2.50
2.50
2.32
1.01
2.02
1.06
1.80
1.12
1.00
2.05
2.02
3.01
3.03
3.01
f1 (ppm)

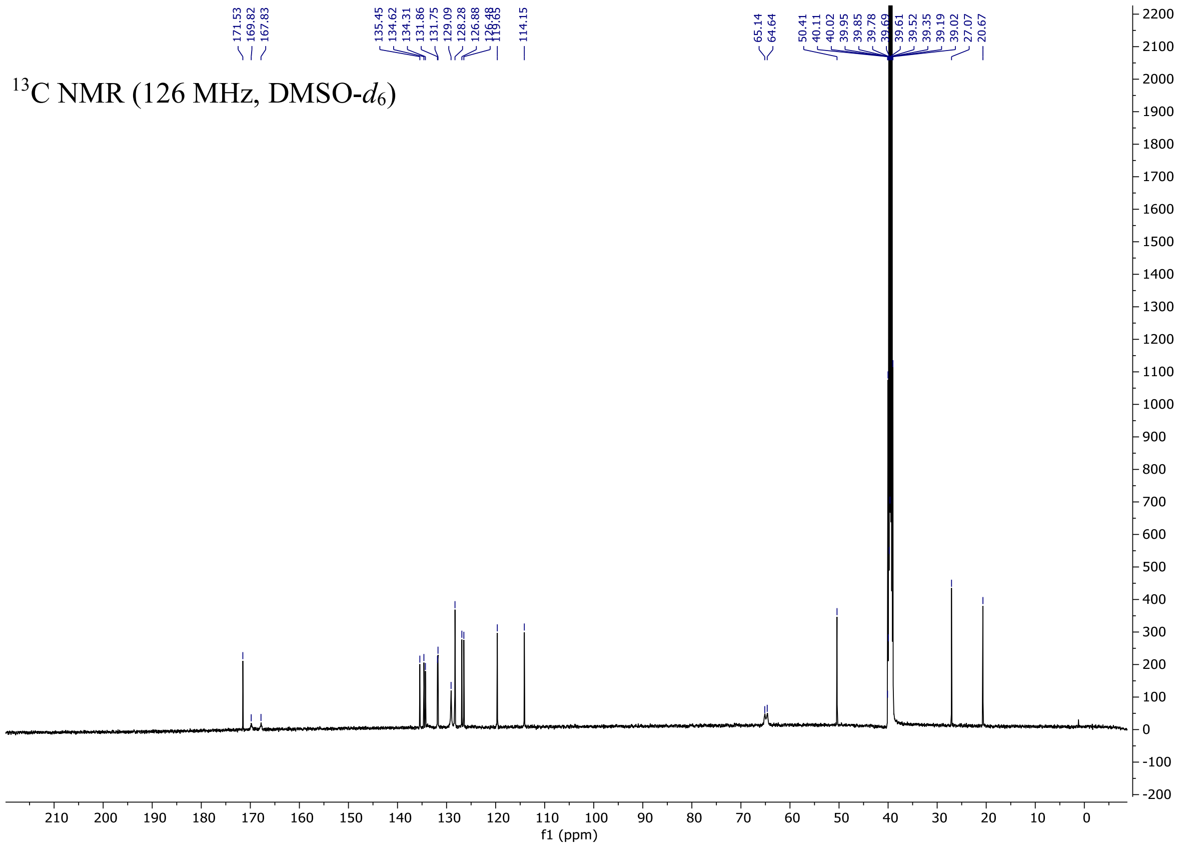

^{13}C NMR (126 MHz, DMSO-d_6)
171.53
169.82
167.83
135.45
134.62
134.31
131.86
131.75
129.09
128.28
126.88
126.48
119.65
114.15
65.14
64.64
50.41
40.11
40.02
39.95
39.85
39.78
39.69
39.61
39.52
39.35
39.19
39.02
27.07
20.67
f1 (ppm)

Compound 29:

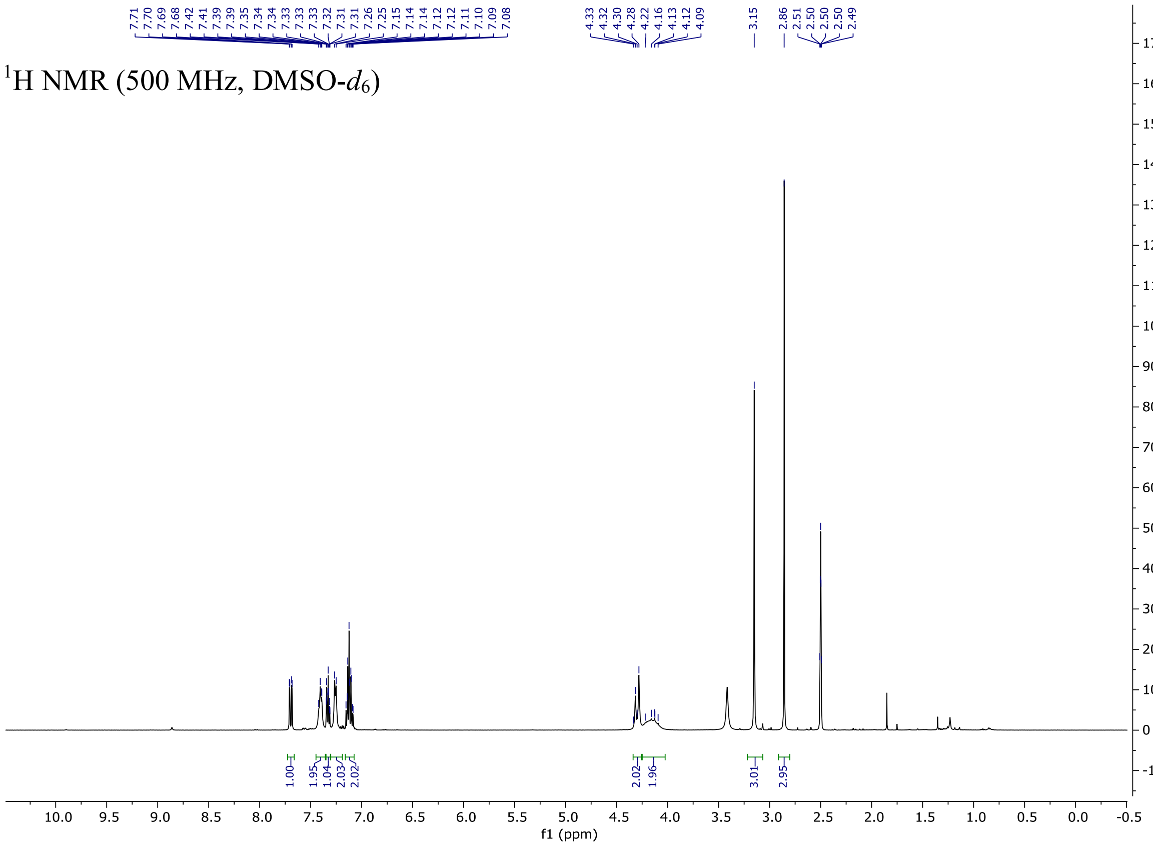

1H NMR (500 MHz, DMSO-d6)
7.71 7.70 7.69 7.68 7.42 7.41 7.39 7.39 7.35 7.34 7.34 7.33 7.33 7.33 7.32 7.31 7.31 7.26 7.25 7.15 7.14 7.14 7.12 7.12 7.11 7.10 7.09 7.08
4.33 4.32 4.30 4.28 4.22 4.16 4.13 4.12 4.09
3.15
2.86 2.51 2.50 2.50 2.50 2.49
1.00 1.95 1.04 2.03 2.02 2.02 1.96 3.01 2.95
f1 (ppm)

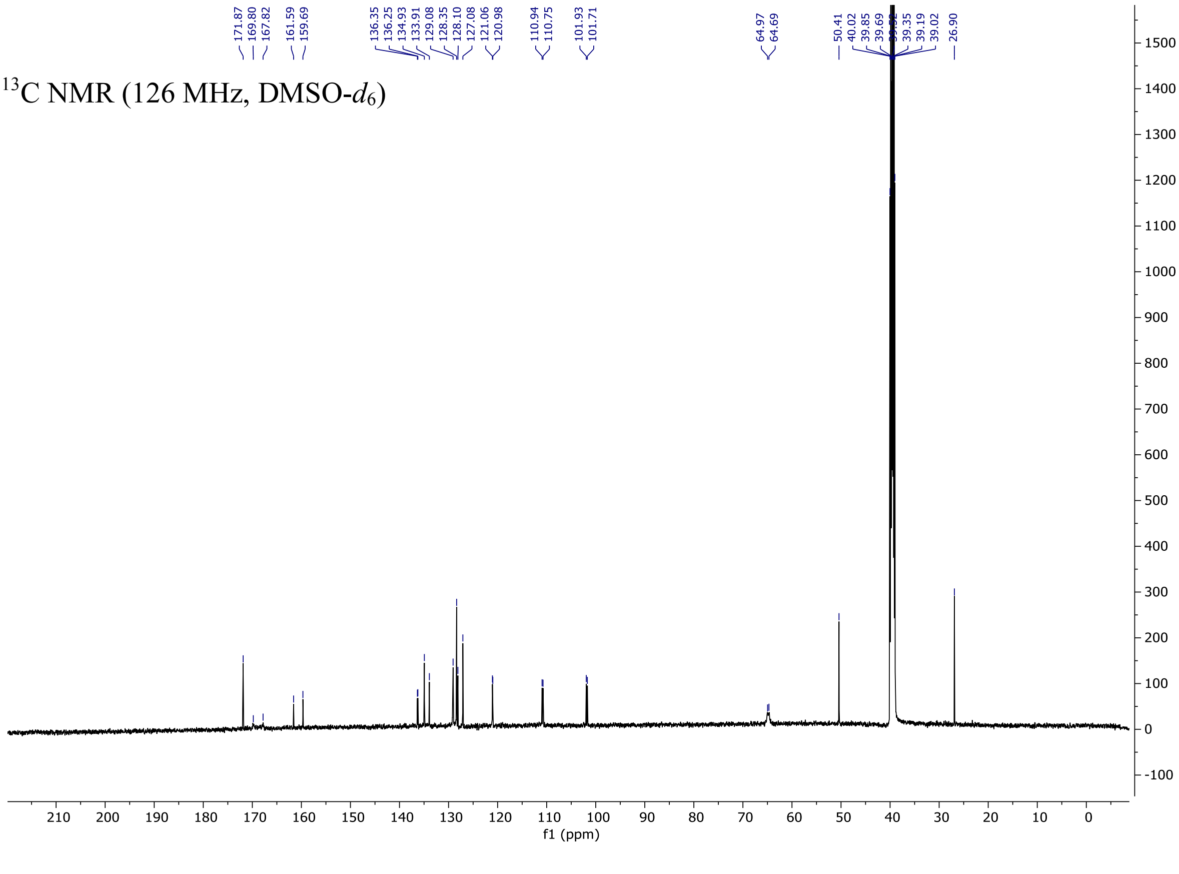

13C NMR (126 MHz, DMSO-d6)
171.87 169.80 167.82 161.59 159.69
136.35 136.25 134.93 133.91 129.08 128.35 128.10 127.08 121.06 120.98
110.94 110.75 101.93 101.71
64.97 64.69
50.41 40.02 39.85 39.69 39.35 39.19 39.02 26.90
f1 (ppm)

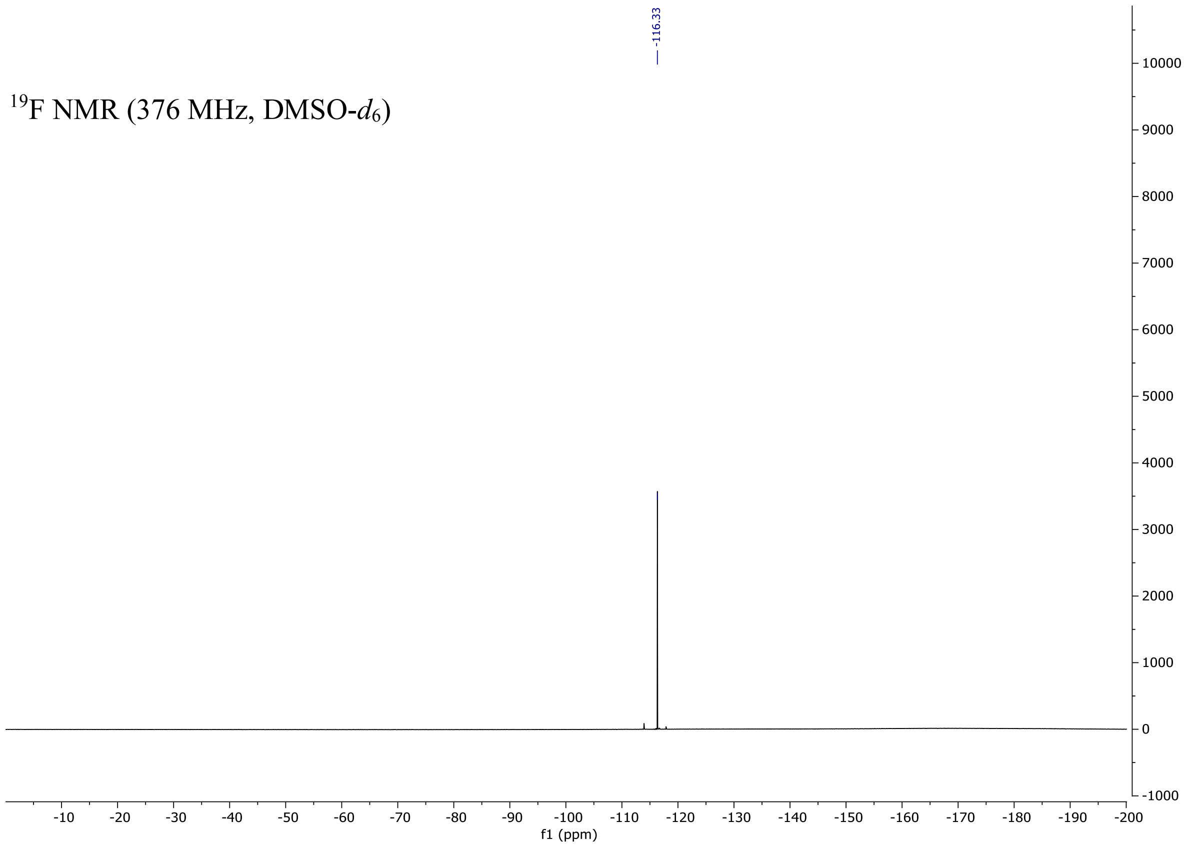

^{19}F NMR (376 MHz, DMSO-d_6)
-116.33
f1 (ppm)

Compound 30:

^{1}H NMR (500 MHz, DMSO-d_6)

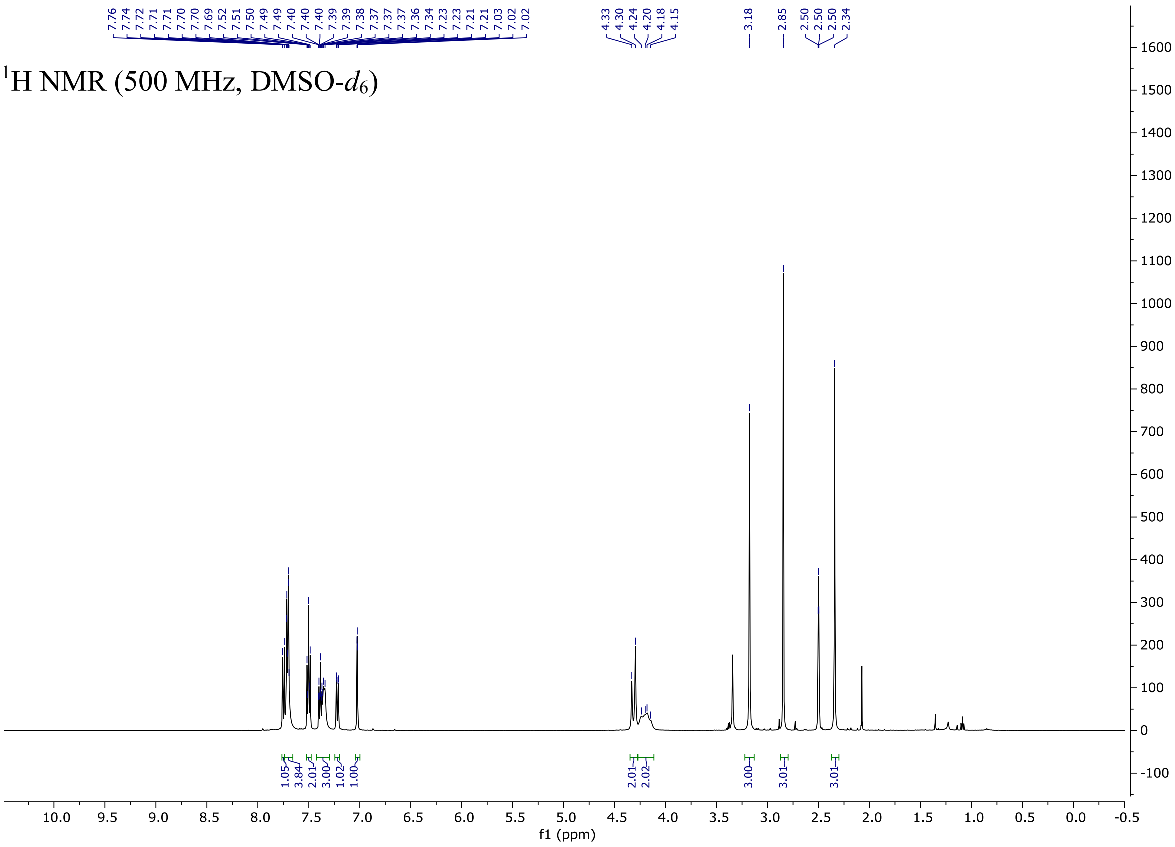

^{13}C NMR (126 MHz, DMSO-d_6)

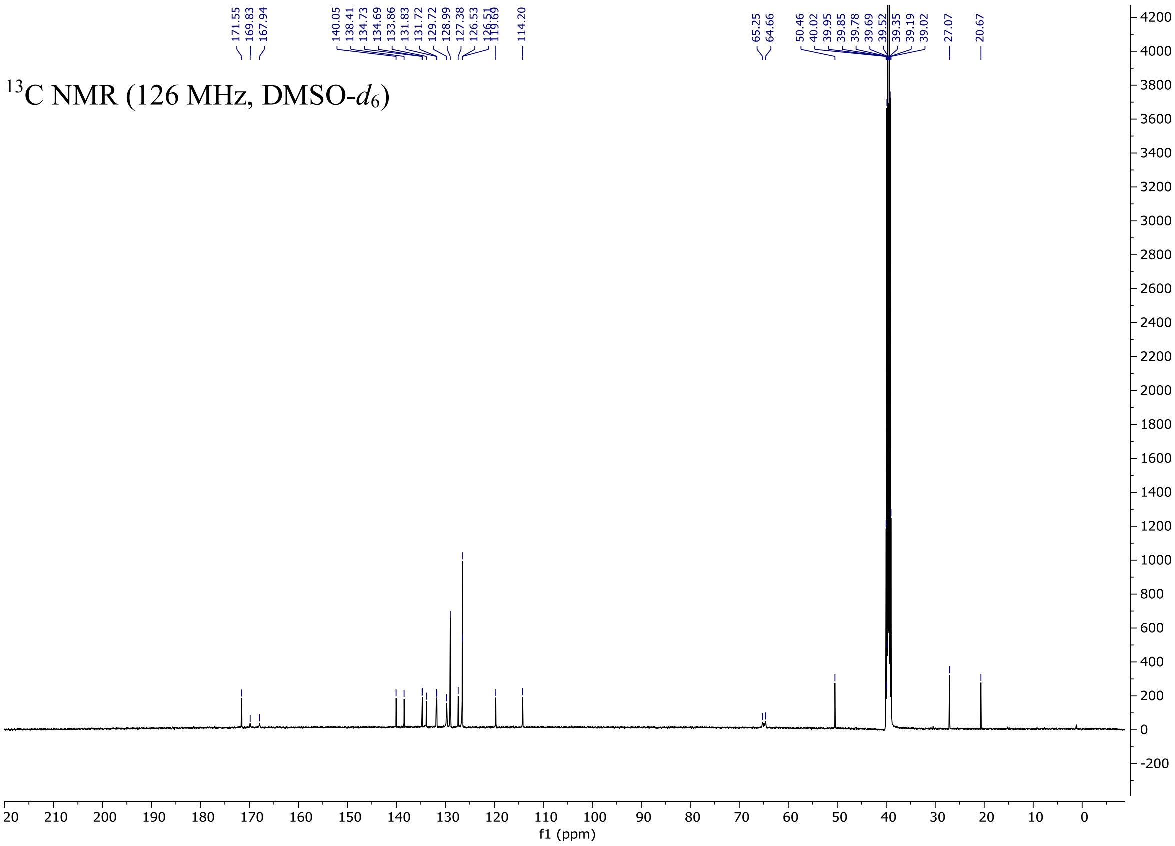

Compound 31:

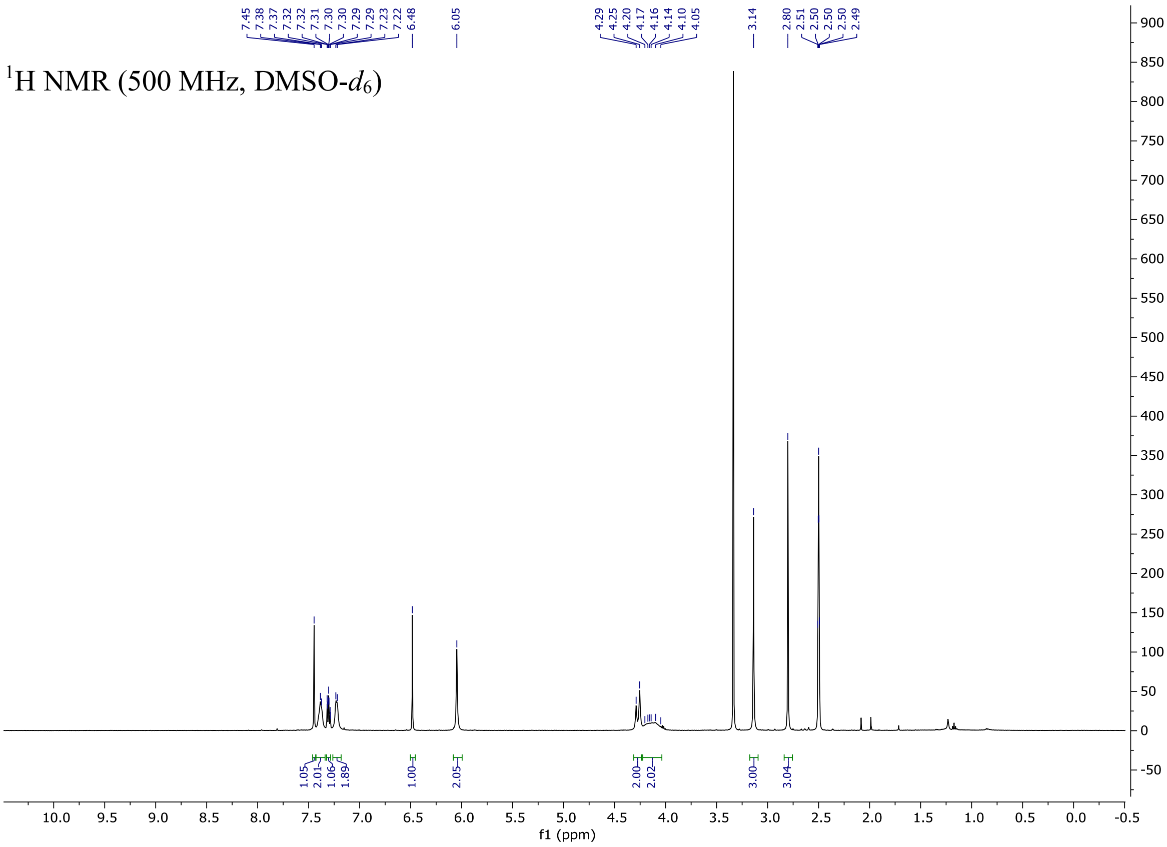

7.45
7.38
7.37
7.32
7.32
7.31
7.30
7.30
7.29
7.29
7.29
7.23
7.22
6.48
6.05
4.29
4.25
4.20
4.17
4.16
4.14
4.10
4.05
3.14
2.80
2.51
2.50
2.50
2.50
2.49
^{1}H NMR (500 MHz, DMSO-d_6)
1.05
2.01
1.06
1.89
1.00
2.05
2.00
2.02
3.00
3.04
f1 (ppm)

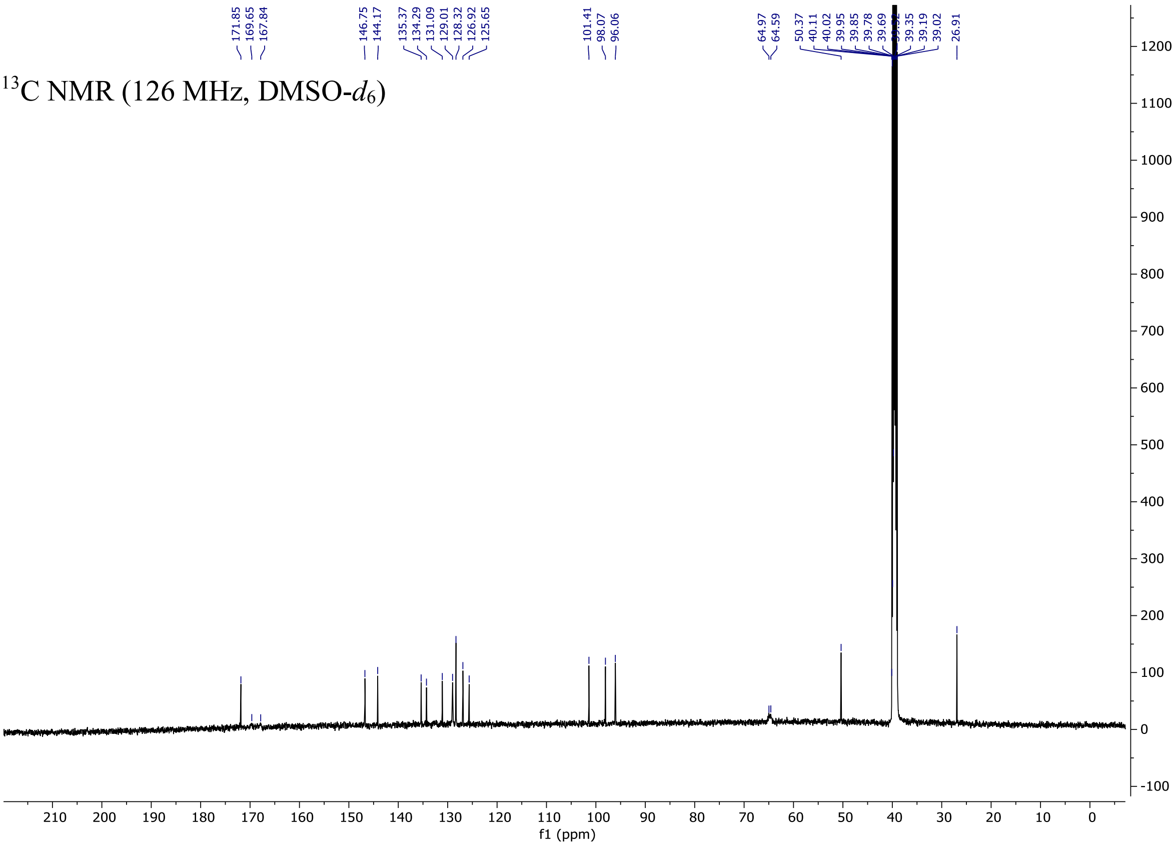

171.85
169.65
167.84
146.75
144.17
135.37
134.29
131.09
129.01
128.32
126.92
125.65
101.41
98.07
96.06
64.97
64.59
50.37
40.11
40.02
39.95
39.85
39.78
39.69
39.35
39.19
39.02
26.91
^{13}C NMR (126 MHz, DMSO-d_6)
f1 (ppm)

Compound 32:

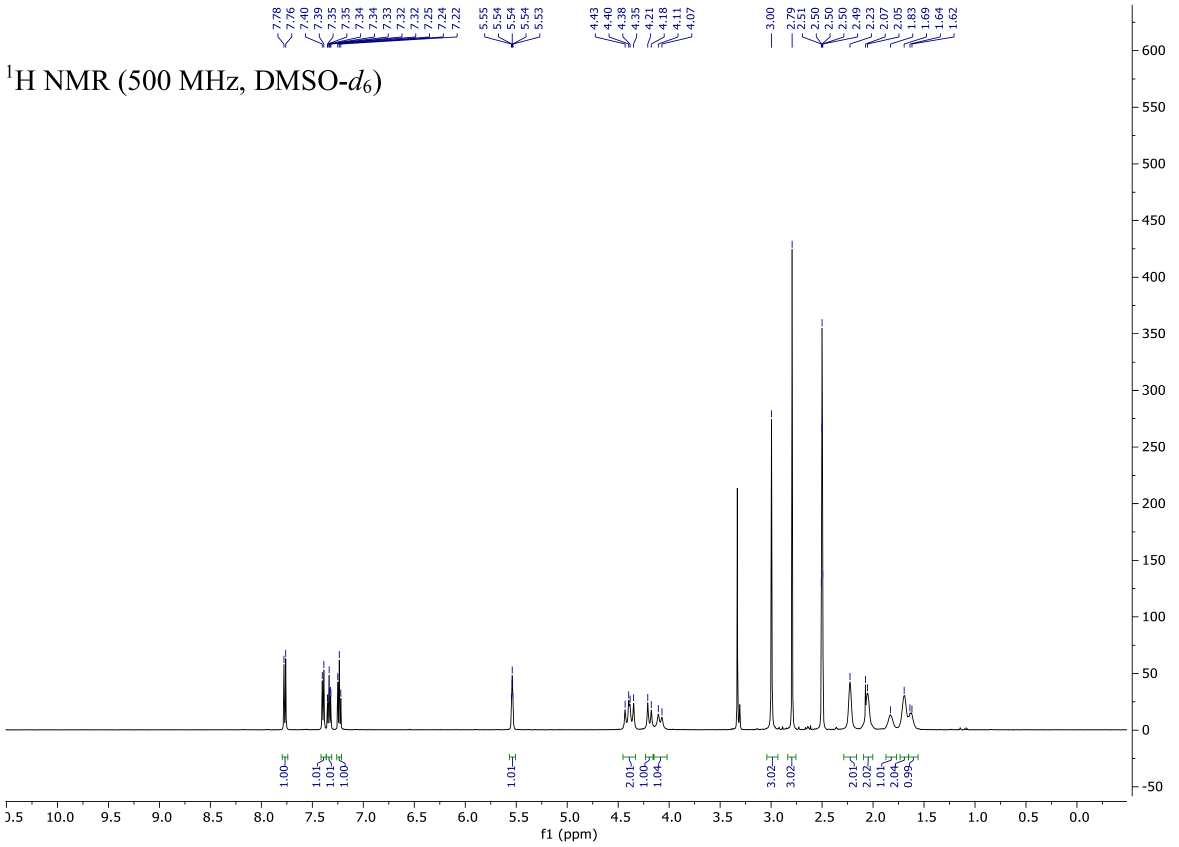

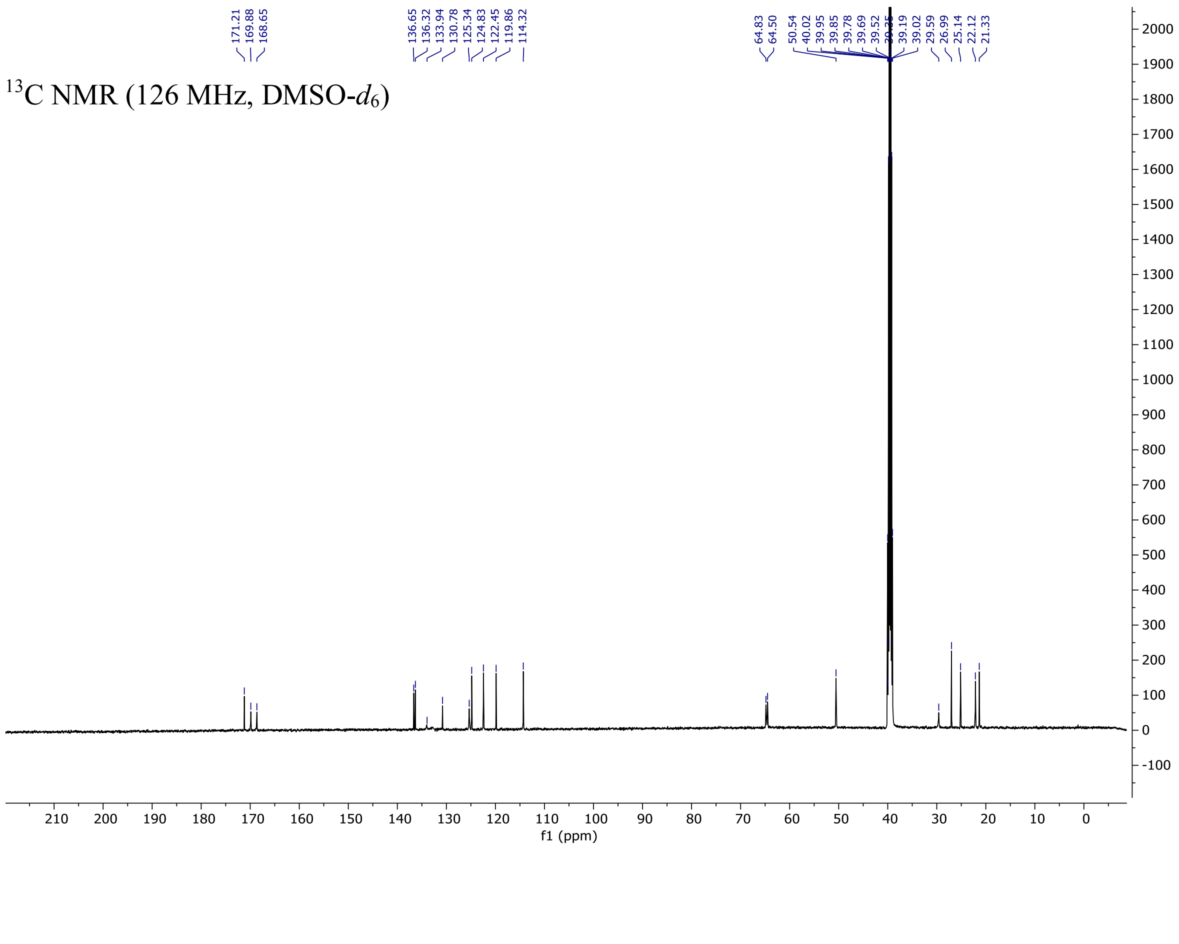

Compound 33:

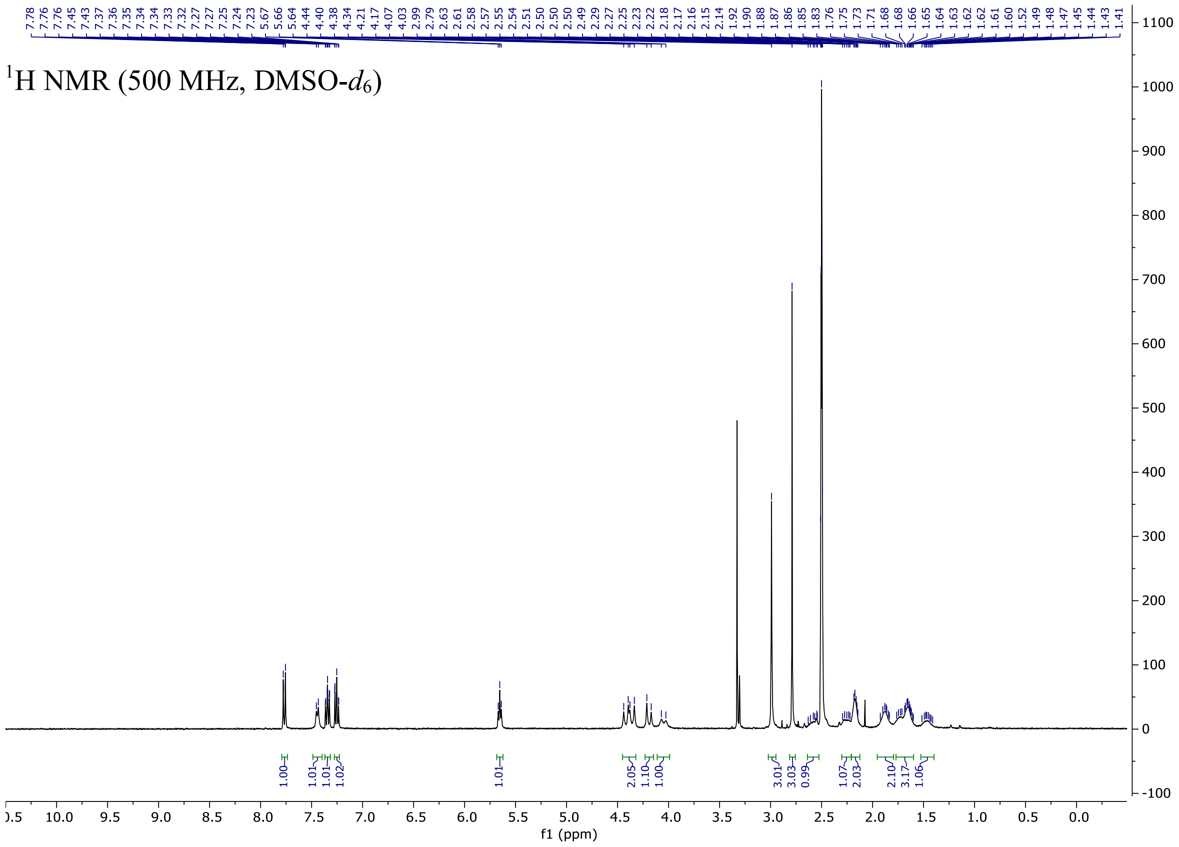

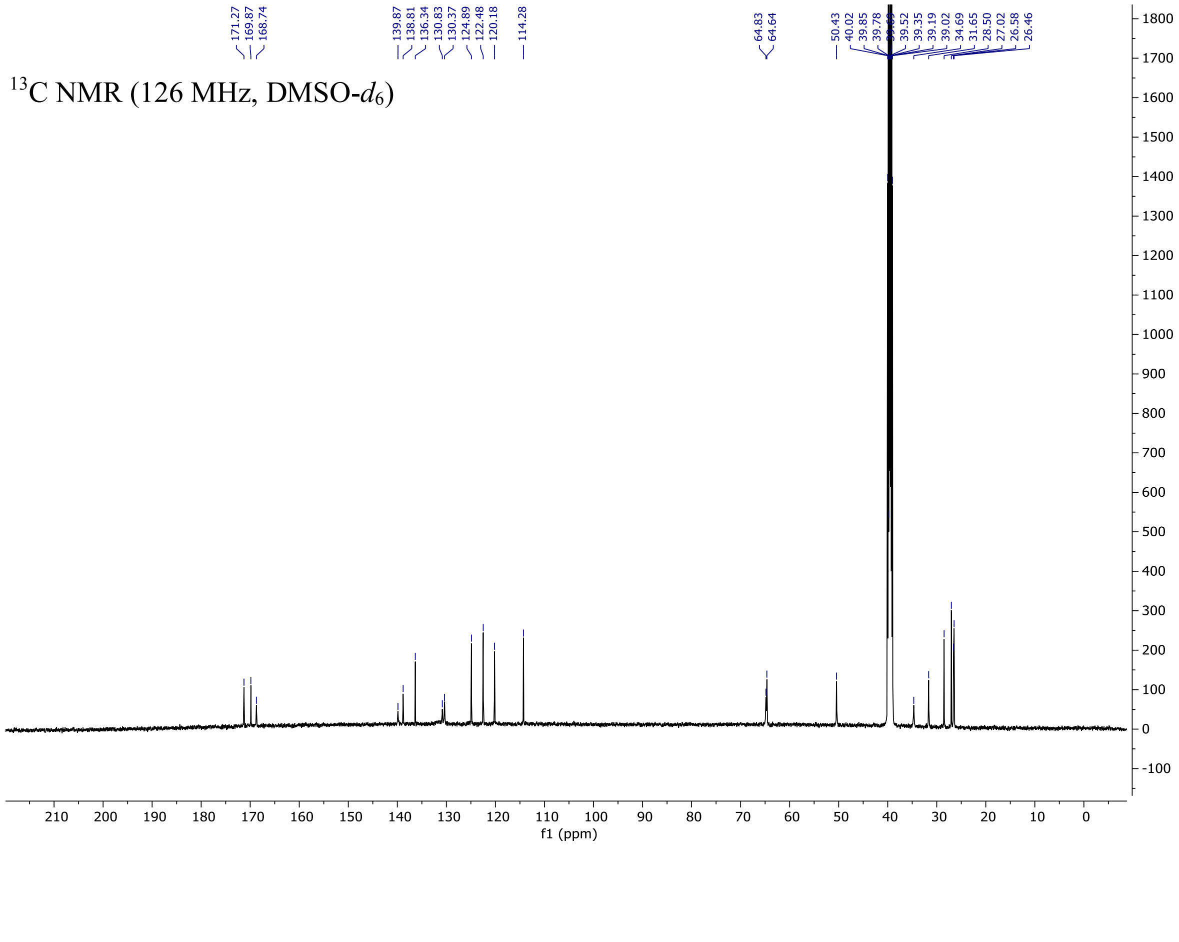

Compound 34:

^{1}H NMR (500 MHz, DMSO-d_6)

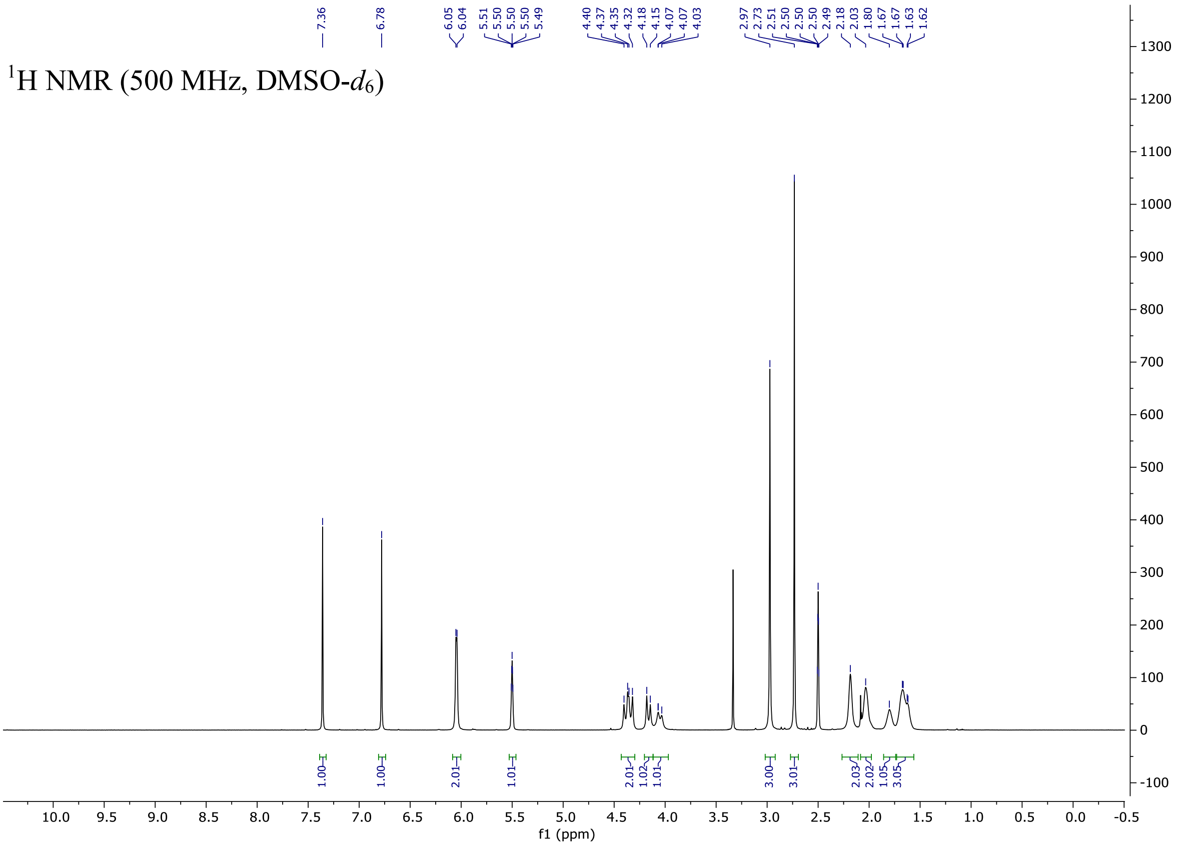

^{13}C NMR (126 MHz, DMSO-d_6)

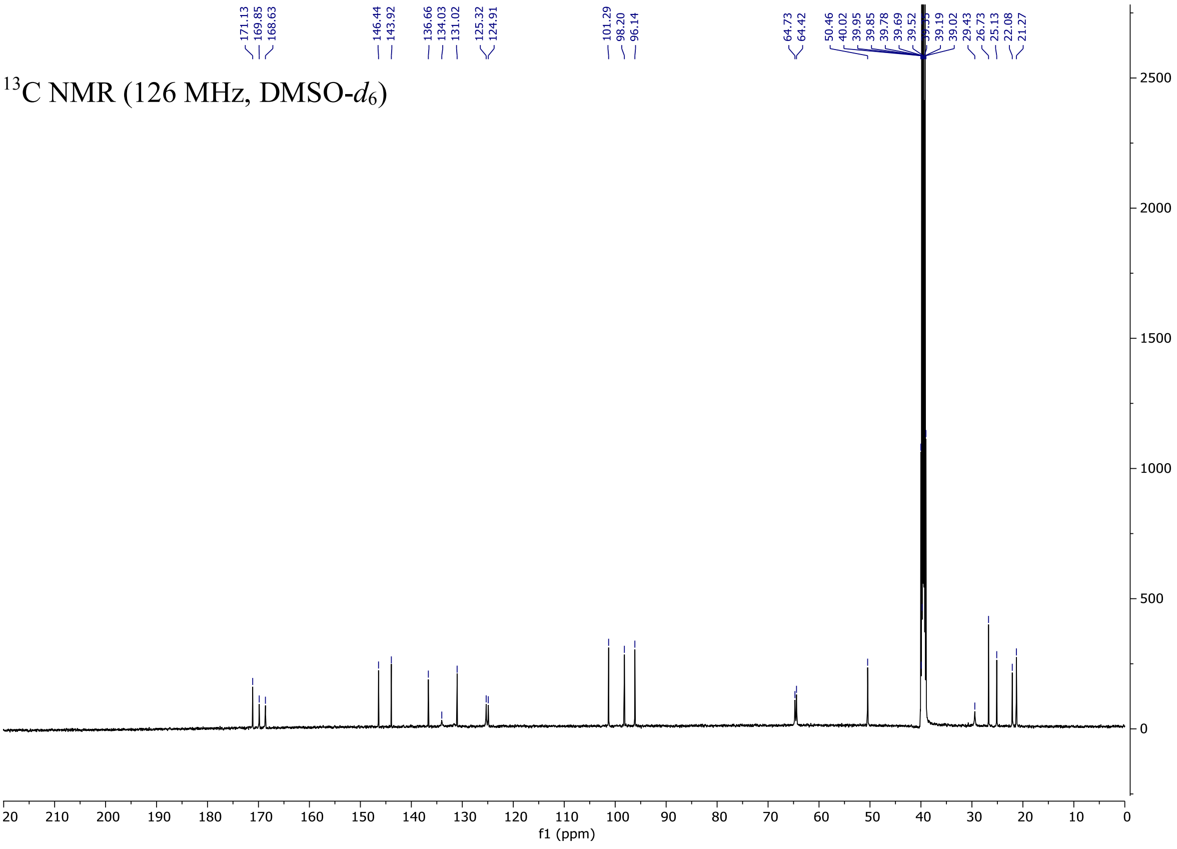

Compound 35:

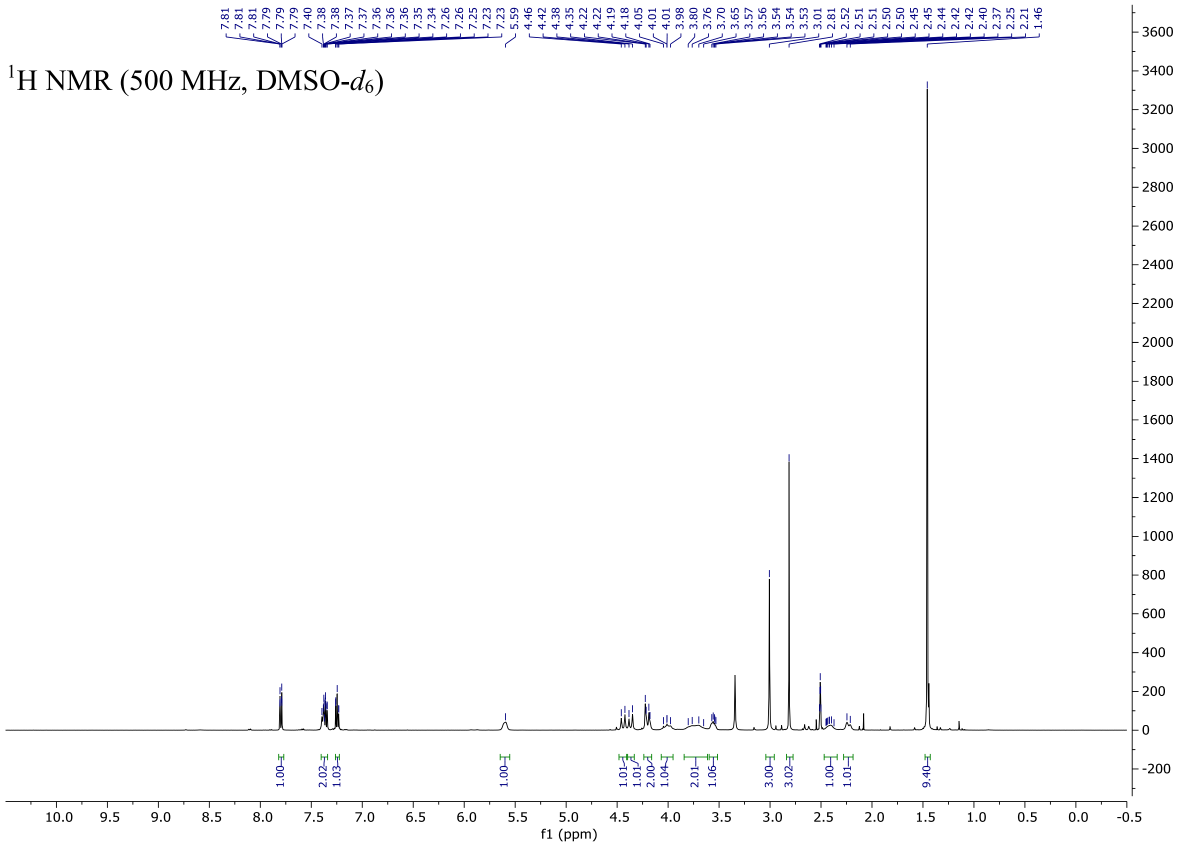
^{1}H NMR (500 MHz, DMSO-d_6)
7.81 7.81 7.81 7.79 7.79 7.79 7.40 7.38 7.38 7.37 7.37 7.36 7.36 7.36 7.35 7.34 7.26 7.26 7.25 7.23 7.23 7.23 5.59 4.46 4.42 4.38 4.35 4.22 4.22 4.19 4.18 4.05 4.01 4.01 3.98 3.80 3.76 3.70 3.65 3.57 3.56 3.54 3.54 3.53 3.01 2.81 2.52 2.51 2.51 2.50 2.50 2.45 2.45 2.44 2.42 2.42 2.40 2.37 2.25 2.21 1.46
1.00 2.02 1.03 1.00 1.01 1.01 2.00 1.04 2.01 1.06 3.00 3.02 1.00 1.01 9.40
f1 (ppm)

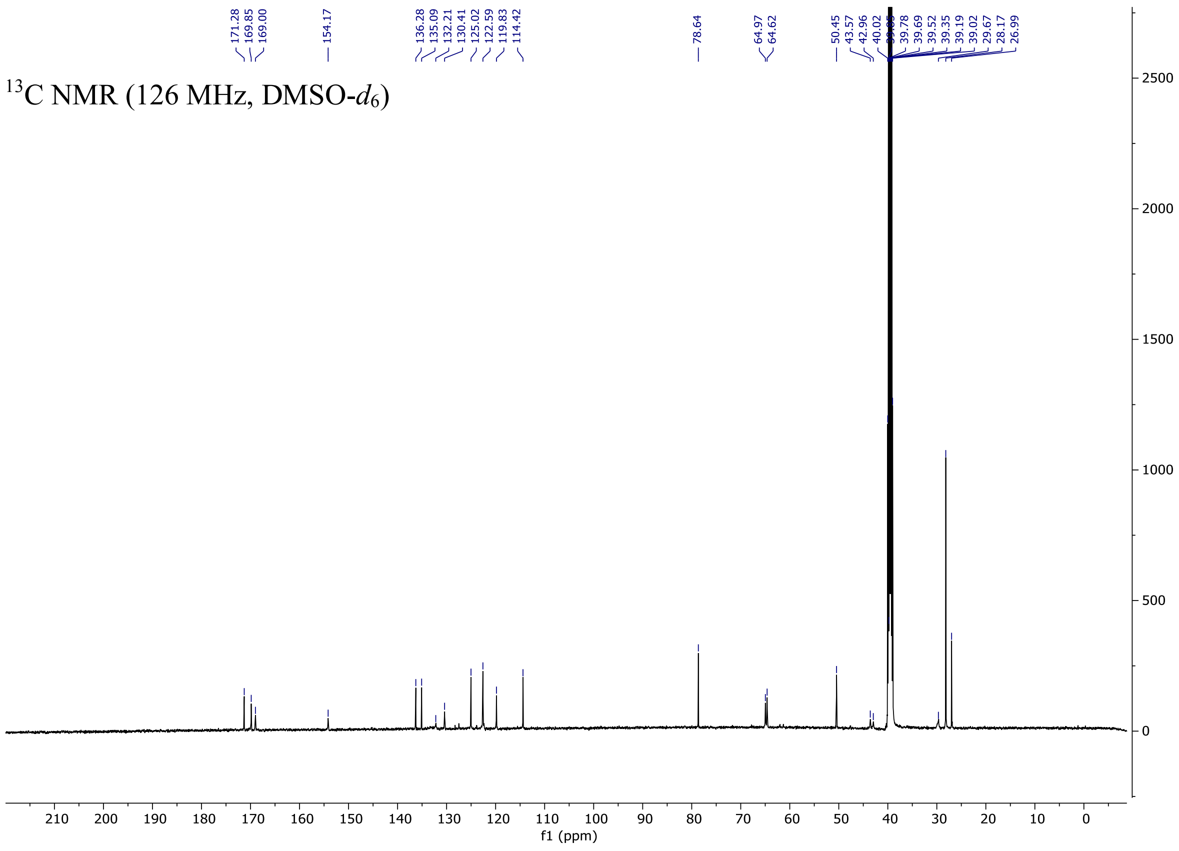
^{13}C NMR (126 MHz, DMSO-d_6)
171.28 169.85 169.00 154.17 136.28 135.09 132.21 130.41 125.02 122.59 119.83 114.42 78.64 64.97 64.62 50.45 43.57 42.96 40.02 39.78 39.69 39.52 39.35 39.19 39.02 29.67 28.17 26.99
f1 (ppm)

Compound 37:

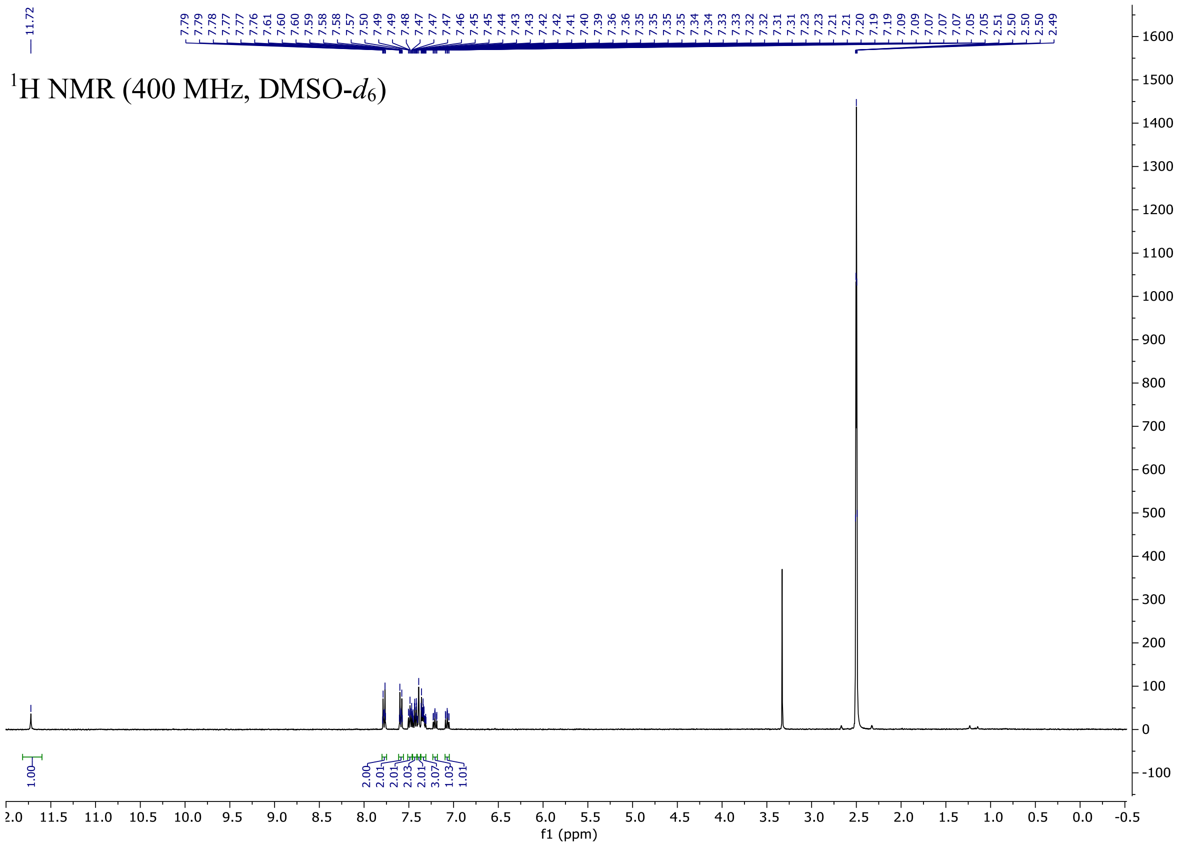

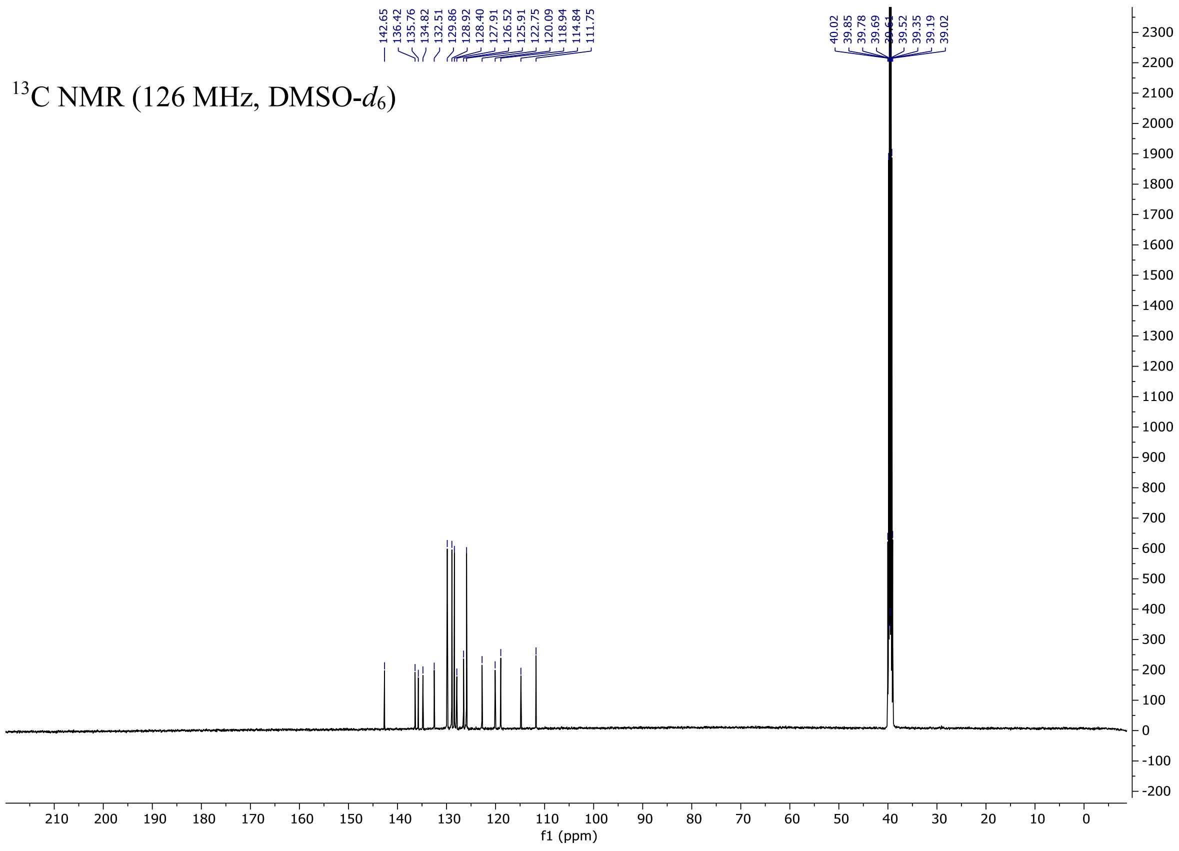

Compound 38:

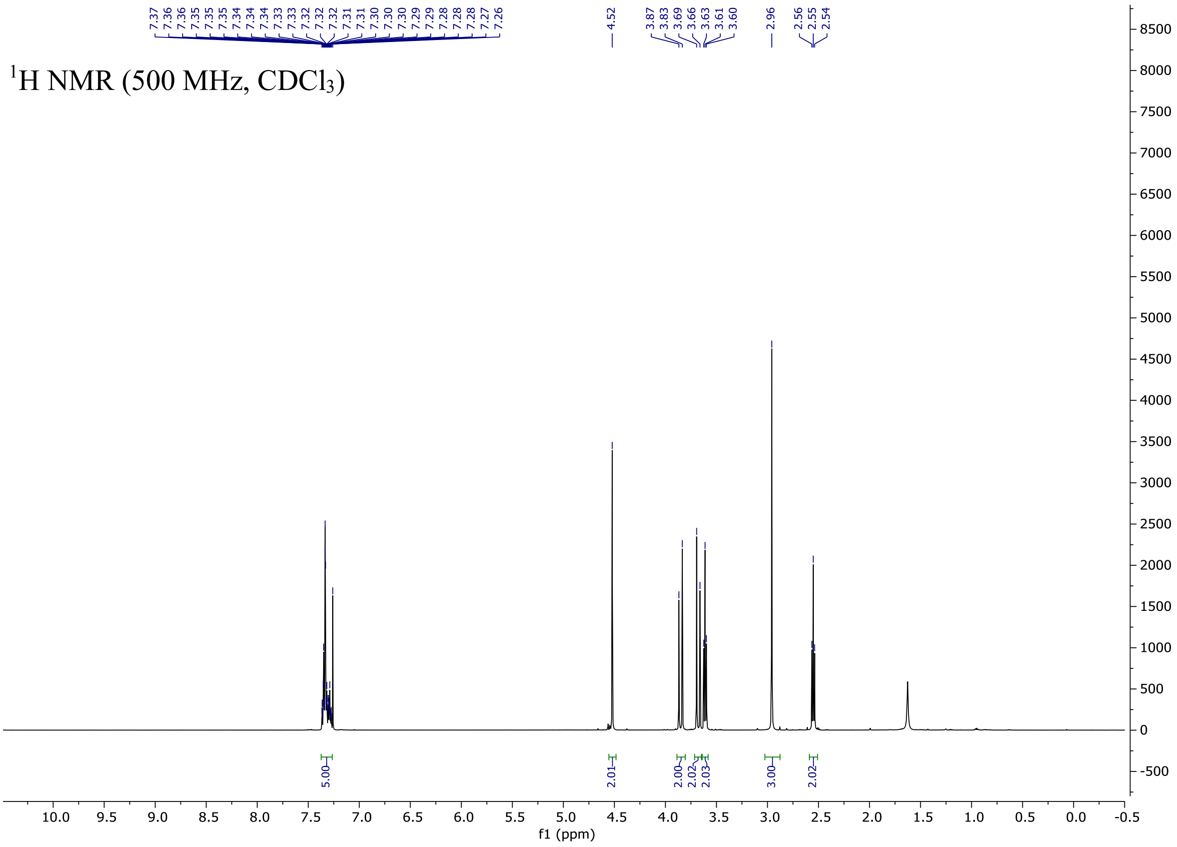

^{1}H NMR (500 MHz, CDCl$_3$)
7.37 7.36 7.36 7.35 7.35 7.35 7.34 7.34 7.34 7.33 7.33 7.32 7.32 7.32 7.31 7.31 7.30 7.30 7.29 7.29 7.28 7.28 7.28 7.27 7.26
4.52
3.87 3.83 3.69 3.66 3.63 3.61 3.60
2.96
2.56 2.55 2.54
5.00 2.01 2.00 2.02 2.03 3.00 2.02
f1 (ppm)

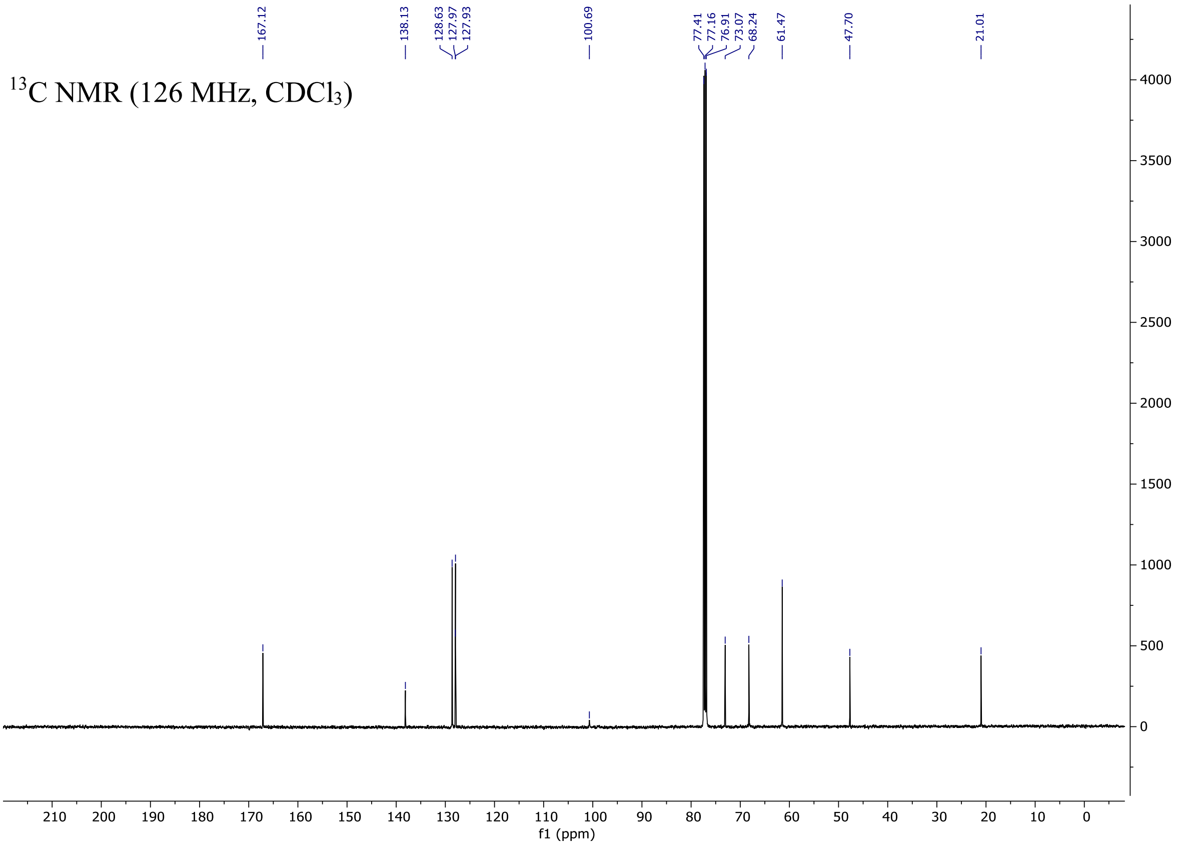

^{13}C NMR (126 MHz, CDCl$_3$)
167.12
138.13
128.63 127.97 127.93
100.69
77.41 77.16 76.91 73.07 68.24
61.47
47.70
21.01
f1 (ppm)

Compound 39:

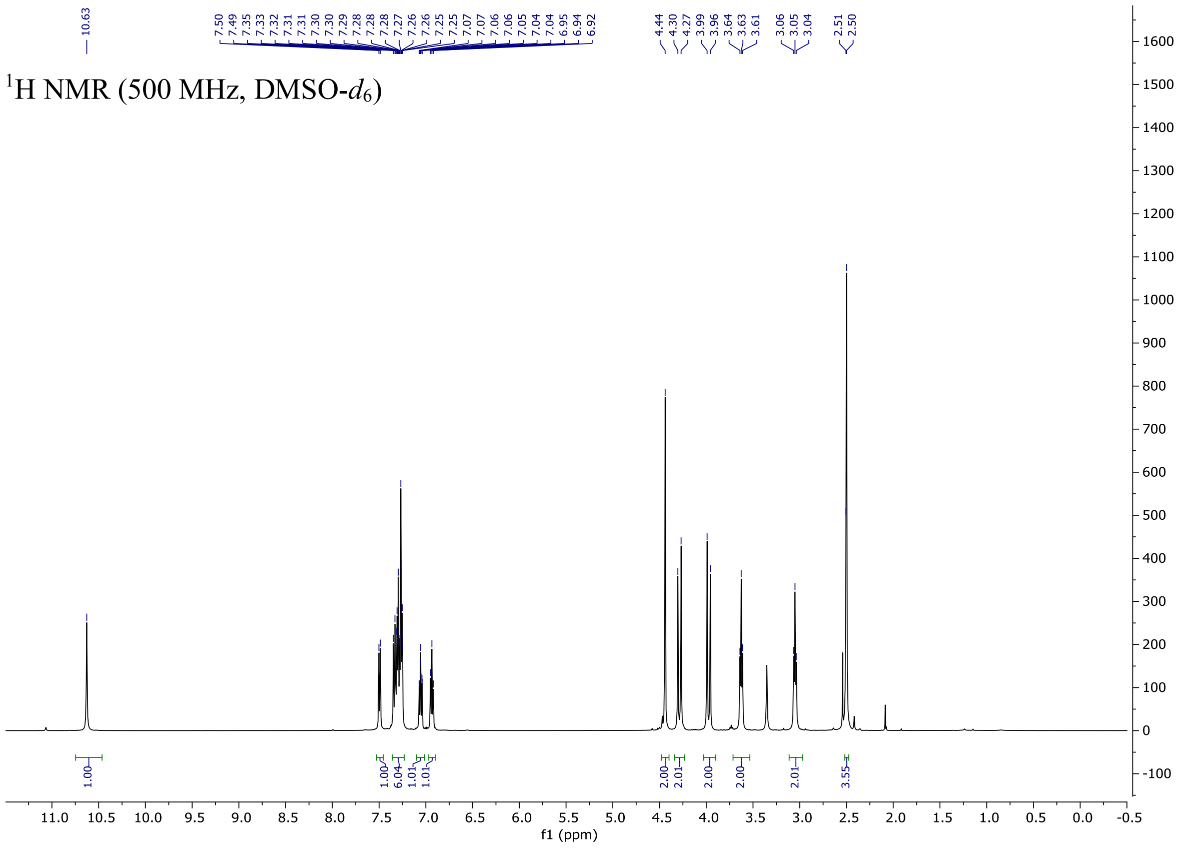

10.63
7.50 7.49 7.35 7.33 7.32 7.31 7.31 7.30 7.30 7.29 7.28 7.28 7.27 7.26 7.26 7.25 7.25 7.07 7.07 7.06 7.06 7.05 7.04 7.04 6.95 6.94 6.92
4.44 4.30 4.27 3.99 3.96 3.64 3.63 3.61 3.06 3.05 3.04 2.51 2.50
¹H NMR (500 MHz, DMSO-d6)
1.00 1.00 6.04 1.01 1.01 2.00 2.01 2.00 2.00 2.01 3.55
f1 (ppm)

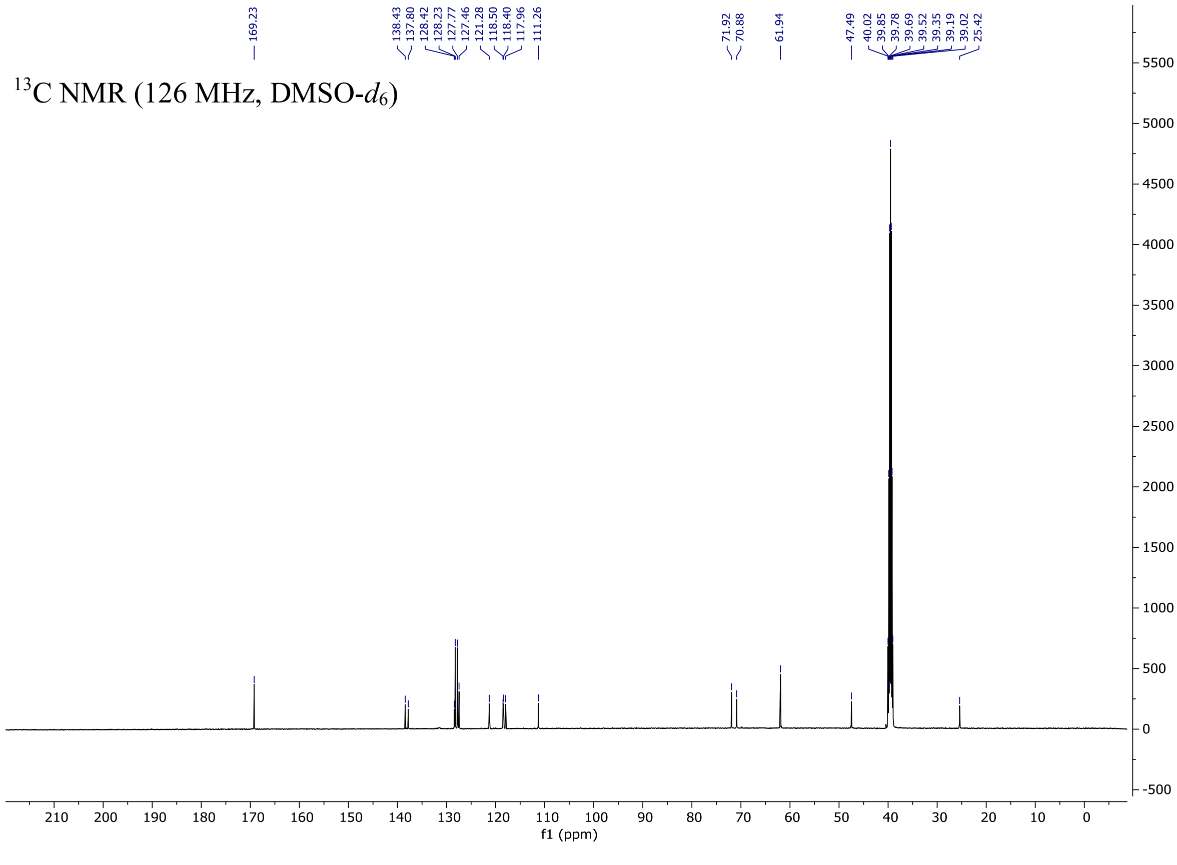

169.23
138.43 137.80 128.42 128.23 127.77 127.46 121.28 118.50 118.40 117.96 111.26
71.92 70.88 61.94 47.49 40.02 39.85 39.78 39.69 39.52 39.35 39.19 39.02 25.42
¹³C NMR (126 MHz, DMSO-d6)
f1 (ppm)

Compound 40:

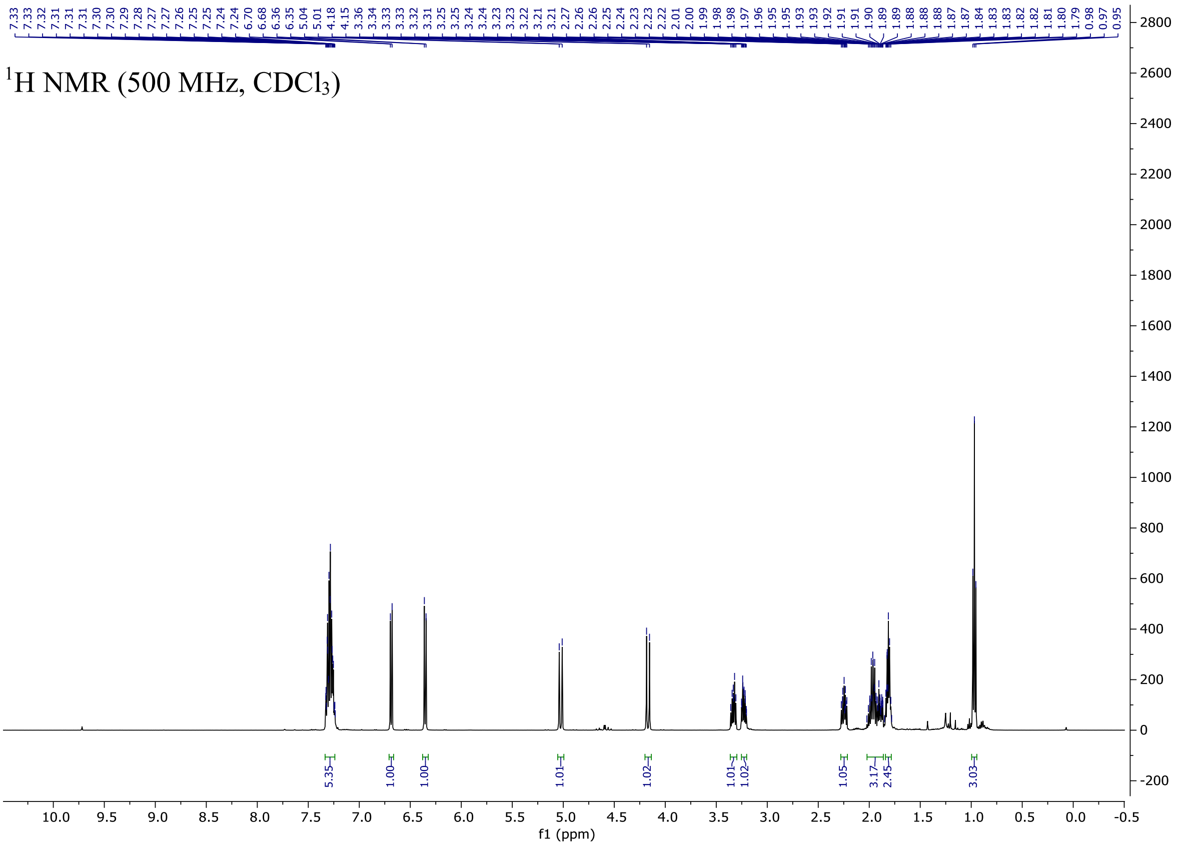

¹H NMR (500 MHz, CDCl3)
f1 (ppm)

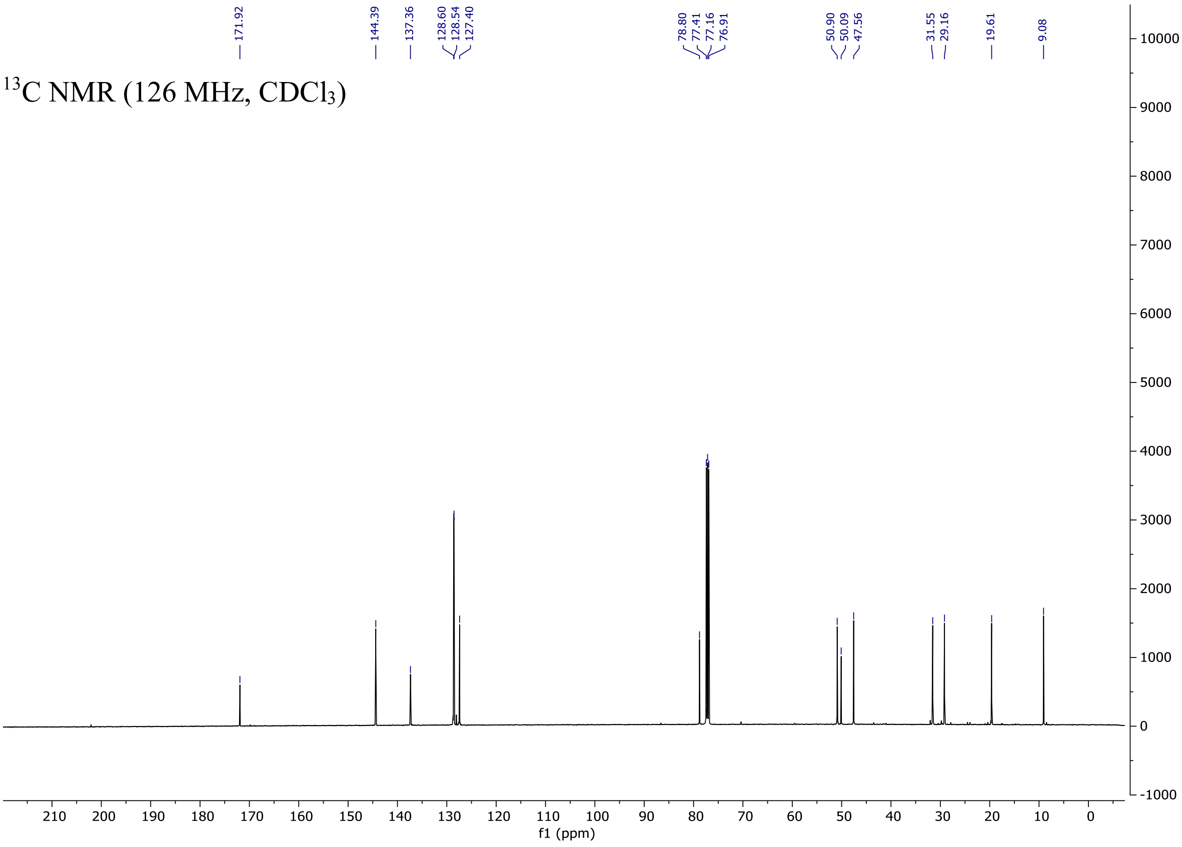

171.92
144.39
137.36
128.60
128.54
127.40
78.80
77.41
77.16
76.91
50.90
50.09
47.56
31.55
29.16
19.61
9.08
¹³C NMR (126 MHz, CDCl3)
f1 (ppm)

Compound 41:

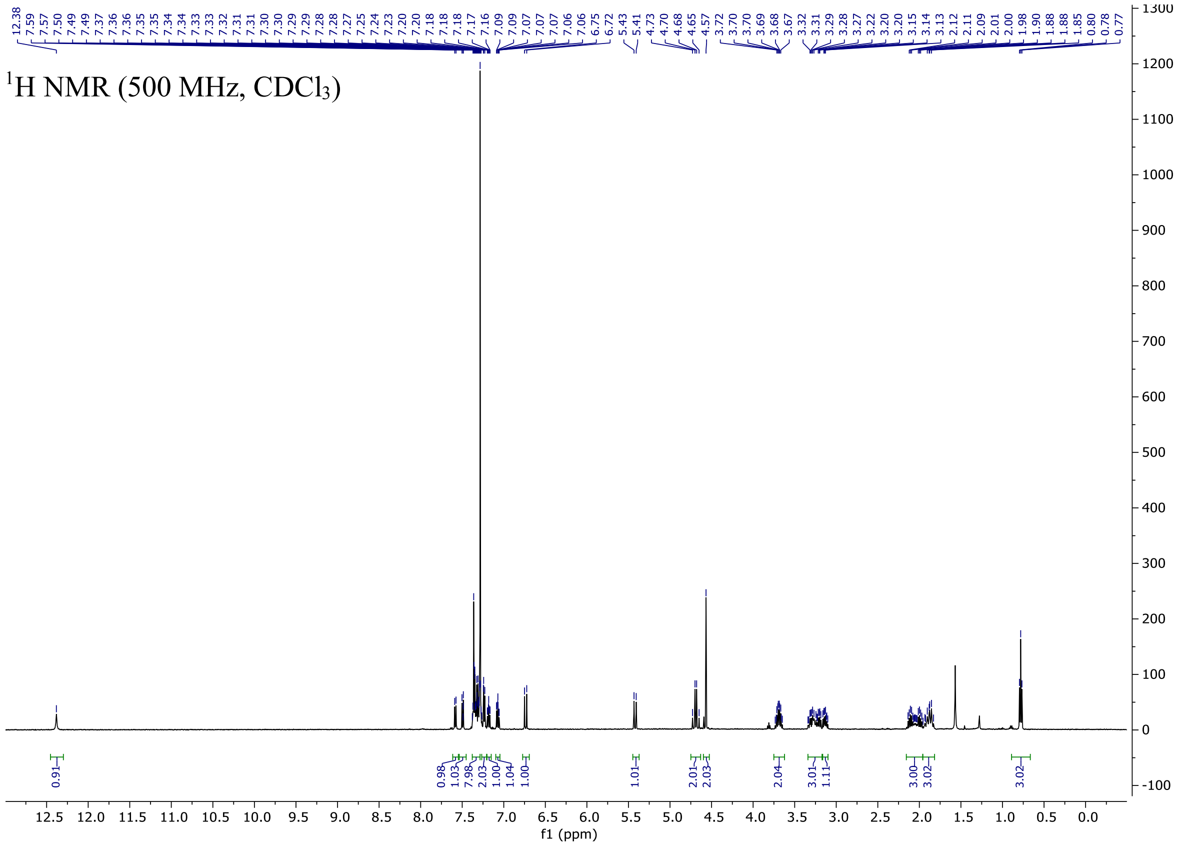

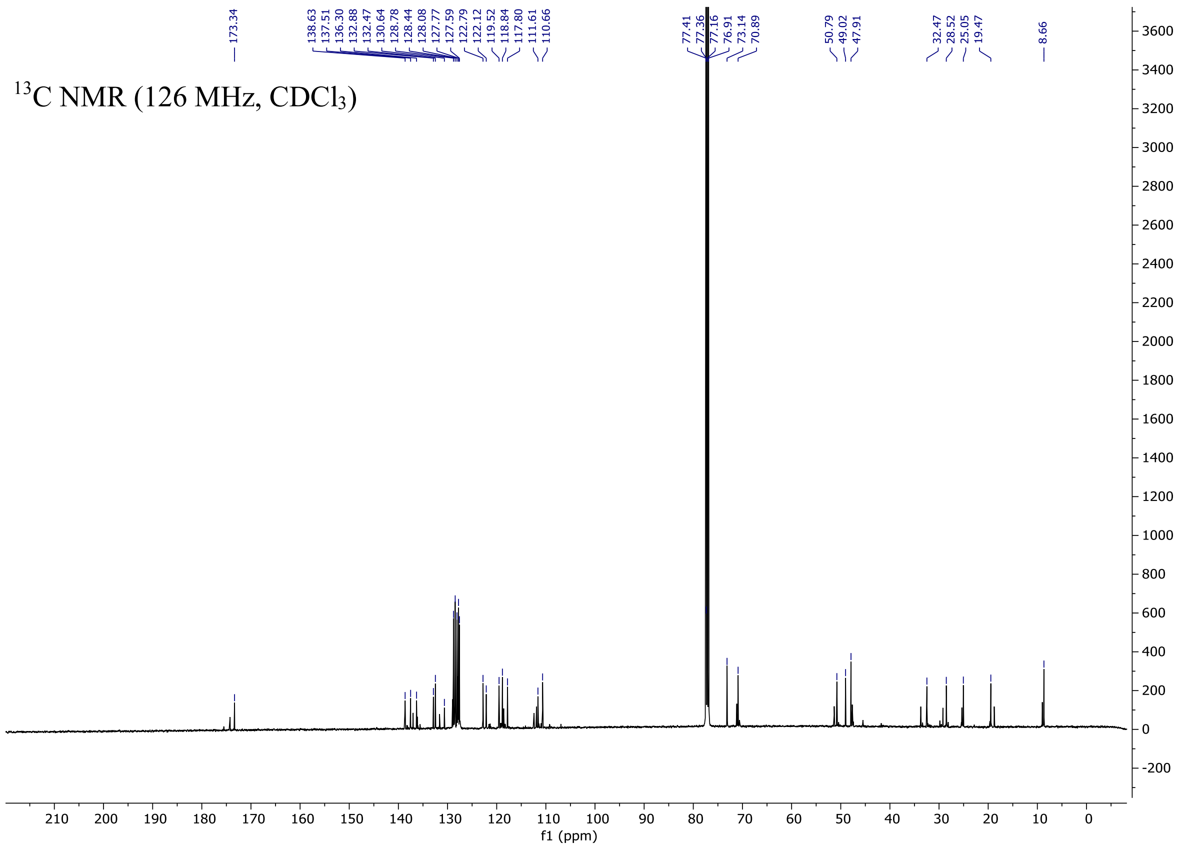

Compound 42:

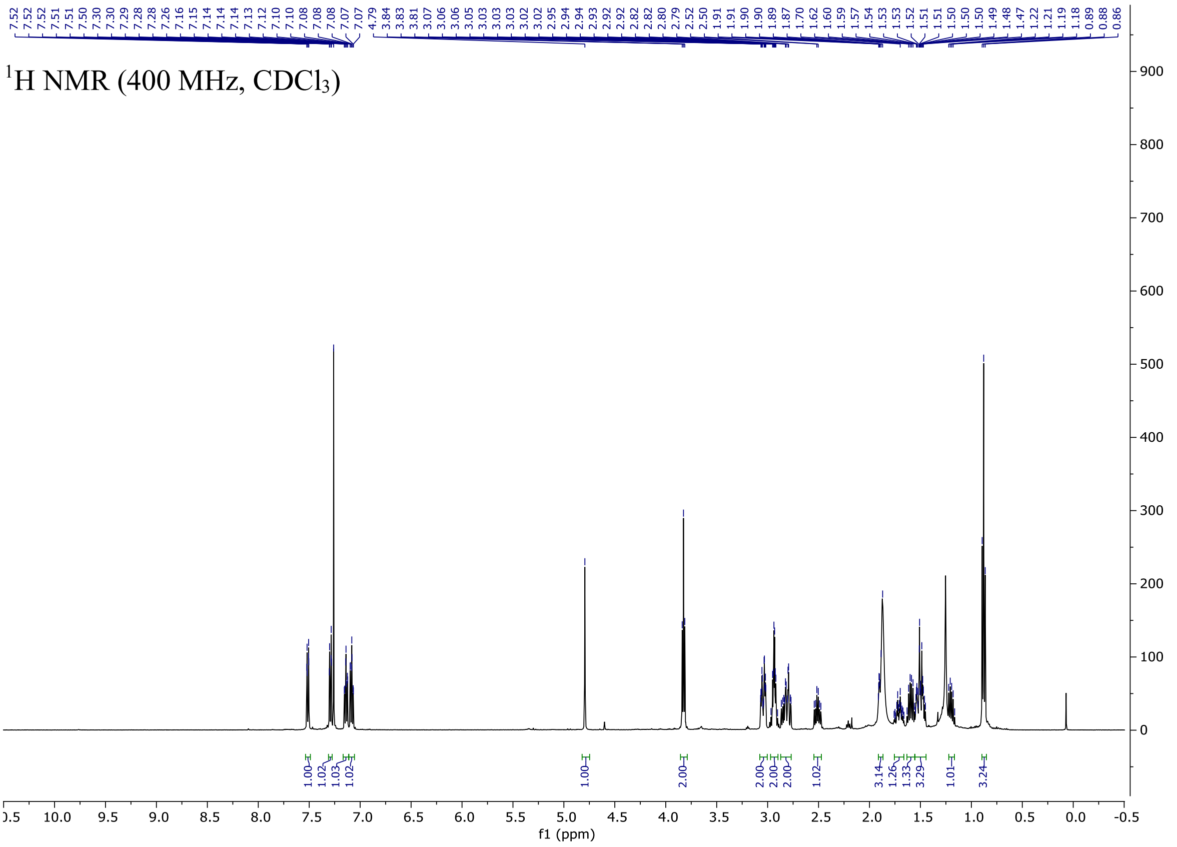

1H NMR (400 MHz, CDCl3)
f1 (ppm)

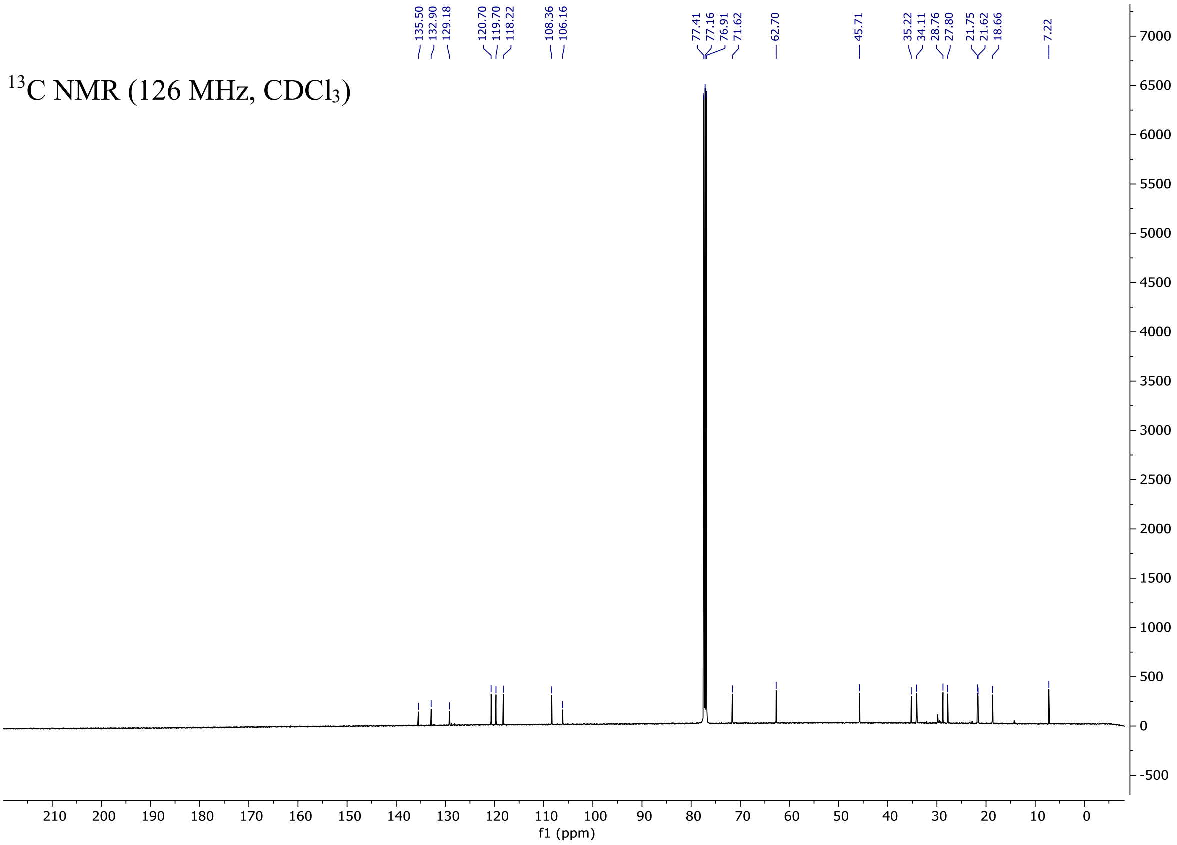

13C NMR (126 MHz, CDCl3)
135.50
132.90
129.18
120.70
119.70
118.22
108.36
106.16
77.41
77.16
76.91
71.62
62.70
45.71
35.22
34.11
28.76
27.80
21.75
21.62
18.66
7.22
f1 (ppm)
